# Global, regional and national trends in the burden of nutritional deficiencies in children, 1990–2021

**DOI:** 10.3389/fnut.2025.1565620

**Published:** 2025-07-02

**Authors:** Zhuoqiong Liu, Yangyang Liu, Jiaxin Yang, Liru Yan, Qiushi Li, Ying Gao

**Affiliations:** ^1^Department of Developmental Behavior Pediatrics, Harbin Medical University Affiliated Sixth Hospital, Harbin, China; ^2^Department of Pediatric Internal Medicine, Harbin Medical University Affiliated Sixth Hospital, Harbin, China

**Keywords:** nutritional deficiencies, children, disease burden, incidence, DALY

## Abstract

**Background:**

The objective of this study was to systematically assess global, regional, and national trends in the prevalence and burden of nutritional deficiencies among children aged 0–14 years from 1990 to 2021. Specifically, four major subtypes of malnutrition: protein-energy malnutrition, Vitamin A deficiency, iodine deficiency, and dietary iron deficiency.

**Methods:**

This study utilized data from the 2021 Global Burden of Disease (GBD) database to analyze incidence and disability-adjusted life year (DALY) rates. The estimated annual percentage change (EAPC) was calculated, with the data stratified based on age, gender, region, socio-demographic index (SDI), and country.

**Results:**

Between 1990 and 2021, the global burden of nutritional deficiencies (all ages 0–14 combined) in children declined. The age-standardized DALY rate of dietary iron deficiency had the lowest EAPC (− 0.53; 95% CI: − 0.62, − 0.45). The highest burden of nutritional deficiencies and its subtypes was in Sub-Saharan Africa, but the age-standardized DALY rate of dietary iron deficiency and the age-standardized incidence rate (ASIR) of protein-energy malnutrition were highest in South Asia (1103.19 and 3185.45 respectively). Age-standardized DALYs of protein-energy malnutrition increased substantially in high-income North America (EAPC: 3.30) and Western Europe (EAPC: 2.29). The age group with the greatest burden of nutritional deficiencies and of its subtypes was 0–4 years age group.

**Conclusion:**

From 1990 to 2021, Sub-Saharan Africa has consistently faced the most severe nutritional deficiencies. Meanwhile, South Asia continues to struggle with significant protein-energy and dietary iron nutritional shortfalls. Conversely, high-income North America and Western Europe have experienced a notable rise in protein-energy deficiency.

## 1 Introduction

According to the World Health Organization (WHO), nutritional deficiencies are defined as inadequate or excessive nutrient intake, essential nutrient imbalance, or impaired nutrient utilization (1). Four common deficiencies—protein-energy malnutrition (PEM) and micronutrient deficiencies (iron, Vitamin A, and iodine)—disproportionately affect children (2), critically impacting growth and development. Vitamin A deficiency is a major cause of childhood blindness and mortality, is also linked to infectious diseases, anemia, and reproductive health issues (3), affecting an estimated 190 million preschool children (4). PEM can lead to developmental delays, infections, cognitive deficits, and death. Iron deficiency primarily causes anemia, representing one of the most significant global disease burdens (5); remarkably, 41.7% of children under five years of age worldwide are iron deficient (6). The Global Nutrition Report 2020 lists iodine deficiency, Vitamin A deficiency, and PEM as major global risk factors for mortality and disability-adjusted life years (DALYs) (7). Although considerable progress has been made, these deficiencies persist as major health challenges, particularly in low- and middle-income countries (LMICs) (8), highlighting the urgent need for global nutritional improvement.

Human growth and development are critically vulnerable during the first 14 years. Nutritional deficiencies manifest as wasting, being underweight, and stunted growth. Affecting an estimated one-third of children worldwide under 5 years of age (9). Children aged 5–10 years are considered school-age, and those 10–14 years are pre-adolescents (10), However, the prevalence of nutritional deficiencies in children older than 5 years is rarely studied (11, 12), creating a significant knowledge gap. The WHO estimates that 230 million children worldwide between 0 and 14 years of age suffer from nutritional deficiencies, 90% of whom live in low-income countries (13). This age range encompasses critical developmental stages from infancy through pre-adolescence. In general, children up to 14 years fall under pediatrics due to their vulnerability related to food and dependence on adults. Yet, the lack of uniform terminology for the 0–14 age group reflects a bias toward younger children, leaving the magnitude of nutritional deficiencies in older children largely unknown (14).

Therefore, this study specifically focuses on the entire pediatric spectrum (0–14 years) to comprehensively quantify the burden across all critical developmental stages, filling this persistent research void. Examining deficiencies across this full spectrum is crucial for informing more targeted and developmentally appropriate public health strategies, health system planning, and nutrition programs. Understanding distinct burden profiles by age allows for the design of life-stage-specific interventions, from infant supplementation to school feeding and adolescent education. In 2015, the United Nations included eliminating all forms of malnutrition as a 2030 Sustainable Development Goal, elevating nutrition to a strategic priority. Given the limited reporting on the burden for the 0–14 age group, this analysis uses Global Burden of Disease 2021 data to quantify the global, regional, and national burden of nutritional deficiencies and its subtypes from 1990 to 2021. We aim to identify the main contributing subtypes in different regions to help develop targeted prevention and intervention strategies.

## 2 Materials and methods

### 2.1 Data sources

The data were obtained from Global Burden of Disease (GBD) 2021 database which is derived from 100,983 data sources including vital registration systems, verbal autopsy, census, household survey, disease registry, and health service linkage data. This database covers 204 countries and territories and 811 subnational districts and includes analysis of 371 diseases and injuries. The data were retrieved for the years 1990–2021 by region, country, and major nutritional deficiencies (vitamin A deficiency, dietary iron deficiency, iodine deficiency, and protein-energy malnutrition). We extracted data on annual incident cases, incidence rates, DALYs and rates, and their 95% uncertainty intervals (UIs) for nutritional deficiencies in children aged 0–14 years. Incidence rates and DALY rates were reported separately for both sexes across four age groups: <1 year, 1–4 years, 5–9 years, and 10–14 years. Data extraction was conducted using GBD Results Tool (GHDx)^1^. A waiver of informed consent has been approved by the University of Washington Institutional Review Board to use identified data in GBD study. No individual participants were involved in this study. Ethical approval reference number: https://www.healthdata.org/.

The GBD study employs rigorous statistical methods (e.g., DisMod-MR, spatiotemporal Gaussian process regression, ensemble models) and extensive data processing to address missing data, model estimates for locations/years with sparse data, and quantify uncertainty. Data availability varied by location, year, and deficiency; however, the GBD methodology aims to produce complete and comparable time series for all 204 countries and territories from 1990 to 2021, leveraging all available sources and accounting for data gaps through modeling.

### 2.2 Case definitions and variable selection

Four nutritional deficiency subtypes were modeled in GBD 2021: protein-energy malnutrition (PEM), Vitamin A deficiency, iodine deficiency, and dietary iron deficiency. These specific deficiencies were chosen as they represent the major nutrition-related risk factors for mortality and morbidity in children aged 0–14 years according to WHO and the Global Nutrition Report (7), and are consistently prioritized in global nutrition initiatives. While GBD models other micronutrient deficiencies (e.g., zinc, folate, vitamin D), this study focuses on these core four due to their established high burden and policy relevance for the target pediatric population.

*PEM*: Included moderate and severe acute malnutrition (also known as wasting), defined by weight loss relative to height. PEM burden (non-fatal) was disaggregated into four groups: moderate wasting without oedema (WHZ < −2SD to < −3SD), moderate wasing with oedema (WHZ < −2SD to < −3SD), severe wasting without oedema (WHZ < −3SD), and severe wasting with oedema (WHZ < −3SD). Defined using WHO 2006 growth standards (weight-for-height Z-scores, WHZ).

*Iodine deficiency*: Estimated non-fatal burden only, comprising visible goitre (grade 2) and sequelae (thyroid dysfunction, heart failure, intellectual disability). Sub-clinical deficiency and grade 1 goitre were excluded.

*Vitamin A deficiency*: Defined as serum retinol concentration < 0.7 μmol/L.

*Dietary iron deficiency*: Defined as mild, moderate, or severe anemia specifically attributed to inadequate dietary iron intake, excluding other causes of iron deficiency.

We only estimated the non-fatal burden of iodine deficiency as visible goitre (grade 2) and its sequelae (thyroid dysfunction, heart failure, intellectual disability (historically known as cretinism)), but not sub-clinical iodine deficiency or non-visible goitre (grade 1) due to iodine deficiency. Vitamin A deficiency is defined in GBD 2021 as serum retinol concentration < 0.7 μmol/L. Dietary iron deficiency used in the GBD cause analysis is defined as mild, moderate, or severe anemia due to inadequate dietary iron intake, and excludes other causes of inadequate absolute or functional iron availability for the body’s needs (13).

Nutritional deficiencies were categorized using ICD-10 codes: PEM (E40-E46.9, E64.0), iodine deficiency (E00-E02), dietary iron deficiency (D50-D50.9), Vitamin A deficiency (E50-E50.9, E64.1), and other nutritional deficiencies (D51-D53.9, E51-E61.9, E63-E64, E64.2-E64.9) (15).

To explore the relationship between socioeconomic development and nutritional burden, we used the Socio-demographic Index (SDI). SDI is a composite index (0–1) combining income per capita, average years of schooling (≥15 years), and total fertility rate (<25 years). Countries were grouped into five SDI quintiles: low (<0.455), low-middle (0.455–0.608), middle (0.608–0.689), high-middle (0.689–0.805), and high (>0.805) (16, 17).

### 2.3 Statistical analysis

To quantify the global burden of nutritional deficiencies among children aged 0–14 years, we analyzed incidence rates, DALY rates, and estimated annual percentage change (EAPC). Rates and 95% UIs were sourced directly from GBD 2021, calculated using DisMod-MR and other GBD modeling tools. All rates are per 100,000 person-years.

The EAPC measures the average annual trend in a rate over the study period (1990–2021). It was calculated by fitting a linear regression model to the natural logarithm of the rate (y) over calendar year (x): y = a + *β*x + *ε*, where y is ln(rate), x is calendar year, and ε is the error term. The EAPC is derived as 100 × (exp(β) − 1). The 95% confidence interval (CI) for the EAPC was calculated from the standard error (SE) of the β coefficient. An EAPC with a 95% CI not crossing zero was considered statistically significant (*p* < 0.05). Analyses were conducted for the overall 0–14 year age group and stratified by the specific age groups (<1 year, 1–4 years, 5–9 years, 10–14 years) and sex.

Statistical operations were performed using the dplyr package in R software (version 4.2.0).

## 3 Results

### 3.1 Overall nutritional deficiencies

**Figure 1:**
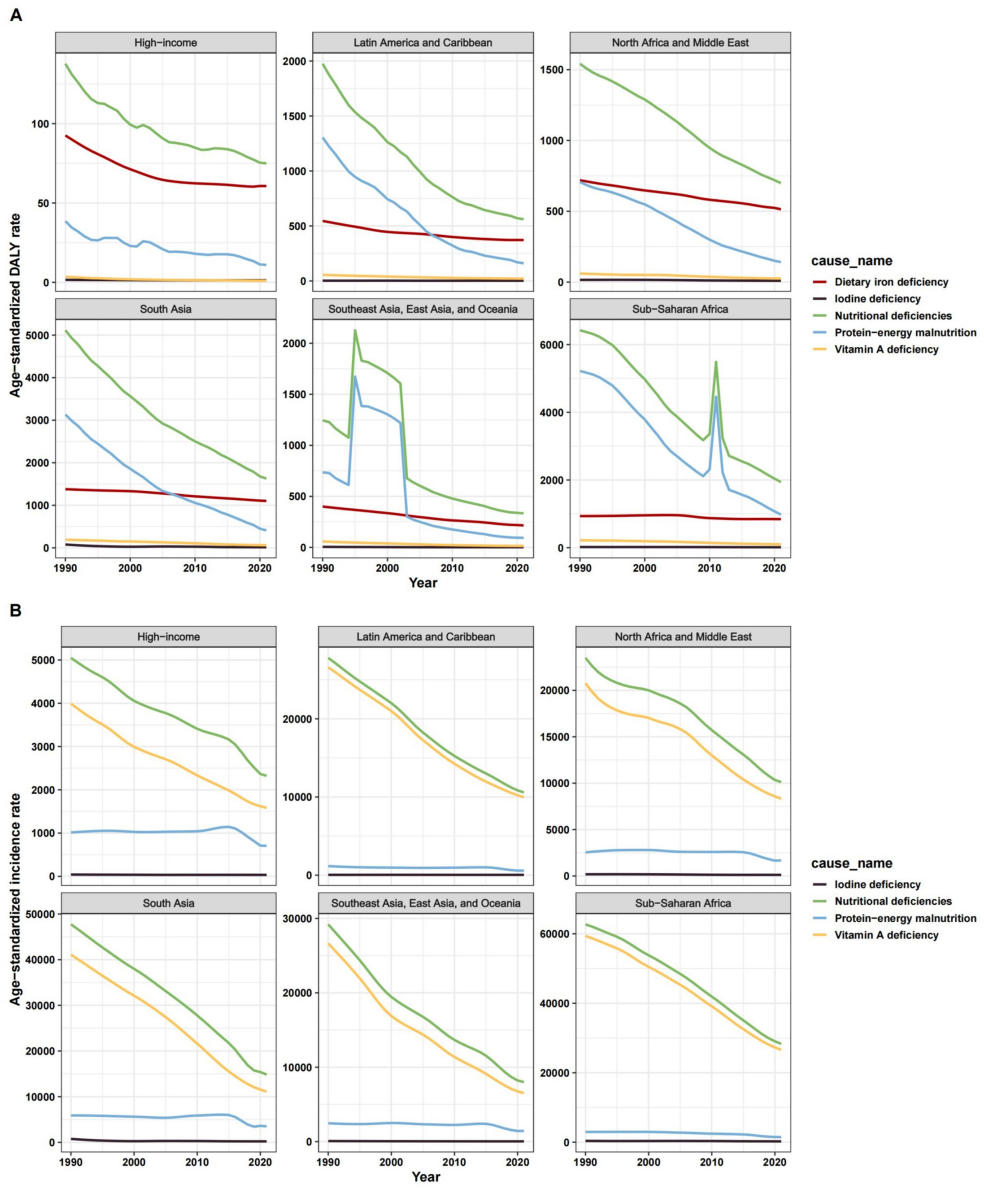
ASR of change trend for nutritional deficiencies and its subtypes among children aged 0–14 years old in 7 super regions, 1990–2021. **(A)** Age-standardized DALY rate. **(B)** Age-standardized incidence rate. ASR, age-standardized rate.

**Figure 2:**
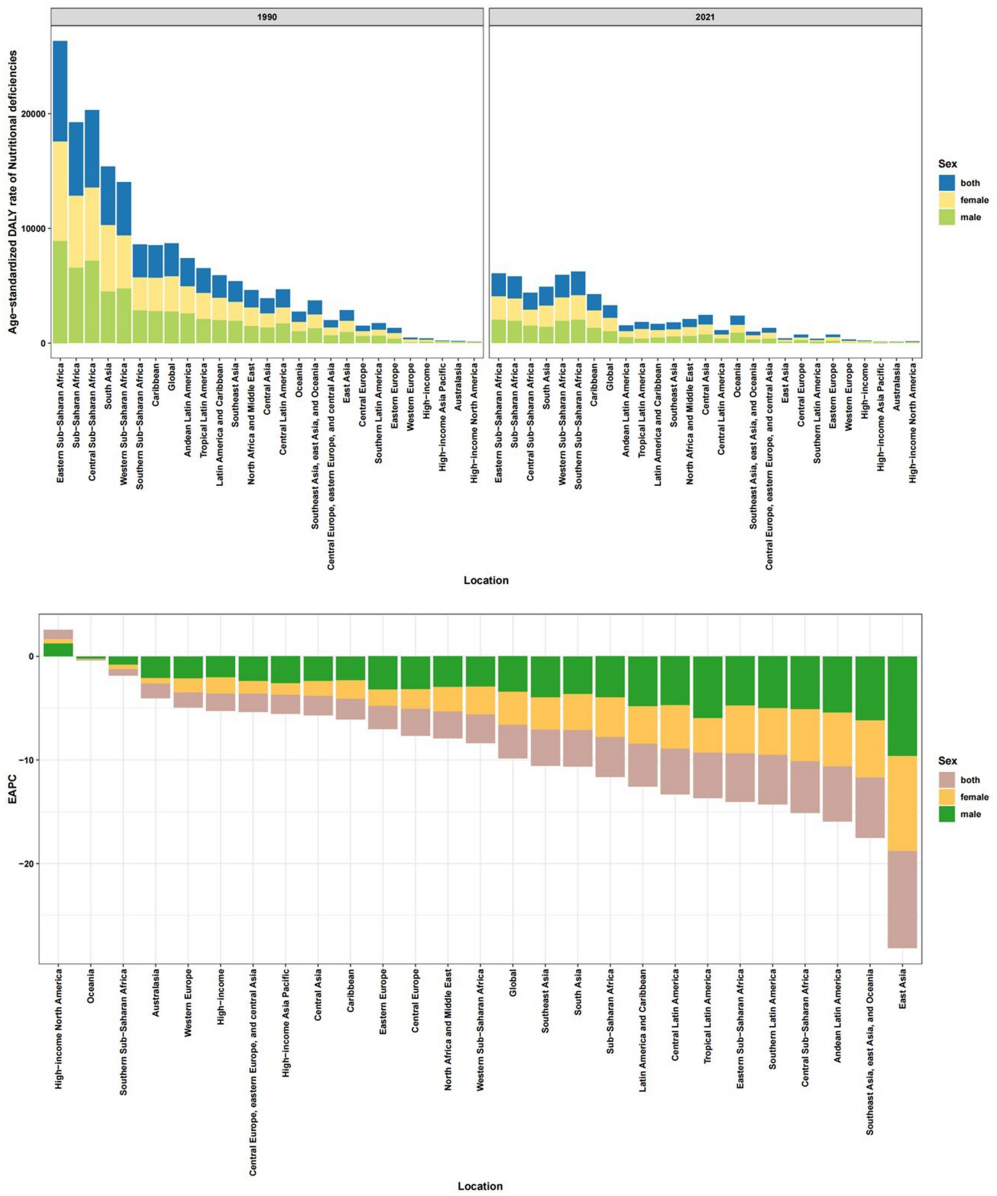
Age-standardized DALY rate of nutritional deficiencies among children aged 0–14 years old in 21 regions, in 2021 and its trends from1990 to 2021.

Globally, nutritional deficiencies among children aged 0–14 years declined significantly from 1990 to 2021, with the age-standardized DALY rate dropping to 1,094.15 (95% UI: 1,093.66–1,094.65) per 100,000 and an incidence rate of 11,563.37 (95% UI: 11,561.77–11,564.97). The EAPC reflected steady declines, with DALY rates decreasing by −3.28 (95% CI: −3.58, −2.98) and incidence rates by −2.66 (95% CI: −2.89, −2.43). This trend was consistent across all five socioeconomic-demographic index (SDI) regions and seven super-regions (Figure 1, Supplementary Figure 1A), though progress varied significantly. Sub-Saharan Africa (SSA) bore the highest burden in 2021, with Southern SSA (2,079.16), Eastern SSA (2,032.43), and Western SSA (1,982.19) reporting the highest DALY rates. In contrast, high-income regions like High-income Asia Pacific (41.25), Australasia (42.16), and High-income North America (51.30) had the lowest burdens (Figure 2). Notably, High-income North America experienced a significant increase in DALY rates (EAPC: 0.90), contrasting sharply with the most rapid declines in East Asia (EAPC: −9.39) and Andean Latin America (EAPC: −5.31) (Figure 1; Supplementary Tables 1, 2; Supplementary Figure 1A).

**Figure 3:**
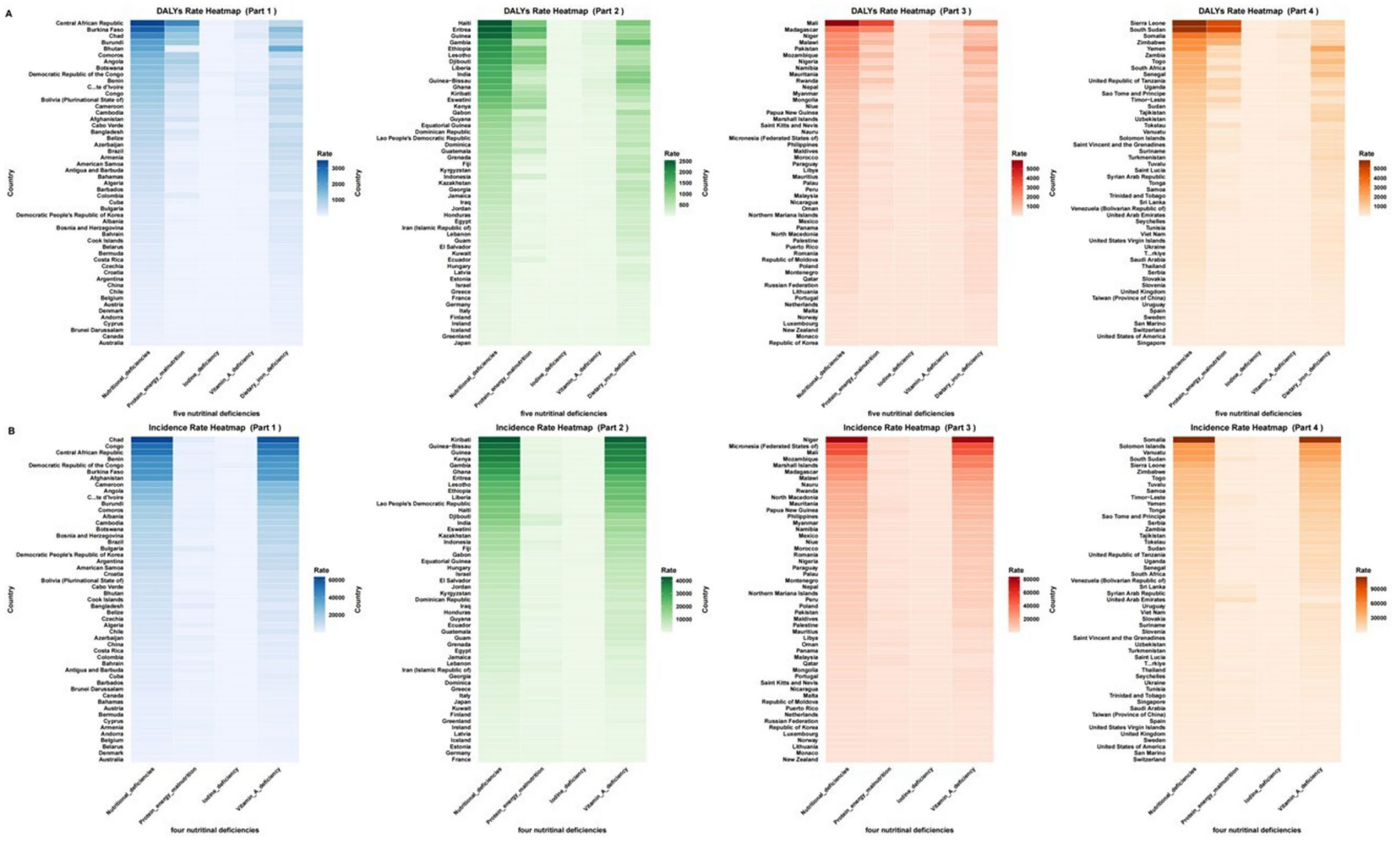
The age-standardized DALY rate and ASIR of nutritional deficiencies and its subtypes among children aged 0–14 years old among 204 countries in 2021. **(A)** Age-standardized DALY rate. **(B)** Age-standardized incidence rate. ASR, age-standardized rate.

At the country level, Mali (5,885.92), Sierra Leone (5,871.21), and South Sudan (5,640.66) had the highest 2021 DALY rates, while Singapore (30.68), Republic of Korea (34.38), and Australia (38.00) reported the lowest (Supplementary Tables 1, 2; Figures 3A,B). Somalia led in 2021 incidence rates (84,789.58), whereas Australia had the lowest (426.26). Countries like DPR Korea, Bangladesh, and Angola achieved the greatest reductions since 1990, while Cameroon, the USA, and Zimbabwe saw rising DALY rates.

### 3.2 Protein-energy malnutrition (PEM)

Global PEM burden declined but remained substantial, particularly in vulnerable regions. In 2021, the age-standardized DALY rate was 403.98 (95% UI: 403.68–404.29), with an incidence rate of 1,684.50 (95% UI: 1,683.87–1,685.13). Progress was slowest in high-SDI regions, with EAPCs of −5.05 (95% CI: −5.60, −4.51) for DALY rates and −1.23 (95% CI: −1.71, −0.75) for incidence rates. Eastern SSA (1,231.09), Southern SSA (1,070.60), and Central SSA (826.96) had the highest 2021 PEM DALY rates, while Australasia (0.82) and High-income Asia Pacific (3.10) reported the lowest (Figure 1; Supplementary Tables 3, 4; Supplementary Figure 2B). South Asia (3,185.45) and Oceania (2,497.73) faced the highest incidence rates. Alarmingly, High-income North America (EAPC: 3.30) and Western Europe (EAPC: 2.29) saw rising PEM DALY rates, while Oceania experienced increasing incidence (EAPC: 0.54). East Asia achieved the steepest reduction (EAPC: −15.54) (Supplementary Tables 3, 4; Supplementary Figure 3B).

Country-level extremes included South Sudan (4,868.93) and Sierra Leone (4,819.92) as the hardest-hit nations. Greece (EAPC: 18.49) and Andorra (EAPC: 8.77) showed alarming increases, whereas DPR Korea (EAPC: −18.18) had the sharpest decline. The UAE (5,795.02) and South Sudan (3,581.20) reported the highest incidence rates (Supplementary Tables 3, 4; Figures 3A,B).

### 3.3 Vitamin A deficiency (VAD)

Global VAD burden decreased, but SSA remained disproportionately affected. The 2021 age-standardized DALY rate was 46.81 (95% UI: 46.71–46.91), with an incidence rate of 9,771.55 (95% UI: 9,770.08–9,773.02). Progress was slowest in low-SDI regions (EAPC: −2.65 for DALYs; −2.90 for incidence). Central SSA (DALY: 114.85; incidence: 28,262.72), Eastern SSA, and Western SSA carried the highest 2021 burdens, while Australasia and High-income North America had negligible rates. Stagnation occurred in the Caribbean (DALY EAPC: −0.96), Oceania, and Central SSA, whereas East Asia achieved the steepest declines (DALY EAPC: −5.20; incidence EAPC: −5.54) (Figure 1; Supplementary Tables 5, 6; Supplementary Figures 4C, 5C).

Somalia (DALY: 359.62; incidence: 81,459.07), Niger, and the Central African Republic had the highest 2021 burdens. Minimal progress was observed in Somalia, Northern Mariana Islands, and Kyrgyzstan (EAPC ≈ − 0.5), while Maldives and Equatorial Guinea showed the fastest declines (EAPC ≈ − 7.5) (Supplementary Tables 5, 6; Figures 3A,B).

### 3.4 Iodine deficiency (IDD)

IDD had the lowest burden among deficiencies but showed sluggish progress, particularly in high-income settings. The 2021 age-standardized DALY rate was 8.14 (95% UI: 8.10–8.18), with an incidence rate of 107.32 (95% UI: 107.18–107.46). EAPCs were −2.70 (95% CI: −3.19, −2.22) for DALYs and −1.25 (95% CI: −1.62, −0.88) for incidence, with minimal improvements in high-SDI regions and Latin America & Caribbean. Central SSA (DALY: 53.50; incidence: 763.31) and South Asia (DALY: 14.15; incidence: 175.79) had the highest 2021 burdens, while Andean Latin America and Oceania reported the lowest. High-income North America (DALY EAPC: −0.07), Australasia, and Central Latin America (incidence EAPC: −0.05) showed negligible declines, whereas Southeast Asia achieved the largest DALY reduction (EAPC: −5.29) (Figure 1; Supplementary Tables 7, 8; Supplementary Figures 6D, 7D).

Democratic Republic of the Congo (DALY: 66.08; incidence: 932.40), Somalia, and Comoros had the highest 2021 burdens. Increases occurred in Madagascar (DALY EAPC: 1.54) and Nepal (incidence EAPC: 0.46), while Sri Lanka (DALY EAPC: −8.73) and Malaysia (incidence EAPC: −6.70) achieved major reductions (Supplementary Tables 7, 8).

### 3.5 Dietary Iron deficiency (DID)

DID was the leading cause of nutrition-related DALYs, but global progress was modest (EAPC: -0.53; 95% CI: −0.62, −0.45). South Asia (1,103.19), Western SSA (1,065.50), and Southern SSA (955.13) had the highest 2021 DALY rates, while high-income regions had the lowest. Oceania (EAPC: 0.43), Western SSA (EAPC: 0.25), and the Caribbean (EAPC: 0.14) experienced rising burdens, contrasting with East Asia’s steepest decline (EAPC: −4.14) (Figure 1; Supplementary Table 9; Supplementary Figure 8E).

Yemen (2,122.26), Mali (1,837.87), and Bhutan (1,815.98) had the highest 2021 burdens. Burkina Faso (EAPC: 2.39), Togo (EAPC: 1.70), and Mali (EAPC: 1.68) saw alarming increases, while Chile (EAPC: −4.25) had the sharpest reduction (Supplementary Table 9).

### 3.6 Gender and age distribution

Females bore a higher overall DALY burden in 2021, while males had higher incidence rates. Males also experienced larger reductions from 1990–2021. Deficiency-specific patterns emerged: PEM and VAD were more prevalent in males, whereas IDD and DID imposed higher DALYs on females. Age stratification revealed a sharp decline in overall DALY burden with age, peaking in infants (<1 year) for all deficiencies combined, PEM, and DID. VAD incidence peaked in 1–4-year-olds, while IDD showed an age-increasing DALY burden, peaking in 10–14-year-olds. VAD DALY rates also peaked in 1–4-year-olds (Supplementary Tables 10, 11, Supplementary Figures 9, 10).

### 3.7 Relationship with SDI

**Figure 4:**
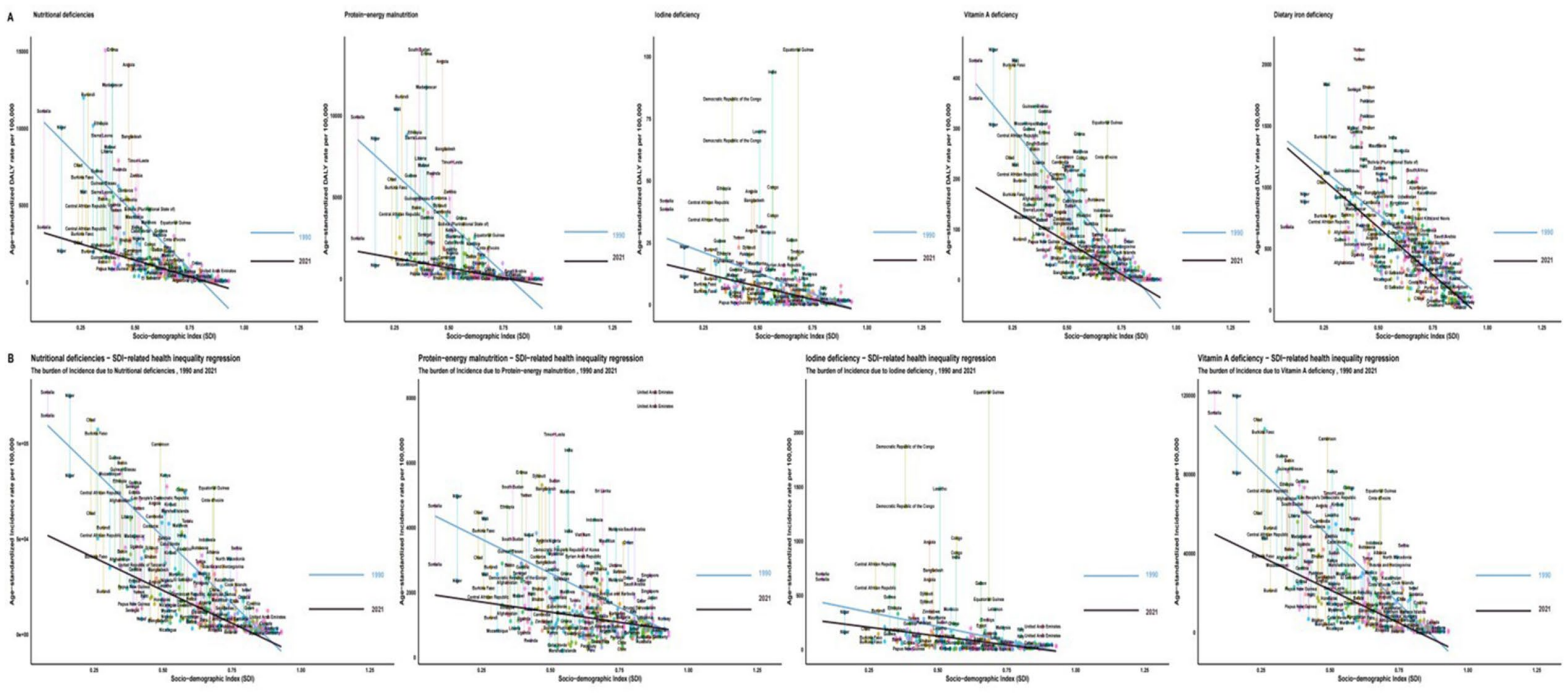
SDI-related health inequality regression for the burden of ASR due to five nutritional deficiencies among children aged 0–14 years old, 1990 and 2021. **(A)** Age-standardized DALY rate. **(B)** Age-standardized incidence rate. ASR, age-standardized rate; SDI, socio-demographic index.

A strong inverse correlation between SDI and nutritional deficiency burden persisted in 2021, with low-SDI regions bearing the highest burdens across all deficiency types: overall (DALY: 2,097.23; incidence: 23,757.40), PEM (DALY: 967.16), VAD (DALY: 112.31; incidence: 21,561.59), IDD (DALY: 11.54; incidence: 213.04), and DID (DALY: 982.19). Regression analysis confirmed this gradient, which flattened slightly in 2021 compared to 1990, suggesting slow progress in reducing absolute inequities over time (Tables 1, 2; Figure 4).

**Table 1:** DALY and its change trend of five common nutritional deficiencies among children aged 0–14 years old in five SDI regions, 1990–2021.

	Nutritional deficiencies	Protein-energy malnutrition	Vitamin A deficiency	Iodine deficiency	Dietary iron deficiency
Global					
Number of DALY in 2021	22777008.08 (17434382.12, 29675587.80)	8615187.61 (6818257.96, 10357178.71)	947378.26 (598906.03, 1357092.55)	167546.80 (91955.21, 276135.81)	12752951.82 (8426415.00, 18788407.42)
Change rate, 1990 to 2021 (%)	−59.57104322	−77.82806904	−49.51480018	−60.1280162	−2.119274276
Age-standardized DALY rate in 2021 (per 100,000)	1094.15 (1093.66, 1094.65)	403.98 (403.68, 404.29)	46.81 (46.71, 46.91)	8.14 (8.10, 8.18)	621.02 (620.66, 621.39)
EAPC, 1990 to 2021 (per 100,000)	−3.28 (−3.58, −2.98)	−5.05 (−5.60, −4.51)	−2.65 (−2.88, −2.42)	−2.70 (−3.19, −2.22)	−0.53 (−0.62, −0.45)
High SDI					
Number of DALY in 2021	159295.60 (108333.31, 230562.33)	22058.58 (14693.33, 32865.18)	1359.23 (828.21, 2056.04)	2103.36 (795.03, 4007.24)	131539.00 (85257.57, 194563.90)
Change rate, 1990 to 2021 (%)	−51.32977372	−61.76411349	−83.27811775	−14.27744679	−48.25513232
Age-standardized DALY rate in 2021 (per 100,000)	93.60 (93.11, 94.09)	12.53 (12.36, 12.70)	0.80 (0.75, 0.85)	1.15 (1.11, 1.21)	77.90 (77.45, 78.35)
EAPC, 1990 to 2021 (per 100,000)	−1.86 (−2.01, −1.70)	−1.87 (−2.19, −1.54)	−5.39 (−5.75, −5.03)	−0.42 (−0.48, −0.37)	−1.83 (−2.01, −1.65)
High-middle SDI					
Number of DALY in 2021	506848.37 (349753.73, 719279.10)	67616.53 (54864.19, 81309.52)	15113.07 (9137.65, 21755.34)	6380.61 (3111.03, 11182.35)	409033.73 (265282.59, 610485.33)
Change rate, 1990 to 2021 (%)	−74.18512308	−91.68040448	−77.63815058	−56.94181075	−59.33425793
Age-standardized DALY rate in 2021 (per 100,000)	221.88 (221.22, 222.54)	29.85 (29.60, 30.11)	6.63 (6.52, 6.75)	2.62 (2.55, 2.68)	178.86 (178.28, 179.45)
EAPC, 1990 to 2021 (per 100,000)	−3.86 (−4.00, −3.72)	−7.26 (−7.50, −7.02)	−4.41 (−4.52, −4.29)	−2.48 (−2.60, −2.35)	−2.52 (−2.61, −2.42)
Middle SDI					
Number of DALY in 2021	3448974.02 (2492621.85, 4749777.59)	871499.04 (732852.57, 1041726.58)	101373.47 (63816.68, 144682.22)	19626.57 (9164.99, 35467.56)	2405147.05 (1602875.79, 3540148.47)
Change rate, 1990 to 2021 (%)	−67.17043361	−85.53395841	−71.76255697	−77.08431185	−33.37658852
Age-standardized DALY rate in 2021 (per 100,000)	606.63 (605.93, 607.32)	153.39 (153.02, 153.75)	18.08 (17.96, 18.20)	3.29 (3.25, 3.34)	422.53 (421.96, 423.11)
EAPC, 1990 to 2021 (per 100,000)	−3.12 (−3.16, −3.07)	−5.38 (−5.51, −5.24)	−3.87 (−4.00, −3.75)	−4.39 (−4.84, −3.94)	−1.16 (−1.24, −1.08)
Low-middle SDI					
Number of DALY in 2021	7975534.20 (5967231.11, 10779230.17)	2399261.04 (1987073.72, 2880495.09)	297716.78 (186920.42, 438832.43)	61191.69 (33761.01, 99819.94)	5078995.76 (3402989.04, 7432217.44)
Change rate, 1990 to 2021 (%)	−66.5490754	−85.20497109	−63.6869554	−73.43665289	−5.519584217
Age-standardized DALY rate in 2021 (per 100,000)	1325.89 (1324.89, 1326.89)	383.35 (382.80, 383.90)	51.24 (51.05, 51.44)	10.29 (10.21, 10.38)	858.20 (857.40, 859.00)
EAPC, 1990 to 2021 (per 100,000)	−4.21 (−4.54, −3.88)	−6.71 (−7.29, −6.13)	−3.87 (−4.06, −3.68)	−4.27 (−4.83, −3.71)	−0.86 (−0.93, −0.78)
Low SDI					
Number of DALY in 2021	10671191.32 (8326069.40, 13612294.36)	5248184.76 (3923165.17, 6561350.08)	531213.06 (345647.80, 760078.09)	78189.06 (45336.57, 129071.50)	4720421.51 (3111023.15, 6848713.85)
Change rate, 1990 to 2021 (%)	−45.74525358	−66.62355465	−14.43581321	−9.986551891	70.12330236
Age-standardized DALY rate in 2021 (per 100,000)	2097.23 (2095.84, 2098.62)	967.16 (966.21, 968.11)	112.31 (111.98, 112.64)	17.54 (17.42, 17.67)	982.19 (981.24, 983.14)
EAPC, 1990 to 2021 (per 100,000)	−3.83 (−4.24, −3.40)	−5.21 (−5.85, −4.57)	−2.83 (−3.04, −2.63)	−2.10 (−2.45, −1.74)	−0.58 (−0.65, −0.51)

SDI, socio-demographic index.

**Table 2:** Incidence and its change trend of four common nutritional deficiencies among children aged 0–14 years old in five SDI regions, 1990–2021.

	Nutritional deficiencies	Protein-energy malnutrition	Vitamin A deficiency	Iodine deficiency
Global				
Number of DALY in 2021	231618972.25 (218064930.54, 246907501.12)	34509841.70 (25924285.50, 44393403.88)	194929059.92 (183819457.37, 208018179.15)	2180070.62 (1627522.15, 2739886.49)
Change rate, 1990 to 2021 (%)	−51.50867703	−38.60738247	−53.37135555	−35.74606072
ASIR in 2021 (per 100,000)	11563.37 (11561.77, 11564.97)	1684.50 (1683.87, 1685.13)	9771.55 (9770.08, 9773.02)	107.32 (107.18, 107.46)
EAPC, 1990 to 2021 (per 100,000)	−2.66 (−2.89, −2.43)	−1.23 (−1.71, −0.75)	−2.90 (−3.12, −2.69)	−1.25 (−1.62, −0.88)
High SDI				
Number of DALY in 2021	2537380.07 (2190544.06, 2926107.78)	1237993.50 (924649.52, 1593213.80)	1260780.74 (1128095.04, 1418480.38)	38605.83 (24945.76, 52745.04)
Change rate, 1990 to 2021 (%)	−64.49349499	−34.59291671	−75.79627809	−13.15158718
ASIR in 2021 (per 100,000)	1469.75 (1467.84, 1471.67)	710.69 (709.36, 712.02)	737.18 (735.82, 738.54)	21.89 (21.67, 22.11)
EAPC, 1990 to 2021 (per 100,000)	−2.78 (−2.92, −2.64)	−0.45 (−0.80, −0.10)	−4.34 (−4.40, −4.29)	−0.33 (−0.37, −0.29)
High-middle SDI				
Number of DALY in 2021	8428116.42 (7618736.64, 9434564.37)	2188590.33 (1669345.80, 2852359.57)	6139851.70 (5494166.73, 6883884.00)	99674.39 (71978.52, 128109.06)
Change rate, 1990 to 2021 (%)	−74.72901627	−49.14209306	−78.72284202	−47.83608892
ASIR in 2021 (per 100,000)	3690.76 (3688.11, 3693.41)	967.36 (965.97, 968.75)	2681.84 (2679.60, 2684.08)	41.56 (41.30, 41.83)
EAPC, 1990 to 2021 (per 100,000)	−3.75 (−3.87, −3.62)	−0.82 (−1.14, −0.49)	−4.46 (−4.60, −4.33)	−1.81 (−1.86, −1.76)
Middle SDI				
Number of DALY in 2021	37300995.48 (34089736.68, 40732020.70)	8458503.09 (6492536.61, 10953092.93)	28490744.83 (25894671.02, 31091337.53)	351747.56 (262599.68, 447283.67)
Change rate, 1990 to 2021 (%)	−71.18633237	−45.56782866	−74.83938135	−48.34156368
ASIR in 2021 (per 100,000)	6703.31 (6701.00, 6705.61)	1542.26 (1541.12, 1543.40)	5101.08 (5099.08, 5103.07)	59.97 (59.77, 60.17)
EAPC, 1990 to 2021 (per 100,000)	−3.66 (−3.85, −3.47)	−1.00 (−1.45, −0.55)	−4.24 (−4.41, −4.07)	−1.56 (−1.95, −1.18)
Low-middle SDI				
Number of DALY in 2021	71860925.70 (65035717.45, 79879336.60)	12119868.16 (9085078.08, 15732814.63)	58999732.22 (52717691.08, 66080299.48)	741325.32 (560080.33, 935249.30)
Change rate, 1990 to 2021 (%)	−60.94773387	−47.22571357	−62.98232867	−55.45146437
ASIR in 2021 (per 100,000)	12494.62 (12491.51, 12497.74)	2072.39 (2071.09, 2073.70)	10295.57 (10292.75, 10298.38)	126.66 (126.37, 126.96)
EAPC, 1990 to 2021 (per 100,000)	−3.51 (−3.74, −3.28)	−1.69 (−2.19, −1.18)	−3.84 (−4.06, −3.62)	−2.57 (−3.08, −2.06)
Low SDI				
Number of DALY in 2021	111324542.56 (105893216.35, 117223145.20)	10482892.80 (7544788.34, 13707354.52)	99893580.94 (95160771.51, 104779753.05)	948068.81 (692719.03, 1186171.08)
Change rate, 1990 to 2021 (%)	−9.74781037	−8.695348261	−10.05070009	16.80807235
ASIR in 2021 (per 100,000)	23757.40 (23752.66, 23762.15)	1982.78 (1981.40, 1984.16)	21561.59 (21557.07, 21566.10)	213.04 (212.60, 213.47)
EAPC, 1990 to 2021 (per 100,000)	−2.58 (−2.80, −2.37)	−1.78 (−2.23, −1.33)	−2.67 (−2.88, −2.46)	−1.40 (−1.69, −1.10)

SDI, socio-demographic index; ASIR, Age-standardized incidence rate.

## 4 Discussion

Our analysis confirms a significant global decline in the burden of nutritional deficiencies among children aged 0–14 from 1990 to 2021, extending to all four deficiency subtypes examined. This encouraging trend underscores the cumulative impact of decades of public health efforts, including nutrition-sensitive agriculture, dietary diversification, improved micronutrient access, economic development, enhanced healthcare (especially prenatal care and vaccination), and targeted food supplementation programs (18, 19). However, this progress masks profound and persistent inequities. The burden remains overwhelmingly concentrated in low-resource settings, particularly SSA, South Asia, and Oceania, reflecting the powerful influence of socio-demographic factors. Crucially, vulnerable age groups bear the brunt of this burden, with children under 5 years old experiencing the highest DALY rates across all deficiencies, highlighting critical windows for intervention where resources must be prioritized.

Persistent Regional Inequities:the disproportionate burden of nutritional deficiencies in Sub-Saharan Africa and South Asia reflects systemic challenges, including poverty, food insecurity, weak healthcare infrastructure, and political instability (18). For instance, Southern Sub-Saharan Africa had the highest DALY rates for protein-energy malnutrition and vitamin A deficiency, exacerbated by conflict, climate shocks, and limited access to fortified foods (19). Conversely, high-income regions achieved the lowest rates through robust public health programs (e.g., universal salt iodization in East Asia) but face rising obesity-linked micronutrient gaps (e.g., iron deficiency in Oceania) (20). Rising protein-energy malnutrition in high-income North America and Western Europe may reflect ultra-processed diets and socioeconomic inequities (21, 22). For example, ultra-processed foods provide >50% of calories in the U. S., linking obesity to micronutrient deficiencies (23). These trends contrast sharply with reductions in low-SDI regions, emphasizing the need for policies addressing both undernutrition and overnutrition.

Detailed age stratification reveals distinct patterns beyond the under-5 focus common in literature: while DALY rates peak in infants (<1 year) for overall deficiencies, PEM, and IDA/DID, VAD incidence peaks in 1–4-year-olds, and IDD uniquely shows increasing DALY burden with age, peaking in 10–14-year-olds (24). This necessitates tailored interventions across childhood stages, prioritizing maternal nutrition and infant & young child feeding (IYCF) programs for <5-year-olds, while developing school-based meal programs and adolescent health initiatives addressing iron needs for older children (25). Gender disparities further complicate progress: females bear a higher overall DALY burden, but males exhibit greater PEM/VAD prevalence, while females face higher IDD/DID DALYs. Notably, males experienced larger reductions over time, suggesting potential gender disparities in intervention effectiveness or access (26). Addressing these inequities requires gender-sensitive approaches targeting cultural practices, intra-household food allocation biases, and healthcare access disparities (27).

A strong inverse relationship between SDI and nutritional deficiency burden persists across all subtypes, though recent trends show slight gradient flattening, indicating potential progress in reducing absolute inequities (28). For low-SDI regions, interventions should prioritize scaling up food fortification (e.g., iodized salt, iron), supplementation programs (e.g., vitamin A), promoting dietary diversity through nutrition-sensitive agriculture, improving WASH infrastructure, strengthening primary healthcare, and addressing systemic poverty through social protection programs (29). In high-income regions, policy shifts toward dietary quality equity are critical: implementing marketing restrictions and taxation on ultra-processed foods, promoting affordable healthy diets, integrating nutritional assessment into pediatric care, and addressing socioeconomic disparities affecting food access (30).

This GBD study fills a critical gap in understanding global, regional, and national trends in childhood nutritional deficiencies (0–14 years) from 1990 to 2021. However, GBD data may underestimate burdens in low-resource settings due to incomplete surveillance systems and misclassification risks (31). The analysis focuses on PEM, iron deficiency, VAD, and IDD, omitting other critical micronutrients (e.g., zinc, folate, vitamin B12). Modeling imputations in data-scarce regions introduce uncertainties, particularly for subtypes and smaller geographies (32). Age-standardization, while enabling comparisons, may obscure intra-group variations within the 0–14 cohort.

Critical research priorities include: (1) Investigating pathophysiological mechanisms linking ultra-processed diets to PEM in HICs and how economic disparities exacerbate dietary inequalities (33); (2) Developing scalable adolescent interventions (e.g., school-based micronutrient supplementation, gender-responsive dietary education) to address iron and iodine deficiencies, particularly in girls (34); (3) Identifying context-specific barriers (e.g., supply chain failures, conflict disruptions) hindering VAD and IDA/DID intervention scalability in stagnating regions like SSA and Oceania (35); (4) Unpacking socio-cultural drivers (e.g., intra-household food allocation biases) of gendered nutritional burdens to design transformative policies (36); and (5) Advancing surveillance methodologies to integrate neglected micronutrients (e.g., zinc, folate) into global burden assessments using standardized biomarkers and integrated surveys (37). These priorities address mechanistic, equity, and monitoring gaps essential for targeted policy action.

## 5 Conclusion

Although the prevalence of childhood nutritional deficiencies and their subtypes has declined worldwide since 1990, South Asia is still the region with the most serious protein-energy and dietary iron nutritional problems, while Sub-Saharan Africa has the highest micronutrient deficiency burden. Nutritional inequalities have not been fundamentally improved due to differences in social development levels. Therefore, targeted nutrition health promotion, dietary diversity, and medical support in low-income regions should be strengthened, alongside improved monitoring and evaluation systems.

## Data Availability

The original contributions presented in the study are included in the article/Supplementary material, further inquiries can be directed to the corresponding author.

## Footnotes

^1^ http://ghdx.healthdata.org/gbd-results-tool
