# Supplementary material for: Global, regional and national trends in the burden of nutritional deficiencies in children, 1990–2021
Source: Front Nutr. 2025 Jul 2;12:1565620. doi: 10.3389/fnut.2025.1565620 (PMC12263351; doi:10.3389/fnut.2025.1565620)
Supplement: Supplementary file 1 [file Image_1.pdf]

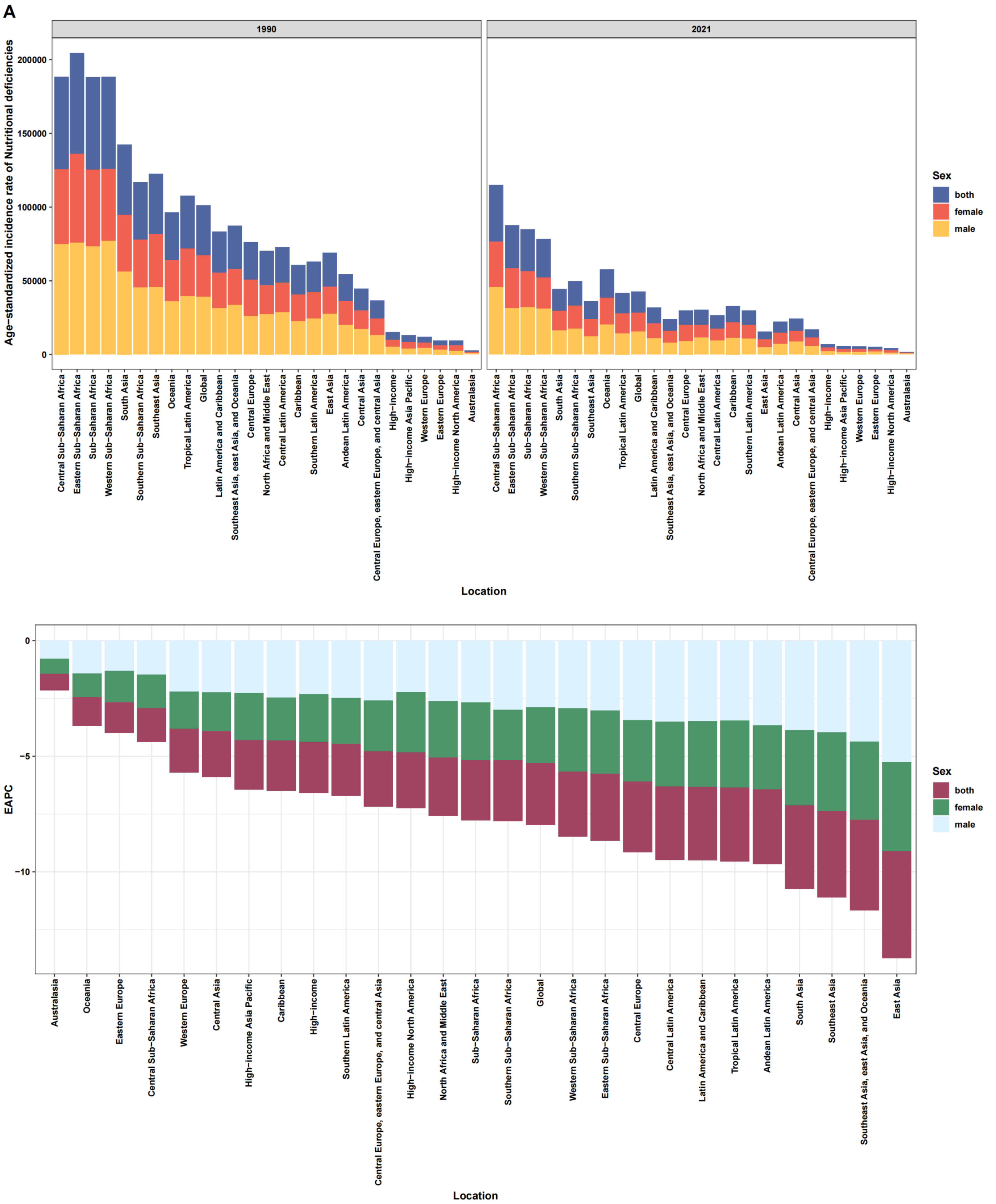

SFigure1 Age-standardized incidence rate of nutritional deficiencies among children aged 0-14 years old in 21 regions, in 2021 and its trends from1990 to 2021

B

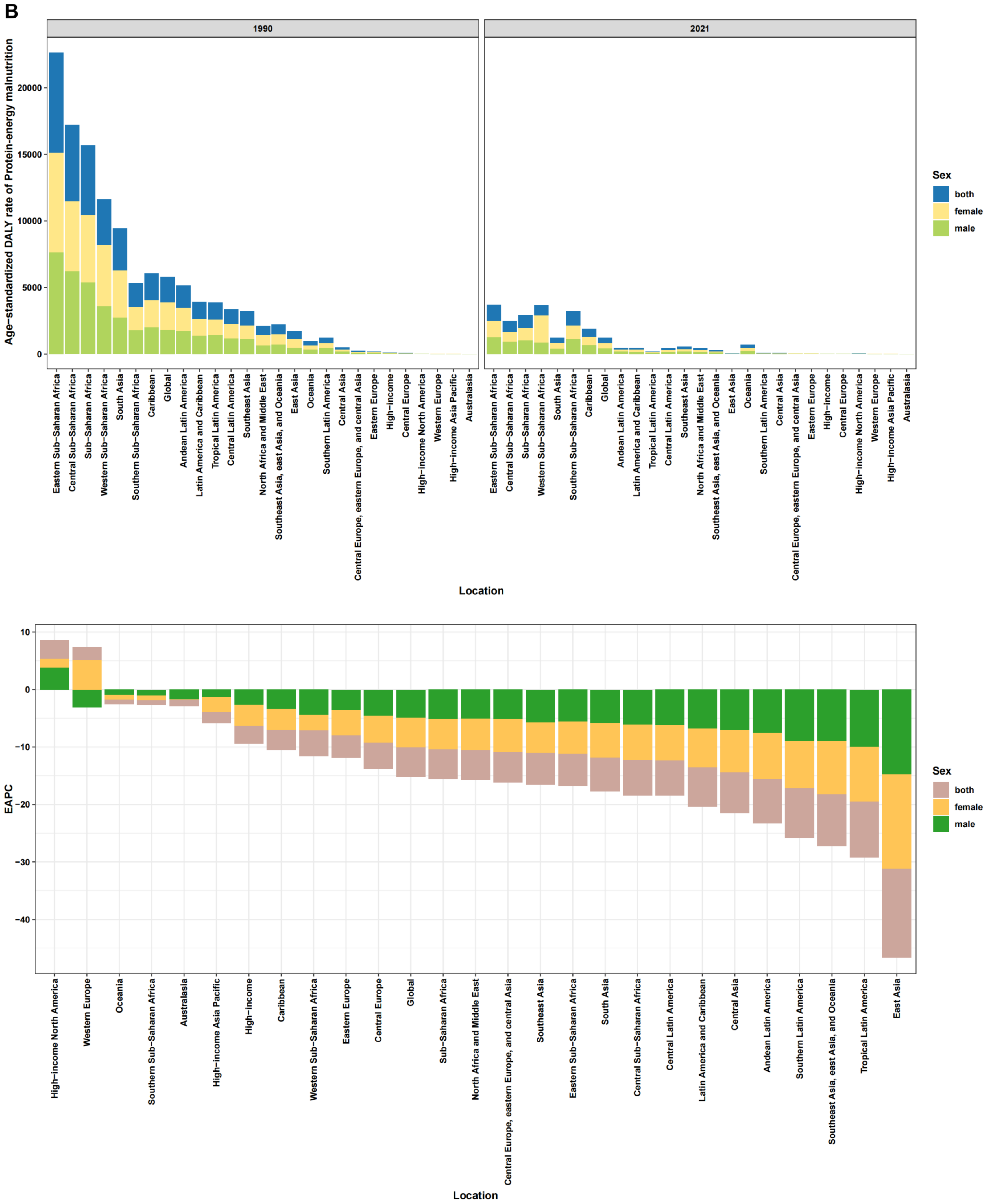

SFigure2 Age-standardized DALY rate of protein-energy malnutrition among children aged 0-14 years old in 21 regions, in 2021 and its trends from1990 to 2021

B

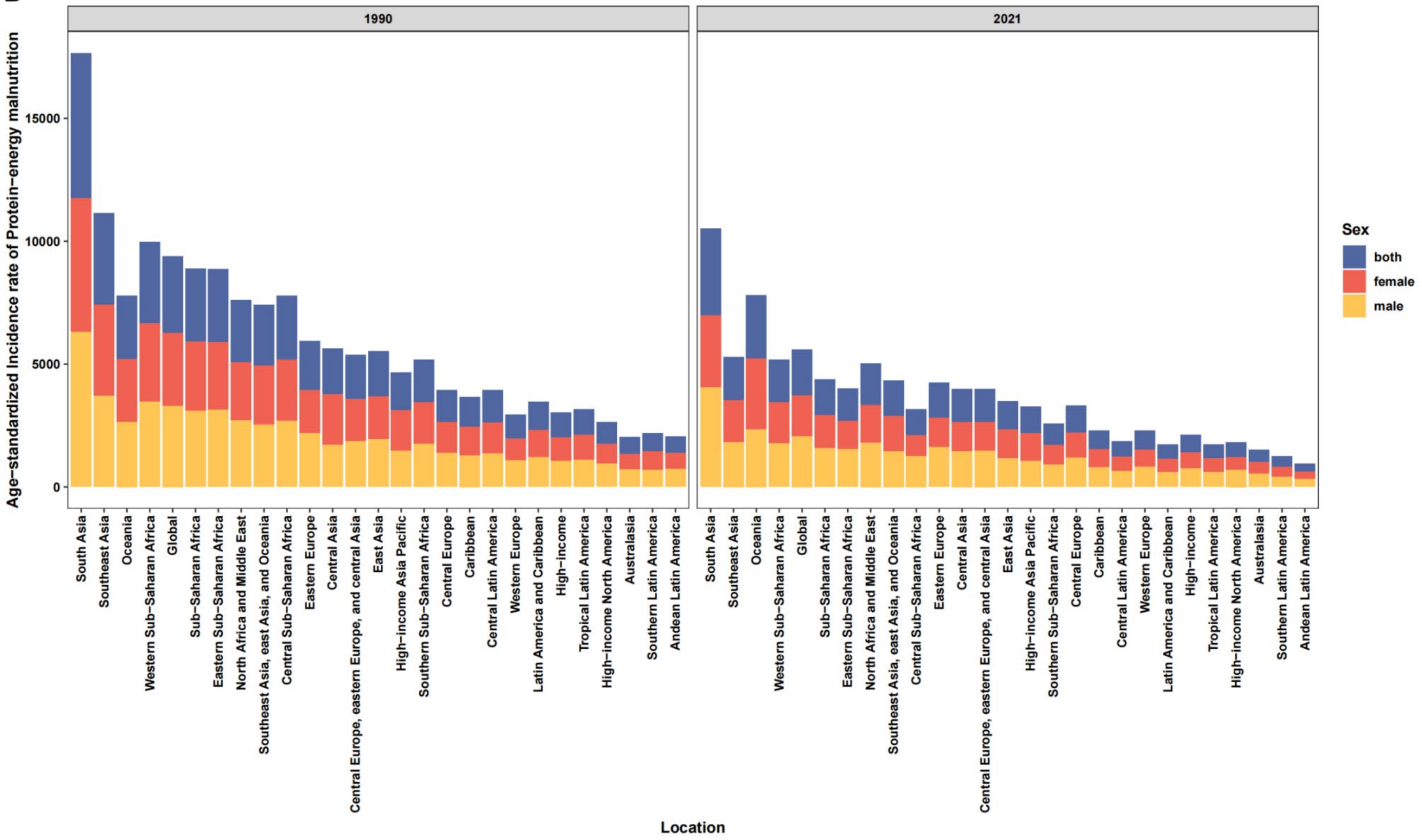

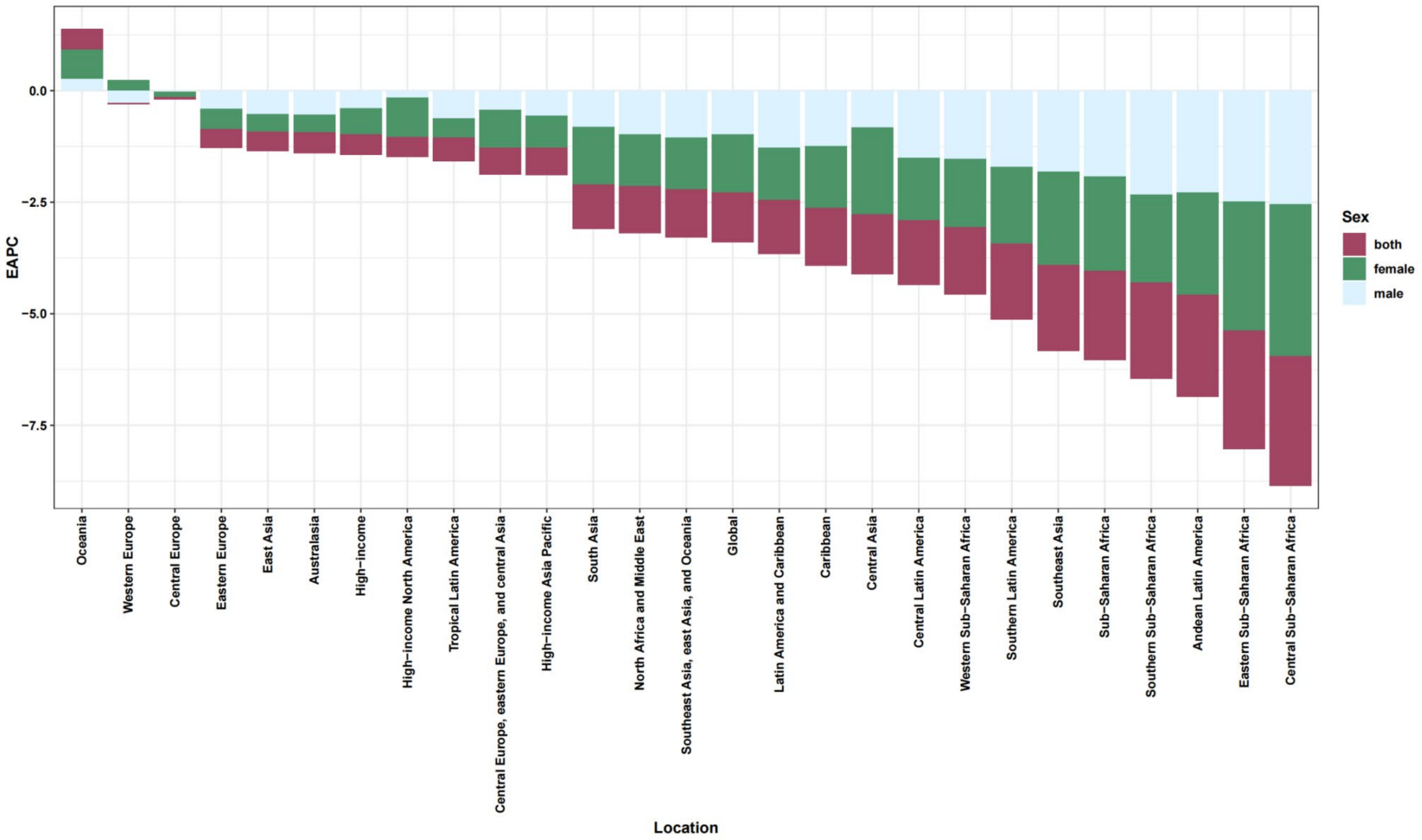

SFigure3 Age-standardized incidence rate of protein-energy malnutrition among children aged 0-14 years old in 21 regions, in 2021 and its trends from1990 to 2021

C

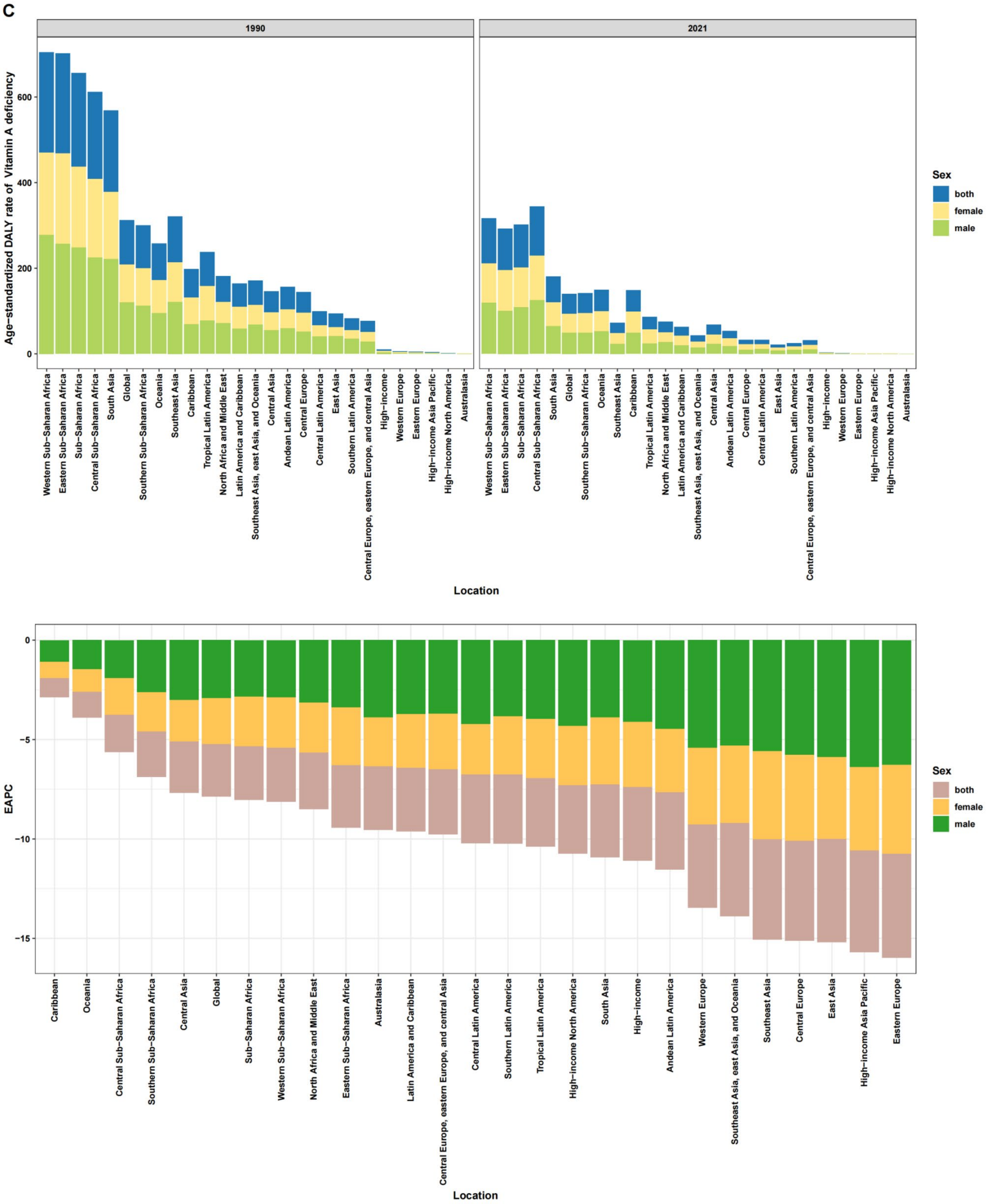

SFigure4 Age-standardized DALY rate of Vitamin A deficiency among children aged 0-14 years old in 21 regions, in 2021 and its trends from1990 to 2021

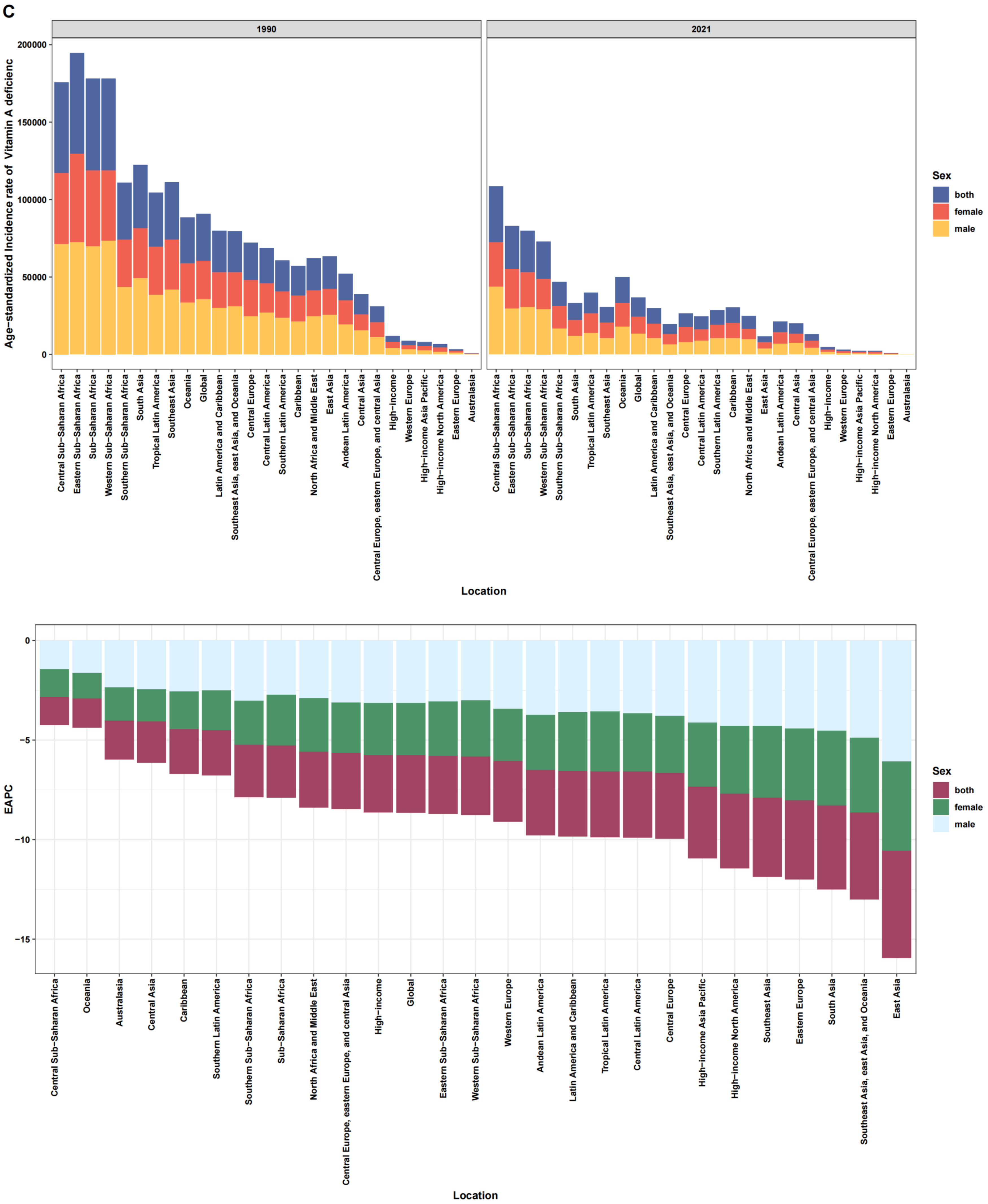

SFigure5 Age-standardized incidence rate of Vitamin A deficiency among children aged 0-14 years old in 21 regions, in 2021 and its trends from1990 to 2021

D

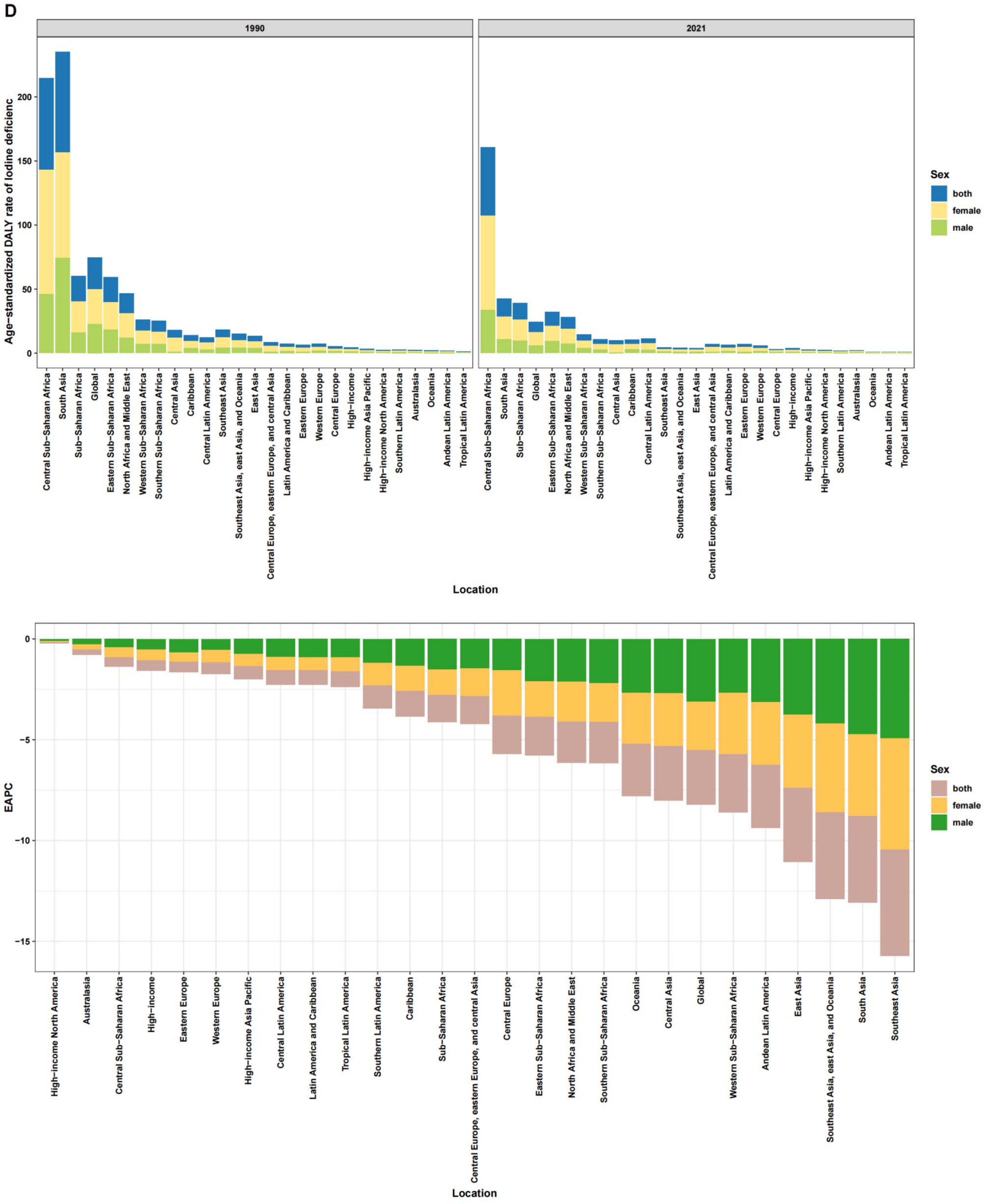

SFigure6 Age-standardized DALY rate of iodine deficiency among children aged 0-14 years old in 21 regions, in 2021 and its trends from1990 to 2021

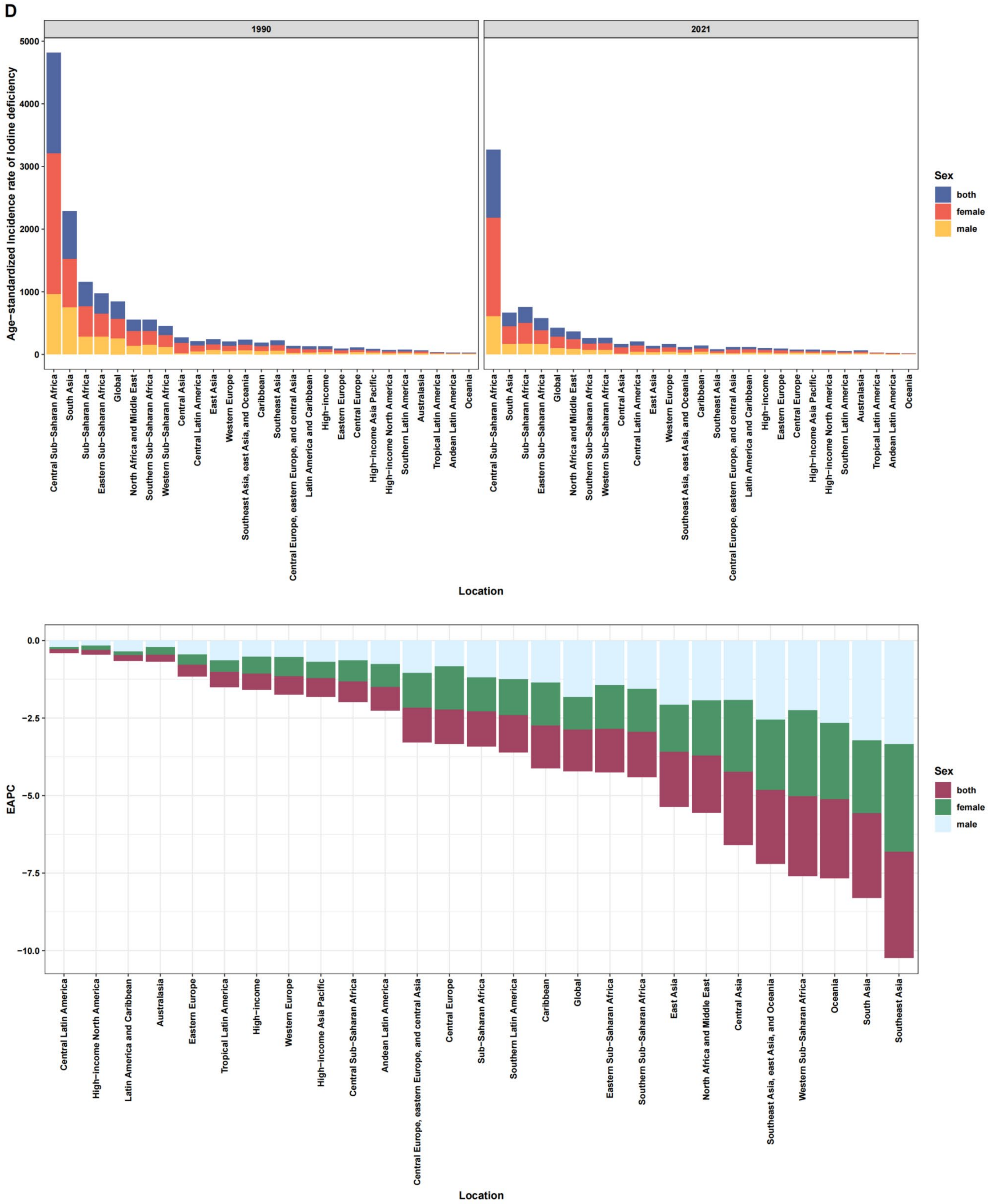

SFigure7 Age-standardized incidence rate of iodine deficiency among children aged 0-14 years old in 21 regions, in 2021 and its trends from1990 to 2021

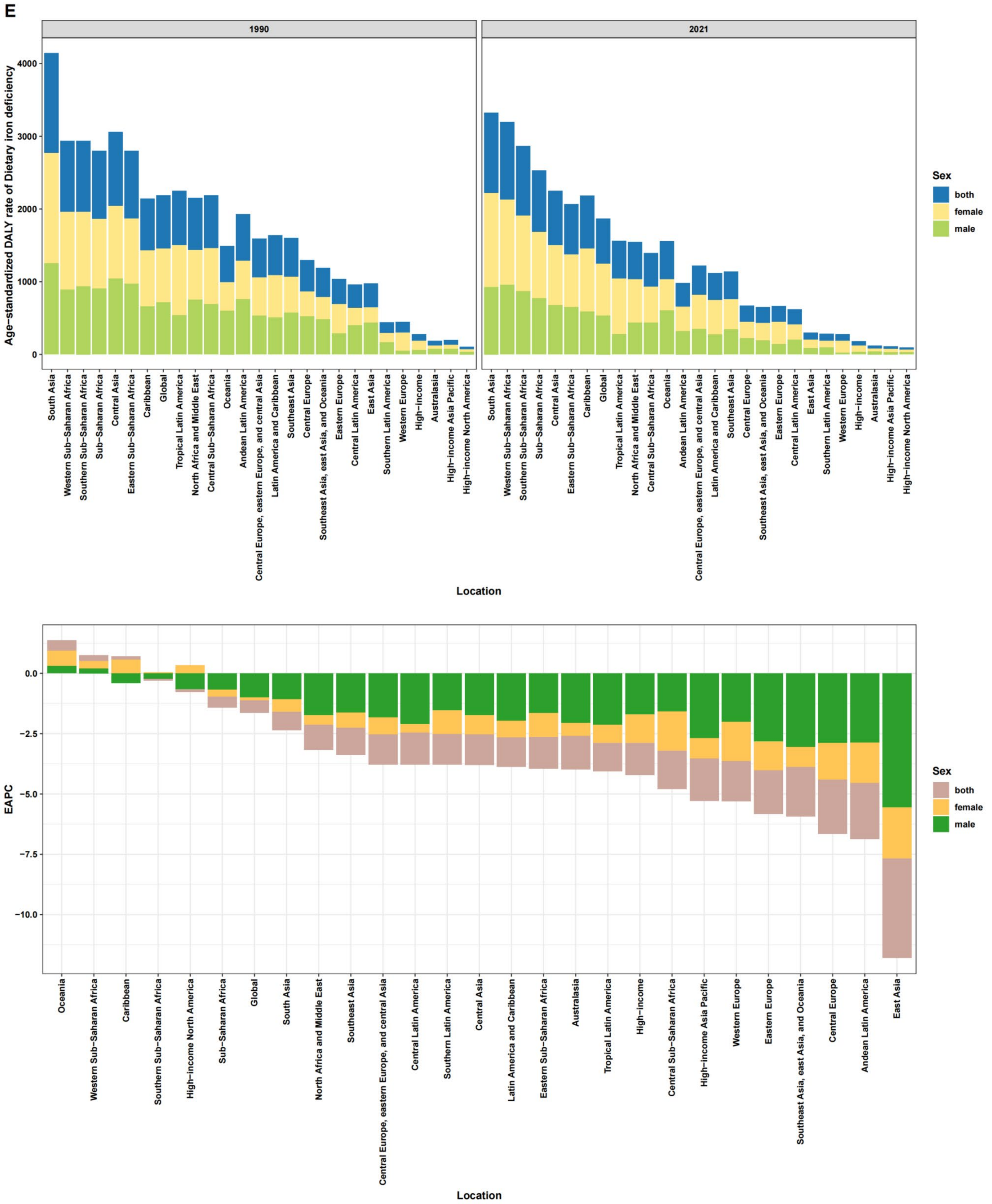

SFigure8 Age-standardized DALY rate of dietary iron deficiency among children aged 0-14 years old in 21 regions, in 2021 and its trends from1990 to 2021

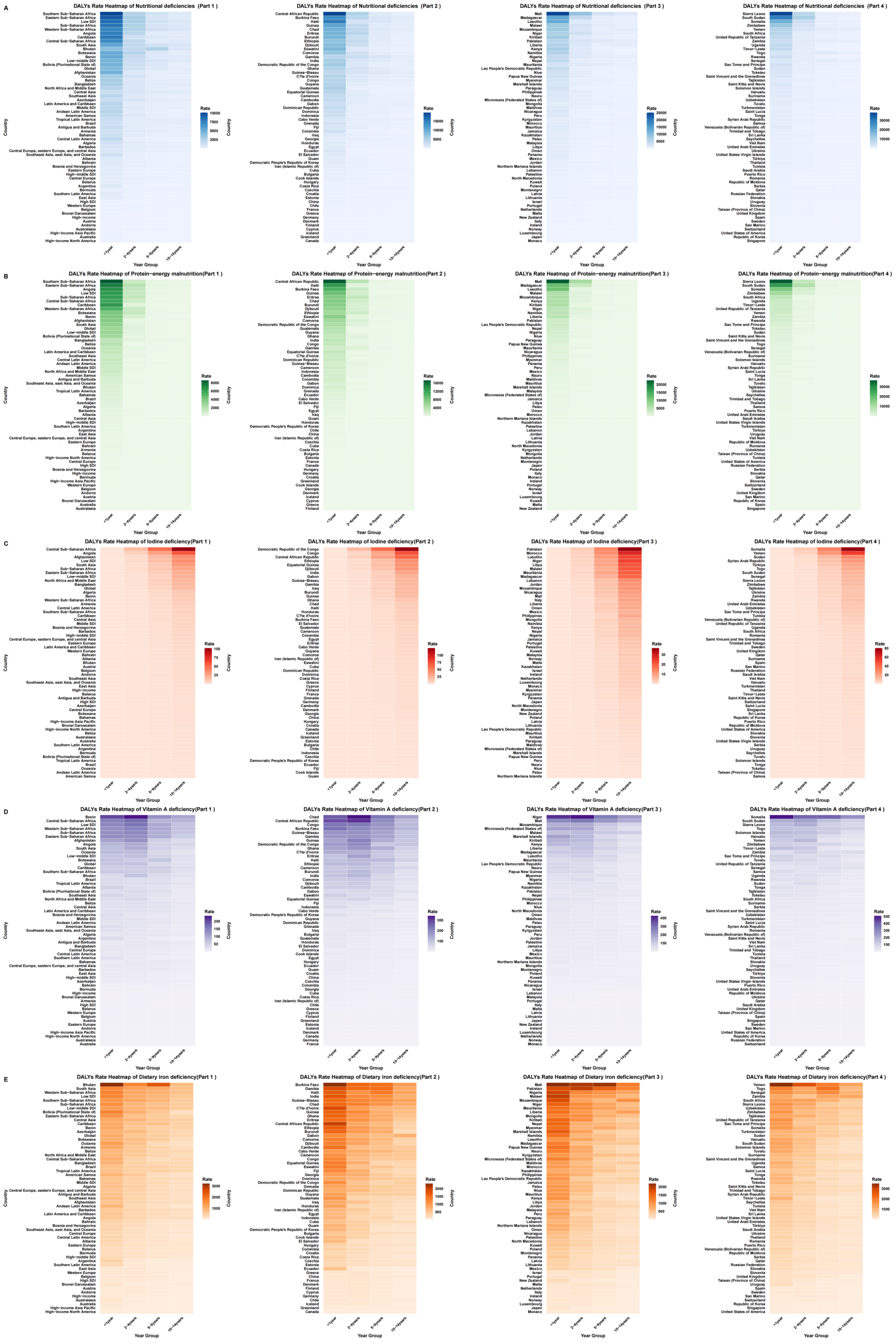

SFigure9. Age special DALY rate of five common nutritinal deficiencies among children aged 0-14 years old in 2021

(A)nutritional deficiencies(B)protein-energy malnutrition(C) iodine deficiency(D)Vitamin A deficiency(E)dietary iron deficiency

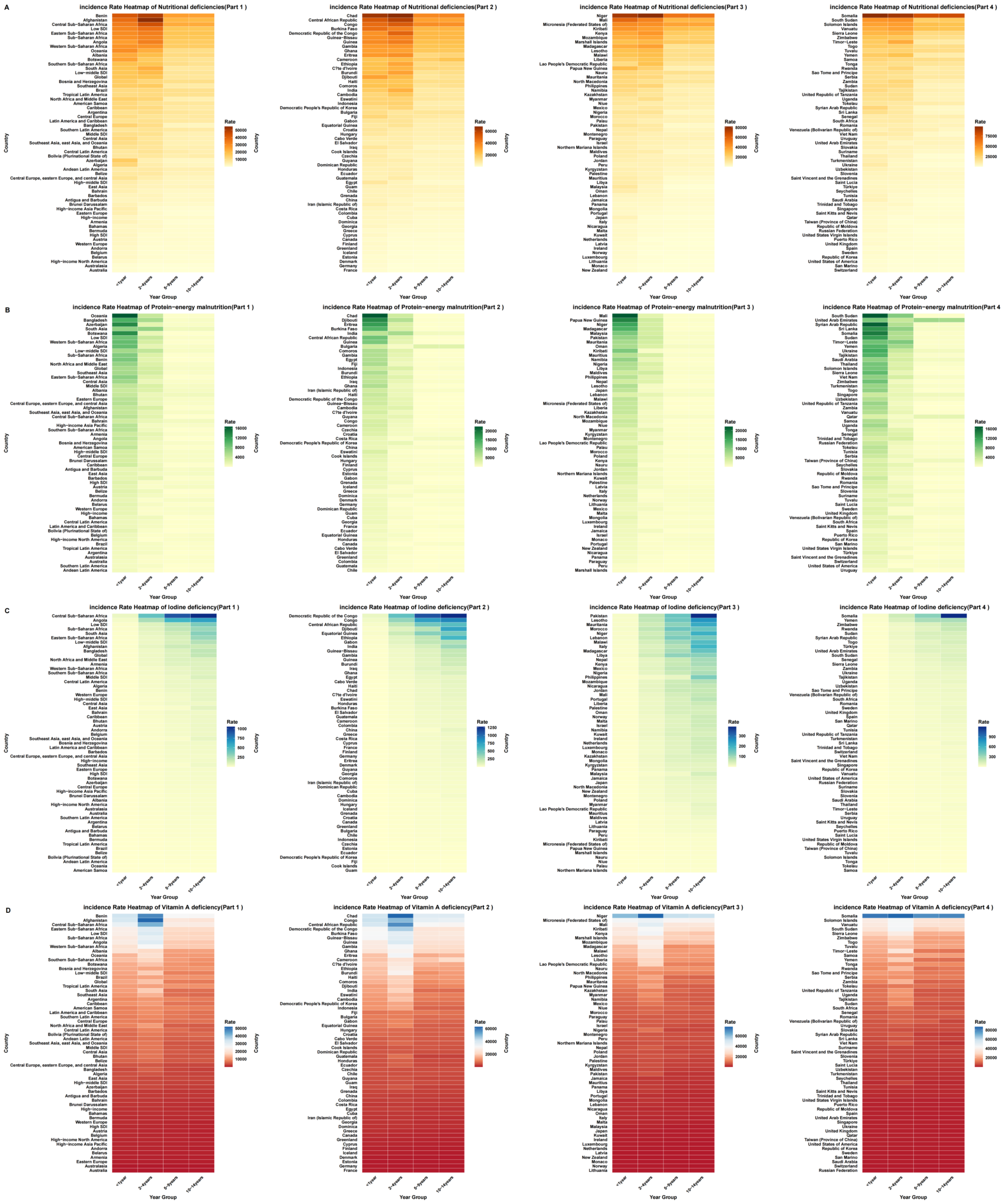

SFigure10 Age special incidence rate of five common nutritinal deficiencies among children aged 0-14 years old in 2021

(A)nutritional deficiencies(B)protein-energy malnutrition (C) iodine deficiency (D)Vitamin A deficiency
