# Supplementary material for: Global, regional and national trends in the burden of nutritional deficiencies in children, 1990–2021
Source: Front Nutr. 2025 Jul 2;12:1565620. doi: 10.3389/fnut.2025.1565620 (PMC12263351; doi:10.3389/fnut.2025.1565620)
Supplement: Supplementary file 2 [file Table_1.DOCX]

**Stable 1. DALYs cases and Age-standardized DALY rate of Nutritional deficiencies in 1990 and 2021 and its trends.**

| location | sex | number | | | Age-standardized rate | | |
| --- | --- | --- | --- | --- | --- | --- | --- |
|  |  | 1990 | 2021 | change rate in case(%) | 1990 | 2021 | EAPC |
| Global | both | 56338352.24(47302244.60,68420786.75) | 22777008.08(17434382.12,29675587.80) | -59.57 | 2896.48(2895.63,2897.32) | 1094.15(1093.66,1094.65) | -3.28(-3.58,-2.98) |
| Central Europe, eastern Europe, and central Asia | both | 726727.39(513512.39,1026046.91) | 361012.36(245752.57,536864.12) | -50.32 | 670.55(668.89,672.21) | 445.19(443.63,446.75) | -1.79(-1.98,-1.60) |
| High-income | both | 277055.69(207384.20,379880.80) | 131021.29(87808.69,192634.99) | -52.71 | 137.77(137.20,138.33) | 75.04(74.60,75.47) | -1.72(-1.88,-1.55) |
| Latin America and Caribbean | both | 3339169.89(2983373.59,3806051.85) | 871227.15(649826.31,1143412.68) | -73.91 | 1975.03(1972.63,1977.43) | 561.35(560.05,562.65) | -4.17(-4.34,-4.01) |
| Southeast Asia, east Asia, and Oceania | both | 6865300.12(5729233.29,8340404.02) | 1463554.55(1052444.83,1972600.51) | -78.68 | 1244.80(1243.77,1245.83) | 333.68(333.09,334.27) | -5.85(-7.01,-4.68) |
| Sub-Saharan Africa | both | 18290654.54(14494246.23,23852025.85) | 10407138.65(8185656.38,13191389.13) | -43.10 | 6423.36(6419.93,6426.78) | 1938.34(1937.02,1939.65) | -3.88(-4.32,-3.43) |
| Andean Latin America | both | 502331.48(413714.23,604000.22) | 107365.99(81361.66,143931.77) | -78.63 | 2473.14(2464.83,2481.48) | 515.77(512.27,519.29) | -5.31(-5.54,-5.09) |
| Australasia | both | 3225.80(1706.36,6676.92) | 2514.01(1253.91,4790.89) | -22.07 | 65.84(63.38,68.38) | 42.60(40.81,44.46) | -1.41(-1.57,-1.25) |
| Caribbean | both | 380110.84(311930.01,458573.51) | 174426.14(128642.46,235282.98) | -54.11 | 2847.35(2837.25,2857.48) | 1423.60(1416.34,1430.89) | -2.03(-2.33,-1.73) |
| Central Asia | both | 349082.58(246675.32,485680.78) | 230946.18(156090.95,336178.39) | -33.84 | 1300.76(1296.11,1305.42) | 809.17(805.65,812.70) | -1.90(-2.04,-1.76) |
| Central Europe | both | 148866.21(98861.11,220032.90) | 43265.79(27873.86,64144.88) | -70.94 | 513.03(510.23,515.85) | 244.19(241.73,246.68) | -2.59(-2.69,-2.49) |
| Central Latin America | both | 1258609.40(1141200.01,1411382.72) | 271124.78(212780.83,351617.75) | -78.46 | 1560.89(1557.74,1564.04) | 376.02(374.40,377.66) | -4.45(-4.58,-4.32) |
| Central Sub-Saharan Africa | both | 2179159.32(1626004.05,3140305.44) | 929607.24(657149.18,1233036.82) | -57.34 | 6778.14(6767.84,6788.44) | 1464.89(1461.64,1468.15) | -5.04(-5.49,-4.59) |
| East Asia | both | 3545256.15(2954921.61,4248166.21) | 340968.49(236727.97,460981.80) | -90.38 | 961.95(960.84,963.06) | 135.26(134.77,135.75) | -9.39(-11.49,-7.24) |
| Eastern Europe | both | 228778.60(159033.25,332062.28) | 86800.39(56215.52,130711.88) | -62.06 | 438.06(436.13,439.99) | 247.82(246.04,249.62) | -2.26(-2.50,-2.02) |
| Eastern Sub-Saharan Africa | both | 9984038.32(7864454.63,13031705.43) | 4078886.95(3262357.63,5143268.05) | -59.15 | 8784.27(8777.97,8790.57) | 2032.43(2030.21,2034.64) | -4.68(-5.45,-3.90) |
| High-income Asia Pacific | both | 26720.95(16096.64,44154.66) | 9020.80(5309.50,14926.52) | -66.24 | 76.57(75.58,77.58) | 41.25(40.33,42.18) | -1.85(-2.09,-1.62) |
| High-income North America | both | 29912.52(18706.40,48249.95) | 34700.49(23861.49,50263.33) | 16.01 | 45.31(44.76,45.86) | 51.30(50.73,51.87) | 0.90(0.59,1.21) |
| North Africa and Middle East | both | 2387237.59(1858016.61,3114362.33) | 1315398.79(953321.05,1836095.73) | -44.90 | 1541.53(1539.39,1543.67) | 698.95(697.66,700.25) | -2.63(-2.73,-2.52) |
| Oceania | both | 32532.63(24640.28,41995.99) | 49951.69(34078.92,74615.81) | 53.54 | 918.31(906.45,930.29) | 799.49(791.41,807.64) | -0.14(-0.31,0.02) |
| South Asia | both | 24452207.02(20030246.90,29864683.93) | 8227655.30(5987212.54,11400253.39) | -66.35 | 5113.94(5111.72,5116.16) | 1628.72(1627.51,1629.92) | -3.54(-3.59,-3.49) |
| Southeast Asia | both | 3287511.33(2664759.66,4127654.70) | 1072634.37(777483.83,1457774.06) | -67.37 | 1805.58(1803.42,1807.74) | 603.36(602.11,604.61) | -3.54(-3.66,-3.43) |
| Southern Latin America | both | 109438.00(91888.67,140412.81) | 19452.51(11977.88,33494.08) | -82.23 | 581.86(577.86,585.89) | 129.45(127.40,131.53) | -4.77(-5.05,-4.48) |
| Southern Sub-Saharan Africa | both | 779109.17(655892.60,937837.07) | 580835.98(465787.20,711664.27) | -25.45 | 2867.52(2859.80,2875.25) | 2079.16(2072.99,2085.35) | -0.62(-0.81,-0.44) |
| Tropical Latin America | both | 1198118.17(1028345.59,1424112.18) | 318310.23(212476.51,453683.70) | -73.43 | 2176.84(2172.59,2181.10) | 613.15(610.89,615.43) | -4.39(-4.59,-4.19) |
| Western Europe | both | 107758.41(70270.54,158034.75) | 65333.48(42901.39,96792.86) | -39.37 | 157.68(156.67,158.69) | 100.93(100.11,101.76) | -1.49(-1.64,-1.34) |
| Western Sub-Saharan Africa | both | 5348347.73(4233796.14,7068840.17) | 4817808.48(3676427.54,6261451.05) | -9.92 | 4687.23(4682.55,4691.92) | 1982.19(1980.23,1984.16) | -2.80(-2.90,-2.69) |
| Afghanistan | both | 130448.32(95965.02,177245.25) | 136131.98(99509.68,183148.87) | 4.36 | 2420.83(2406.28,2435.45) | 851.54(846.67,856.42) | -3.59(-3.97,-3.21) |
| Albania | both | 11311.71(7672.96,16267.91) | 1360.71(893.11,1998.01) | -87.97 | 960.25(941.15,979.66) | 289.17(272.51,306.64) | -4.46(-4.69,-4.23) |
| Algeria | both | 121729.13(86443.69,167834.70) | 63763.80(39543.00,95050.43) | -47.62 | 1108.44(1101.78,1115.12) | 461.74(457.90,465.61) | -3.12(-3.24,-3.00) |
| American Samoa | both | 205.86(156.34,270.64) | 82.76(59.77,113.78) | -59.80 | 721.95(604.22,857.09) | 550.81(418.26,715.08) | -0.85(-0.94,-0.77) |
| Andorra | both | 9.87(5.63,15.74) | 7.69(3.92,12.49) | -22.12 | 113.85(50.46,227.29) | 83.55(32.19,186.13) | -1.01(-1.23,-0.79) |
| Angola | both | 829456.21(598273.06,1232058.85) | 249466.76(177719.01,334912.84) | -69.92 | 14139.43(14104.33,14174.59) | 1477.55(1471.17,1483.94) | -7.52(-7.80,-7.24) |
| Antigua and Barbuda | both | 171.12(121.54,250.31) | 96.28(66.95,150.72) | -43.73 | 835.24(699.86,990.77) | 514.36(404.06,647.76) | -1.60(-1.82,-1.37) |
| Argentina | both | 88386.19(73000.57,116684.40) | 14721.86(8267.64,28218.25) | -83.34 | 698.95(693.57,704.36) | 140.89(138.32,143.50) | -5.09(-5.46,-4.72) |
| Armenia | both | 12048.98(8824.11,16739.83) | 3366.73(2111.06,5246.62) | -72.06 | 1098.46(1077.66,1119.58) | 570.38(549.82,591.55) | -2.35(-2.47,-2.24) |
| Australia | both | 2365.67(1177.39,5506.14) | 1840.27(906.74,3896.96) | -22.21 | 60.04(57.44,62.72) | 38.00(36.14,39.93) | -1.51(-1.69,-1.32) |
| Austria | both | 1858.69(1049.49,3082.74) | 1046.85(555.32,1802.92) | -43.68 | 142.30(135.51,149.35) | 83.77(78.52,89.29) | -1.81(-2.03,-1.59) |
| Azerbaijan | both | 37829.84(27742.73,50435.90) | 16742.95(10711.16,24869.58) | -55.74 | 1460.78(1445.06,1476.63) | 729.94(718.15,741.89) | -2.67(-2.84,-2.49) |
| Bahamas | both | 707.58(511.16,987.82) | 405.73(266.66,610.94) | -42.66 | 832.84(766.75,903.55) | 510.55(456.59,569.77) | -1.56(-1.83,-1.29) |
| Bahrain | both | 1164.72(781.36,1649.52) | 819.84(514.30,1252.29) | -29.61 | 666.74(626.01,709.54) | 275.94(255.83,297.30) | -2.94(-3.10,-2.79) |
| Bangladesh | both | 5334383.47(4397207.50,6278658.29) | 381849.13(271266.28,537500.67) | -92.84 | 9476.15(9467.35,9484.96) | 813.10(810.28,815.93) | -8.09(-8.34,-7.84) |
| Barbados | both | 488.97(352.03,690.29) | 210.16(137.34,317.81) | -57.02 | 755.68(684.01,833.42) | 448.91(385.15,521.14) | -1.64(-1.83,-1.46) |
| Belarus | both | 9787.78(6722.08,13926.88) | 3377.95(2198.90,5186.00) | -65.49 | 401.06(392.60,409.66) | 215.73(207.98,223.71) | -2.43(-2.63,-2.23) |
| Belgium | both | 2600.67(1524.93,3979.44) | 1699.47(920.22,2726.84) | -34.65 | 149.48(143.43,155.73) | 93.79(89.13,98.65) | -1.63(-1.79,-1.48) |
| Belize | both | 2059.12(1765.31,2440.24) | 1042.82(748.59,1485.68) | -49.36 | 2128.66(2026.83,2234.60) | 784.89(732.39,840.43) | -3.10(-3.53,-2.68) |
| Benin | both | 166363.25(112224.87,247163.95) | 92805.44(63840.75,129447.71) | -44.22 | 5433.85(5403.79,5464.06) | 1334.47(1324.98,1344.02) | -4.55(-4.74,-4.35) |
| Bermuda | both | 51.57(33.60,76.77) | 17.60(10.78,28.35) | -65.87 | 394.28(284.80,534.16) | 207.12(115.79,348.35) | -2.07(-2.32,-1.83) |
| Bhutan | both | 8006.24(4823.32,12134.93) | 3682.30(2177.79,5588.03) | -54.01 | 2744.85(2679.21,2811.79) | 1938.79(1872.51,2006.99) | -1.60(-1.76,-1.44) |
| Bolivia (Plurinational State of) | both | 188456.09(135017.41,244603.11) | 45552.75(33401.08,62498.01) | -75.83 | 4758.61(4732.08,4785.27) | 1131.74(1119.88,1143.69) | -4.74(-4.91,-4.57) |
| Bosnia and Herzegovina | both | 5828.19(3480.86,8866.19) | 1370.32(822.19,2138.86) | -76.49 | 537.64(522.99,552.61) | 282.64(266.82,299.22) | -2.58(-2.85,-2.31) |
| Botswana | both | 15242.86(11349.01,19975.23) | 12652.33(8900.12,17214.76) | -17.00 | 2033.05(1994.46,2072.23) | 1436.04(1405.65,1466.95) | -0.94(-1.09,-0.78) |
| Brazil | both | 1179803.01(1012002.96,1401698.01) | 307416.49(204371.75,440015.63) | -73.94 | 2224.93(2220.55,2229.31) | 616.26(613.94,618.59) | -4.45(-4.66,-4.25) |
| Brunei Darussalam | both | 115.25(65.08,195.19) | 70.15(38.99,125.36) | -39.13 | 108.93(87.75,133.87) | 70.46(53.63,91.29) | -1.29(-1.55,-1.02) |
| Bulgaria | both | 8173.22(5103.64,12913.87) | 3055.85(1845.53,4639.05) | -62.61 | 473.39(462.35,484.66) | 312.60(300.72,324.85) | -1.49(-1.64,-1.35) |
| Burkina Faso | both | 429942.36(304723.99,581589.05) | 396068.70(292255.06,532617.36) | -7.88 | 6832.54(6808.14,6857.00) | 3149.14(3138.07,3160.24) | -2.42(-2.71,-2.13) |
| Burundi | both | 397473.98(229564.91,679272.53) | 131797.80(80843.27,196227.70) | -66.84 | 12215.70(12172.05,12259.48) | 2013.29(2001.29,2025.35) | -5.86(-6.07,-5.65) |
| Cabo Verde | both | 6468.94(4838.36,8507.06) | 1187.23(784.63,1882.40) | -81.65 | 3343.09(3252.47,3435.74) | 811.62(762.28,863.56) | -4.87(-5.31,-4.42) |
| Cambodia | both | 301751.68(201811.32,455716.39) | 50041.58(33568.55,70744.19) | -83.42 | 5376.05(5354.87,5397.30) | 915.98(907.26,924.76) | -6.49(-6.86,-6.11) |
| Cameroon | both | 129988.74(94600.33,172693.33) | 150310.73(102840.84,213762.29) | 15.63 | 2075.75(2062.63,2088.95) | 999.76(994.18,1005.37) | -2.58(-2.75,-2.41) |
| Canada | both | 1946.33(1164.25,3183.33) | 2680.40(1406.35,4245.60) | 37.72 | 33.17(31.62,34.79) | 42.17(40.52,43.87) | 1.43(1.07,1.79) |
| Central African Republic | both | 76889.80(51391.59,115019.73) | 91198.31(64696.18,124970.73) | 18.61 | 4968.44(4929.16,5007.98) | 3497.10(3471.82,3522.53) | -0.92(-1.27,-0.56) |
| Chad | both | 284704.78(199455.73,407422.68) | 274978.94(199061.87,367421.56) | -3.42 | 7614.02(7581.65,7646.50) | 2580.49(2569.71,2591.31) | -3.60(-3.74,-3.47) |
| Chile | both | 16790.98(14042.55,20248.54) | 3911.73(2895.69,5707.02) | -76.70 | 335.96(330.17,341.84) | 100.80(97.32,104.39) | -3.84(-4.09,-3.58) |
| China | both | 3457833.63(2887157.76,4145940.91) | 322359.29(224477.27,435032.32) | -90.68 | 972.01(970.88,973.15) | 131.94(131.45,132.43) | -7.21(-7.62,-6.80) |
| Colombia | both | 147850.43(125296.03,176783.78) | 50258.30(38204.33,67007.74) | -66.01 | 1028.41(1022.46,1034.39) | 382.76(378.79,386.76) | -2.78(-3.15,-2.41) |
| Comoros | both | 15412.11(10702.79,20453.20) | 4079.86(2929.52,5476.32) | -73.53 | 5973.65(5867.79,6081.03) | 1643.04(1588.28,1699.30) | -4.31(-4.47,-4.16) |
| Congo | both | 28865.69(21807.77,37685.90) | 23827.45(16844.49,33403.38) | -17.45 | 2482.76(2451.59,2514.24) | 1206.54(1190.02,1223.25) | -2.69(-3.12,-2.25) |
| Cook Islands | both | 32.87(20.97,50.57) | 9.81(6.12,15.13) | -70.16 | 475.96(314.42,695.29) | 265.41(115.16,535.44) | -1.85(-1.96,-1.74) |
| Costa Rica | both | 3770.62(2632.31,5127.01) | 2121.00(1278.68,3185.56) | -43.75 | 309.78(299.12,320.74) | 206.55(197.16,216.30) | -0.90(-1.08,-0.73) |
| Croatia | both | 3360.16(2085.84,5136.97) | 1151.77(718.21,1801.99) | -65.72 | 346.06(333.60,358.90) | 194.19(182.31,206.70) | -2.11(-2.19,-2.02) |
| Cuba | both | 12209.69(7947.74,18783.17) | 6220.21(3785.60,9435.40) | -49.06 | 463.31(454.47,472.28) | 348.81(339.52,358.30) | -0.86(-0.98,-0.74) |
| Cyprus | both | 302.08(163.27,500.21) | 166.26(92.64,284.54) | -44.96 | 158.39(139.92,178.79) | 78.92(66.79,92.72) | -2.65(-2.89,-2.41) |
| Czechia | both | 8125.01(4962.35,12584.29) | 3440.10(2079.44,5277.79) | -57.66 | 378.44(369.57,387.50) | 197.14(190.14,204.35) | -2.03(-2.21,-1.84) |
| C?te d'Ivoire | both | 201665.05(150377.96,270670.43) | 167540.02(113878.04,229963.93) | -16.92 | 2779.74(2765.87,2793.65) | 1290.50(1283.72,1297.31) | -2.53(-2.79,-2.27) |
| Democratic People's Republic of Korea | both | 73070.67(52570.62,101653.18) | 15159.09(10108.65,22967.18) | -79.25 | 1002.61(994.43,1010.85) | 312.67(307.18,318.24) | -12.18(-18.14,-5.80) |
| Democratic Republic of the Congo | both | 1225014.83(832718.28,1788670.44) | 552916.83(366008.88,781017.59) | -54.86 | 5311.94(5301.21,5322.70) | 1356.70(1352.81,1360.60) | -4.43(-5.03,-3.83) |
| Denmark | both | 1240.87(699.40,2088.02) | 756.39(406.72,1237.35) | -39.04 | 148.18(139.48,157.30) | 83.86(77.66,90.46) | -2.14(-2.32,-1.97) |
| Djibouti | both | 11220.51(8061.54,14972.49) | 8153.96(5730.33,11311.14) | -27.33 | 5492.03(5376.57,5609.46) | 1833.73(1789.94,1878.35) | -3.71(-4.12,-3.29) |
| Dominica | both | 247.72(175.25,336.45) | 100.15(68.46,139.39) | -59.57 | 919.51(798.19,1055.25) | 762.86(604.37,954.18) | -0.49(-0.74,-0.24) |
| Dominican Republic | both | 107794.64(89518.12,127062.35) | 26578.85(19062.52,36449.88) | -75.34 | 3211.24(3189.44,3233.17) | 828.46(817.60,839.44) | -3.90(-4.40,-3.40) |
| Ecuador | both | 64426.93(57698.74,72801.10) | 12615.41(9659.49,16635.59) | -80.42 | 1218.60(1207.23,1230.05) | 228.80(224.33,233.34) | -5.80(-6.58,-5.00) |
| Egypt | both | 242207.84(176620.92,323787.75) | 155687.40(103440.17,223561.69) | -35.72 | 967.93(963.72,972.16) | 394.04(391.92,396.17) | -2.48(-2.64,-2.32) |
| El Salvador | both | 31018.70(25142.52,38088.05) | 5768.26(4141.91,8275.99) | -81.40 | 1152.10(1137.50,1166.85) | 292.50(284.25,300.93) | -4.52(-4.80,-4.24) |
| Equatorial Guinea | both | 9563.97(7002.53,12766.67) | 5351.55(3442.04,8183.68) | -44.04 | 3894.47(3806.49,3984.06) | 928.15(901.54,955.39) | -5.88(-6.38,-5.37) |
| Eritrea | both | 288924.30(198733.68,388822.51) | 72674.33(53395.83,101122.44) | -74.85 | 15131.50(15067.55,15195.66) | 2512.77(2491.99,2533.69) | -5.63(-5.98,-5.28) |
| Estonia | both | 1473.96(1027.84,2136.18) | 401.04(250.78,616.94) | -72.79 | 409.44(387.27,432.60) | 183.55(164.83,203.96) | -2.99(-3.21,-2.77) |
| Eswatini | both | 11215.29(8579.10,14645.59) | 6535.08(4786.60,8807.42) | -41.73 | 2159.20(2111.18,2208.07) | 1372.54(1335.07,1410.85) | -1.16(-1.45,-0.87) |
| Ethiopia | both | 3010351.03(2267014.50,4049846.13) | 987163.27(755184.47,1248828.14) | -67.21 | 10342.62(10329.29,10355.96) | 2030.98(2026.50,2035.46) | -5.93(-6.29,-5.57) |
| Fiji | both | 2617.42(1813.49,3591.81) | 1991.01(1357.58,2770.93) | -23.93 | 835.60(799.65,872.83) | 627.21(595.60,660.15) | -0.89(-0.94,-0.83) |
| Finland | both | 1361.42(762.46,2201.95) | 635.41(361.38,982.39) | -53.33 | 146.80(138.61,155.37) | 81.42(74.80,88.53) | -2.14(-2.36,-1.93) |
| France | both | 19870.61(12571.94,32022.33) | 10696.56(6456.01,17446.87) | -46.17 | 173.44(170.89,176.02) | 97.30(95.35,99.29) | -2.05(-2.24,-1.85) |
| Gabon | both | 9368.81(6682.18,12448.82) | 6846.36(4189.03,10695.03) | -26.92 | 2036.58(1991.60,2082.37) | 1029.84(1003.70,1056.53) | -2.32(-2.73,-1.92) |
| Gambia | both | 30000.33(23180.23,38397.45) | 21923.68(15642.34,29796.94) | -26.92 | 5576.29(5504.58,5648.72) | 2055.52(2025.72,2085.66) | -3.70(-3.95,-3.45) |
| Georgia | both | 10253.78(6554.18,15753.17) | 4032.75(2456.94,6237.82) | -60.67 | 749.53(734.18,765.12) | 545.86(527.98,564.23) | -0.97(-1.05,-0.89) |
| Germany | both | 18079.72(10068.58,28287.35) | 10289.90(5699.69,16130.92) | -43.09 | 141.93(139.75,144.15) | 88.56(86.78,90.37) | -1.39(-1.53,-1.25) |
| Ghana | both | 397573.18(283266.68,551976.07) | 218296.42(147321.09,310995.48) | -45.09 | 4815.74(4798.35,4833.18) | 1558.16(1550.97,1565.39) | -3.51(-3.67,-3.36) |
| Greece | both | 2518.24(1411.72,3968.85) | 1309.06(691.33,2267.10) | -48.02 | 137.40(131.66,143.33) | 102.00(96.20,108.07) | -1.10(-1.30,-0.89) |
| Greenland | both | 12.75(7.26,20.71) | 5.53(3.00,9.21) | -56.64 | 85.27(42.97,153.54) | 46.72(15.21,112.74) | -2.06(-2.23,-1.89) |
| Grenada | both | 411.80(311.46,555.53) | 152.99(105.49,224.23) | -62.85 | 1073.24(960.37,1196.38) | 660.89(550.24,788.95) | -1.41(-1.70,-1.12) |
| Guam | both | 237.63(173.99,334.49) | 142.58(95.91,204.81) | -40.00 | 432.79(370.11,503.62) | 327.91(268.34,397.45) | -0.83(-1.04,-0.63) |
| Guatemala | both | 183631.78(162645.68,205527.25) | 44292.18(35215.21,57309.60) | -75.88 | 3402.20(3383.92,3420.55) | 758.12(749.87,766.45) | -4.36(-4.67,-4.04) |
| Guinea | both | 270522.36(189200.49,372166.23) | 168943.41(119553.65,228552.43) | -37.55 | 7248.89(7216.54,7281.36) | 2397.60(2384.72,2410.53) | -3.46(-3.70,-3.22) |
| Guinea-Bissau | both | 36480.24(24823.95,53443.57) | 15250.66(10065.16,21072.36) | -58.19 | 6452.16(6376.43,6528.59) | 1621.20(1593.78,1649.00) | -4.60(-4.79,-4.41) |
| Guyana | both | 11446.56(9352.37,13794.79) | 2201.80(1711.98,3033.01) | -80.76 | 3355.20(3289.46,3422.02) | 982.13(938.48,1027.38) | -3.13(-3.48,-2.77) |
| Haiti | both | 205671.44(155159.33,268267.01) | 124116.63(87442.66,171318.26) | -39.65 | 6044.48(6015.29,6073.78) | 2541.05(2525.68,2556.49) | -2.70(-2.93,-2.47) |
| Honduras | both | 28998.06(22633.69,37026.68) | 13913.51(9229.56,19531.07) | -52.02 | 1119.85(1105.50,1134.36) | 398.28(391.08,405.59) | -3.35(-3.58,-3.12) |
| Hungary | both | 9110.71(5538.71,14100.16) | 3142.74(1938.96,4896.76) | -65.50 | 441.04(431.25,451.02) | 223.47(215.16,232.03) | -2.15(-2.31,-1.99) |
| Iceland | both | 71.87(41.04,113.14) | 46.30(25.79,80.36) | -35.58 | 117.13(89.92,150.55) | 72.01(51.68,98.23) | -1.75(-1.93,-1.57) |
| India | both | 16635281.93(13341569.16,20995106.90) | 5903144.78(4228492.52,8249347.21) | -64.51 | 4684.24(4681.78,4686.70) | 1640.79(1639.36,1642.22) | -3.21(-3.33,-3.08) |
| Indonesia | both | 1415613.92(1133366.73,1787201.54) | 423685.82(314900.72,561370.14) | -70.07 | 1978.65(1975.02,1982.29) | 609.90(607.89,611.91) | -3.68(-3.78,-3.58) |
| Iran (Islamic Republic of) | both | 272299.25(208121.18,365370.18) | 73578.69(46317.96,116145.68) | -72.98 | 1058.52(1054.24,1062.81) | 370.84(367.95,373.74) | -3.12(-3.25,-2.99) |
| Iraq | both | 105310.52(77349.88,140292.60) | 65724.22(43250.47,97816.75) | -37.59 | 1177.63(1169.99,1185.31) | 491.12(487.08,495.18) | -3.10(-3.29,-2.91) |
| Ireland | both | 1410.53(794.00,2176.17) | 741.99(417.25,1170.39) | -47.40 | 153.59(145.15,162.41) | 78.79(72.91,85.06) | -2.40(-2.60,-2.19) |
| Israel | both | 2828.17(1704.58,4636.26) | 2827.48(1465.20,4377.37) | -0.02 | 189.71(182.37,197.28) | 110.07(105.85,114.43) | -1.90(-2.05,-1.76) |
| Italy | both | 10600.99(6078.71,17588.21) | 5842.23(3132.97,9174.95) | -44.89 | 125.03(122.49,127.61) | 82.37(80.13,84.66) | -1.30(-1.43,-1.18) |
| Jamaica | both | 12455.82(10122.60,15492.89) | 3106.39(2016.24,4780.10) | -75.06 | 1226.09(1201.02,1251.58) | 528.45(508.25,549.30) | -2.34(-2.91,-1.77) |
| Japan | both | 14064.05(7763.86,24574.03) | 6655.38(3786.37,11129.35) | -52.68 | 62.78(61.66,63.92) | 43.94(42.81,45.10) | -1.07(-1.17,-0.96) |
| Jordan | both | 13390.38(8764.11,19775.70) | 15231.91(9389.95,23120.73) | 13.75 | 777.61(763.55,791.88) | 412.22(405.16,419.39) | -2.30(-2.45,-2.16) |
| Kazakhstan | both | 65266.67(42689.49,95143.79) | 32280.48(19602.20,58491.96) | -50.54 | 1164.48(1154.74,1174.27) | 568.47(561.82,575.19) | -2.97(-3.25,-2.69) |
| Kenya | both | 531168.56(410408.25,674581.26) | 259912.22(210157.96,324513.37) | -51.07 | 3587.57(3576.20,3598.98) | 1267.30(1261.68,1272.94) | -3.08(-3.26,-2.90) |
| Kiribati | both | 2000.57(1490.16,2587.27) | 969.58(706.11,1280.89) | -51.53 | 3257.89(3056.36,3470.04) | 1517.58(1395.08,1648.56) | -2.49(-2.55,-2.44) |
| Kuwait | both | 2493.90(1584.65,3704.37) | 2299.58(1413.79,3526.27) | -7.79 | 433.19(415.19,451.79) | 274.04(262.13,286.39) | -1.53(-1.73,-1.33) |
| Kyrgyzstan | both | 17857.55(12249.47,25560.66) | 14751.75(9222.68,22753.70) | -17.39 | 1015.30(999.45,1031.35) | 619.03(608.33,629.88) | -1.83(-1.98,-1.68) |
| Lao People's Democratic Republic | both | 103585.41(73009.79,166550.83) | 19879.52(14326.45,27897.30) | -80.81 | 4865.84(4833.80,4898.06) | 811.08(799.09,823.22) | -6.03(-6.21,-5.84) |
| Latvia | both | 2453.60(1635.14,3502.73) | 649.35(410.40,1016.55) | -73.53 | 416.98(399.52,435.02) | 216.97(199.47,235.68) | -2.41(-2.53,-2.29) |
| Lebanon | both | 7891.59(5129.55,11606.08) | 4596.07(2808.24,7153.67) | -41.76 | 724.76(707.69,742.15) | 362.53(351.33,374.01) | -2.28(-2.38,-2.19) |
| Lesotho | both | 21343.30(16522.15,27325.53) | 13129.81(9410.19,17924.40) | -38.48 | 2584.30(2544.71,2624.38) | 1898.15(1861.85,1935.01) | -0.73(-0.95,-0.51) |
| Liberia | both | 142822.01(103016.36,201527.21) | 40008.03(27978.44,53791.16) | -71.99 | 8507.60(8453.36,8562.12) | 1639.02(1621.19,1657.00) | -6.06(-6.58,-5.53) |
| Libya | both | 11979.62(7977.09,17505.68) | 6592.59(4165.99,9995.83) | -44.97 | 640.02(627.74,652.50) | 464.33(452.15,476.77) | -0.90(-1.14,-0.66) |
| Lithuania | both | 3253.10(2227.14,4899.18) | 877.67(573.34,1336.02) | -73.02 | 379.09(365.28,393.32) | 213.17(198.38,228.83) | -2.00(-2.08,-1.92) |
| Luxembourg | both | 92.67(54.19,145.66) | 67.34(39.95,112.65) | -27.33 | 142.07(112.94,176.87) | 69.23(52.83,89.43) | -2.50(-2.75,-2.25) |
| Madagascar | both | 903024.81(731618.86,1063387.80) | 361408.74(261028.74,496532.78) | -59.98 | 12834.84(12803.44,12866.31) | 2858.53(2848.15,2868.93) | -4.35(-4.56,-4.13) |
| Malawi | both | 556900.06(396846.99,776454.98) | 182621.90(127542.59,251986.19) | -67.21 | 8804.23(8776.67,8831.86) | 2051.41(2040.84,2062.02) | -5.11(-5.43,-4.79) |
| Malaysia | both | 58962.37(40367.79,84034.86) | 32772.92(22100.03,47064.68) | -44.42 | 850.54(843.08,858.05) | 430.48(425.40,435.62) | -2.30(-2.38,-2.22) |
| Maldives | both | 4720.86(3341.59,6659.00) | 620.14(389.66,970.95) | -86.86 | 3901.35(3780.15,4025.57) | 630.37(578.34,686.11) | -5.89(-6.45,-5.32) |
| Mali | both | 632095.61(431489.55,896942.35) | 807729.45(591617.87,1070435.33) | 27.79 | 11994.09(11959.15,12029.11) | 5885.92(5871.53,5900.33) | -2.28(-2.62,-1.94) |
| Malta | both | 148.96(88.87,244.54) | 58.82(33.76,96.91) | -60.51 | 178.06(148.97,211.49) | 94.83(71.04,124.43) | -2.16(-2.26,-2.06) |
| Marshall Islands | both | 264.09(185.19,362.77) | 156.17(105.45,220.80) | -40.86 | 1001.61(865.71,1153.96) | 793.20(656.67,951.47) | -0.84(-1.02,-0.67) |
| Mauritania | both | 48923.03(34135.99,69735.87) | 23665.18(15427.41,36295.82) | -51.63 | 4260.52(4216.87,4304.52) | 1216.89(1200.19,1233.78) | -3.96(-4.08,-3.85) |
| Mauritius | both | 3391.96(2428.43,5040.46) | 932.05(597.85,1455.98) | -72.52 | 993.33(956.32,1031.49) | 454.54(422.77,488.18) | -2.12(-2.31,-1.92) |
| Mexico | both | 732590.63(652852.19,822459.88) | 107949.78(83053.60,140050.32) | -85.26 | 1763.77(1759.11,1768.44) | 315.80(313.68,317.93) | -5.37(-5.55,-5.18) |
| Micronesia (Federated States of) | both | 665.25(474.06,876.70) | 226.75(149.64,328.06) | -65.92 | 1152.30(1049.99,1262.38) | 696.82(597.69,808.72) | -1.69(-1.76,-1.62) |
| Monaco | both | 2.26(1.33,3.66) | 2.45(1.26,4.19) | 8.45 | 65.63(7.88,259.90) | 50.46(7.19,185.68) | -0.93(-0.97,-0.88) |
| Mongolia | both | 13291.17(8897.88,19059.20) | 9199.33(5752.26,13711.34) | -30.79 | 1438.06(1412.24,1464.25) | 826.98(809.09,845.19) | -2.09(-2.22,-1.96) |
| Montenegro | both | 533.08(337.65,809.26) | 258.58(154.31,409.36) | -51.49 | 328.25(299.06,359.68) | 230.15(201.06,262.55) | -1.58(-1.80,-1.36) |
| Morocco | both | 125800.90(88985.90,172093.47) | 55021.74(35990.08,82129.59) | -56.26 | 1189.63(1182.53,1196.76) | 554.94(549.96,559.95) | -2.47(-2.63,-2.30) |
| Mozambique | both | 770801.05(527635.03,1106268.04) | 317526.80(236647.89,434526.84) | -58.81 | 10191.27(10164.71,10217.89) | 1937.80(1930.26,1945.36) | -5.62(-5.83,-5.40) |
| Myanmar | both | 394739.61(279391.20,525284.39) | 146067.76(93274.77,223644.06) | -63.00 | 2480.91(2472.37,2489.48) | 877.08(872.20,881.98) | -3.65(-3.78,-3.53) |
| Namibia | both | 23540.97(18049.12,30243.31) | 13588.87(9535.98,18351.66) | -42.28 | 3089.28(3042.56,3136.57) | 1353.84(1326.97,1381.14) | -2.35(-2.54,-2.15) |
| Nauru | both | 58.43(41.38,77.88) | 36.90(25.43,52.12) | -36.85 | 903.71(631.06,1259.09) | 700.81(451.79,1044.94) | -0.86(-1.33,-0.38) |
| Nepal | both | 680650.38(492910.44,948824.88) | 102760.46(71533.42,148616.47) | -84.90 | 6263.38(6246.29,6280.50) | 1061.71(1054.71,1068.75) | -5.69(-5.99,-5.39) |
| Netherlands | both | 3890.17(2285.53,6165.90) | 2471.16(1366.80,3944.54) | -36.48 | 146.39(141.56,151.35) | 96.11(92.16,100.20) | -1.42(-1.56,-1.29) |
| New Zealand | both | 860.12(362.31,2253.28) | 673.74(263.33,1989.55) | -21.67 | 93.13(86.30,100.38) | 64.93(59.64,70.60) | -1.03(-1.16,-0.90) |
| Nicaragua | both | 32994.81(26408.15,40961.10) | 8759.77(6214.94,12196.90) | -73.45 | 1544.03(1525.62,1562.62) | 396.94(387.63,406.42) | -4.83(-5.08,-4.57) |
| Niger | both | 566994.29(377322.18,876909.51) | 313339.87(227930.37,422566.65) | -44.74 | 10081.45(10049.54,10113.44) | 2053.55(2045.42,2061.70) | -5.70(-6.01,-5.40) |
| Nigeria | both | 1469970.57(1125453.10,1968794.12) | 1651044.76(1164597.82,2326016.83) | 12.32 | 2858.12(2852.65,2863.60) | 1493.06(1490.58,1495.55) | -2.13(-2.36,-1.91) |
| Niue | both | 7.09(4.97,10.06) | 3.79(2.91,4.91) | -46.56 | 725.88(232.49,1759.29) | 820.02(137.22,2762.11) | -0.81(-1.26,-0.36) |
| North Macedonia | both | 3012.35(1893.94,4519.58) | 977.71(599.98,1527.37) | -67.54 | 572.00(550.35,594.34) | 299.86(279.98,320.88) | -2.35(-2.49,-2.20) |
| Northern Mariana Islands | both | 63.13(42.75,90.52) | 39.61(26.34,57.88) | -37.25 | 416.33(305.40,556.80) | 345.01(233.56,494.87) | -0.21(-0.39,-0.03) |
| Norway | both | 1011.51(581.61,1640.94) | 637.36(350.74,1053.55) | -36.99 | 130.15(121.75,139.00) | 72.89(67.01,79.19) | -1.94(-2.20,-1.67) |
| Oman | both | 12577.19(9451.06,17476.57) | 5065.67(3286.85,7709.71) | -59.72 | 1258.76(1234.44,1283.45) | 391.54(379.87,403.50) | -3.21(-3.64,-2.77) |
| Pakistan | both | 1793885.00(1388130.58,2322753.77) | 1836218.62(1292076.50,2534169.05) | 2.36 | 3188.12(3182.98,3193.28) | 2076.11(2072.88,2079.33) | -1.17(-1.28,-1.05) |
| Palau | both | 34.08(23.52,48.67) | 15.25(9.92,23.28) | -55.25 | 646.87(419.36,962.24) | 452.80(231.57,811.62) | -1.00(-1.08,-0.92) |
| Palestine | both | 6861.97(4571.51,9997.57) | 5694.62(3477.70,8806.06) | -17.01 | 620.35(604.25,636.78) | 297.10(288.87,305.52) | -2.45(-2.53,-2.38) |
| Panama | both | 8475.94(7043.05,10344.78) | 5051.93(3960.11,6646.61) | -40.40 | 809.48(789.30,830.08) | 310.32(299.45,321.51) | -3.58(-3.94,-3.23) |
| Papua New Guinea | both | 19505.94(13858.09,26464.74) | 39452.96(26224.22,60419.65) | 102.26 | 881.15(866.64,895.85) | 813.45(804.21,822.77) | 0.11(-0.12,0.33) |
| Paraguay | both | 18315.16(14095.41,23883.76) | 10893.75(7960.01,15164.78) | -40.52 | 908.82(893.93,923.91) | 550.21(539.23,561.37) | -1.77(-2.12,-1.42) |
| Peru | both | 249448.46(191320.87,315090.08) | 49197.83(34885.18,67877.15) | -80.28 | 2274.68(2263.94,2285.46) | 441.82(437.41,446.27) | -5.65(-5.93,-5.37) |
| Philippines | both | 466357.74(384250.60,573104.71) | 240805.11(171798.87,328429.83) | -48.36 | 1637.53(1632.26,1642.81) | 686.84(683.82,689.87) | -2.45(-2.63,-2.27) |
| Poland | both | 48195.15(29494.77,73181.44) | 13928.51(8490.09,22233.56) | -71.10 | 511.24(506.36,516.17) | 236.95(232.76,241.20) | -2.57(-2.68,-2.47) |
| Portugal | both | 3932.54(2241.77,6237.84) | 1301.05(723.74,2200.05) | -66.92 | 203.99(197.15,211.03) | 102.26(96.45,108.35) | -2.39(-2.62,-2.17) |
| Puerto Rico | both | 4892.72(3310.20,7154.54) | 1188.39(751.43,1787.80) | -75.71 | 478.93(464.43,493.78) | 286.73(268.75,305.72) | -1.78(-2.06,-1.51) |
| Qatar | both | 667.26(418.82,990.36) | 1198.69(760.82,1835.50) | 79.64 | 487.69(448.28,529.73) | 228.56(214.78,243.03) | -2.80(-2.95,-2.66) |
| Republic of Korea | both | 11949.78(7090.11,18962.50) | 2034.89(1156.36,3375.10) | -82.97 | 103.61(101.60,105.65) | 34.38(32.77,36.05) | -3.40(-3.83,-2.97) |
| Republic of Moldova | both | 5921.99(3944.13,8487.50) | 1287.52(809.03,1892.98) | -78.26 | 458.87(446.36,471.65) | 242.86(228.74,257.69) | -2.20(-2.35,-2.05) |
| Romania | both | 31518.15(20060.26,48149.86) | 8290.08(5163.17,12359.80) | -73.70 | 575.40(568.60,582.26) | 276.33(269.96,282.83) | -2.54(-2.64,-2.45) |
| Russian Federation | both | 139998.69(92153.19,213518.03) | 58617.06(36656.69,89925.46) | -58.13 | 398.32(396.09,400.56) | 225.86(223.89,227.84) | -2.32(-2.62,-2.03) |
| Rwanda | both | 305626.21(212856.53,423978.27) | 65147.94(48621.51,85930.62) | -78.68 | 7335.04(7305.34,7364.84) | 1165.16(1155.11,1175.28) | -7.40(-8.07,-6.72) |
| Saint Kitts and Nevis | both | 300.19(251.62,360.91) | 86.57(65.00,113.24) | -71.16 | 1806.95(1578.52,2061.17) | 731.05(560.20,941.39) | -2.70(-3.24,-2.15) |
| Saint Lucia | both | 695.63(527.30,901.87) | 188.51(129.81,276.63) | -72.90 | 1200.83(1102.85,1305.67) | 616.26(523.08,722.60) | -2.05(-2.40,-1.69) |
| Saint Vincent and the Grenadines | both | 753.50(591.94,948.63) | 215.12(159.31,295.65) | -71.45 | 1714.15(1579.70,1857.76) | 823.06(703.98,958.02) | -2.31(-2.74,-1.87) |
| Samoa | both | 772.95(551.24,1077.30) | 529.12(346.92,773.46) | -31.55 | 840.42(770.91,914.84) | 554.40(501.35,611.79) | -1.25(-1.34,-1.16) |
| San Marino | both | 4.10(2.31,6.55) | 3.13(1.76,5.58) | -23.63 | 109.71(26.71,314.80) | 77.95(14.35,259.75) | -1.09(-1.32,-0.85) |
| Sao Tome and Principe | both | 4151.02(3121.15,5208.75) | 977.85(664.07,1464.60) | -76.44 | 6040.81(5829.82,6257.87) | 1237.33(1155.35,1323.99) | -5.17(-5.70,-4.63) |
| Saudi Arabia | both | 78702.42(55770.31,107057.47) | 25043.58(15964.09,36961.05) | -68.18 | 1109.30(1100.89,1117.77) | 334.84(330.39,339.34) | -3.65(-3.72,-3.58) |
| Senegal | both | 209670.40(156501.29,272969.76) | 116312.64(74598.84,167491.23) | -44.53 | 4891.69(4867.90,4915.57) | 1731.75(1721.07,1742.47) | -3.70(-3.88,-3.52) |
| Serbia | both | 10489.63(6430.87,16649.30) | 3186.60(2008.21,4947.87) | -69.62 | 484.75(474.81,494.86) | 248.71(239.39,258.33) | -2.44(-2.57,-2.32) |
| Seychelles | both | 150.73(103.03,214.45) | 94.02(63.04,136.85) | -37.63 | 612.71(510.99,729.88) | 386.75(306.14,483.28) | -1.32(-1.44,-1.19) |
| Sierra Leone | both | 247671.80(174941.99,333225.48) | 290100.91(194743.59,422458.64) | 17.13 | 9555.12(9510.41,9600.00) | 5871.21(5845.37,5897.15) | -0.72(-1.20,-0.24) |
| Singapore | both | 591.87(335.73,1026.70) | 260.38(140.27,453.50) | -56.01 | 88.06(80.36,96.38) | 30.68(26.82,34.97) | -3.32(-3.73,-2.90) |
| Slovakia | both | 5530.60(3393.41,8281.77) | 1972.25(1240.31,2915.33) | -64.34 | 423.16(411.25,435.35) | 227.52(216.90,238.55) | -2.15(-2.20,-2.10) |
| Slovenia | both | 1297.59(800.18,1979.79) | 500.81(313.68,840.55) | -61.40 | 321.42(302.86,340.90) | 160.00(145.33,175.84) | -2.58(-2.73,-2.43) |
| Solomon Islands | both | 2507.08(1846.98,3363.73) | 2570.57(1801.25,3705.39) | 2.53 | 1144.72(1090.88,1200.67) | 829.59(793.05,867.45) | -0.85(-0.95,-0.76) |
| Somalia | both | 497368.94(332218.62,690677.34) | 424956.15(301233.61,588145.89) | -14.56 | 11137.38(11102.68,11172.15) | 3585.81(3574.08,3597.58) | -2.51(-5.10,0.15) |
| South Africa | both | 557389.46(463179.18,678324.54) | 302040.92(234312.25,381646.52) | -45.81 | 3004.46(2994.67,3014.27) | 1847.95(1840.63,1855.30) | -1.14(-1.31,-0.98) |
| South Sudan | both | 464187.53(323647.83,673733.25) | 274252.61(188992.05,402018.56) | -40.92 | 15081.28(15032.19,15130.49) | 5640.66(5616.68,5664.72) | -3.25(-3.87,-2.62) |
| Spain | both | 10545.43(5823.88,17103.88) | 5030.64(2751.65,8319.59) | -52.30 | 151.06(147.95,154.23) | 84.91(82.42,87.46) | -2.03(-2.23,-1.84) |
| Sri Lanka | both | 52129.88(36255.56,72551.85) | 23488.82(15765.02,34391.17) | -54.94 | 943.71(934.79,952.69) | 478.91(472.18,485.71) | -2.29(-2.40,-2.18) |
| Sudan | both | 484497.36(323730.08,745031.39) | 193991.18(135951.16,284154.66) | -59.96 | 4348.45(4334.37,4362.57) | 1102.09(1096.73,1107.48) | -4.37(-4.51,-4.23) |
| Suriname | both | 2346.42(1774.29,3132.11) | 1123.09(765.37,1606.91) | -52.14 | 1711.14(1637.21,1787.77) | 768.32(719.98,819.28) | -2.68(-2.92,-2.44) |
| Sweden | both | 1913.31(1094.86,3132.49) | 1414.37(734.32,2487.18) | -26.08 | 125.23(119.34,131.35) | 81.47(77.05,86.08) | -1.47(-1.63,-1.31) |
| Switzerland | both | 1298.52(728.65,2127.91) | 941.48(512.40,1663.87) | -27.50 | 114.67(108.12,121.53) | 73.32(68.46,78.45) | -1.62(-1.81,-1.43) |
| Syrian Arab Republic | both | 85012.69(62652.27,116056.33) | 21031.77(13942.38,30423.97) | -75.26 | 1352.48(1342.70,1362.32) | 608.53(599.33,617.85) | -2.25(-2.59,-1.91) |
| Taiwan (Province of China) | both | 14351.85(10009.36,20270.62) | 3450.11(2226.84,5304.82) | -75.96 | 262.45(257.70,267.27) | 118.99(114.67,123.44) | -2.59(-2.99,-2.19) |
| Tajikistan | both | 37328.19(26322.86,49921.19) | 37564.06(24419.32,53451.71) | 0.63 | 1459.38(1443.35,1475.54) | 1002.28(991.44,1013.21) | -1.57(-1.82,-1.32) |
| Thailand | both | 103454.54(72845.63,145949.13) | 29249.88(19663.01,42371.51) | -71.73 | 614.05(609.94,618.19) | 308.79(304.89,312.74) | -2.09(-2.24,-1.94) |
| Timor-Leste | both | 32083.71(19143.25,58331.66) | 6895.75(5110.03,9521.91) | -78.51 | 7907.44(7813.44,8002.38) | 1198.09(1166.87,1229.97) | -6.61(-7.07,-6.15) |
| Togo | both | 72163.26(52350.77,96189.79) | 67268.36(43006.46,98918.52) | -6.78 | 3582.64(3553.42,3612.05) | 1925.62(1910.11,1941.23) | -2.18(-2.47,-1.89) |
| Tokelau | both | 6.99(4.92,9.74) | 3.70(2.67,4.77) | -47.06 | 855.76(249.24,2194.63) | 892.48(139.82,3085.81) | -1.19(-1.74,-0.64) |
| Tonga | both | 467.47(343.38,630.37) | 294.09(209.15,407.55) | -37.09 | 790.71(704.18,885.50) | 578.66(501.96,664.25) | -0.87(-0.97,-0.78) |
| Trinidad and Tobago | both | 4310.33(3131.41,5825.57) | 1422.17(918.57,2113.43) | -67.01 | 1031.02(997.86,1065.05) | 525.18(495.85,555.90) | -2.52(-2.67,-2.37) |
| Tunisia | both | 24215.99(16640.91,33662.69) | 10357.95(6611.99,15879.92) | -57.23 | 765.71(755.41,776.13) | 374.17(366.46,382.01) | -2.29(-2.40,-2.18) |
| Türkiye | both | 260389.09(192764.63,356785.97) | 64569.23(41805.52,93455.16) | -75.20 | 1207.13(1202.13,1212.15) | 349.92(346.99,352.88) | -4.09(-4.30,-3.88) |
| Turkmenistan | both | 21113.90(14622.13,28797.41) | 11879.90(7565.00,17772.86) | -43.73 | 1279.56(1260.80,1298.53) | 761.56(747.00,776.35) | -1.85(-1.94,-1.76) |
| Tuvalu | both | 92.31(69.72,119.77) | 28.44(19.13,40.36) | -69.19 | 1542.33(1160.68,2019.56) | 657.25(409.12,1008.81) | -2.54(-2.72,-2.36) |
| Uganda | both | 632998.70(437745.78,875696.15) | 321489.05(210620.81,452393.99) | -49.21 | 5045.46(5030.61,5060.35) | 1318.87(1313.61,1324.15) | -4.45(-4.67,-4.23) |
| Ukraine | both | 65889.47(47491.48,90430.78) | 21589.80(13944.79,32095.61) | -67.23 | 570.59(565.88,575.33) | 362.24(356.86,367.68) | -1.76(-2.00,-1.51) |
| United Arab Emirates | both | 4794.22(3381.27,6905.90) | 5411.74(3406.15,8016.01) | 12.88 | 748.26(725.32,771.78) | 400.26(388.90,411.89) | -1.94(-2.07,-1.82) |
| United Kingdom | both | 22076.64(13904.92,33882.56) | 17282.56(11006.82,27975.09) | -21.72 | 198.06(195.25,200.91) | 153.91(151.45,156.40) | -0.80(-0.91,-0.68) |
| United Republic of Tanzania | both | 1219054.83(904078.39,1590880.22) | 484350.11(352628.61,683764.26) | -60.27 | 7905.57(7889.67,7921.49) | 1640.50(1635.19,1645.83) | -5.37(-5.57,-5.17) |
| United States of America | both | 27952.76(17553.05,45070.95) | 32014.02(21935.44,46870.61) | 14.53 | 46.56(45.97,47.15) | 52.24(51.64,52.85) | 0.85(0.55,1.16) |
| United States Virgin Islands | both | 224.99(161.96,330.68) | 49.90(31.35,76.07) | -77.82 | 658.08(567.69,759.70) | 371.31(267.01,506.20) | -1.80(-2.06,-1.54) |
| Uruguay | both | 4255.63(3426.16,5596.48) | 817.86(483.76,1441.37) | -80.78 | 475.23(459.69,491.21) | 118.54(109.48,128.19) | -4.43(-4.64,-4.22) |
| Uzbekistan | both | 134092.51(88834.37,192323.24) | 101128.22(63087.11,152227.22) | -24.58 | 1450.77(1442.43,1459.16) | 970.84(964.50,977.22) | -1.59(-1.67,-1.50) |
| Vanuatu | both | 918.05(663.14,1226.10) | 1155.92(810.52,1606.18) | 25.91 | 948.56(875.41,1026.48) | 829.68(775.49,886.82) | -0.58(-0.77,-0.40) |
| Venezuela (Bolivarian Republic of) | both | 89278.42(76977.13,105954.13) | 33010.04(24514.60,44160.46) | -63.03 | 970.57(963.15,978.03) | 408.32(403.17,413.53) | -3.38(-4.01,-2.75) |
| Viet Nam | both | 345813.24(252256.07,468615.88) | 96604.90(63502.98,139908.43) | -72.06 | 1247.31(1242.79,1251.84) | 386.22(383.57,388.88) | -3.91(-4.06,-3.75) |
| Yemen | both | 393497.33(292647.24,532564.70) | 402359.66(296735.08,536297.08) | 2.25 | 4690.78(4674.69,4706.91) | 2828.67(2819.26,2838.12) | -1.82(-1.91,-1.73) |
| Zambia | both | 372390.43(268786.97,533435.14) | 179801.17(126443.14,243360.11) | -51.72 | 6949.42(6922.07,6976.85) | 1954.56(1944.60,1964.56) | -4.36(-4.79,-3.93) |
| Zimbabwe | both | 150377.29(113176.89,190063.46) | 232888.96(176724.94,301519.77) | 54.87 | 2644.92(2629.82,2660.09) | 2820.62(2806.69,2834.61) | 0.82(0.51,1.13) |
| Global | female | 28867890.30(24026370.94,35163959.01) | 11888839.85(9056934.59,15680395.26) | -58.82 | 3044.34(3043.09,3045.58) | 1180.92(1180.19,1181.66) | -3.16(-3.43,-2.88) |
| Central Europe, eastern Europe, and central Asia | female | 350892.25(244360.56,508303.07) | 198899.30(132625.47,293665.77) | -43.32 | 656.16(653.84,658.49) | 502.50(500.14,504.86) | -1.25(-1.39,-1.10) |
| High-income | female | 160223.01(116035.99,223334.65) | 86101.37(57991.24,127375.76) | -46.26 | 168.48(167.57,169.39) | 102.93(102.19,103.67) | -1.55(-1.72,-1.39) |
| Latin America and Caribbean | female | 1630147.03(1452937.68,1854981.79) | 490554.81(360071.82,662754.27) | -69.91 | 1947.81(1944.42,1951.20) | 655.12(653.13,657.12) | -3.61(-3.76,-3.45) |
| Southeast Asia, east Asia, and Oceania | female | 3189844.24(2709325.22,3863933.73) | 739273.28(535101.04,992725.78) | -76.82 | 1192.30(1190.84,1193.76) | 355.44(354.56,356.32) | -5.51(-6.67,-4.33) |
| Sub-Saharan Africa | female | 8895750.96(6975529.89,11658545.86) | 5167187.33(4042792.75,6512113.89) | -41.91 | 6288.89(6284.07,6293.71) | 1954.10(1952.22,1955.97) | -3.80(-4.22,-3.37) |
| Andean Latin America | female | 240005.93(195638.38,292613.70) | 51515.38(38271.66,69460.18) | -78.54 | 2349.18(2337.61,2360.79) | 506.15(501.18,511.16) | -5.21(-5.44,-4.97) |
| Australasia | female | 1225.05(685.80,2164.59) | 1207.95(677.10,2234.68) | -1.40 | 51.71(48.65,54.91) | 42.39(39.89,45.02) | -0.57(-0.70,-0.45) |
| Caribbean | female | 191530.43(149961.14,237920.73) | 90016.06(62038.37,124037.48) | -53.00 | 2932.70(2918.07,2947.38) | 1529.74(1519.02,1540.52) | -1.80(-2.11,-1.48) |
| Central Asia | female | 165806.18(115676.03,228690.72) | 120884.44(80262.62,178262.79) | -27.09 | 1260.23(1253.72,1266.76) | 884.00(878.72,889.30) | -1.44(-1.54,-1.33) |
| Central Europe | female | 60232.33(39212.11,88435.48) | 21403.06(13891.37,31314.76) | -64.47 | 415.82(412.26,419.40) | 245.04(241.53,248.59) | -1.91(-1.99,-1.83) |
| Central Latin America | female | 578499.55(524321.43,643955.58) | 132384.34(103268.87,170957.67) | -77.12 | 1424.02(1419.75,1428.31) | 374.48(372.17,376.80) | -4.17(-4.30,-4.05) |
| Central Sub-Saharan Africa | female | 1026117.36(701647.62,1510437.12) | 439362.01(307019.61,596941.69) | -57.18 | 6373.81(6359.64,6388.00) | 1416.37(1411.83,1420.93) | -4.99(-5.42,-4.56) |
| East Asia | female | 1691098.54(1417493.13,2001395.74) | 174560.31(119930.02,242443.00) | -89.68 | 945.61(944.02,947.21) | 145.68(144.95,146.41) | -9.20(-11.37,-6.97) |
| Eastern Europe | female | 124853.74(82422.69,186624.90) | 56611.80(36339.68,85612.34) | -54.66 | 485.61(482.74,488.49) | 327.41(324.51,330.33) | -1.58(-1.74,-1.42) |
| Eastern Sub-Saharan Africa | female | 4853990.07(3705444.42,6273070.85) | 2001539.60(1582089.46,2487050.50) | -58.77 | 8660.58(8651.66,8669.51) | 2044.84(2041.68,2048.00) | -4.61(-5.36,-3.87) |
| High-income Asia Pacific | female | 11554.11(7079.57,18343.90) | 5060.20(2931.87,8217.47) | -56.20 | 67.39(66.08,68.73) | 46.69(45.32,48.09) | -1.11(-1.28,-0.94) |
| High-income North America | female | 13131.70(8107.85,21890.61) | 15267.50(9529.70,22903.48) | 16.26 | 41.56(40.81,42.32) | 46.15(45.39,46.92) | 0.40(0.25,0.54) |
| North Africa and Middle East | female | 1215529.41(948165.00,1564609.66) | 721923.20(524872.72,1004598.08) | -40.61 | 1598.33(1595.22,1601.44) | 794.54(792.58,796.51) | -2.35(-2.44,-2.25) |
| Oceania | female | 14166.68(10676.78,19725.07) | 21551.51(14729.17,32089.31) | 52.13 | 793.72(777.82,809.88) | 707.65(696.67,718.78) | -0.06(-0.25,0.14) |
| South Asia | female | 13425503.41(10806137.59,16506182.68) | 4484900.55(3223785.48,6225210.09) | -66.59 | 5792.40(5788.98,5795.81) | 1849.05(1847.22,1850.89) | -3.48(-3.54,-3.42) |
| Southeast Asia | female | 1484579.02(1187652.40,1890457.93) | 543161.46(395572.81,734621.61) | -63.41 | 1670.66(1667.68,1673.64) | 633.31(631.48,635.15) | -3.13(-3.23,-3.04) |
| Southern Latin America | female | 48405.65(40318.90,62438.38) | 9535.86(5808.58,16605.15) | -80.30 | 512.88(507.54,518.26) | 126.87(124.01,129.79) | -4.51(-4.78,-4.24) |
| Southern Sub-Saharan Africa | female | 379571.12(314132.72,458554.04) | 299259.20(239111.81,367545.61) | -21.16 | 2903.72(2892.64,2914.82) | 2141.41(2132.54,2150.32) | -0.46(-0.69,-0.23) |
| Tropical Latin America | female | 620111.12(511192.09,770894.48) | 216639.04(141363.90,320977.36) | -65.06 | 2266.58(2260.46,2272.73) | 865.66(861.84,869.50) | -3.35(-3.51,-3.19) |
| Western Europe | female | 85906.50(55831.13,128843.45) | 55029.85(36436.18,80874.76) | -35.94 | 262.69(260.81,264.58) | 176.53(174.96,178.11) | -1.37(-1.50,-1.24) |
| Western Sub-Saharan Africa | female | 2636072.41(2025815.85,3561400.34) | 2427026.52(1832643.84,3130787.58) | -7.93 | 4606.16(4599.58,4612.75) | 2014.64(2011.83,2017.45) | -2.71(-2.82,-2.61) |
| Afghanistan | female | 77994.83(54065.05,107047.34) | 79588.66(56055.76,112183.25) | 2.04 | 2938.81(2915.91,2961.86) | 1038.66(1030.93,1046.43) | -3.51(-3.83,-3.18) |
| Albania | female | 4488.21(2974.30,6882.85) | 660.06(418.88,1001.72) | -85.29 | 797.03(771.92,822.78) | 285.82(262.09,311.27) | -3.83(-4.05,-3.61) |
| Algeria | female | 56625.00(38471.69,80146.15) | 34842.72(21035.86,53657.22) | -38.47 | 1057.46(1048.20,1066.78) | 525.03(519.17,530.94) | -2.54(-2.65,-2.42) |
| American Samoa | female | 106.81(78.20,141.21) | 45.22(31.96,62.82) | -57.66 | 765.41(593.45,974.28) | 601.31(408.71,860.31) | -0.79(-0.91,-0.67) |
| Andorra | female | 8.25(4.37,13.47) | 6.83(3.32,11.34) | -17.26 | 203.39(81.36,433.44) | 153.58(54.70,359.05) | -0.95(-1.14,-0.76) |
| Angola | female | 370611.78(232091.98,583139.56) | 114965.05(76152.99,156550.51) | -68.98 | 12613.54(12566.48,12660.74) | 1397.28(1388.50,1406.11) | -7.35(-7.62,-7.08) |
| Antigua and Barbuda | female | 94.59(65.74,144.04) | 57.69(39.28,88.21) | -39.01 | 948.77(747.58,1190.96) | 637.28(466.16,855.90) | -1.30(-1.47,-1.12) |
| Argentina | female | 38835.77(31619.45,51213.26) | 6727.41(3619.06,12853.34) | -82.68 | 607.15(600.04,614.34) | 127.68(124.22,131.22) | -5.05(-5.40,-4.69) |
| Armenia | female | 5261.79(3762.71,7386.51) | 1684.70(1033.63,2593.73) | -67.98 | 986.50(958.36,1015.29) | 605.54(575.00,637.38) | -1.68(-1.81,-1.55) |
| Australia | female | 970.02(528.69,1798.93) | 973.23(532.86,1729.55) | 0.33 | 50.36(47.05,53.87) | 41.41(38.70,44.28) | -0.55(-0.70,-0.41) |
| Austria | female | 1514.28(820.95,2565.28) | 917.22(478.81,1646.59) | -39.43 | 241.08(228.27,254.45) | 152.55(142.32,163.35) | -1.61(-1.82,-1.40) |
| Azerbaijan | female | 17919.61(12877.16,24321.97) | 8691.82(5584.29,12850.70) | -51.50 | 1419.59(1397.47,1441.98) | 799.18(781.34,817.37) | -2.24(-2.39,-2.09) |
| Bahamas | female | 387.95(271.64,555.00) | 254.13(153.80,393.35) | -34.49 | 936.84(838.26,1044.80) | 636.67(552.90,731.01) | -1.18(-1.39,-0.98) |
| Bahrain | female | 609.47(395.39,896.96) | 555.23(326.02,913.42) | -8.90 | 720.00(659.99,784.23) | 381.91(348.40,417.97) | -2.06(-2.22,-1.91) |
| Bangladesh | female | 2948282.15(2353249.90,3549671.43) | 217298.85(149085.96,308467.61) | -92.63 | 10824.02(10810.39,10837.65) | 941.82(937.52,946.14) | -8.07(-8.35,-7.78) |
| Barbados | female | 288.17(199.29,439.58) | 140.29(87.01,219.85) | -51.32 | 905.33(795.28,1027.66) | 611.47(506.90,733.34) | -1.19(-1.34,-1.03) |
| Belarus | female | 5483.21(3539.42,8052.73) | 2307.27(1436.57,3662.02) | -57.92 | 459.96(447.13,473.08) | 301.17(288.19,314.63) | -1.66(-1.79,-1.54) |
| Belgium | female | 2121.38(1158.85,3406.85) | 1474.49(776.80,2471.07) | -30.49 | 253.60(242.18,265.46) | 168.77(159.75,178.20) | -1.47(-1.62,-1.32) |
| Belize | female | 976.48(805.56,1229.29) | 577.16(376.42,864.46) | -40.89 | 2041.50(1899.90,2191.49) | 895.45(816.82,980.22) | -2.38(-2.74,-2.03) |
| Benin | female | 84025.24(54747.76,126508.31) | 46560.02(31009.84,67464.60) | -44.59 | 5629.13(5585.16,5673.38) | 1377.30(1363.58,1391.14) | -4.62(-4.86,-4.38) |
| Bermuda | female | 31.71(20.48,48.06) | 13.12(8.09,20.78) | -58.63 | 487.52(318.79,718.80) | 312.40(156.96,571.03) | -1.42(-1.60,-1.25) |
| Bhutan | female | 4243.25(2515.80,6546.71) | 1979.75(1103.90,2988.42) | -53.34 | 2964.12(2866.10,3064.80) | 2108.10(2010.39,2209.68) | -1.55(-1.70,-1.40) |
| Bolivia (Plurinational State of) | female | 89777.52(64094.46,124562.80) | 21575.41(15434.68,29755.13) | -75.97 | 4422.93(4386.44,4459.67) | 1095.32(1078.63,1112.20) | -4.69(-4.86,-4.51) |
| Bosnia and Herzegovina | female | 2271.38(1296.89,3573.82) | 664.98(391.08,1075.04) | -70.72 | 422.42(404.09,441.42) | 278.71(256.49,302.48) | -1.72(-1.89,-1.55) |
| Botswana | female | 6879.43(4821.48,9578.20) | 6101.54(4084.18,8513.69) | -11.31 | 1871.82(1819.17,1925.67) | 1350.35(1308.61,1393.14) | -0.66(-0.85,-0.47) |
| Brazil | female | 609986.64(501959.75,758640.40) | 211150.73(136568.87,315114.88) | -65.38 | 2314.85(2308.55,2321.17) | 878.30(874.37,882.25) | -3.37(-3.54,-3.21) |
| Brunei Darussalam | female | 39.35(21.96,65.99) | 29.70(15.79,51.21) | -24.53 | 78.04(53.29,110.85) | 62.68(40.85,92.99) | -0.64(-0.83,-0.45) |
| Bulgaria | female | 3444.52(2008.90,5266.29) | 1425.69(833.06,2177.20) | -58.61 | 400.62(386.26,415.41) | 295.05(278.74,312.13) | -1.17(-1.28,-1.06) |
| Burkina Faso | female | 209698.36(138416.72,311570.68) | 202909.29(143105.21,279800.45) | -3.24 | 6755.42(6720.73,6790.26) | 3269.41(3253.40,3285.49) | -2.23(-2.47,-2.00) |
| Burundi | female | 189149.03(108550.71,347121.14) | 58591.62(35118.53,93374.18) | -69.02 | 11886.28(11825.29,11947.53) | 1806.43(1790.36,1822.62) | -6.02(-6.24,-5.80) |
| Cabo Verde | female | 3548.99(2511.31,4835.58) | 684.14(441.40,1083.02) | -80.72 | 3846.32(3708.26,3988.49) | 951.66(876.55,1032.02) | -4.78(-5.28,-4.27) |
| Cambodia | female | 141076.58(91284.90,217782.11) | 24700.33(16297.49,37856.59) | -82.49 | 5118.04(5088.54,5147.68) | 937.22(924.66,949.91) | -6.30(-6.69,-5.90) |
| Cameroon | female | 61381.41(42322.11,84775.43) | 77504.03(51114.63,112802.82) | 26.27 | 1980.12(1961.80,1998.58) | 1064.65(1056.43,1072.92) | -2.29(-2.45,-2.14) |
| Canada | female | 1006.86(597.82,1658.15) | 1140.22(605.89,2206.01) | 13.25 | 35.10(32.85,37.48) | 37.00(34.78,39.34) | 0.22(0.08,0.36) |
| Central African Republic | female | 35679.73(21571.70,58999.52) | 40841.85(26617.65,59782.29) | 14.47 | 4574.15(4520.67,4628.14) | 3125.20(3091.24,3159.45) | -0.97(-1.27,-0.67) |
| Chad | female | 129384.73(83849.33,211472.17) | 124292.42(86378.11,174101.43) | -3.94 | 7006.25(6962.06,7050.66) | 2415.47(2400.59,2430.43) | -3.64(-3.80,-3.48) |
| Chile | female | 7776.51(6322.00,9704.41) | 2427.43(1686.57,3579.17) | -68.79 | 322.11(314.02,330.36) | 128.56(123.01,134.31) | -2.98(-3.26,-2.70) |
| China | female | 1649724.86(1381489.52,1950505.17) | 165360.28(114147.65,228257.04) | -89.98 | 956.53(954.90,958.16) | 142.44(141.71,143.18) | -6.94(-7.49,-6.39) |
| Colombia | female | 62973.90(52838.22,76254.77) | 23820.31(17680.28,32050.78) | -62.17 | 885.47(877.59,893.40) | 388.79(383.08,394.58) | -2.13(-2.53,-1.73) |
| Comoros | female | 7356.65(4976.99,10495.13) | 2109.99(1455.40,3016.88) | -71.32 | 5885.31(5735.78,6037.96) | 1786.60(1705.05,1871.23) | -3.97(-4.12,-3.83) |
| Congo | female | 12762.13(8935.87,17597.47) | 11717.14(7739.43,17444.82) | -8.19 | 2227.61(2185.88,2269.97) | 1208.86(1185.57,1232.51) | -2.32(-2.73,-1.91) |
| Cook Islands | female | 16.31(10.08,24.73) | 6.09(3.83,9.30) | -62.68 | 490.96(264.92,842.30) | 337.25(109.73,821.06) | -1.22(-1.28,-1.17) |
| Costa Rica | female | 1727.62(1148.58,2499.68) | 1070.04(624.13,1653.78) | -38.06 | 291.34(276.59,306.71) | 215.89(202.08,230.45) | -0.93(-1.00,-0.87) |
| Croatia | female | 1577.64(955.74,2596.80) | 645.45(388.09,984.64) | -59.09 | 328.20(311.06,346.10) | 221.28(203.31,240.53) | -1.48(-1.55,-1.41) |
| Cuba | female | 7213.90(4309.07,11083.51) | 4248.46(2452.14,6576.69) | -41.11 | 574.85(560.79,589.19) | 493.11(477.37,509.28) | -0.45(-0.52,-0.37) |
| Cyprus | female | 237.93(123.37,392.99) | 146.02(78.23,251.85) | -38.63 | 262.19(227.57,300.88) | 144.63(120.89,171.86) | -2.32(-2.55,-2.10) |
| Czechia | female | 3651.03(2069.09,5763.10) | 1857.38(1127.57,2849.82) | -49.13 | 338.22(326.41,350.39) | 215.68(205.28,226.50) | -1.50(-1.61,-1.39) |
| C?te d'Ivoire | female | 92275.78(65224.20,131138.10) | 85824.44(54144.06,120801.71) | -6.99 | 2567.84(2548.89,2586.91) | 1393.69(1383.57,1403.87) | -2.06(-2.32,-1.81) |
| Democratic People's Republic of Korea | female | 33699.83(23651.54,48214.34) | 7000.99(4540.79,11250.37) | -79.23 | 925.32(914.19,936.55) | 291.65(284.10,299.37) | -12.24(-18.25,-5.78) |
| Democratic Republic of the Congo | female | 598328.48(381511.00,901463.84) | 265719.06(169605.09,397092.60) | -55.59 | 5185.37(5170.31,5200.47) | 1336.57(1331.08,1342.08) | -4.45(-5.02,-3.88) |
| Denmark | female | 985.78(513.99,1572.19) | 645.97(344.69,1076.47) | -34.47 | 246.08(229.80,263.26) | 149.05(137.10,161.81) | -1.94(-2.11,-1.78) |
| Djibouti | female | 4824.75(3191.84,7428.76) | 3734.42(2469.41,5314.11) | -22.60 | 5020.33(4860.34,5184.48) | 1792.62(1729.76,1857.27) | -3.57(-4.03,-3.11) |
| Dominica | female | 122.22(83.99,172.15) | 56.22(35.21,81.51) | -54.00 | 960.56(784.91,1166.37) | 860.95(629.46,1159.34) | -0.23(-0.41,-0.05) |
| Dominican Republic | female | 53156.31(42688.16,65556.33) | 13604.38(9148.63,19285.21) | -74.41 | 3234.76(3203.80,3265.98) | 905.20(889.03,921.61) | -3.53(-4.04,-3.02) |
| Ecuador | female | 30276.46(26797.77,34206.60) | 5343.27(4116.03,7131.10) | -82.35 | 1131.55(1116.05,1147.23) | 182.21(176.51,188.05) | -6.18(-6.87,-5.49) |
| Egypt | female | 118411.78(86676.85,156348.37) | 85841.09(55783.63,131118.36) | -27.51 | 963.66(957.65,969.71) | 454.81(451.54,458.10) | -2.14(-2.27,-2.01) |
| El Salvador | female | 14168.45(11233.31,17588.09) | 2845.98(1950.06,4059.84) | -79.91 | 1036.93(1017.32,1056.86) | 307.92(295.83,320.40) | -3.96(-4.19,-3.74) |
| Equatorial Guinea | female | 4394.15(2982.04,6484.17) | 2641.50(1613.25,4121.53) | -39.89 | 3728.22(3604.37,3855.39) | 1000.85(960.58,1042.47) | -5.50(-6.00,-5.00) |
| Eritrea | female | 125221.54(85590.21,182431.53) | 33092.54(23170.59,46468.48) | -73.57 | 13881.64(13793.32,13970.41) | 2369.70(2340.80,2398.88) | -5.55(-5.93,-5.16) |
| Estonia | female | 832.77(576.80,1270.93) | 288.13(172.01,460.70) | -65.40 | 472.07(438.44,507.73) | 269.55(237.41,305.14) | -2.05(-2.20,-1.90) |
| Eswatini | female | 4814.50(3320.10,6654.02) | 3000.65(2150.27,4197.83) | -37.67 | 1956.21(1891.04,2023.14) | 1300.06(1248.03,1353.83) | -0.92(-1.17,-0.67) |
| Ethiopia | female | 1497878.15(1098779.71,2038715.70) | 500327.90(379620.91,641919.94) | -66.60 | 10504.12(10484.90,10523.36) | 2124.37(2117.85,2130.90) | -5.82(-6.19,-5.45) |
| Fiji | female | 1184.42(811.36,1670.88) | 888.32(591.90,1248.65) | -25.00 | 775.00(725.59,827.06) | 577.08(533.87,623.03) | -1.01(-1.09,-0.92) |
| Finland | female | 1088.39(599.69,1762.93) | 542.72(297.94,840.83) | -50.14 | 243.61(228.30,259.74) | 145.31(132.50,159.12) | -1.93(-2.13,-1.74) |
| France | female | 15640.33(9136.87,25769.20) | 9116.37(5176.53,15236.54) | -41.71 | 283.22(278.49,288.00) | 172.62(168.86,176.44) | -1.83(-2.01,-1.65) |
| Gabon | female | 4341.09(2804.34,6519.19) | 3477.42(1690.10,5906.56) | -19.90 | 1922.77(1861.09,1986.08) | 1033.75(997.43,1071.14) | -2.22(-2.60,-1.84) |
| Gambia | female | 14510.82(10638.81,19287.83) | 11416.56(7556.68,15980.31) | -21.32 | 5469.52(5368.69,5571.82) | 2160.14(2116.95,2204.03) | -3.50(-3.76,-3.24) |
| Georgia | female | 5232.20(3187.03,7758.43) | 2229.06(1336.53,3398.85) | -57.40 | 780.10(757.81,802.92) | 630.18(602.56,658.80) | -0.71(-0.79,-0.64) |
| Germany | female | 14888.56(8138.07,23664.52) | 9109.72(5033.42,14855.37) | -38.81 | 242.61(238.46,246.81) | 162.68(159.20,166.23) | -1.20(-1.29,-1.10) |
| Ghana | female | 207248.41(139861.50,304903.04) | 107749.98(68551.56,163288.00) | -48.01 | 5001.54(4976.29,5026.88) | 1575.95(1565.66,1586.29) | -3.54(-3.71,-3.37) |
| Greece | female | 2147.60(1179.72,3422.02) | 1153.96(599.24,2019.24) | -46.27 | 247.84(236.61,259.49) | 186.90(175.57,198.81) | -1.09(-1.28,-0.90) |
| Greenland | female | 3.71(2.09,6.00) | 2.28(1.24,4.03) | -38.49 | 52.15(12.18,149.05) | 39.87(5.26,151.04) | -0.85(-0.94,-0.76) |
| Grenada | female | 204.11(145.84,286.72) | 83.48(54.72,124.58) | -59.10 | 1142.49(978.51,1327.45) | 754.03(588.59,955.43) | -1.12(-1.35,-0.90) |
| Guam | female | 123.65(88.46,172.67) | 81.94(54.81,117.59) | -33.73 | 469.41(376.87,578.94) | 389.91(298.13,502.57) | -0.48(-0.61,-0.35) |
| Guatemala | female | 90296.47(79226.61,102046.41) | 21704.04(17147.54,29179.50) | -75.96 | 3377.39(3351.43,3403.51) | 754.30(742.64,766.11) | -4.40(-4.71,-4.10) |
| Guinea | female | 138768.45(93362.14,197064.58) | 82207.19(58330.58,113720.56) | -40.76 | 7640.10(7592.49,7687.94) | 2382.62(2364.29,2401.06) | -3.73(-3.95,-3.50) |
| Guinea-Bissau | female | 17152.41(10403.80,28101.47) | 7387.77(4668.40,11219.29) | -56.93 | 6067.50(5963.29,6173.12) | 1615.50(1576.64,1655.12) | -4.44(-4.64,-4.24) |
| Guyana | female | 5260.84(4212.24,6501.18) | 1146.35(865.70,1613.42) | -78.21 | 3207.27(3114.60,3302.17) | 1090.02(1024.47,1158.90) | -2.54(-2.92,-2.15) |
| Haiti | female | 104042.89(73017.88,144350.28) | 62274.61(41369.25,88175.26) | -40.15 | 6149.31(6107.44,6191.42) | 2646.71(2624.39,2669.20) | -2.51(-2.78,-2.23) |
| Honduras | female | 14007.50(10681.84,17868.39) | 6932.10(4569.56,10010.52) | -50.51 | 1080.92(1060.74,1101.41) | 411.57(401.14,422.23) | -3.20(-3.42,-2.97) |
| Hungary | female | 3847.85(2276.57,6487.15) | 1607.86(952.32,2545.18) | -58.21 | 369.60(357.03,382.54) | 231.81(219.82,244.32) | -1.55(-1.65,-1.45) |
| Iceland | female | 60.63(33.24,97.49) | 40.70(22.43,67.77) | -32.88 | 204.35(152.65,268.95) | 131.07(91.70,182.68) | -1.66(-1.83,-1.49) |
| India | female | 9204916.55(7051134.58,11652388.04) | 3378054.71(2398720.29,4756878.32) | -63.30 | 5354.57(5350.77,5358.37) | 1965.35(1963.10,1967.60) | -3.00(-3.11,-2.89) |
| Indonesia | female | 704986.23(551491.90,908275.67) | 217420.20(159617.35,289970.13) | -69.16 | 2011.57(2006.31,2016.85) | 638.48(635.54,641.44) | -3.57(-3.67,-3.48) |
| Iran (Islamic Republic of) | female | 132787.87(98907.04,178270.69) | 44033.33(26741.74,69968.81) | -66.84 | 1050.54(1044.47,1056.64) | 451.12(446.61,455.67) | -2.50(-2.64,-2.35) |
| Iraq | female | 49493.10(36053.43,66223.90) | 36378.91(23209.33,55992.28) | -26.50 | 1145.76(1135.00,1156.60) | 554.32(548.22,560.47) | -2.61(-2.75,-2.47) |
| Ireland | female | 1120.97(603.62,1789.49) | 656.52(361.95,1059.66) | -41.43 | 257.25(241.33,273.99) | 144.45(132.96,156.73) | -2.12(-2.31,-1.92) |
| Israel | female | 2284.80(1332.15,3746.84) | 2481.44(1239.12,3867.42) | 8.61 | 318.47(304.68,332.76) | 199.39(191.20,207.86) | -1.68(-1.81,-1.55) |
| Italy | female | 8405.65(4612.76,14109.30) | 4885.28(2566.04,7762.52) | -41.88 | 208.80(204.01,213.67) | 143.77(139.50,148.15) | -1.16(-1.27,-1.05) |
| Jamaica | female | 6129.34(4821.35,7785.71) | 1875.12(1178.96,2864.61) | -69.41 | 1257.77(1221.85,1294.54) | 647.52(616.15,680.21) | -1.76(-2.19,-1.33) |
| Japan | female | 6530.66(3795.60,10537.47) | 3576.66(2024.15,5982.65) | -45.23 | 58.45(56.93,60.00) | 47.84(46.18,49.55) | -0.57(-0.69,-0.46) |
| Jordan | female | 6098.56(4000.08,9419.49) | 8838.12(5314.35,13842.19) | 44.92 | 725.72(706.37,745.50) | 486.81(475.97,497.85) | -1.45(-1.54,-1.35) |
| Kazakhstan | female | 31128.53(19673.20,46205.06) | 18700.53(11241.23,29648.19) | -39.92 | 1129.38(1115.79,1143.09) | 684.75(674.31,695.32) | -2.06(-2.22,-1.89) |
| Kenya | female | 263375.85(191212.65,338919.84) | 119465.29(95950.96,151233.16) | -54.64 | 3578.32(3562.15,3594.55) | 1157.91(1150.24,1165.62) | -3.25(-3.46,-3.05) |
| Kiribati | female | 1047.31(702.35,1491.42) | 459.21(313.53,634.86) | -56.15 | 3275.47(2986.61,3586.44) | 1406.61(1238.44,1592.55) | -2.80(-2.93,-2.68) |
| Kuwait | female | 1526.61(907.40,2320.24) | 1659.64(998.30,2621.84) | 8.71 | 545.13(516.45,575.02) | 405.37(384.75,426.87) | -0.97(-1.12,-0.82) |
| Kyrgyzstan | female | 8251.57(5301.17,12386.33) | 8275.58(5016.25,12713.41) | 0.29 | 949.02(927.30,971.14) | 710.29(694.00,726.88) | -1.04(-1.12,-0.97) |
| Lao People's Democratic Republic | female | 48028.19(29985.33,82147.68) | 9514.10(6401.11,13653.86) | -80.19 | 4665.77(4620.78,4711.12) | 811.94(794.82,829.35) | -5.86(-6.03,-5.68) |
| Latvia | female | 1350.83(883.11,2060.92) | 438.89(261.13,697.34) | -67.51 | 468.87(442.64,496.32) | 301.11(271.88,332.86) | -1.65(-1.74,-1.57) |
| Lebanon | female | 3982.26(2495.44,6068.44) | 3065.92(1806.07,4870.68) | -23.01 | 760.63(735.58,786.35) | 503.30(484.40,522.79) | -1.35(-1.42,-1.28) |
| Lesotho | female | 8900.35(6625.38,11896.74) | 5447.01(3825.51,7662.91) | -38.80 | 2223.36(2170.74,2276.98) | 1561.67(1515.16,1609.33) | -0.84(-1.03,-0.65) |
| Liberia | female | 71464.53(48749.35,104832.65) | 21020.51(14259.18,29422.32) | -70.59 | 8475.71(8398.76,8553.24) | 1763.98(1737.85,1790.42) | -5.79(-6.34,-5.24) |
| Libya | female | 6079.92(4059.42,9090.10) | 3742.76(2259.68,5926.39) | -38.44 | 652.35(634.93,670.14) | 532.15(513.77,551.07) | -0.46(-0.64,-0.28) |
| Lithuania | female | 1874.96(1253.38,2796.21) | 592.69(360.51,891.30) | -68.39 | 447.53(426.29,469.61) | 294.29(269.67,320.70) | -1.47(-1.54,-1.39) |
| Luxembourg | female | 74.29(41.89,118.51) | 57.73(33.00,98.05) | -22.29 | 236.53(182.38,302.52) | 123.81(92.26,163.26) | -2.32(-2.54,-2.10) |
| Madagascar | female | 429982.35(342739.69,521502.93) | 167144.77(114506.92,235657.66) | -61.13 | 12537.31(12493.25,12581.49) | 2697.07(2682.80,2711.40) | -4.51(-4.72,-4.29) |
| Malawi | female | 273693.05(189980.29,390470.87) | 93120.38(59663.99,142793.32) | -65.98 | 8778.90(8739.81,8818.13) | 2102.38(2087.35,2117.50) | -5.11(-5.44,-4.77) |
| Malaysia | female | 28212.80(19300.63,41163.38) | 18131.84(11897.81,26377.18) | -35.73 | 844.84(834.20,855.58) | 489.48(481.74,497.32) | -1.86(-1.92,-1.81) |
| Maldives | female | 2539.08(1678.32,3798.92) | 341.20(200.40,576.02) | -86.56 | 4275.58(4094.82,4462.54) | 700.07(623.41,784.23) | -5.73(-6.38,-5.07) |
| Mali | female | 305442.70(201825.18,462278.13) | 360013.63(249209.29,481361.29) | 17.87 | 11831.93(11782.38,11881.65) | 5129.59(5110.49,5148.76) | -2.83(-3.18,-2.49) |
| Malta | female | 121.29(69.43,204.00) | 51.69(29.56,88.32) | -57.39 | 302.61(247.74,366.60) | 174.24(127.68,232.98) | -1.95(-2.04,-1.86) |
| Marshall Islands | female | 115.24(77.08,165.28) | 70.79(48.53,100.42) | -38.57 | 878.62(699.07,1092.75) | 712.11(531.46,938.39) | -0.80(-1.00,-0.60) |
| Mauritania | female | 24093.12(15953.92,37387.77) | 12285.13(7022.40,18654.73) | -49.01 | 4300.88(4238.19,4364.29) | 1291.80(1267.45,1316.53) | -3.78(-3.97,-3.59) |
| Mauritius | female | 1503.06(1005.04,2334.95) | 499.60(316.61,740.34) | -66.76 | 902.78(852.73,955.19) | 487.79(441.59,537.78) | -1.84(-1.96,-1.71) |
| Mexico | female | 336852.95(302218.68,378146.73) | 53527.15(41205.26,69065.53) | -84.11 | 1593.51(1587.24,1599.80) | 311.35(308.36,314.36) | -5.15(-5.36,-4.93) |
| Micronesia (Federated States of) | female | 305.64(208.41,422.43) | 103.58(67.51,148.23) | -66.11 | 1036.75(898.50,1191.36) | 642.96(509.48,803.07) | -1.62(-1.68,-1.57) |
| Monaco | female | 1.87(1.05,3.16) | 2.14(1.04,3.74) | 14.29 | 115.25(9.82,510.40) | 89.06(10.30,354.21) | -0.92(-1.00,-0.85) |
| Mongolia | female | 5775.89(3706.83,8570.13) | 4675.00(2868.12,7249.04) | -19.06 | 1267.65(1233.29,1302.75) | 865.79(839.70,892.52) | -1.46(-1.55,-1.38) |
| Montenegro | female | 257.77(153.58,402.73) | 132.40(76.10,213.92) | -48.63 | 322.95(282.20,368.33) | 242.25(200.11,291.30) | -1.28(-1.46,-1.09) |
| Morocco | female | 59338.99(42079.91,82548.53) | 27898.18(16897.33,42143.90) | -52.99 | 1118.68(1108.93,1128.50) | 575.04(567.83,582.32) | -2.18(-2.27,-2.09) |
| Mozambique | female | 376260.82(234528.09,561918.32) | 171597.09(117654.79,227390.54) | -54.39 | 9970.98(9933.61,10008.47) | 2187.51(2176.18,2198.89) | -5.13(-5.37,-4.89) |
| Myanmar | female | 135391.32(95429.08,191121.17) | 66174.09(40727.96,104731.64) | -51.12 | 1757.95(1747.70,1768.25) | 829.52(822.76,836.34) | -2.70(-2.81,-2.58) |
| Namibia | female | 10216.91(7237.80,13891.10) | 6313.02(4345.32,8987.99) | -38.21 | 2694.75(2632.77,2757.88) | 1259.63(1223.02,1297.13) | -2.01(-2.28,-1.74) |
| Nauru | female | 28.20(19.61,39.53) | 17.93(12.25,25.79) | -36.42 | 837.64(476.88,1375.63) | 677.85(342.74,1219.70) | -0.77(-1.25,-0.29) |
| Nepal | female | 389450.61(275860.85,550900.88) | 56250.25(38182.83,81427.07) | -85.56 | 7206.76(7180.39,7233.20) | 1198.74(1188.12,1209.44) | -5.77(-6.12,-5.43) |
| Netherlands | female | 3266.42(1848.18,5351.53) | 2136.53(1133.09,3407.92) | -34.59 | 253.40(244.22,262.85) | 172.04(164.41,179.95) | -1.32(-1.44,-1.20) |
| New Zealand | female | 255.03(120.46,505.92) | 234.72(105.77,478.45) | -7.96 | 57.82(50.29,66.25) | 47.12(40.86,54.15) | -0.61(-0.67,-0.55) |
| Nicaragua | female | 13768.42(10869.70,17563.75) | 4195.84(2976.78,5713.44) | -69.53 | 1302.12(1277.88,1326.73) | 392.56(379.33,406.15) | -4.44(-4.76,-4.12) |
| Niger | female | 301395.20(183231.27,457985.67) | 154168.79(109886.73,213548.83) | -48.85 | 10705.16(10658.19,10752.29) | 2070.68(2059.05,2082.37) | -5.96(-6.34,-5.58) |
| Nigeria | female | 715410.34(521610.00,1001458.96) | 873394.40(599284.79,1267617.19) | 22.08 | 2757.44(2749.89,2765.01) | 1593.80(1590.18,1597.42) | -1.82(-2.03,-1.60) |
| Niue | female | 2.92(2.03,4.06) | 1.99(1.50,2.68) | -31.83 | 695.63(101.39,2485.97) | 913.68(53.71,4481.07) | -0.44(-0.92,0.05) |
| North Macedonia | female | 1252.64(709.01,1936.76) | 471.35(281.70,747.30) | -62.37 | 479.40(451.36,508.85) | 294.45(266.60,324.65) | -1.78(-1.87,-1.68) |
| Northern Mariana Islands | female | 34.81(23.85,49.86) | 22.55(14.51,33.43) | -35.21 | 460.06(300.09,680.35) | 403.59(236.91,651.49) | -0.17(-0.30,-0.04) |
| Norway | female | 781.62(428.02,1294.18) | 527.32(288.08,850.65) | -32.54 | 209.00(193.57,225.38) | 125.66(114.51,137.66) | -1.76(-2.00,-1.51) |
| Oman | female | 6070.01(4338.52,8603.78) | 2767.25(1739.49,4246.94) | -54.41 | 1243.05(1208.75,1278.12) | 442.84(425.21,461.05) | -2.79(-3.23,-2.34) |
| Pakistan | female | 878610.85(660521.24,1121103.42) | 831316.98(604617.98,1128333.11) | -5.38 | 3152.70(3145.34,3160.07) | 1931.93(1927.49,1936.38) | -1.30(-1.42,-1.18) |
| Palau | female | 16.98(11.45,24.64) | 7.96(5.24,11.72) | -53.13 | 648.67(338.68,1145.82) | 484.33(179.41,1087.54) | -0.84(-0.90,-0.79) |
| Palestine | female | 3155.12(2060.50,4550.70) | 3189.11(1946.69,5007.68) | 1.08 | 594.21(571.72,617.39) | 339.83(327.31,352.74) | -1.89(-1.98,-1.81) |
| Panama | female | 3970.27(3290.50,4765.35) | 2745.91(2104.80,3629.77) | -30.84 | 761.44(733.40,790.35) | 353.74(337.18,370.95) | -3.13(-3.53,-2.72) |
| Papua New Guinea | female | 8119.31(5700.62,12762.03) | 16813.55(11150.86,25931.23) | 107.08 | 731.17(712.09,750.66) | 712.15(699.66,724.82) | 0.32(0.05,0.59) |
| Paraguay | female | 10124.47(7681.12,13126.15) | 5488.31(3721.37,7976.99) | -45.79 | 938.02(916.53,959.91) | 573.52(557.58,589.83) | -1.75(-2.08,-1.41) |
| Peru | female | 119951.96(89238.46,154064.00) | 24596.70(17311.48,33824.49) | -79.49 | 2205.82(2190.71,2221.01) | 460.45(453.99,467.00) | -5.37(-5.70,-5.04) |
| Philippines | female | 214584.18(175937.15,270585.55) | 119121.39(83085.74,169890.67) | -44.49 | 1578.03(1570.57,1585.52) | 720.48(716.04,724.95) | -2.20(-2.36,-2.04) |
| Poland | female | 18785.79(10672.54,31834.03) | 6784.16(4185.95,10459.48) | -63.89 | 401.03(394.91,407.22) | 234.13(228.22,240.16) | -1.86(-1.92,-1.79) |
| Portugal | female | 2940.43(1651.06,4802.72) | 1099.96(600.81,1943.04) | -62.59 | 325.66(312.97,338.78) | 179.70(168.58,191.41) | -2.13(-2.31,-1.94) |
| Puerto Rico | female | 2786.21(1771.31,4055.17) | 822.08(501.23,1283.10) | -70.49 | 563.84(541.55,586.88) | 394.30(364.79,425.84) | -1.21(-1.38,-1.04) |
| Qatar | female | 375.60(230.67,571.67) | 871.51(534.88,1357.22) | 132.03 | 573.61(512.54,640.14) | 341.12(317.18,366.47) | -1.99(-2.12,-1.86) |
| Republic of Korea | female | 4732.36(2780.06,7644.91) | 1297.97(715.51,2179.99) | -72.57 | 85.14(82.55,87.79) | 44.02(41.46,46.71) | -2.08(-2.34,-1.82) |
| Republic of Moldova | female | 2988.26(1906.48,4513.01) | 828.11(489.93,1219.66) | -72.29 | 473.16(455.20,491.68) | 320.22(297.30,344.61) | -1.32(-1.40,-1.25) |
| Romania | female | 12137.74(7578.74,19406.89) | 3987.19(2438.93,6341.09) | -67.15 | 445.28(436.82,453.88) | 267.94(259.05,277.07) | -1.80(-1.88,-1.72) |
| Russian Federation | female | 78279.08(47377.91,121512.66) | 39488.10(24075.94,60962.11) | -49.55 | 453.54(450.17,456.92) | 309.32(306.06,312.61) | -1.58(-1.77,-1.40) |
| Rwanda | female | 147627.10(93533.86,212036.79) | 32286.22(23357.36,42614.13) | -78.13 | 6815.72(6775.44,6856.19) | 1176.43(1162.07,1190.92) | -7.18(-7.86,-6.50) |
| Saint Kitts and Nevis | female | 155.85(128.14,190.50) | 48.10(35.53,64.48) | -69.14 | 1898.14(1568.98,2280.25) | 866.25(608.13,1205.49) | -2.30(-2.77,-1.83) |
| Saint Lucia | female | 352.19(258.62,471.28) | 114.35(75.29,172.88) | -67.53 | 1280.36(1137.94,1436.70) | 766.95(621.18,939.69) | -1.50(-1.76,-1.23) |
| Saint Vincent and the Grenadines | female | 361.80(280.55,469.11) | 119.64(82.55,168.76) | -66.93 | 1708.82(1520.13,1916.17) | 958.36(779.85,1169.04) | -1.74(-2.13,-1.35) |
| Samoa | female | 347.15(244.71,474.74) | 258.34(169.00,400.73) | -25.58 | 797.71(700.98,904.69) | 568.58(491.95,654.30) | -1.04(-1.08,-1.00) |
| San Marino | female | 3.42(1.85,5.75) | 2.70(1.48,4.94) | -21.13 | 191.74(38.50,599.12) | 143.44(21.74,516.34) | -0.95(-1.15,-0.74) |
| Sao Tome and Principe | female | 1896.57(1330.67,2454.64) | 528.37(345.36,798.28) | -72.14 | 5640.34(5348.97,5944.20) | 1346.95(1226.86,1476.53) | -4.78(-5.30,-4.26) |
| Saudi Arabia | female | 37560.81(26592.51,52431.60) | 15615.42(9625.03,24487.86) | -58.43 | 1080.86(1069.08,1092.75) | 432.47(425.24,439.79) | -2.86(-2.96,-2.75) |
| Senegal | female | 100826.54(73720.98,134741.73) | 56495.28(34579.16,85491.35) | -43.97 | 4692.08(4659.19,4725.15) | 1746.68(1731.27,1762.20) | -3.47(-3.63,-3.32) |
| Serbia | female | 4599.47(2747.68,7286.96) | 1571.07(953.18,2460.98) | -65.84 | 403.08(390.70,415.78) | 252.28(238.92,266.26) | -1.75(-1.85,-1.65) |
| Seychelles | female | 71.68(46.17,108.05) | 53.72(34.51,80.44) | -25.06 | 597.28(457.27,769.22) | 454.18(332.03,609.24) | -0.83(-0.88,-0.79) |
| Sierra Leone | female | 122623.22(79974.55,177635.12) | 166049.65(111904.99,243706.02) | 35.41 | 9089.21(9027.40,9151.36) | 6460.10(6421.77,6498.62) | -0.04(-0.62,0.54) |
| Singapore | female | 251.74(129.48,467.24) | 155.87(80.33,268.88) | -38.08 | 78.03(67.78,89.56) | 37.64(31.63,44.51) | -2.18(-2.59,-1.78) |
| Slovakia | female | 2332.46(1403.85,3756.00) | 986.59(601.18,1532.17) | -57.70 | 357.29(341.88,373.26) | 230.82(215.68,246.81) | -1.57(-1.62,-1.53) |
| Slovenia | female | 622.61(349.92,1022.01) | 297.35(182.42,470.41) | -52.24 | 310.97(285.27,338.54) | 193.82(170.95,219.13) | -1.78(-1.88,-1.68) |
| Solomon Islands | female | 1117.61(767.89,1581.85) | 1129.93(804.23,1560.91) | 1.10 | 975.21(904.22,1050.61) | 741.25(691.55,793.73) | -0.71(-0.81,-0.62) |
| Somalia | female | 256032.98(161166.51,399820.83) | 198094.38(130854.31,287640.53) | -22.63 | 12045.81(11993.37,12098.43) | 3469.54(3452.96,3486.18) | -2.78(-5.34,-0.16) |
| South Africa | female | 278816.34(229778.24,340939.60) | 160158.54(120763.92,203800.93) | -42.56 | 3173.82(3159.47,3188.24) | 1979.41(1968.69,1990.17) | -0.96(-1.19,-0.73) |
| South Sudan | female | 214053.61(140725.37,326511.90) | 132283.22(87845.81,188148.83) | -38.20 | 13746.01(13677.87,13814.41) | 5597.54(5563.24,5632.02) | -2.98(-3.62,-2.35) |
| Spain | female | 8529.51(4534.31,14178.51) | 4374.77(2266.34,7269.76) | -48.71 | 260.53(254.55,266.62) | 154.59(149.72,159.59) | -1.87(-2.04,-1.70) |
| Sri Lanka | female | 26010.44(17803.57,36592.23) | 13681.98(8863.31,20603.54) | -47.40 | 951.61(938.97,964.39) | 560.27(550.01,570.68) | -1.81(-1.90,-1.73) |
| Sudan | female | 253083.83(153586.00,418252.45) | 93537.98(61577.70,133620.69) | -63.04 | 4706.06(4684.91,4727.28) | 1109.65(1101.96,1117.39) | -4.60(-4.71,-4.49) |
| Suriname | female | 1127.34(805.46,1541.27) | 612.95(401.08,903.32) | -45.63 | 1671.10(1568.38,1779.24) | 867.08(794.64,944.85) | -2.14(-2.36,-1.93) |
| Sweden | female | 1582.51(901.07,2674.71) | 1216.28(620.15,2054.30) | -23.14 | 214.00(202.86,225.60) | 145.65(137.13,154.60) | -1.34(-1.48,-1.20) |
| Switzerland | female | 1079.52(586.27,1764.20) | 826.20(420.23,1476.34) | -23.47 | 197.68(185.25,210.76) | 133.52(124.05,143.54) | -1.46(-1.63,-1.29) |
| Syrian Arab Republic | female | 39916.22(28712.24,53396.93) | 11788.70(7306.32,17433.53) | -70.47 | 1313.30(1299.55,1327.16) | 680.25(666.60,694.13) | -1.82(-2.12,-1.52) |
| Taiwan (Province of China) | female | 7673.85(5294.52,11391.71) | 2199.04(1384.66,3401.08) | -71.34 | 287.60(280.47,294.88) | 156.11(149.02,163.47) | -2.00(-2.32,-1.67) |
| Tajikistan | female | 16384.54(11220.49,22516.66) | 16943.90(10887.96,24294.21) | 3.41 | 1295.07(1273.57,1316.84) | 938.93(923.87,954.18) | -1.40(-1.65,-1.16) |
| Thailand | female | 58935.51(42191.96,83321.43) | 18288.24(11787.10,27623.45) | -68.97 | 702.70(696.48,708.96) | 393.85(387.57,400.21) | -1.81(-1.95,-1.66) |
| Timor-Leste | female | 15010.84(8595.24,29843.74) | 2998.88(2134.48,4256.79) | -80.02 | 7846.16(7709.83,7984.51) | 1095.25(1052.38,1139.50) | -6.92(-7.39,-6.44) |
| Togo | female | 34838.61(24823.92,48063.40) | 36506.60(22930.00,54030.21) | 4.79 | 3493.96(3452.95,3535.36) | 2169.98(2146.54,2193.62) | -1.66(-1.96,-1.35) |
| Tokelau | female | 3.98(2.69,5.73) | 2.02(1.42,2.76) | -49.34 | 816.40(93.00,3203.05) | 1004.74(51.27,5122.57) | -0.80(-1.42,-0.17) |
| Tonga | female | 227.48(157.11,308.94) | 145.57(102.74,199.56) | -36.01 | 783.65(661.06,923.59) | 599.73(488.19,730.15) | -0.81(-0.93,-0.69) |
| Trinidad and Tobago | female | 2238.33(1555.89,3250.18) | 890.29(558.64,1362.72) | -60.23 | 1094.73(1046.62,1144.60) | 669.31(622.68,718.79) | -1.84(-1.96,-1.73) |
| Tunisia | female | 12348.12(8180.39,17503.46) | 6230.97(3722.64,9832.54) | -49.54 | 803.43(788.41,818.68) | 468.79(456.43,481.41) | -1.71(-1.84,-1.58) |
| Türkiye | female | 126734.54(86551.81,176448.54) | 40086.09(24653.06,60718.76) | -68.37 | 1197.98(1190.87,1205.12) | 444.77(440.07,449.51) | -3.19(-3.43,-2.94) |
| Turkmenistan | female | 9886.41(6668.15,13621.42) | 6225.89(3770.01,9636.44) | -37.03 | 1215.61(1189.65,1242.00) | 820.43(798.89,842.44) | -1.41(-1.47,-1.36) |
| Tuvalu | female | 41.94(28.23,60.02) | 13.06(8.88,19.11) | -68.87 | 1329.66(829.55,2039.96) | 612.61(286.71,1165.82) | -2.30(-2.47,-2.14) |
| Uganda | female | 281067.51(182580.21,410104.95) | 153952.53(96766.92,225102.78) | -45.23 | 4659.33(4639.06,4679.67) | 1303.24(1295.78,1310.73) | -4.03(-4.25,-3.81) |
| Ukraine | female | 34044.63(23786.34,48269.37) | 12668.61(7804.70,20251.25) | -62.79 | 593.74(586.94,600.60) | 421.84(413.72,430.08) | -1.33(-1.53,-1.14) |
| United Arab Emirates | female | 2285.69(1545.84,3296.25) | 3030.77(1870.81,4772.67) | 32.60 | 739.83(707.22,773.62) | 457.88(440.63,475.67) | -1.53(-1.63,-1.43) |
| United Kingdom | female | 16950.44(10801.72,25417.13) | 13508.85(8505.80,21254.09) | -20.30 | 316.61(311.47,321.81) | 248.21(243.71,252.78) | -0.84(-0.92,-0.77) |
| United Republic of Tanzania | female | 591116.90(428146.28,789629.56) | 250597.10(177403.41,364747.80) | -57.61 | 7739.40(7717.09,7761.76) | 1749.24(1741.47,1757.05) | -5.01(-5.19,-4.83) |
| United States of America | female | 12120.84(7235.99,20345.95) | 14124.76(8706.17,21540.34) | 16.53 | 42.24(41.44,43.04) | 47.10(46.29,47.92) | 0.40(0.26,0.55) |
| United States Virgin Islands | female | 114.73(78.34,168.43) | 31.41(18.82,51.07) | -72.63 | 702.77(571.69,856.92) | 488.75(321.05,721.10) | -1.13(-1.34,-0.93) |
| Uruguay | female | 1791.07(1418.39,2427.14) | 380.49(223.89,683.66) | -78.76 | 397.20(377.08,418.19) | 112.71(100.35,126.28) | -4.11(-4.39,-3.84) |
| Uzbekistan | female | 65965.63(43430.10,95615.52) | 53457.96(31389.19,83813.35) | -18.96 | 1464.40(1452.45,1476.43) | 1082.11(1072.47,1091.82) | -1.22(-1.30,-1.14) |
| Vanuatu | female | 423.16(285.32,594.81) | 515.86(354.29,711.27) | 21.91 | 858.44(759.09,967.81) | 746.42(673.02,825.99) | -0.66(-0.90,-0.41) |
| Venezuela (Bolivarian Republic of) | female | 40733.98(35768.26,47524.14) | 15542.97(11540.29,20682.64) | -61.84 | 875.28(865.22,885.43) | 393.29(386.05,400.64) | -3.01(-3.62,-2.40) |
| Viet Nam | female | 106081.54(70534.50,151929.88) | 51478.30(32822.57,79217.33) | -51.47 | 799.68(794.48,804.90) | 430.64(426.62,434.68) | -2.13(-2.18,-2.09) |
| Yemen | female | 220386.13(158105.02,307606.78) | 217687.50(156442.61,284667.86) | -1.22 | 5367.48(5342.75,5392.31) | 3160.33(3146.14,3174.57) | -1.87(-1.96,-1.79) |
| Zambia | female | 192880.79(136409.80,264770.10) | 83399.65(57395.97,120642.10) | -56.76 | 7003.37(6964.53,7042.38) | 1799.86(1786.30,1813.51) | -4.73(-5.11,-4.35) |
| Zimbabwe | female | 69943.59(52116.06,89726.98) | 118238.43(86015.33,156776.37) | 69.05 | 2461.40(2440.75,2482.20) | 2799.96(2780.27,2819.77) | 1.05(0.76,1.34) |
| Global | male | 27470461.94(22961074.21,33395407.67) | 10888168.23(8381472.38,14146947.35) | -60.36 | 2756.79(2755.64,2757.93) | 1012.67(1012.01,1013.34) | -3.41(-3.74,-3.08) |
| Central Europe, eastern Europe, and central Asia | male | 375835.14(265071.58,526550.92) | 162113.05(109051.16,239838.86) | -56.87 | 684.26(681.90,686.62) | 391.24(389.18,393.30) | -2.35(-2.58,-2.12) |
| High-income | male | 116832.68(87762.41,159856.30) | 44919.92(28855.57,71450.30) | -61.55 | 108.55(107.86,109.25) | 48.45(47.97,48.94) | -2.01(-2.22,-1.80) |
| Latin America and Caribbean | male | 1709022.87(1525807.83,1956095.86) | 380672.33(290208.56,499799.55) | -77.73 | 2000.28(1996.89,2003.67) | 471.31(469.63,473.00) | -4.80(-4.97,-4.62) |
| Southeast Asia, east Asia, and Oceania | male | 3675455.87(3008813.23,4525847.94) | 724281.27(526785.38,973011.16) | -80.29 | 1293.45(1291.99,1294.90) | 314.06(313.27,314.86) | -6.16(-7.32,-4.99) |
| Sub-Saharan Africa | male | 9394903.58(7531755.18,12255289.99) | 5239951.31(4085957.33,6707879.66) | -44.23 | 6553.71(6548.84,6558.58) | 1921.49(1919.64,1923.33) | -3.95(-4.42,-3.49) |
| Andean Latin America | male | 262325.55(211598.49,320165.65) | 55850.61(42126.23,76248.81) | -78.71 | 2593.44(2581.51,2605.42) | 525.10(520.18,530.06) | -5.41(-5.65,-5.18) |
| Australasia | male | 2000.75(869.85,5130.97) | 1306.06(467.01,3453.16) | -34.72 | 79.27(75.48,83.21) | 42.81(40.28,45.47) | -2.06(-2.24,-1.87) |
| Caribbean | male | 188580.42(147476.10,234345.41) | 84410.09(60820.32,116547.73) | -55.24 | 2762.20(2748.26,2776.20) | 1320.61(1310.79,1330.49) | -2.28(-2.56,-2.00) |
| Central Asia | male | 183276.40(129900.07,261641.67) | 110061.73(72442.14,160509.85) | -39.95 | 1339.47(1332.85,1346.11) | 739.30(734.61,744.01) | -2.35(-2.53,-2.16) |
| Central Europe | male | 88633.88(58357.97,126985.97) | 21862.73(13791.53,33522.54) | -75.33 | 606.82(602.53,611.14) | 243.46(240.00,246.95) | -3.15(-3.26,-3.03) |
| Central Latin America | male | 680109.85(611744.45,771699.37) | 138740.44(108499.58,178863.44) | -79.60 | 1694.05(1689.44,1698.67) | 377.51(375.22,379.80) | -4.70(-4.84,-4.56) |
| Central Sub-Saharan Africa | male | 1153041.96(844168.40,1645172.21) | 490245.23(342848.46,688900.79) | -57.48 | 7173.10(7158.18,7188.05) | 1511.42(1506.77,1516.09) | -5.09(-5.55,-4.62) |
| East Asia | male | 1854157.61(1496387.48,2276020.19) | 166408.18(118086.85,222655.75) | -91.03 | 979.39(977.85,980.93) | 126.05(125.39,126.70) | -9.58(-11.63,-7.49) |
| Eastern Europe | male | 103924.85(70212.64,152951.90) | 30188.59(19269.38,46294.96) | -70.95 | 391.87(389.30,394.46) | 172.41(170.28,174.56) | -3.19(-3.54,-2.83) |
| Eastern Sub-Saharan Africa | male | 5130048.25(4028116.99,6729123.48) | 2077347.34(1649395.40,2615092.78) | -59.51 | 8904.08(8895.18,8912.99) | 2020.07(2016.96,2023.17) | -4.74(-5.54,-3.94) |
| High-income Asia Pacific | male | 15166.85(8337.44,26391.98) | 3960.59(2039.85,7398.19) | -73.89 | 85.13(83.66,86.63) | 36.08(34.86,37.33) | -2.59(-2.87,-2.31) |
| High-income North America | male | 16780.82(9858.70,27390.83) | 19432.99(12326.28,29230.47) | 15.80 | 48.88(48.09,49.69) | 56.22(55.39,57.05) | 1.25(0.73,1.76) |
| North Africa and Middle East | male | 1171708.19(884590.54,1554720.86) | 593475.59(427526.18,828143.93) | -49.35 | 1487.73(1484.79,1490.67) | 609.14(607.45,610.83) | -2.94(-3.06,-2.82) |
| Oceania | male | 18365.96(13369.60,24302.98) | 28400.19(18714.46,44137.30) | 54.63 | 1032.85(1015.44,1050.50) | 883.63(871.88,895.51) | -0.21(-0.35,-0.06) |
| South Asia | male | 11026703.61(9039557.88,13731527.33) | 3742754.75(2647334.94,5198330.49) | -66.06 | 4484.76(4481.89,4487.64) | 1426.08(1424.51,1427.65) | -3.61(-3.65,-3.56) |
| Southeast Asia | male | 1802932.31(1417346.65,2289359.56) | 529472.91(383859.99,707575.38) | -70.63 | 1933.38(1930.27,1936.50) | 575.08(573.38,576.79) | -3.92(-4.05,-3.78) |
| Southern Latin America | male | 61032.35(49833.94,79781.96) | 9916.66(5173.50,22591.14) | -83.75 | 648.52(642.59,654.49) | 131.88(128.95,134.85) | -4.99(-5.29,-4.68) |
| Southern Sub-Saharan Africa | male | 399538.05(328643.53,507584.44) | 281576.77(220674.71,350358.59) | -29.52 | 2828.15(2817.42,2838.91) | 2018.00(2009.42,2026.61) | -0.78(-0.96,-0.60) |
| Tropical Latin America | male | 578007.05(492541.30,692570.94) | 101671.19(67425.58,153824.82) | -82.41 | 2086.66(2080.77,2092.56) | 371.14(368.63,373.66) | -5.93(-6.18,-5.67) |
| Western Europe | male | 21851.91(13424.65,33878.77) | 10303.62(5621.61,19185.14) | -52.85 | 57.83(57.03,58.63) | 29.06(28.48,29.66) | -2.10(-2.40,-1.80) |
| Western Sub-Saharan Africa | male | 2712275.32(2156392.02,3523322.36) | 2390781.96(1817066.05,3100845.89) | -11.85 | 4762.69(4756.03,4769.36) | 1946.57(1943.83,1949.32) | -2.88(-2.99,-2.77) |
| Afghanistan | male | 52453.49(35998.24,80512.42) | 56543.32(38213.41,79999.14) | 7.80 | 1932.77(1914.57,1951.12) | 678.21(672.18,684.29) | -3.70(-4.16,-3.24) |
| Albania | male | 6823.49(4382.36,10765.84) | 700.65(446.84,1079.18) | -89.73 | 1107.28(1078.97,1136.17) | 292.43(269.31,317.13) | -4.94(-5.19,-4.69) |
| Algeria | male | 65104.13(43756.07,93203.84) | 28921.09(17138.65,46242.37) | -55.58 | 1157.48(1147.94,1167.07) | 401.76(396.74,406.82) | -3.71(-3.83,-3.58) |
| American Samoa | male | 99.05(70.78,139.00) | 37.54(25.52,54.27) | -62.11 | 682.68(527.05,872.28) | 504.01(332.10,739.64) | -0.93(-0.98,-0.88) |
| Andorra | male | 1.62(0.70,3.28) | 0.86(0.37,1.68) | -46.84 | 31.78(2.40,167.69) | 15.68(0.17,149.43) | -2.28(-2.59,-1.96) |
| Angola | male | 458844.43(311894.22,687376.60) | 134501.71(91844.13,186811.05) | -70.69 | 15639.27(15587.29,15691.38) | 1555.00(1545.78,1564.26) | -7.67(-7.95,-7.38) |
| Antigua and Barbuda | male | 76.53(52.43,132.77) | 38.59(25.85,66.29) | -49.57 | 722.44(548.61,937.21) | 395.67(263.99,574.68) | -1.97(-2.29,-1.66) |
| Argentina | male | 49550.42(39594.92,66917.22) | 7994.45(3621.44,19958.94) | -83.87 | 788.06(780.04,796.15) | 153.47(149.70,157.33) | -5.13(-5.52,-4.74) |
| Armenia | male | 6787.19(4808.53,9526.41) | 1682.03(990.57,2815.45) | -75.22 | 1204.26(1173.87,1235.27) | 539.46(511.89,568.22) | -2.92(-3.05,-2.80) |
| Australia | male | 1395.65(506.83,4099.99) | 867.04(253.79,2779.18) | -37.88 | 69.23(65.30,73.35) | 34.77(32.27,37.44) | -2.36(-2.58,-2.14) |
| Austria | male | 344.41(154.32,728.16) | 129.62(56.95,258.46) | -62.36 | 48.56(43.48,54.14) | 19.10(15.91,22.82) | -2.95(-3.26,-2.64) |
| Azerbaijan | male | 19910.23(14010.95,27600.35) | 8051.13(4658.73,12498.11) | -59.56 | 1499.63(1477.36,1522.16) | 669.77(654.16,685.67) | -3.06(-3.30,-2.83) |
| Bahamas | male | 319.63(226.69,465.22) | 151.60(94.51,245.29) | -52.57 | 731.02(644.40,826.92) | 385.22(318.95,462.33) | -2.13(-2.50,-1.77) |
| Bahrain | male | 555.24(346.70,824.92) | 264.61(144.88,485.89) | -52.34 | 616.34(561.76,675.03) | 175.54(153.13,200.47) | -4.22(-4.35,-4.09) |
| Bangladesh | male | 2386101.33(1876130.94,2912480.26) | 164550.28(115206.87,242763.36) | -93.10 | 8213.13(8201.83,8224.44) | 688.10(684.43,691.78) | -8.14(-8.36,-7.92) |
| Barbados | male | 200.80(141.29,299.03) | 69.87(42.55,110.09) | -65.20 | 610.21(520.09,712.62) | 294.56(223.09,383.38) | -2.37(-2.60,-2.14) |
| Belarus | male | 4304.57(2736.60,6470.45) | 1070.68(610.87,1833.58) | -75.13 | 344.27(333.23,355.59) | 135.12(126.45,144.27) | -3.63(-3.96,-3.29) |
| Belgium | male | 479.29(243.25,943.68) | 224.98(102.69,471.76) | -53.06 | 50.49(45.98,55.37) | 22.14(19.27,25.38) | -2.66(-2.86,-2.46) |
| Belize | male | 1082.63(911.62,1278.01) | 465.66(339.63,644.99) | -56.99 | 2211.78(2067.04,2364.62) | 675.33(606.77,750.04) | -3.87(-4.37,-3.37) |
| Benin | male | 82338.02(47778.61,129154.00) | 46245.42(29349.99,70152.52) | -43.83 | 5248.74(5207.64,5290.11) | 1291.65(1278.55,1304.85) | -4.48(-4.62,-4.33) |
| Bermuda | male | 19.86(11.72,33.11) | 4.48(2.46,7.30) | -77.43 | 302.87(174.52,493.63) | 105.36(27.22,292.27) | -3.44(-3.80,-3.07) |
| Bhutan | male | 3762.99(2024.27,6138.88) | 1702.55(785.42,2822.99) | -54.76 | 2533.80(2446.52,2623.59) | 1771.61(1682.58,1864.45) | -1.66(-1.84,-1.48) |
| Bolivia (Plurinational State of) | male | 98678.57(63755.10,135764.80) | 23977.35(17056.81,35043.13) | -75.70 | 5082.91(5044.50,5121.55) | 1166.93(1150.12,1183.93) | -4.79(-4.95,-4.62) |
| Bosnia and Herzegovina | male | 3556.81(2077.79,5838.62) | 705.35(396.96,1186.46) | -80.17 | 647.22(624.69,670.41) | 286.29(264.04,310.02) | -3.23(-3.57,-2.89) |
| Botswana | male | 8363.43(5945.59,11028.42) | 6550.79(4405.48,9530.71) | -21.67 | 2189.56(2133.43,2246.86) | 1518.85(1474.93,1563.80) | -1.16(-1.31,-1.00) |
| Brazil | male | 569816.37(485145.04,681991.45) | 96265.76(63445.21,147101.96) | -83.11 | 2134.46(2128.39,2140.54) | 365.03(362.49,367.58) | -6.06(-6.32,-5.80) |
| Brunei Darussalam | male | 75.90(39.64,140.58) | 40.45(19.35,74.29) | -46.70 | 137.51(104.86,177.46) | 77.75(53.59,109.81) | -1.71(-2.01,-1.41) |
| Bulgaria | male | 4728.70(2785.70,7868.38) | 1630.16(928.97,2647.10) | -65.53 | 542.58(525.97,559.61) | 329.19(312.11,347.02) | -1.74(-1.92,-1.56) |
| Burkina Faso | male | 220244.00(154091.56,308907.40) | 193159.41(133653.19,266131.43) | -12.30 | 6906.73(6872.44,6941.14) | 3026.78(3011.52,3042.11) | -2.60(-2.94,-2.26) |
| Burundi | male | 208324.95(113465.60,357173.29) | 73206.18(40548.27,114550.06) | -64.86 | 12542.67(12480.26,12605.33) | 2219.82(2202.01,2237.74) | -5.72(-5.92,-5.51) |
| Cabo Verde | male | 2919.95(2045.13,3902.50) | 503.09(306.68,911.85) | -82.77 | 2852.92(2735.72,2974.14) | 674.57(611.26,743.10) | -5.00(-5.36,-4.63) |
| Cambodia | male | 160675.10(102410.98,250988.00) | 25341.25(16449.66,41305.96) | -84.23 | 5622.89(5592.57,5653.34) | 896.38(884.29,908.60) | -6.66(-7.01,-6.30) |
| Cameroon | male | 68607.33(47769.63,94572.93) | 72806.70(45643.61,112033.53) | 6.12 | 2167.75(2148.99,2186.64) | 936.85(929.27,944.48) | -2.86(-3.06,-2.66) |
| Canada | male | 939.47(517.89,1610.12) | 1540.18(509.25,2502.32) | 63.94 | 31.35(29.23,33.59) | 47.06(44.66,49.58) | 2.39(1.70,3.09) |
| Central African Republic | male | 41210.07(27288.15,64511.00) | 50356.46(35663.43,71367.70) | 22.19 | 5353.04(5295.66,5410.91) | 3854.49(3817.21,3892.06) | -0.88(-1.28,-0.47) |
| Chad | male | 155320.05(103341.58,224074.52) | 150686.52(103853.54,212336.15) | -2.98 | 8201.75(8154.58,8249.15) | 2737.38(2721.83,2753.00) | -3.58(-3.70,-3.46) |
| Chile | male | 9014.47(7438.49,11459.74) | 1484.30(1019.72,2243.31) | -83.53 | 349.06(340.80,357.48) | 74.25(70.01,78.70) | -4.89(-5.14,-4.63) |
| China | male | 1808108.77(1455045.34,2214966.80) | 156999.00(112588.85,209669.54) | -91.32 | 988.82(987.25,990.40) | 122.68(122.02,123.33) | -7.46(-7.79,-7.12) |
| Colombia | male | 84876.53(69562.40,104697.52) | 26437.99(19578.89,36224.59) | -68.85 | 1166.57(1157.69,1175.50) | 377.00(371.50,382.57) | -3.38(-3.76,-3.00) |
| Comoros | male | 8055.46(5339.32,11265.58) | 1969.87(1293.30,2859.43) | -75.55 | 6059.54(5910.24,6211.87) | 1505.21(1432.34,1581.03) | -4.67(-4.85,-4.48) |
| Congo | male | 16103.56(11622.77,22102.02) | 12110.31(8315.86,17794.37) | -24.80 | 2734.91(2688.75,2781.70) | 1202.50(1179.15,1226.22) | -3.01(-3.47,-2.56) |
| Cook Islands | male | 16.57(9.54,29.07) | 3.72(2.01,6.36) | -77.54 | 461.79(251.15,785.99) | 197.66(41.59,603.41) | -2.65(-2.81,-2.50) |
| Costa Rica | male | 2043.01(1365.54,2936.07) | 1050.96(610.04,1679.66) | -48.56 | 327.48(312.23,343.31) | 197.67(185.04,211.00) | -0.88(-1.18,-0.57) |
| Croatia | male | 1782.52(1039.46,3011.32) | 506.32(286.92,838.08) | -71.60 | 362.81(344.91,381.45) | 168.61(153.16,185.32) | -2.74(-2.84,-2.64) |
| Cuba | male | 4995.79(3178.49,7817.93) | 1971.74(1153.17,3322.63) | -60.53 | 357.66(346.82,368.77) | 213.61(203.38,224.24) | -1.60(-1.82,-1.39) |
| Cyprus | male | 64.16(29.09,130.76) | 20.24(8.53,42.71) | -68.45 | 60.81(46.63,78.53) | 17.62(10.71,27.78) | -4.43(-4.79,-4.07) |
| Czechia | male | 4473.98(2605.58,7416.91) | 1582.72(906.70,2564.30) | -64.62 | 416.71(403.58,430.19) | 179.49(170.13,189.25) | -2.55(-2.80,-2.29) |
| C?te d'Ivoire | male | 109389.27(76220.10,151980.76) | 81715.58(52754.25,119945.27) | -25.30 | 2983.82(2963.65,3004.10) | 1194.88(1185.79,1204.02) | -2.96(-3.23,-2.70) |
| Democratic People's Republic of Korea | male | 39370.84(26310.06,55975.22) | 8158.10(4915.31,13249.44) | -79.28 | 1077.25(1065.29,1089.31) | 332.76(324.83,340.84) | -12.14(-18.04,-5.81) |
| Democratic Republic of the Congo | male | 626686.35(405202.26,955757.05) | 287197.76(179252.24,437737.14) | -54.17 | 5435.45(5420.15,5450.78) | 1375.92(1370.41,1381.45) | -4.42(-5.05,-3.78) |
| Denmark | male | 255.09(119.01,541.42) | 110.42(47.84,222.61) | -56.71 | 54.84(48.13,62.33) | 21.97(17.97,26.70) | -3.20(-3.44,-2.96) |
| Djibouti | male | 6395.76(4584.21,9142.41) | 4419.54(3022.85,6458.55) | -30.90 | 5919.59(5754.64,6088.30) | 1871.63(1810.88,1933.98) | -3.82(-4.20,-3.44) |
| Dominica | male | 125.50(86.13,176.03) | 43.93(28.98,62.50) | -65.00 | 883.36(720.15,1074.92) | 668.27(462.59,941.75) | -0.78(-1.12,-0.44) |
| Dominican Republic | male | 54638.33(43963.64,66437.26) | 12974.48(8726.13,18053.78) | -76.25 | 3185.82(3155.18,3216.70) | 754.24(739.73,768.99) | -4.29(-4.80,-3.79) |
| Ecuador | male | 34150.48(29244.04,40603.04) | 7272.14(5496.88,9576.46) | -78.71 | 1302.85(1286.29,1319.58) | 273.56(266.74,280.52) | -5.51(-6.39,-4.62) |
| Egypt | male | 123796.06(85284.51,174434.78) | 69846.31(44856.25,102771.43) | -43.58 | 972.26(966.36,978.20) | 337.44(334.71,340.20) | -2.84(-3.04,-2.63) |
| El Salvador | male | 16850.26(13528.03,20919.99) | 2922.27(2065.90,4223.98) | -82.66 | 1263.23(1241.73,1285.03) | 277.99(266.80,289.55) | -5.02(-5.35,-4.69) |
| Equatorial Guinea | male | 5169.83(3415.86,7230.19) | 2710.05(1573.76,4388.35) | -47.58 | 4047.86(3923.68,4175.19) | 878.07(842.45,914.89) | -6.15(-6.66,-5.64) |
| Eritrea | male | 163702.75(104205.24,233380.09) | 39581.79(27971.37,56613.77) | -75.82 | 16300.24(16208.16,16392.73) | 2646.94(2617.20,2676.94) | -5.70(-6.02,-5.38) |
| Estonia | male | 641.19(425.47,928.09) | 112.90(66.44,188.67) | -82.39 | 349.07(320.40,379.73) | 101.96(82.63,124.71) | -4.65(-4.99,-4.30) |
| Eswatini | male | 6400.79(4595.40,8757.50) | 3534.43(2388.55,5139.12) | -44.78 | 2356.55(2286.46,2428.34) | 1443.75(1390.15,1499.01) | -1.36(-1.70,-1.01) |
| Ethiopia | male | 1512472.88(1084987.26,2116030.79) | 486835.37(373491.09,622209.80) | -67.81 | 10188.33(10169.85,10206.85) | 1942.18(1936.04,1948.34) | -6.05(-6.41,-5.69) |
| Fiji | male | 1433.00(965.85,2048.76) | 1102.69(714.84,1662.68) | -23.05 | 892.40(840.79,946.49) | 673.81(628.33,721.88) | -0.79(-0.86,-0.72) |
| Finland | male | 273.02(128.05,568.74) | 92.70(42.14,186.69) | -66.05 | 54.19(47.89,61.21) | 20.46(16.41,25.38) | -3.29(-3.62,-2.96) |
| France | male | 4230.28(2371.37,7827.18) | 1580.19(891.81,3031.88) | -62.65 | 68.55(66.43,70.72) | 25.18(23.89,26.53) | -3.19(-3.48,-2.89) |
| Gabon | male | 5027.72(3463.14,6918.39) | 3368.94(1957.32,5563.84) | -32.99 | 2146.21(2081.12,2212.92) | 1017.67(980.51,1055.95) | -2.44(-2.88,-2.01) |
| Gambia | male | 15489.51(11651.62,20474.83) | 10507.12(6945.92,15408.72) | -32.17 | 5678.00(5576.36,5781.08) | 1949.90(1909.00,1991.50) | -3.90(-4.15,-3.66) |
| Georgia | male | 5021.58(2932.12,8329.14) | 1803.69(958.08,3174.02) | -64.08 | 720.00(698.98,741.52) | 468.69(445.75,492.55) | -1.24(-1.33,-1.14) |
| Germany | male | 3191.17(1320.34,6295.65) | 1180.19(477.94,2543.25) | -63.02 | 46.45(44.81,48.13) | 18.49(17.43,19.61) | -2.68(-3.09,-2.27) |
| Ghana | male | 190324.76(129624.85,273034.47) | 110546.44(71673.14,168509.74) | -41.92 | 4638.36(4614.40,4662.41) | 1540.66(1530.60,1550.77) | -3.49(-3.65,-3.33) |
| Greece | male | 370.64(135.62,863.79) | 155.10(57.89,307.89) | -58.15 | 33.25(29.86,36.99) | 21.11(17.86,24.87) | -1.35(-1.69,-1.01) |
| Greenland | male | 9.04(4.75,15.85) | 3.25(1.51,5.78) | -64.09 | 117.11(50.14,236.28) | 53.06(10.57,164.98) | -2.78(-2.99,-2.58) |
| Grenada | male | 207.69(154.02,281.66) | 69.52(47.62,105.83) | -66.53 | 1006.23(854.56,1178.46) | 573.74(431.84,750.70) | -1.71(-2.12,-1.30) |
| Guam | male | 113.98(78.93,169.22) | 60.64(37.78,92.54) | -46.80 | 397.69(315.74,495.67) | 269.62(196.41,362.56) | -1.26(-1.57,-0.94) |
| Guatemala | male | 93335.31(81128.86,106144.64) | 22588.15(17634.91,28893.99) | -75.80 | 3426.07(3400.35,3451.94) | 761.51(749.86,773.31) | -4.31(-4.66,-3.96) |
| Guinea | male | 131753.91(91554.13,186606.72) | 86736.23(57646.39,126402.86) | -34.17 | 6871.43(6827.54,6915.55) | 2409.36(2391.30,2427.54) | -3.20(-3.46,-2.93) |
| Guinea-Bissau | male | 19327.83(12996.29,28319.89) | 7862.89(4815.83,11370.21) | -59.32 | 6830.31(6720.72,6941.30) | 1625.95(1587.39,1665.25) | -4.75(-4.93,-4.57) |
| Guyana | male | 6185.72(5033.06,7422.46) | 1055.45(775.10,1477.22) | -82.94 | 3487.16(3394.55,3581.88) | 877.70(820.39,938.17) | -3.69(-4.06,-3.32) |
| Haiti | male | 101628.55(70184.44,138413.27) | 61842.02(42044.20,89470.44) | -39.15 | 5934.93(5894.32,5975.77) | 2435.61(2414.52,2456.86) | -2.90(-3.08,-2.71) |
| Honduras | male | 14990.56(11247.65,19933.80) | 6981.41(4527.74,10513.42) | -53.43 | 1157.26(1136.91,1177.90) | 385.47(375.58,395.58) | -3.49(-3.73,-3.25) |
| Hungary | male | 5262.86(3086.10,8288.59) | 1534.88(857.91,2559.78) | -70.84 | 509.33(494.48,524.55) | 215.58(204.15,227.52) | -2.66(-2.86,-2.46) |
| Iceland | male | 11.24(5.06,23.03) | 5.60(2.45,11.75) | -50.18 | 33.40(16.59,62.58) | 15.62(5.32,38.18) | -2.49(-2.78,-2.20) |
| India | male | 7430365.38(5912789.92,9427088.79) | 2525090.07(1784505.25,3501533.18) | -66.02 | 4065.15(4061.98,4068.32) | 1345.05(1343.24,1346.86) | -3.46(-3.62,-3.31) |
| Indonesia | male | 710627.69(552758.46,911011.50) | 206265.62(144374.17,293459.08) | -70.97 | 1947.26(1942.26,1952.28) | 582.86(580.12,585.62) | -3.79(-3.91,-3.68) |
| Iran (Islamic Republic of) | male | 139511.38(100720.94,191957.30) | 29545.36(17144.49,49235.30) | -78.82 | 1066.31(1060.28,1072.36) | 294.73(291.09,298.40) | -3.83(-3.95,-3.72) |
| Iraq | male | 55817.42(37808.39,79808.70) | 29345.30(18631.94,46592.92) | -47.43 | 1207.80(1196.98,1218.68) | 431.54(426.20,436.93) | -3.58(-3.83,-3.34) |
| Ireland | male | 289.56(130.68,574.98) | 85.46(41.03,159.67) | -70.48 | 55.34(48.99,62.39) | 16.15(12.83,20.18) | -4.22(-4.47,-3.98) |
| Israel | male | 543.37(242.09,1144.48) | 346.05(148.56,703.72) | -36.31 | 67.75(62.07,73.85) | 25.32(22.68,28.21) | -3.22(-3.47,-2.98) |
| Italy | male | 2195.34(991.85,5184.11) | 956.95(416.98,2217.21) | -56.41 | 45.80(43.83,47.85) | 24.36(22.77,26.05) | -2.00(-2.21,-1.79) |
| Jamaica | male | 6326.48(5085.81,7782.93) | 1231.27(728.83,2074.17) | -80.54 | 1193.60(1158.78,1229.26) | 413.24(387.85,439.96) | -3.05(-3.78,-2.32) |
| Japan | male | 7533.39(3613.32,16184.14) | 3078.71(1491.62,6558.54) | -59.13 | 66.90(65.26,68.58) | 40.27(38.73,41.86) | -1.55(-1.65,-1.46) |
| Jordan | male | 7291.81(4320.81,11460.06) | 6393.78(3736.44,10167.83) | -12.32 | 827.00(806.73,847.67) | 341.70(332.57,351.03) | -3.21(-3.40,-3.02) |
| Kazakhstan | male | 34138.14(21632.59,55765.76) | 13579.95(6691.72,34192.75) | -60.22 | 1197.88(1183.98,1211.90) | 458.09(449.74,466.57) | -3.95(-4.36,-3.54) |
| Kenya | male | 267792.71(197819.08,358210.70) | 140446.93(111819.71,177931.42) | -47.55 | 3597.06(3581.06,3613.11) | 1372.92(1364.72,1381.15) | -2.93(-3.09,-2.77) |
| Kiribati | male | 953.26(706.16,1285.75) | 510.37(365.15,698.52) | -46.46 | 3242.77(2964.95,3541.26) | 1620.89(1446.32,1811.93) | -2.21(-2.30,-2.12) |
| Kuwait | male | 967.29(596.93,1502.70) | 639.95(349.82,1095.03) | -33.84 | 325.09(303.30,348.06) | 149.84(137.50,163.05) | -2.63(-2.89,-2.37) |
| Kyrgyzstan | male | 9605.98(6291.35,14795.81) | 6476.17(3862.53,11033.65) | -32.58 | 1079.62(1056.63,1103.00) | 531.70(517.78,545.92) | -2.63(-2.86,-2.39) |
| Lao People's Democratic Republic | male | 55557.21(35847.67,90071.86) | 10365.43(6901.48,15044.47) | -81.34 | 5057.51(5011.98,5103.38) | 809.64(792.90,826.66) | -6.18(-6.37,-5.99) |
| Latvia | male | 1102.77(711.36,1676.96) | 210.46(116.97,346.50) | -80.92 | 366.64(343.74,390.71) | 137.74(118.32,159.61) | -3.57(-3.75,-3.38) |
| Lebanon | male | 3909.34(2461.01,5847.08) | 1530.14(880.73,2494.69) | -60.86 | 692.38(669.16,716.22) | 234.35(221.76,247.50) | -3.58(-3.70,-3.46) |
| Lesotho | male | 12442.96(9062.70,16442.62) | 7682.80(5178.38,11334.22) | -38.26 | 2929.38(2870.66,2989.07) | 2226.97(2171.61,2283.48) | -0.65(-0.92,-0.37) |
| Liberia | male | 71357.47(47734.77,105429.70) | 18987.52(12111.09,28471.84) | -73.39 | 8541.00(8464.61,8617.94) | 1516.28(1492.05,1540.82) | -6.33(-6.83,-5.83) |
| Libya | male | 5899.69(3740.61,8618.21) | 2849.83(1784.14,4511.62) | -51.70 | 627.38(610.11,645.03) | 399.68(383.69,416.21) | -1.39(-1.70,-1.08) |
| Lithuania | male | 1378.15(896.65,2040.21) | 284.99(165.15,475.39) | -79.32 | 312.73(295.12,331.16) | 136.01(119.51,154.26) | -2.86(-2.94,-2.79) |
| Luxembourg | male | 18.37(8.99,36.26) | 9.60(4.73,20.15) | -47.74 | 52.19(30.64,84.82) | 17.48(7.99,34.66) | -3.44(-3.82,-3.07) |
| Madagascar | male | 473042.46(377989.98,571338.13) | 194263.97(132535.10,277235.15) | -58.93 | 13126.39(13081.67,13171.24) | 3011.90(2996.88,3026.97) | -4.21(-4.43,-3.98) |
| Malawi | male | 283207.02(200548.96,399078.90) | 89501.52(61742.89,126178.71) | -68.40 | 8823.79(8784.98,8862.74) | 1997.07(1982.24,2011.99) | -5.12(-5.41,-4.82) |
| Malaysia | male | 30749.57(20185.15,43434.79) | 14641.09(8987.30,22625.38) | -52.39 | 855.13(844.71,865.66) | 374.86(368.21,381.60) | -2.77(-2.87,-2.67) |
| Maldives | male | 2181.79(1455.60,3102.74) | 278.94(175.02,448.04) | -87.22 | 3539.74(3378.76,3706.68) | 565.38(495.92,642.28) | -6.05(-6.52,-5.57) |
| Mali | male | 326652.90(214182.03,464161.14) | 447715.82(311323.04,627782.95) | 37.06 | 12149.82(12100.58,12199.21) | 6614.55(6593.14,6636.02) | -1.82(-2.16,-1.47) |
| Malta | male | 27.67(12.13,57.39) | 7.13(3.09,14.28) | -74.21 | 59.97(39.40,88.82) | 21.12(8.42,45.61) | -3.28(-3.52,-3.05) |
| Marshall Islands | male | 148.85(100.98,207.31) | 85.38(54.31,128.95) | -42.64 | 1117.27(919.95,1346.71) | 869.01(672.69,1108.17) | -0.88(-1.03,-0.72) |
| Mauritania | male | 24829.91(16733.39,36966.96) | 11380.05(6908.60,19720.40) | -54.17 | 4220.20(4159.55,4281.55) | 1141.75(1118.96,1164.91) | -4.16(-4.25,-4.06) |
| Mauritius | male | 1888.90(1342.29,3086.39) | 432.44(265.92,797.04) | -77.11 | 1080.86(1026.87,1137.14) | 421.90(378.93,468.65) | -2.40(-2.66,-2.13) |
| Mexico | male | 395737.68(350589.67,446772.22) | 54422.63(42124.03,70932.90) | -86.25 | 1930.50(1923.61,1937.40) | 320.14(317.15,323.16) | -5.56(-5.72,-5.39) |
| Micronesia (Federated States of) | male | 359.61(247.15,525.74) | 123.17(77.12,191.49) | -65.75 | 1258.52(1111.31,1420.78) | 747.28(605.65,914.04) | -1.74(-1.81,-1.66) |
| Monaco | male | 0.38(0.17,0.82) | 0.31(0.13,0.68) | -19.93 | 19.92(0.00,327.88) | 12.00(0.00,228.57) | -1.78(-1.89,-1.67) |
| Mongolia | male | 7515.28(4824.27,10454.00) | 4524.33(2779.29,7218.63) | -39.80 | 1604.80(1566.47,1643.87) | 789.62(765.19,814.66) | -2.65(-2.82,-2.47) |
| Montenegro | male | 275.32(150.89,425.78) | 126.18(73.28,212.74) | -54.17 | 333.22(292.26,378.63) | 219.08(179.97,264.69) | -1.86(-2.12,-1.61) |
| Morocco | male | 66461.92(44433.68,97954.45) | 27123.56(16767.32,42613.43) | -59.19 | 1258.59(1248.30,1268.95) | 535.90(529.02,542.84) | -2.73(-2.96,-2.49) |
| Mozambique | male | 394540.23(241830.28,604604.97) | 145929.71(98307.95,213543.28) | -63.01 | 10400.51(10362.82,10438.32) | 1682.90(1672.99,1692.85) | -6.15(-6.34,-5.96) |
| Myanmar | male | 259348.29(178089.35,362001.88) | 79893.67(47084.18,137558.82) | -69.19 | 3182.11(3168.54,3195.73) | 921.36(914.37,928.40) | -4.30(-4.43,-4.17) |
| Namibia | male | 13324.06(10089.70,17443.03) | 7275.85(4959.21,10298.76) | -45.39 | 3475.62(3406.03,3546.32) | 1445.93(1406.80,1485.93) | -2.61(-2.80,-2.42) |
| Nauru | male | 30.22(20.75,42.50) | 18.96(12.16,28.49) | -37.25 | 965.24(586.70,1506.85) | 721.44(386.84,1243.11) | -0.93(-1.41,-0.45) |
| Nepal | male | 291199.77(198984.33,412693.59) | 46510.22(31487.37,73714.16) | -84.03 | 5365.09(5343.15,5387.11) | 930.95(921.76,940.21) | -5.60(-5.85,-5.35) |
| Netherlands | male | 623.75(318.82,1146.86) | 334.62(152.04,962.18) | -46.35 | 43.94(40.51,47.61) | 23.79(21.27,26.57) | -2.09(-2.37,-1.81) |
| New Zealand | male | 605.10(196.38,1850.67) | 439.02(113.07,1632.35) | -27.45 | 126.65(115.56,138.60) | 81.81(73.49,90.87) | -1.24(-1.41,-1.08) |
| Nicaragua | male | 19226.39(14614.45,24938.01) | 4563.94(3102.97,6749.02) | -76.26 | 1777.23(1749.69,1805.12) | 401.10(388.08,414.48) | -5.15(-5.36,-4.94) |
| Niger | male | 265599.09(172299.30,428455.36) | 159171.08(108308.70,225147.74) | -40.07 | 9481.89(9438.57,9525.37) | 2035.85(2024.50,2047.24) | -5.45(-5.69,-5.21) |
| Nigeria | male | 754560.23(578691.31,1004963.65) | 777650.36(523776.08,1138851.92) | 3.06 | 2946.15(2938.27,2954.05) | 1384.74(1381.34,1388.15) | -2.46(-2.71,-2.21) |
| Niue | male | 4.17(2.83,6.28) | 1.80(1.30,2.50) | -56.86 | 756.01(134.74,2461.60) | 736.00(28.00,4048.05) | -1.15(-1.55,-0.75) |
| North Macedonia | male | 1759.71(1044.33,2739.51) | 506.37(295.07,830.15) | -71.22 | 659.13(626.62,692.98) | 304.89(276.95,335.08) | -2.80(-2.99,-2.61) |
| Northern Mariana Islands | male | 28.32(17.73,45.53) | 17.06(10.21,27.71) | -39.76 | 368.81(227.24,571.12) | 290.92(155.16,504.45) | -0.25(-0.51,0.01) |
| Norway | male | 229.89(102.69,490.28) | 110.05(45.81,248.10) | -52.13 | 55.19(48.08,63.14) | 22.87(18.63,27.90) | -2.76(-3.10,-2.42) |
| Oman | male | 6507.18(4695.94,9433.37) | 2298.42(1414.44,3774.88) | -64.68 | 1274.72(1240.37,1309.82) | 342.24(326.96,358.08) | -3.65(-4.07,-3.22) |
| Pakistan | male | 915274.15(683670.99,1200593.03) | 1004901.64(665399.01,1485111.09) | 9.79 | 3223.13(3215.92,3230.34) | 2211.54(2206.89,2216.20) | -1.05(-1.16,-0.94) |
| Palau | male | 17.10(11.32,25.64) | 7.29(4.41,12.28) | -57.35 | 645.06(342.03,1124.93) | 422.46(148.03,975.26) | -1.15(-1.27,-1.04) |
| Palestine | male | 3706.84(2339.37,5613.66) | 2505.51(1387.28,4111.39) | -32.41 | 645.04(622.17,668.56) | 256.45(245.74,267.54) | -3.03(-3.12,-2.93) |
| Panama | male | 4505.67(3560.81,5705.20) | 2306.02(1723.61,3143.25) | -48.82 | 855.47(826.63,885.12) | 269.22(255.12,283.94) | -4.04(-4.40,-3.69) |
| Papua New Guinea | male | 11386.63(7569.69,16034.62) | 22639.41(14292.49,36674.95) | 98.82 | 1018.17(996.61,1040.09) | 905.91(892.44,919.53) | -0.04(-0.23,0.16) |
| Paraguay | male | 8190.69(5778.09,11503.60) | 5405.44(3692.21,7662.58) | -34.01 | 880.29(859.72,901.25) | 527.81(512.74,543.24) | -1.79(-2.16,-1.43) |
| Peru | male | 129496.50(99910.85,168046.77) | 24601.13(17235.05,35768.68) | -81.00 | 2341.88(2326.63,2357.21) | 425.26(419.24,431.35) | -5.91(-6.18,-5.63) |
| Philippines | male | 251773.57(203045.01,318850.97) | 121683.72(87282.88,171124.48) | -51.67 | 1691.50(1684.09,1698.93) | 655.10(651.00,659.22) | -2.68(-2.88,-2.48) |
| Poland | male | 29409.36(16903.71,46293.54) | 7144.35(3947.23,12612.64) | -75.71 | 616.58(609.04,624.20) | 239.61(233.70,245.64) | -3.12(-3.25,-3.00) |
| Portugal | male | 992.11(486.01,2053.41) | 201.09(97.77,384.02) | -79.73 | 88.35(82.74,94.32) | 28.08(24.23,32.45) | -3.72(-4.11,-3.33) |
| Puerto Rico | male | 2106.51(1380.44,3266.42) | 366.31(221.80,604.20) | -82.61 | 397.15(378.61,416.41) | 184.02(163.37,206.75) | -2.75(-3.19,-2.31) |
| Qatar | male | 291.66(169.50,462.83) | 327.18(187.32,554.93) | 12.18 | 407.68(358.07,462.45) | 120.12(106.29,135.34) | -4.36(-4.51,-4.21) |
| Republic of Korea | male | 7217.43(3747.39,12308.85) | 736.92(381.97,1402.43) | -89.79 | 120.39(117.36,123.47) | 25.18(23.22,27.28) | -4.86(-5.40,-4.32) |
| Republic of Moldova | male | 2933.73(1908.60,4422.54) | 459.41(261.29,754.22) | -84.34 | 444.68(427.36,462.55) | 169.74(153.14,187.77) | -3.34(-3.61,-3.07) |
| Romania | male | 19380.41(11252.50,30118.74) | 4302.89(2555.53,6648.53) | -77.80 | 700.00(689.46,710.67) | 284.31(275.23,293.63) | -3.10(-3.21,-2.99) |
| Russian Federation | male | 61719.61(37878.29,100438.92) | 19128.96(11477.37,31825.81) | -69.01 | 344.72(341.79,347.67) | 146.62(144.36,148.91) | -3.43(-3.89,-2.97) |
| Rwanda | male | 157999.11(112029.05,226492.10) | 32861.72(23215.41,45907.33) | -79.20 | 7855.82(7812.18,7899.66) | 1153.43(1139.38,1167.62) | -7.61(-8.28,-6.94) |
| Saint Kitts and Nevis | male | 144.34(116.93,192.75) | 38.47(28.34,53.51) | -73.35 | 1716.98(1409.22,2076.22) | 598.47(388.51,889.35) | -3.18(-3.82,-2.54) |
| Saint Lucia | male | 343.43(255.24,463.50) | 74.16(47.84,111.45) | -78.41 | 1119.95(987.99,1265.63) | 472.44(359.82,611.76) | -2.74(-3.21,-2.26) |
| Saint Vincent and the Grenadines | male | 391.70(311.92,512.19) | 95.48(70.49,137.89) | -75.62 | 1717.94(1529.75,1924.43) | 692.26(539.72,877.30) | -2.95(-3.44,-2.46) |
| Samoa | male | 425.80(291.31,622.60) | 270.78(162.93,419.33) | -36.41 | 880.69(782.71,988.23) | 540.88(468.91,621.26) | -1.45(-1.58,-1.31) |
| San Marino | male | 0.68(0.30,1.32) | 0.43(0.19,0.87) | -36.25 | 30.90(0.13,330.27) | 17.84(0.00,290.59) | -1.58(-2.00,-1.16) |
| Sao Tome and Principe | male | 2254.45(1607.21,2979.66) | 449.48(283.79,746.35) | -80.06 | 6426.88(6124.28,6741.38) | 1128.43(1018.43,1247.81) | -5.56(-6.11,-5.00) |
| Saudi Arabia | male | 41141.60(28745.19,56568.81) | 9428.16(5717.07,15426.42) | -77.08 | 1138.45(1126.45,1150.55) | 246.44(241.07,251.91) | -4.51(-4.61,-4.41) |
| Senegal | male | 108843.86(74928.18,149072.90) | 59817.36(33997.84,89622.86) | -45.04 | 5088.50(5054.18,5123.01) | 1718.92(1704.13,1733.82) | -3.91(-4.11,-3.71) |
| Serbia | male | 5890.16(3357.42,9809.55) | 1615.54(896.49,2712.46) | -72.57 | 578.17(562.23,594.47) | 246.23(233.29,259.76) | -3.10(-3.26,-2.95) |
| Seychelles | male | 79.05(53.32,114.71) | 40.30(25.88,62.61) | -49.02 | 626.77(484.88,799.54) | 321.36(221.57,453.25) | -1.87(-2.07,-1.67) |
| Sierra Leone | male | 125048.58(84864.90,176673.12) | 124051.26(79797.94,191524.82) | -0.80 | 10010.90(9946.39,10075.74) | 5297.25(5262.52,5332.15) | -1.41(-1.80,-1.02) |
| Singapore | male | 340.13(170.14,699.22) | 104.51(48.21,225.80) | -69.27 | 97.78(86.51,110.25) | 23.68(19.00,29.21) | -4.54(-4.92,-4.16) |
| Slovakia | male | 3198.15(1781.93,5200.71) | 985.65(597.43,1630.63) | -69.18 | 486.21(468.25,504.74) | 224.39(209.63,239.96) | -2.64(-2.69,-2.58) |
| Slovenia | male | 674.98(391.30,1096.14) | 203.46(112.69,346.05) | -69.86 | 331.39(304.96,359.64) | 128.11(109.92,148.64) | -3.48(-3.67,-3.28) |
| Solomon Islands | male | 1389.47(938.34,1920.30) | 1440.64(921.72,2176.18) | 3.68 | 1300.47(1221.00,1384.01) | 910.65(857.89,965.95) | -0.96(-1.05,-0.86) |
| Somalia | male | 241335.96(160360.36,351532.29) | 226861.77(154565.68,316952.24) | -6.00 | 10312.26(10266.30,10358.38) | 3693.33(3676.75,3709.97) | -2.25(-4.87,0.45) |
| South Africa | male | 278573.12(222153.46,360316.38) | 141882.37(109870.99,180615.53) | -49.07 | 2833.09(2819.81,2846.43) | 1719.72(1709.74,1729.75) | -1.33(-1.48,-1.18) |
| South Sudan | male | 250133.93(165301.20,367850.15) | 141969.39(90904.80,218691.94) | -43.24 | 16311.10(16240.76,16381.69) | 5681.12(5647.62,5714.78) | -3.47(-4.09,-2.84) |
| Spain | male | 2015.92(853.26,4130.53) | 655.87(270.43,1357.01) | -67.47 | 47.57(45.43,49.81) | 19.01(17.53,20.59) | -3.08(-3.41,-2.75) |
| Sri Lanka | male | 26119.44(17683.45,38888.36) | 9806.84(6505.35,14343.48) | -62.45 | 935.76(923.21,948.44) | 399.61(390.89,408.48) | -2.84(-2.98,-2.70) |
| Sudan | male | 231413.54(148808.31,387927.60) | 100453.21(66479.51,152684.38) | -56.59 | 4025.07(4006.29,4043.93) | 1095.07(1087.59,1102.59) | -4.14(-4.31,-3.97) |
| Suriname | male | 1219.08(897.47,1683.26) | 510.14(346.27,787.80) | -58.15 | 1750.95(1645.53,1861.80) | 676.25(612.63,745.10) | -3.22(-3.49,-2.96) |
| Sweden | male | 330.81(148.54,628.34) | 198.10(79.37,487.64) | -40.12 | 41.01(36.61,45.82) | 20.81(17.94,24.06) | -2.19(-2.46,-1.91) |
| Switzerland | male | 218.99(110.53,433.98) | 115.28(55.93,243.42) | -47.36 | 35.48(30.82,40.72) | 16.38(13.46,19.82) | -2.57(-2.87,-2.27) |
| Syrian Arab Republic | male | 45096.46(32566.85,61480.95) | 9243.06(6065.93,13460.34) | -79.50 | 1389.62(1375.74,1403.62) | 538.90(526.56,551.46) | -2.70(-3.06,-2.34) |
| Taiwan (Province of China) | male | 6678.00(4261.89,10109.41) | 1251.07(761.56,1986.92) | -81.27 | 239.03(232.72,245.48) | 84.50(79.44,89.80) | -3.42(-3.91,-2.94) |
| Tajikistan | male | 20943.65(14567.06,29071.17) | 20620.16(12730.67,30827.02) | -1.54 | 1618.64(1594.98,1642.57) | 1061.57(1046.06,1077.27) | -1.71(-1.97,-1.45) |
| Thailand | male | 44519.03(29041.28,66972.68) | 10961.64(7383.08,17072.92) | -75.38 | 529.05(523.64,534.51) | 228.16(223.44,232.97) | -2.49(-2.65,-2.34) |
| Timor-Leste | male | 17072.86(9285.31,32255.02) | 3896.87(2689.20,5558.10) | -77.18 | 7951.85(7822.71,8082.75) | 1294.53(1249.55,1340.81) | -6.34(-6.79,-5.89) |
| Togo | male | 37324.66(24464.75,52802.21) | 30761.76(16733.84,47917.19) | -17.58 | 3669.74(3628.18,3711.67) | 1696.02(1675.56,1716.68) | -2.72(-2.98,-2.45) |
| Tokelau | male | 3.01(1.91,4.64) | 1.69(1.17,2.36) | -44.05 | 897.54(142.49,3095.92) | 791.83(26.92,4470.34) | -1.55(-2.03,-1.08) |
| Tonga | male | 240.00(167.90,333.90) | 148.52(99.54,229.58) | -38.12 | 797.30(678.04,932.61) | 559.20(456.66,678.89) | -0.94(-1.03,-0.84) |
| Trinidad and Tobago | male | 2072.00(1461.88,3092.91) | 531.88(334.01,836.27) | -74.33 | 968.74(923.37,1015.86) | 386.64(351.14,424.97) | -3.42(-3.61,-3.23) |
| Tunisia | male | 11867.87(7647.29,17820.65) | 4126.99(2375.21,6647.85) | -65.23 | 729.79(715.69,744.12) | 287.11(277.67,296.80) | -2.97(-3.05,-2.89) |
| Türkiye | male | 133654.55(94656.15,193075.02) | 24483.14(14411.64,38557.25) | -81.68 | 1215.76(1208.72,1222.83) | 259.98(256.39,263.61) | -5.14(-5.34,-4.94) |
| Turkmenistan | male | 11227.49(7443.11,15940.36) | 5654.02(3328.84,9221.69) | -49.64 | 1341.47(1314.50,1368.86) | 706.28(686.65,726.35) | -2.27(-2.40,-2.14) |
| Tuvalu | male | 50.37(36.21,70.03) | 15.38(9.95,23.50) | -69.46 | 1727.93(1189.53,2450.75) | 697.94(360.39,1237.88) | -2.70(-2.89,-2.51) |
| Uganda | male | 351931.19(226020.71,534000.67) | 167536.52(103752.97,250610.32) | -52.40 | 5425.18(5403.50,5446.94) | 1334.03(1326.61,1341.48) | -4.81(-5.05,-4.57) |
| Ukraine | male | 31844.84(22803.52,43157.83) | 8921.19(5843.23,13507.26) | -71.99 | 547.91(541.40,554.49) | 306.10(299.01,313.31) | -2.22(-2.53,-1.92) |
| United Arab Emirates | male | 2508.53(1697.00,3745.63) | 2380.97(1411.01,4029.07) | -5.08 | 755.91(723.86,789.09) | 345.35(330.54,360.69) | -2.38(-2.55,-2.22) |
| United Kingdom | male | 5126.20(2417.02,9507.85) | 3773.71(1221.80,9816.63) | -26.38 | 84.72(82.25,87.25) | 63.79(61.65,65.99) | -0.60(-0.87,-0.34) |
| United Republic of Tanzania | male | 627937.92(453620.97,845041.97) | 233753.01(155595.70,356208.97) | -62.77 | 8069.36(8046.72,8092.05) | 1532.48(1525.25,1539.75) | -5.74(-5.96,-5.51) |
| United States of America | male | 15831.92(9235.40,26127.17) | 17889.26(11230.24,27168.00) | 12.99 | 50.68(49.83,51.54) | 57.16(56.28,58.05) | 1.16(0.66,1.67) |
| United States Virgin Islands | male | 110.26(74.15,166.33) | 18.49(10.59,30.51) | -83.23 | 613.57(493.09,756.56) | 262.52(146.30,441.00) | -2.68(-3.01,-2.35) |
| Uruguay | male | 2464.56(1922.78,3465.51) | 437.36(229.36,885.84) | -82.25 | 550.49(527.05,574.78) | 124.04(111.05,138.21) | -4.68(-4.87,-4.50) |
| Uzbekistan | male | 68126.88(43709.80,101049.38) | 47670.26(28168.36,78090.37) | -30.03 | 1436.65(1425.01,1448.36) | 866.43(858.11,874.81) | -1.97(-2.06,-1.87) |
| Vanuatu | male | 494.89(345.98,695.54) | 640.06(425.01,911.52) | 29.33 | 1031.12(926.04,1145.42) | 907.43(829.14,991.45) | -0.52(-0.66,-0.39) |
| Venezuela (Bolivarian Republic of) | male | 48544.44(40141.14,59723.51) | 17467.07(12428.46,23690.95) | -64.02 | 1062.19(1051.34,1073.14) | 422.54(415.23,429.95) | -3.69(-4.35,-3.03) |
| Viet Nam | male | 239731.69(166623.08,344322.84) | 45126.60(28972.07,68961.95) | -81.18 | 1672.19(1664.91,1679.50) | 345.26(341.78,348.78) | -5.22(-5.42,-5.02) |
| Yemen | male | 173111.21(124202.31,248485.74) | 184672.16(133823.17,254829.37) | 6.68 | 4059.02(4038.19,4079.93) | 2514.55(2502.08,2527.07) | -1.77(-1.87,-1.66) |
| Zambia | male | 179509.64(119446.75,281510.07) | 96401.53(60365.57,137398.33) | -46.30 | 6896.43(6857.95,6935.09) | 2110.53(2095.95,2125.20) | -4.02(-4.51,-3.54) |
| Zimbabwe | male | 80433.69(58344.87,107981.24) | 114650.53(81390.45,157289.32) | 42.54 | 2824.29(2802.31,2846.41) | 2840.89(2821.19,2860.70) | 0.62(0.29,0.95) |

**Stable 2. Incidence cases and ASIR of Nutritional deficiencies in 1990 and 2021 and its trends.**

| location | sex | number | | | Age-standardized rate | | |
| --- | --- | --- | --- | --- | --- | --- | --- |
|  |  | 1990 | 2021 | change rate in case(%) | 1990 | 2021 | EAPC |
| Global | both | 477650346.66(452179852.92,503276987.23) | 231618972.25(218064930.54,246907501.12) | -51.51 | 26959.89(26957.29,26962.49) | 11563.37(11561.77,11564.97) | -2.66(-2.89,-2.43) |
| Central Europe, eastern Europe, and central Asia | both | 10322248.41(9595856.08,11117758.37) | 3682690.51(3368978.18,4015853.26) | -64.32 | 9701.03(9694.74,9707.32) | 4581.61(4576.61,4586.63) | -2.53(-2.72,-2.35) |
| High-income | both | 7299231.92(6663515.72,8068232.25) | 3177723.13(2780450.90,3566959.87) | -56.46 | 3946.99(3943.96,3950.01) | 1811.57(1809.47,1813.68) | -2.24(-2.40,-2.08) |
| Latin America and Caribbean | both | 30706792.04(28443679.05,33141934.35) | 11674520.53(10287640.74,13301167.28) | -61.98 | 21317.14(21309.16,21325.11) | 8181.89(8176.93,8186.86) | -3.16(-3.24,-3.08) |
| Southeast Asia, east Asia, and Oceania | both | 118320563.55(107016104.91,129030203.05) | 28130059.26(25162035.66,31612530.06) | -76.23 | 23278.99(23274.51,23283.47) | 6388.50(6385.96,6391.03) | -3.95(-4.12,-3.79) |
| Sub-Saharan Africa | both | 111030884.53(107577013.23,114485530.20) | 108139639.15(103343722.94,113084282.81) | -2.60 | 47779.34(47769.80,47788.87) | 22276.20(22271.70,22280.70) | -2.52(-2.70,-2.35) |
| Andean Latin America | both | 2158036.84(1914010.24,2438624.72) | 1040385.07(892436.12,1215035.62) | -51.79 | 14424.73(14404.14,14445.34) | 5751.68(5740.01,5763.37) | -3.31(-3.56,-3.05) |
| Australasia | both | 34848.76(27179.46,43900.74) | 28471.13(22207.77,36405.99) | -18.30 | 739.00(730.76,747.31) | 492.17(486.14,498.27) | -0.87(-1.10,-0.64) |
| Caribbean | both | 1868283.12(1680586.22,2066452.96) | 1003619.04(864703.16,1173647.19) | -46.28 | 16085.62(16060.92,16110.35) | 8684.65(8666.49,8702.83) | -2.15(-2.23,-2.07) |
| Central Asia | both | 3070177.97(2753312.76,3430282.84) | 1781511.01(1565484.95,1985668.39) | -41.97 | 11770.93(11756.80,11785.08) | 6292.22(6282.37,6302.08) | -2.08(-2.26,-1.90) |
| Central Europe | both | 5879197.25(5357764.98,6397155.02) | 1391070.15(1272314.10,1529414.35) | -76.34 | 20175.47(20158.15,20192.80) | 7905.15(7891.26,7919.05) | -3.15(-3.29,-3.01) |
| Central Latin America | both | 12006234.71(10863642.38,13256372.40) | 4304181.63(3678013.40,5057176.88) | -64.15 | 18553.40(18542.24,18564.57) | 6886.22(6879.31,6893.14) | -3.08(-3.18,-2.99) |
| Central Sub-Saharan Africa | both | 12786567.64(11689371.08,13946911.99) | 17838402.36(15632824.04,20061523.89) | 39.51 | 48421.60(48392.98,48450.23) | 30044.22(30029.32,30059.13) | -1.45(-1.88,-1.02) |
| East Asia | both | 61142423.29(51473737.80,71631666.39) | 10306917.60(8472025.48,12594286.89) | -83.14 | 18357.21(18352.32,18362.10) | 3882.54(3880.04,3885.04) | -4.80(-4.93,-4.68) |
| Eastern Europe | both | 1372873.18(1139749.71,1622856.89) | 510109.35(406631.30,625530.23) | -62.84 | 2663.98(2659.16,2668.80) | 1499.78(1495.25,1504.33) | -1.52(-1.72,-1.31) |
| Eastern Sub-Saharan Africa | both | 48944744.19(47079101.55,50750637.37) | 41911470.49(39211879.40,44542197.51) | -14.37 | 52293.16(52277.44,52308.87) | 23159.12(23151.62,23166.62) | -2.79(-2.99,-2.60) |
| High-income Asia Pacific | both | 1200692.73(1014729.84,1413409.04) | 345187.05(285594.40,412327.33) | -71.25 | 3482.25(3475.55,3488.97) | 1584.41(1578.72,1590.12) | -2.14(-2.30,-1.98) |
| High-income North America | both | 1398380.20(1171458.17,1655417.40) | 744363.56(605465.03,905531.68) | -46.77 | 2243.17(2239.27,2247.08) | 1115.40(1112.73,1118.08) | -2.23(-2.57,-1.90) |
| North Africa and Middle East | both | 26931357.08(25407355.11,28457772.61) | 15143533.20(13825206.22,16494393.59) | -43.77 | 18684.03(18676.44,18691.62) | 8257.66(8253.17,8262.15) | -2.54(-2.81,-2.27) |
| Oceania | both | 699814.52(631857.80,782161.44) | 824655.47(724106.21,955016.32) | 17.84 | 25330.13(25266.55,25393.83) | 15458.76(15422.74,15494.84) | -1.17(-1.36,-0.98) |
| South Asia | both | 173039269.12(156210115.99,190594508.66) | 61670806.46(51971512.92,73583507.79) | -64.36 | 38815.69(38809.43,38821.95) | 12585.16(12581.74,12588.58) | -3.50(-3.83,-3.17) |
| Southeast Asia | both | 56478325.75(51826462.51,60988061.39) | 16998486.19(15236119.57,19015151.45) | -69.90 | 32877.01(32867.77,32886.25) | 9922.57(9917.46,9927.68) | -3.67(-3.78,-3.55) |
| Southern Latin America | both | 2418544.24(2104158.59,2799690.98) | 1088561.89(844642.16,1343756.04) | -54.99 | 16779.85(16757.66,16802.07) | 7678.88(7663.64,7694.14) | -2.40(-2.61,-2.19) |
| Southern Sub-Saharan Africa | both | 6216306.39(5601158.14,6923076.14) | 3137306.82(2729603.72,3617705.49) | -49.53 | 29728.04(29703.23,29752.87) | 13053.90(13038.48,13069.33) | -2.58(-2.67,-2.50) |
| Tropical Latin America | both | 14674237.37(12875041.12,16823780.89) | 5326334.80(4130854.85,6818547.97) | -63.70 | 27501.31(27486.50,27516.14) | 10596.80(10587.34,10606.28) | -3.23(-3.35,-3.12) |
| Western Europe | both | 2246765.99(2021040.62,2496486.42) | 971139.51(839034.86,1125154.16) | -56.78 | 3172.53(3168.14,3176.93) | 1425.59(1422.60,1428.58) | -2.00(-2.23,-1.77) |
| Western Sub-Saharan Africa | both | 43083266.31(41384803.40,44897594.15) | 45252459.49(42580966.69,48131899.35) | 5.03 | 47194.88(47179.75,47210.02) | 20443.95(20437.56,20450.35) | -2.74(-2.82,-2.66) |
| Afghanistan | both | 2495284.08(2257776.91,2762517.39) | 4637329.30(3880626.25,5353126.81) | 85.84 | 56190.84(56115.82,56265.93) | 31572.57(31541.52,31603.63) | -1.93(-2.31,-1.55) |
| Albania | both | 391253.12(329424.49,452968.89) | 65018.97(53105.79,79853.29) | -83.38 | 34742.39(34626.47,34858.60) | 14945.95(14824.19,15068.52) | -3.19(-3.43,-2.94) |
| Algeria | both | 1442275.71(1171845.94,1729881.72) | 650827.13(536230.70,784696.92) | -54.87 | 13355.31(13331.90,13378.75) | 4787.90(4775.50,4800.33) | -3.44(-3.52,-3.36) |
| American Samoa | both | 3070.03(2420.01,3806.58) | 1242.77(936.32,1623.48) | -59.52 | 15879.95(15289.58,16487.92) | 8938.34(8408.49,9496.47) | -1.62(-1.88,-1.37) |
| Andorra | both | 248.31(198.45,311.65) | 126.24(96.42,164.48) | -49.16 | 2701.85(2350.13,3096.49) | 1246.19(1021.34,1513.32) | -2.39(-2.49,-2.30) |
| Angola | both | 2682405.11(2356691.01,2986716.82) | 3360557.03(2717779.16,4010341.08) | 25.28 | 53775.50(53705.66,53845.40) | 21550.52(21525.80,21575.26) | -3.07(-3.28,-2.85) |
| Antigua and Barbuda | both | 1225.72(1012.89,1502.72) | 483.25(399.32,588.16) | -60.57 | 6612.15(6219.00,7025.03) | 2837.98(2571.94,3126.19) | -2.42(-2.57,-2.28) |
| Argentina | both | 1775473.70(1496901.61,2112197.35) | 876308.37(652167.29,1131236.46) | -50.64 | 18387.45(18359.11,18415.83) | 8816.85(8797.35,8836.39) | -2.29(-2.49,-2.09) |
| Armenia | both | 21547.78(17063.80,26174.68) | 8615.94(6928.08,10710.56) | -60.01 | 1958.45(1930.57,1986.65) | 1546.05(1511.52,1581.20) | -0.55(-0.72,-0.39) |
| Australia | both | 22852.44(17039.22,29890.68) | 20454.23(15690.26,26719.00) | -10.49 | 587.01(578.90,595.20) | 426.26(420.09,432.51) | -0.71(-0.89,-0.53) |
| Austria | both | 43018.73(34209.85,54568.24) | 19479.15(15173.55,24721.67) | -54.72 | 3183.18(3151.32,3215.30) | 1493.63(1471.81,1515.72) | -2.16(-2.41,-1.90) |
| Azerbaijan | both | 152700.92(123063.44,188842.52) | 99529.59(81887.63,121300.16) | -34.82 | 6039.61(6007.17,6072.18) | 4363.22(4334.20,4392.40) | -1.28(-1.82,-0.73) |
| Bahamas | both | 2859.40(2263.65,3592.98) | 1308.93(1017.71,1668.08) | -54.22 | 3572.97(3434.34,3716.19) | 1697.82(1597.57,1803.30) | -2.12(-2.25,-1.99) |
| Bahrain | both | 11536.25(9503.79,13897.99) | 8597.41(7129.67,10408.08) | -25.47 | 6750.82(6618.49,6885.21) | 2895.62(2830.80,2961.65) | -2.31(-2.48,-2.15) |
| Bangladesh | both | 14038236.14(12253982.77,15911586.36) | 2824929.34(2353807.55,3309840.05) | -79.88 | 26656.72(26641.46,26671.98) | 6248.82(6240.80,6256.85) | -4.43(-4.66,-4.20) |
| Barbados | both | 3127.48(2465.12,3857.66) | 1288.15(1054.35,1614.93) | -58.81 | 5026.47(4838.16,5220.78) | 2790.85(2627.40,2962.61) | -1.51(-1.66,-1.37) |
| Belarus | both | 62802.58(49619.53,80890.20) | 15997.11(12858.50,19483.25) | -74.53 | 2679.80(2658.03,2701.70) | 1086.14(1068.59,1103.93) | -2.77(-2.84,-2.70) |
| Belgium | both | 57418.69(45654.29,70657.75) | 22661.57(17957.19,27206.78) | -60.53 | 3176.81(3149.16,3204.65) | 1182.42(1166.22,1198.80) | -2.70(-2.90,-2.49) |
| Belize | both | 12285.38(9803.45,14903.07) | 6637.84(5130.68,8369.94) | -45.97 | 14839.61(14560.39,15123.05) | 5496.95(5356.26,5640.65) | -2.97(-3.05,-2.89) |
| Benin | both | 1624641.17(1493837.71,1760295.53) | 2109547.00(1796341.29,2478619.00) | 29.85 | 65175.36(65068.20,65282.67) | 33718.37(33669.63,33767.18) | -2.06(-2.15,-1.96) |
| Bermuda | both | 417.60(328.79,532.24) | 133.83(110.67,164.68) | -67.95 | 3458.59(3116.43,3829.88) | 1598.94(1321.24,1922.69) | -2.56(-2.60,-2.51) |
| Bhutan | both | 89617.25(74298.76,103717.03) | 12151.40(9926.74,15040.48) | -86.44 | 33499.48(33262.31,33738.00) | 6464.90(6342.35,6589.38) | -5.38(-5.53,-5.23) |
| Bolivia (Plurinational State of) | both | 385814.53(307496.36,471059.26) | 227760.85(177813.38,287729.03) | -40.97 | 13991.17(13943.77,14038.70) | 6517.63(6489.37,6546.00) | -2.65(-2.89,-2.42) |
| Bosnia and Herzegovina | both | 302222.99(247921.49,367835.16) | 55527.05(42965.33,70426.33) | -81.63 | 27786.73(27682.21,27891.56) | 11426.63(11326.35,11527.65) | -3.46(-3.73,-3.20) |
| Botswana | both | 210355.49(181122.07,239309.24) | 88529.06(69965.39,109003.97) | -57.91 | 34977.95(34818.74,35137.75) | 12547.39(12459.01,12636.27) | -3.20(-3.28,-3.12) |
| Brazil | both | 14334063.47(12533767.00,16457964.73) | 5184504.32(3997121.32,6678662.66) | -63.83 | 27730.93(27715.81,27746.06) | 10743.01(10733.28,10752.75) | -3.22(-3.34,-3.11) |
| Brunei Darussalam | both | 5430.95(4423.13,6652.47) | 2322.90(1900.00,2786.20) | -57.23 | 5557.90(5398.30,5721.16) | 2311.00(2209.12,2416.67) | -2.58(-2.71,-2.46) |
| Bulgaria | both | 314867.30(245405.64,396039.77) | 105748.16(87738.76,129824.28) | -66.42 | 18121.47(18054.07,18189.08) | 10668.76(10600.78,10737.10) | -1.45(-1.66,-1.24) |
| Burkina Faso | both | 3800346.96(3565343.61,4025792.14) | 3423575.73(2842115.97,4024071.47) | -9.91 | 78223.45(78139.52,78307.44) | 31391.04(31355.21,31426.91) | -3.17(-3.31,-3.04) |
| Burundi | both | 1198730.13(1064410.96,1362989.03) | 1123409.60(915171.73,1347861.32) | -6.28 | 43507.37(43423.08,43591.79) | 18551.88(18514.65,18589.17) | -3.26(-3.58,-2.95) |
| Cabo Verde | both | 56545.26(47353.49,66161.48) | 9020.33(6904.09,11593.12) | -84.05 | 35589.10(35280.32,35899.99) | 6338.37(6200.33,6478.96) | -5.66(-5.84,-5.48) |
| Cambodia | both | 2378221.93(2108738.36,2646788.60) | 658741.45(534869.26,825326.29) | -72.30 | 48707.35(48640.63,48774.15) | 12881.53(12848.08,12915.05) | -4.38(-4.45,-4.31) |
| Cameroon | both | 3532280.03(3335494.88,3726678.34) | 2933246.48(2299749.94,3592452.51) | -16.96 | 72123.57(72044.24,72202.97) | 21670.89(21644.76,21697.04) | -3.70(-3.96,-3.44) |
| Canada | both | 259881.60(204166.04,330582.13) | 96437.88(74815.23,121276.47) | -62.89 | 4499.40(4481.03,4517.83) | 1540.76(1530.51,1551.06) | -3.14(-3.35,-2.92) |
| Central African Republic | both | 709166.61(645487.90,779824.85) | 959035.76(829505.34,1100698.69) | 35.23 | 56049.93(55909.90,56190.24) | 41012.81(40924.68,41101.10) | -0.98(-1.04,-0.93) |
| Chad | both | 2515808.69(2381203.53,2660717.98) | 4589760.01(3986506.91,5213535.30) | 82.44 | 82870.07(82760.05,82980.20) | 48576.28(48528.32,48624.28) | -1.78(-1.86,-1.70) |
| Chile | both | 522297.12(407144.07,655881.47) | 167073.51(123543.22,225464.10) | -68.01 | 13114.66(13077.18,13152.22) | 4613.93(4590.63,4637.33) | -3.23(-3.46,-2.99) |
| China | both | 59545747.41(49943077.71,70103143.68) | 9749461.84(7927764.16,12054996.57) | -83.63 | 18523.26(18518.27,18528.26) | 3779.81(3777.31,3782.32) | -4.89(-5.02,-4.77) |
| Colombia | both | 1106091.74(877256.42,1351262.20) | 312676.51(245429.52,397907.71) | -71.73 | 9387.58(9368.95,9406.24) | 2948.70(2937.74,2959.71) | -3.74(-3.82,-3.66) |
| Comoros | both | 99123.59(87201.40,112752.07) | 37530.53(29871.27,46322.21) | -62.14 | 45091.96(44790.38,45395.14) | 15512.38(15343.99,15682.25) | -3.58(-3.67,-3.48) |
| Congo | both | 616297.56(556220.78,680108.47) | 797400.52(677904.70,931740.94) | 29.39 | 57893.38(57740.39,58046.69) | 41493.50(41397.07,41590.12) | -0.97(-1.22,-0.72) |
| Cook Islands | both | 1317.46(1053.79,1656.61) | 218.78(166.97,283.05) | -83.39 | 19975.64(18857.64,21146.41) | 5802.63(5011.95,6694.84) | -3.86(-4.01,-3.72) |
| Costa Rica | both | 95209.81(75924.95,120140.70) | 36396.25(29070.57,44709.96) | -61.77 | 8412.27(8355.87,8468.97) | 3555.11(3516.91,3593.67) | -2.30(-2.54,-2.05) |
| Croatia | both | 150063.51(118373.91,188329.08) | 43359.34(33612.82,54584.85) | -71.11 | 15358.57(15276.24,15441.26) | 7329.53(7256.39,7403.29) | -2.72(-2.92,-2.52) |
| Cuba | both | 142435.54(108746.14,178572.77) | 47094.20(36401.35,59923.16) | -66.94 | 5582.41(5551.79,5613.16) | 2695.53(2669.83,2721.43) | -2.40(-2.50,-2.29) |
| Cyprus | both | 11411.19(8887.39,14719.14) | 3243.43(2637.73,3987.04) | -71.58 | 5763.84(5651.25,5878.28) | 1467.75(1414.93,1522.15) | -4.00(-4.42,-3.57) |
| Czechia | both | 289752.68(219728.17,368365.97) | 91402.94(71880.46,115792.10) | -68.45 | 13263.26(13211.62,13315.07) | 5337.97(5301.57,5374.58) | -2.61(-2.82,-2.41) |
| C?te d'Ivoire | both | 2974816.83(2672834.05,3272445.23) | 2286415.87(1853721.16,2789996.22) | -23.14 | 50862.89(50801.43,50924.41) | 19297.61(19270.90,19324.35) | -3.01(-3.10,-2.91) |
| Democratic People's Republic of Korea | both | 1281489.71(1050200.51,1520029.18) | 503619.46(387924.91,636268.06) | -60.70 | 20372.73(20334.26,20411.25) | 10774.19(10742.23,10806.22) | -2.68(-3.00,-2.36) |
| Democratic Republic of the Congo | both | 8562789.14(7505051.94,9674274.79) | 12620894.73(10630669.58,14862292.99) | 47.39 | 46348.80(46315.32,46382.30) | 32899.59(32880.20,32918.99) | -0.97(-1.49,-0.44) |
| Denmark | both | 19629.77(15633.39,24001.53) | 8771.91(6953.78,10764.37) | -55.31 | 2220.66(2187.52,2254.21) | 912.29(892.23,932.72) | -2.38(-2.64,-2.12) |
| Djibouti | both | 66499.18(56268.31,76713.72) | 69632.29(56744.56,83935.74) | 4.71 | 36861.40(36559.59,37165.19) | 16321.90(16190.60,16454.04) | -2.74(-2.94,-2.55) |
| Dominica | both | 1806.51(1405.53,2292.69) | 313.30(251.48,400.20) | -82.66 | 7252.19(6901.79,7616.79) | 2389.87(2108.15,2702.83) | -3.38(-3.56,-3.20) |
| Dominican Republic | both | 447560.98(373056.83,526169.61) | 153083.81(117600.29,197417.87) | -65.80 | 16466.30(16415.09,16517.64) | 5184.55(5157.19,5212.03) | -3.89(-3.99,-3.79) |
| Ecuador | both | 471075.55(384257.12,558150.72) | 252082.94(194142.38,313071.79) | -46.49 | 12104.47(12067.19,12141.84) | 4991.70(4970.94,5012.54) | -2.97(-3.22,-2.71) |
| Egypt | both | 2457648.52(2126567.75,2912174.17) | 1374238.23(1121046.04,1654873.13) | -44.08 | 10616.34(10601.98,10630.72) | 3617.50(3611.00,3624.01) | -2.64(-2.99,-2.29) |
| El Salvador | both | 500121.40(422183.24,607987.31) | 112976.21(86918.05,145231.21) | -77.41 | 23064.27(22995.89,23132.82) | 6233.26(6194.87,6271.85) | -4.29(-4.62,-3.95) |
| Equatorial Guinea | both | 121129.55(109649.93,133313.90) | 46150.31(35282.98,57810.63) | -61.90 | 58864.63(58507.95,59222.99) | 8007.40(7929.63,8085.78) | -7.00(-7.25,-6.75) |
| Eritrea | both | 968917.35(859855.90,1080244.01) | 601940.90(500881.34,720963.74) | -37.87 | 58301.19(58176.09,58426.50) | 22897.28(22834.44,22960.26) | -2.98(-3.15,-2.82) |
| Estonia | both | 8662.30(6911.48,10945.82) | 2086.92(1680.20,2613.87) | -75.91 | 2498.19(2442.66,2554.72) | 982.21(938.03,1028.06) | -2.94(-3.08,-2.80) |
| Eswatini | both | 136743.72(116146.84,157981.03) | 50301.41(39141.69,63030.05) | -63.21 | 34782.61(34586.30,34979.80) | 12180.96(12067.65,12295.12) | -3.34(-3.41,-3.28) |
| Ethiopia | both | 15351629.92(14031098.55,16709693.74) | 8820694.96(7001769.22,10704573.49) | -42.54 | 60848.46(60815.80,60881.13) | 19613.49(19599.58,19627.41) | -3.99(-4.36,-3.62) |
| Fiji | both | 64082.29(51867.81,77745.75) | 27439.86(21353.38,35296.35) | -57.18 | 22664.04(22478.79,22850.53) | 9941.64(9817.35,10067.20) | -2.45(-2.57,-2.34) |
| Finland | both | 28877.57(23041.55,35876.66) | 10910.94(8710.25,13622.19) | -62.22 | 2996.53(2959.84,3033.60) | 1300.57(1274.69,1326.90) | -2.36(-2.51,-2.21) |
| France | both | 134758.19(107728.05,164251.61) | 91260.13(72781.24,115257.17) | -32.28 | 1151.15(1144.67,1157.66) | 776.48(771.19,781.81) | -0.66(-0.88,-0.43) |
| Gabon | both | 94779.68(76891.88,113822.46) | 54364.02(40959.69,69609.27) | -42.64 | 22663.06(22509.27,22817.67) | 8544.07(8468.07,8620.61) | -2.84(-3.07,-2.61) |
| Gambia | both | 282149.72(257855.90,308708.55) | 268447.41(218843.88,325329.49) | -4.86 | 59441.67(59207.75,59676.32) | 26622.25(26515.07,26729.77) | -2.55(-2.59,-2.50) |
| Georgia | both | 43529.31(33852.71,55584.46) | 16548.88(12784.71,20913.95) | -61.98 | 3191.82(3160.19,3223.69) | 2245.04(2209.13,2281.43) | -1.22(-1.81,-0.62) |
| Germany | both | 169607.68(131643.74,211407.99) | 93185.44(70026.41,118395.70) | -45.06 | 1293.07(1286.60,1299.57) | 766.16(761.04,771.30) | -1.45(-1.64,-1.27) |
| Ghana | both | 3990620.26(3617832.82,4362612.55) | 3239465.69(2653636.02,3901228.92) | -18.82 | 57712.08(57651.67,57772.55) | 24759.82(24731.18,24788.49) | -2.79(-2.94,-2.64) |
| Greece | both | 87732.11(67556.79,112347.95) | 28694.09(22639.36,35374.48) | -67.29 | 4490.82(4458.67,4523.18) | 2055.94(2030.80,2081.35) | -2.14(-2.53,-1.74) |
| Greenland | both | 332.85(269.43,415.48) | 150.28(119.49,189.46) | -54.85 | 2235.61(1985.85,2509.27) | 1262.63(1058.55,1496.81) | -2.39(-2.74,-2.05) |
| Grenada | both | 5122.12(4044.57,6384.44) | 866.54(683.78,1121.09) | -83.08 | 15238.82(14801.73,15686.10) | 4000.65(3721.39,4296.85) | -4.11(-4.38,-3.84) |
| Guam | both | 4428.80(3544.25,5553.69) | 1678.15(1308.40,2129.27) | -62.11 | 10538.70(10213.05,10872.48) | 4561.72(4334.21,4798.76) | -2.65(-2.81,-2.49) |
| Guatemala | both | 763169.03(645982.85,892496.93) | 238765.10(184781.50,312767.54) | -68.71 | 18055.25(18011.17,18099.40) | 4943.43(4922.08,4964.86) | -4.13(-4.30,-3.95) |
| Guinea | both | 1933841.73(1778594.39,2092561.58) | 1845728.78(1518135.96,2183326.69) | -4.56 | 67984.74(67882.03,68087.57) | 29747.36(29701.41,29793.38) | -2.60(-2.68,-2.52) |
| Guinea-Bissau | both | 319536.34(288452.22,343812.98) | 277284.28(229278.69,325577.38) | -13.22 | 64698.10(64459.57,64937.32) | 30351.07(30231.01,30471.51) | -2.35(-2.41,-2.28) |
| Guyana | both | 51022.74(41930.37,61808.37) | 11067.66(8859.34,13633.39) | -78.31 | 16843.95(16687.03,17002.04) | 5129.90(5028.30,5233.13) | -3.54(-3.63,-3.44) |
| Haiti | both | 970829.35(836793.41,1117179.43) | 704760.06(567143.16,866368.67) | -27.41 | 34448.61(34374.75,34522.59) | 15894.06(15854.44,15933.77) | -2.71(-2.79,-2.63) |
| Honduras | both | 326465.27(269166.20,393385.55) | 166924.81(127673.39,215791.68) | -48.87 | 14403.52(14350.62,14456.57) | 5096.72(5070.72,5122.82) | -3.24(-3.32,-3.16) |
| Hungary | both | 330827.94(253366.19,407395.09) | 97071.46(73515.46,123070.61) | -70.66 | 15764.03(15706.35,15821.89) | 7020.80(6974.31,7067.56) | -2.63(-2.76,-2.51) |
| Iceland | both | 1683.52(1322.75,2059.15) | 735.72(591.36,926.38) | -56.30 | 2624.22(2491.56,2762.66) | 1077.79(997.05,1163.90) | -2.53(-2.70,-2.37) |
| India | both | 141742823.03(125881363.35,158346867.02) | 52735986.64(43693300.82,64699966.49) | -62.79 | 42536.51(42528.95,42544.08) | 15113.52(15109.07,15117.97) | -3.27(-3.64,-2.90) |
| Indonesia | both | 27312593.06(23750903.50,31134725.90) | 7255891.97(5927987.20,9014680.01) | -73.43 | 40670.16(40653.69,40686.64) | 10879.44(10870.86,10888.02) | -4.10(-4.18,-4.02) |
| Iran (Islamic Republic of) | both | 3292359.86(2672025.64,4072452.99) | 620332.23(497463.09,755282.12) | -81.16 | 13004.15(12989.21,13019.09) | 3156.32(3147.91,3164.75) | -3.93(-4.27,-3.59) |
| Iraq | both | 1490532.46(1221568.27,1804836.06) | 692169.33(566750.07,840569.91) | -53.56 | 17446.44(17416.30,17476.61) | 5208.74(5195.55,5221.95) | -3.86(-4.32,-3.39) |
| Ireland | both | 32474.89(25684.78,40565.14) | 11163.62(8400.01,14451.55) | -65.62 | 3396.47(3357.06,3436.27) | 1118.80(1097.03,1140.94) | -3.12(-3.39,-2.86) |
| Israel | both | 271839.47(216264.64,336333.67) | 171130.38(130009.22,218307.69) | -37.05 | 17757.90(17687.62,17828.40) | 6459.31(6427.07,6491.69) | -2.99(-3.24,-2.74) |
| Italy | both | 453405.65(365808.77,570528.16) | 154380.91(121569.91,188403.90) | -65.95 | 4989.26(4973.74,5004.82) | 2041.60(2030.79,2052.46) | -2.25(-2.54,-1.96) |
| Jamaica | both | 71595.55(57419.21,90000.93) | 19285.27(14979.16,25041.84) | -73.06 | 8543.23(8476.91,8609.98) | 3352.79(3302.14,3404.09) | -2.88(-3.00,-2.77) |
| Japan | both | 708720.17(577325.31,861281.38) | 260525.58(209229.97,316931.95) | -63.24 | 3169.84(3161.90,3177.79) | 1728.62(1721.49,1735.77) | -1.53(-1.72,-1.35) |
| Jordan | both | 253847.07(208050.44,306638.60) | 214212.32(168275.39,274401.41) | -15.61 | 15343.05(15280.39,15405.92) | 6002.37(5975.32,6029.53) | -3.12(-3.63,-2.60) |
| Kazakhstan | both | 1162203.19(957795.57,1401210.11) | 560258.84(435558.23,707867.57) | -51.79 | 22106.98(22064.41,22149.62) | 10209.42(10181.07,10237.83) | -2.55(-2.75,-2.36) |
| Kenya | both | 7135292.27(6429004.60,7787375.70) | 5479717.92(4392234.09,6679085.79) | -23.20 | 62989.82(62940.84,63038.83) | 29739.03(29712.39,29765.69) | -2.31(-2.58,-2.03) |
| Kiribati | both | 15525.01(13766.57,17303.15) | 13851.67(11399.91,16272.75) | -10.78 | 50999.79(50147.52,51863.30) | 32933.23(32356.03,33518.74) | -1.31(-1.37,-1.26) |
| Kuwait | both | 21985.86(17896.34,26657.92) | 11684.83(9357.54,14056.41) | -46.85 | 3875.15(3820.86,3930.04) | 1405.85(1378.76,1433.37) | -3.53(-3.66,-3.39) |
| Kyrgyzstan | both | 128305.31(104031.52,158674.10) | 119510.06(96663.28,152154.36) | -6.85 | 7407.00(7363.72,7450.47) | 5235.41(5204.02,5266.96) | -0.87(-1.16,-0.58) |
| Lao People's Democratic Republic | both | 1044494.53(943761.59,1143632.91) | 391569.16(309515.28,483722.07) | -62.51 | 55151.98(55038.83,55265.32) | 16833.96(16777.26,16890.80) | -4.08(-4.27,-3.89) |
| Latvia | both | 14154.76(11674.44,17094.35) | 3201.83(2566.18,3886.10) | -77.38 | 2477.72(2434.62,2521.42) | 1103.97(1063.78,1145.38) | -2.54(-2.69,-2.38) |
| Lebanon | both | 90999.71(74513.79,110729.90) | 41122.81(34889.18,49217.96) | -54.81 | 8471.32(8412.44,8530.53) | 3290.24(3256.36,3324.41) | -2.94(-3.26,-2.62) |
| Lesotho | both | 341370.18(299803.55,387348.31) | 128289.23(104530.30,156806.84) | -62.42 | 49580.81(49404.04,49758.09) | 20573.85(20453.21,20695.07) | -2.87(-2.91,-2.82) |
| Liberia | both | 548729.68(487117.39,611484.24) | 420474.32(329493.14,522653.48) | -23.37 | 46330.47(46198.14,46463.10) | 18912.70(18851.39,18974.17) | -3.13(-3.30,-2.97) |
| Libya | both | 165492.73(135868.10,201458.56) | 56324.87(46706.21,68322.04) | -65.97 | 8985.62(8939.13,9032.31) | 4002.53(3966.38,4038.96) | -2.04(-2.29,-1.78) |
| Lithuania | both | 21828.48(17676.96,26155.02) | 3819.20(3032.83,4708.18) | -82.50 | 2617.40(2580.58,2654.64) | 953.40(921.67,986.01) | -3.25(-3.35,-3.15) |
| Luxembourg | both | 1627.98(1315.15,1979.30) | 1027.33(825.42,1272.34) | -36.90 | 2420.18(2296.33,2549.40) | 1009.61(945.52,1077.24) | -2.43(-2.61,-2.24) |
| Madagascar | both | 2348592.08(2044687.67,2672217.01) | 2713553.93(2273670.54,3223725.48) | 15.54 | 41178.52(41121.47,41235.63) | 22655.43(22626.13,22684.75) | -2.01(-2.25,-1.77) |
| Malawi | both | 2570633.15(2314507.55,2828481.20) | 1677942.92(1372016.90,2042734.17) | -34.73 | 54239.33(54167.79,54310.95) | 20669.55(20636.10,20703.05) | -3.33(-3.57,-3.09) |
| Malaysia | both | 662748.58(559059.12,780265.91) | 233462.85(187341.40,284765.30) | -64.77 | 9510.39(9485.19,9535.63) | 3035.02(3021.37,3048.72) | -3.29(-3.49,-3.08) |
| Maldives | both | 49006.98(42663.13,55237.69) | 5612.94(4614.24,6787.52) | -88.55 | 44357.02(43935.31,44781.85) | 5793.38(5631.74,5958.72) | -6.44(-6.95,-5.92) |
| Mali | both | 3386132.72(3162809.25,3590711.11) | 4615648.91(3924485.46,5333705.60) | 36.31 | 79061.11(78970.71,79151.58) | 38378.59(38341.12,38416.10) | -2.37(-2.43,-2.31) |
| Malta | both | 4818.68(3740.78,6001.32) | 1166.48(899.88,1462.13) | -75.79 | 5545.85(5380.15,5715.68) | 1806.81(1699.25,1919.78) | -3.23(-3.46,-3.01) |
| Marshall Islands | both | 10537.40(9080.43,12057.09) | 4639.03(3679.13,5595.13) | -55.98 | 48115.09(47151.85,49094.16) | 26736.90(25929.67,27564.62) | -1.79(-1.86,-1.71) |
| Mauritania | both | 441811.31(392308.30,497549.21) | 257384.12(208477.61,312218.91) | -41.74 | 45476.45(45331.72,45621.54) | 13651.85(13595.55,13708.34) | -3.63(-3.72,-3.54) |
| Mauritius | both | 49034.11(41238.89,59224.64) | 9073.67(7367.49,10996.12) | -81.50 | 15070.19(14925.63,15215.89) | 4457.24(4357.54,4558.78) | -3.86(-3.99,-3.74) |
| Mexico | both | 8071132.95(7037562.60,9253347.03) | 2829795.88(2246756.29,3617159.27) | -64.94 | 24235.18(24217.49,24252.89) | 8993.69(8982.54,9004.85) | -3.08(-3.24,-2.93) |
| Micronesia (Federated States of) | both | 25723.98(22724.86,28491.66) | 12203.41(10185.88,14346.06) | -52.56 | 55680.34(54966.39,56401.78) | 39978.52(39229.37,40739.54) | -0.95(-1.01,-0.89) |
| Monaco | both | 52.34(41.82,63.68) | 44.04(31.96,59.07) | -15.87 | 1461.11(1071.24,1960.39) | 875.25(626.64,1198.87) | -1.17(-1.34,-1.00) |
| Mongolia | both | 76303.91(61432.49,94544.05) | 25978.73(20676.21,32593.20) | -65.95 | 8099.61(8037.11,8162.48) | 2354.44(2324.10,2385.10) | -4.46(-4.62,-4.30) |
| Montenegro | both | 21950.01(17141.07,27615.18) | 7641.63(5923.36,9734.33) | -65.19 | 13661.26(13471.76,13852.93) | 6890.43(6728.27,7055.78) | -2.79(-3.22,-2.36) |
| Morocco | both | 2462704.84(2054886.88,2907180.27) | 793728.23(623441.12,1015204.87) | -67.77 | 24874.85(24841.61,24908.12) | 8129.36(8110.43,8148.34) | -3.45(-3.79,-3.10) |
| Mozambique | both | 4088847.45(3735040.23,4456444.00) | 3909913.83(3190644.12,4670598.08) | -4.38 | 64216.64(64150.08,64283.25) | 27015.49(26986.97,27044.03) | -2.91(-3.08,-2.75) |
| Myanmar | both | 6878596.29(5898292.87,7847708.71) | 1580210.57(1251744.49,1969893.22) | -77.03 | 46471.06(46433.81,46508.33) | 10165.01(10147.97,10182.08) | -5.15(-5.29,-5.01) |
| Namibia | both | 151936.41(127983.45,180877.57) | 83907.31(66553.83,105329.28) | -44.77 | 24587.67(24455.40,24720.50) | 10043.31(9970.44,10116.61) | -3.04(-3.23,-2.85) |
| Nauru | both | 967.65(787.69,1171.10) | 626.96(486.10,787.81) | -35.21 | 22660.21(21178.20,24221.73) | 15767.43(14498.55,17123.90) | -0.98(-1.55,-0.40) |
| Nepal | both | 2905191.25(2540110.67,3285990.18) | 635380.62(509629.18,804353.12) | -78.13 | 32278.49(32237.90,32319.11) | 6827.92(6809.80,6846.08) | -5.04(-5.10,-4.98) |
| Netherlands | both | 63750.96(51603.47,79359.74) | 29587.20(22303.94,38781.52) | -53.59 | 2311.02(2292.13,2330.04) | 1088.71(1075.83,1101.72) | -1.82(-2.04,-1.59) |
| New Zealand | both | 11996.31(9393.53,14780.65) | 8016.90(6515.56,9759.72) | -33.17 | 1458.83(1431.36,1486.73) | 811.43(792.82,830.41) | -1.16(-1.49,-0.82) |
| Nicaragua | both | 235747.11(193032.88,281477.77) | 39505.45(31217.12,49135.28) | -83.24 | 12674.08(12618.68,12729.66) | 1975.09(1954.55,1995.81) | -6.32(-6.95,-5.68) |
| Niger | both | 3819956.45(3645161.97,3964268.31) | 8268668.04(7505554.52,9038968.54) | 116.46 | 90693.94(90596.16,90791.80) | 62736.59(62690.81,62782.40) | -1.17(-1.22,-1.11) |
| Nigeria | both | 9578574.96(8248241.23,11025825.82) | 8312796.30(6652251.19,10116259.84) | -13.21 | 22789.35(22773.50,22805.21) | 7859.70(7853.87,7865.54) | -3.53(-3.89,-3.17) |
| Niue | both | 149.01(117.02,182.86) | 33.89(26.58,42.73) | -77.26 | 18573.33(15557.90,22044.41) | 8786.59(5929.07,12677.39) | -2.26(-2.39,-2.13) |
| North Macedonia | both | 160682.81(129739.59,191637.39) | 44993.23(34991.96,57201.61) | -72.00 | 30566.39(30408.98,30724.47) | 13746.66(13612.39,13882.02) | -2.90(-3.05,-2.76) |
| Northern Mariana Islands | both | 1066.69(851.62,1328.09) | 691.61(521.80,869.08) | -35.16 | 8653.89(8110.10,9226.22) | 6189.87(5705.95,6707.89) | -0.45(-0.70,-0.20) |
| Norway | both | 20681.11(16865.22,24866.82) | 9255.16(7103.94,11961.30) | -55.25 | 2551.59(2514.89,2588.72) | 994.86(973.69,1016.41) | -2.33(-2.61,-2.04) |
| Oman | both | 133151.48(109292.65,162027.23) | 44115.71(36539.59,52781.92) | -66.87 | 15217.20(15129.28,15305.50) | 3567.01(3531.34,3602.96) | -4.17(-4.50,-3.85) |
| Pakistan | both | 14263401.45(12329516.55,16358673.77) | 5462358.47(4443679.88,6560057.59) | -61.70 | 27751.13(27735.36,27766.90) | 6152.80(6147.12,6158.48) | -4.24(-4.53,-3.96) |
| Palau | both | 557.73(434.36,700.66) | 234.40(175.06,299.45) | -57.97 | 12271.29(11215.13,13407.68) | 7251.33(6292.56,8329.11) | -1.36(-1.53,-1.18) |
| Palestine | both | 220804.81(181215.71,269591.75) | 96722.46(75140.99,123982.83) | -56.20 | 22160.74(22062.33,22259.48) | 5188.13(5153.48,5222.97) | -4.30(-4.51,-4.09) |
| Panama | both | 62378.12(50265.03,78131.77) | 32967.42(25621.32,42058.11) | -47.15 | 7432.85(7371.01,7495.10) | 2851.93(2819.20,2884.96) | -2.91(-3.26,-2.57) |
| Papua New Guinea | both | 362858.99(304771.62,438238.24) | 557443.89(463748.66,684209.04) | 53.63 | 20302.87(20231.28,20374.66) | 13233.55(13195.65,13271.54) | -0.82(-1.07,-0.56) |
| Paraguay | both | 340173.90(270502.37,426222.34) | 141830.47(108729.70,182770.15) | -58.31 | 20275.96(20204.30,20347.83) | 7067.67(7029.03,7106.48) | -3.25(-3.31,-3.19) |
| Peru | both | 1301146.76(1080088.02,1533366.87) | 560541.27(439162.95,712283.76) | -56.92 | 15645.46(15616.82,15674.14) | 5884.37(5868.18,5900.59) | -3.65(-3.94,-3.36) |
| Philippines | both | 6880596.44(6007480.96,7968933.53) | 4337982.56(3502132.69,5240343.18) | -36.95 | 26643.99(26622.56,26665.43) | 12772.06(12759.07,12785.07) | -2.05(-2.36,-1.74) |
| Poland | both | 1723003.25(1376581.24,2145055.25) | 354742.01(275101.59,447535.08) | -79.41 | 18208.65(18179.86,18237.48) | 6068.66(6047.63,6089.75) | -3.47(-3.53,-3.42) |
| Portugal | both | 144759.65(111883.64,184157.62) | 32784.61(25409.11,41050.43) | -77.35 | 7129.64(7089.71,7169.75) | 2427.18(2399.33,2455.30) | -2.99(-3.28,-2.69) |
| Puerto Rico | both | 43068.38(33547.29,55274.10) | 6167.94(5000.05,7471.54) | -85.68 | 4327.21(4283.80,4370.99) | 1446.19(1406.25,1487.11) | -3.39(-3.50,-3.27) |
| Qatar | both | 5670.26(4631.17,6896.00) | 11686.58(9182.25,14609.10) | 106.10 | 4272.57(4154.02,4393.73) | 2337.03(2293.17,2381.57) | -1.35(-1.59,-1.11) |
| Republic of Korea | both | 450749.45(357022.34,563161.25) | 63090.75(50485.97,77977.78) | -86.00 | 3996.85(3984.27,4009.46) | 1051.59(1042.62,1060.62) | -3.94(-4.19,-3.70) |
| Republic of Moldova | both | 43628.56(35433.35,53055.39) | 8338.61(6675.86,10425.03) | -80.89 | 3504.81(3469.97,3539.91) | 1630.17(1592.78,1668.29) | -2.53(-2.78,-2.28) |
| Romania | both | 1080905.39(849297.67,1306226.86) | 247328.44(190004.07,319304.24) | -77.12 | 19580.93(19541.70,19620.23) | 8301.31(8266.56,8336.18) | -2.89(-3.06,-2.72) |
| Russian Federation | both | 704838.34(554866.66,860069.96) | 288980.86(222978.52,364285.66) | -59.00 | 2045.52(2040.34,2050.71) | 1138.28(1133.70,1142.88) | -1.73(-1.91,-1.55) |
| Rwanda | both | 1387335.68(1189052.31,1594132.13) | 777456.55(621840.12,943924.47) | -43.96 | 39308.76(39238.21,39379.42) | 15470.95(15434.05,15507.92) | -3.49(-3.86,-3.12) |
| Saint Kitts and Nevis | both | 1006.41(786.93,1277.32) | 230.55(187.84,282.88) | -77.09 | 7112.85(6654.57,7596.35) | 2343.59(2030.71,2694.63) | -3.36(-3.50,-3.23) |
| Saint Lucia | both | 5169.47(3974.75,6446.83) | 1119.89(875.97,1394.96) | -78.34 | 10025.32(9739.40,10317.97) | 3824.24(3588.15,4073.07) | -2.80(-2.97,-2.63) |
| Saint Vincent and the Grenadines | both | 5119.74(3990.14,6513.18) | 1124.54(869.21,1455.62) | -78.04 | 12557.00(12194.16,12928.72) | 4567.56(4286.56,4863.73) | -3.14(-3.28,-3.00) |
| Samoa | both | 22915.84(19076.96,27311.65) | 15898.14(12757.93,19545.56) | -30.62 | 31878.94(31445.56,32317.11) | 19732.49(19411.42,20057.79) | -1.46(-1.53,-1.39) |
| San Marino | both | 77.19(60.84,93.09) | 44.28(33.97,57.08) | -42.63 | 1934.36(1498.37,2470.73) | 1015.02(720.43,1405.16) | -1.53(-1.84,-1.22) |
| Sao Tome and Principe | both | 28550.20(25055.48,32090.32) | 11263.60(8939.68,14042.60) | -60.55 | 49959.47(49347.35,50577.66) | 14547.26(14262.66,14836.48) | -4.13(-4.37,-3.90) |
| Saudi Arabia | both | 430179.94(352466.03,505891.52) | 169471.60(135011.55,208848.16) | -60.60 | 6325.67(6305.13,6346.25) | 2312.41(2300.56,2324.30) | -2.57(-2.88,-2.25) |
| Senegal | both | 2111248.27(1933057.76,2301145.33) | 634894.08(494665.37,822219.75) | -69.93 | 56910.40(56829.04,56991.85) | 9893.81(9868.07,9919.61) | -5.05(-5.53,-4.57) |
| Serbia | both | 766099.58(659195.84,897273.53) | 189028.47(148359.33,239284.83) | -75.33 | 35655.88(35571.26,35740.67) | 14536.24(14465.75,14607.02) | -3.23(-3.49,-2.96) |
| Seychelles | both | 2579.70(2010.17,3244.90) | 741.82(601.99,912.66) | -71.24 | 10839.06(10400.35,11292.61) | 3137.64(2899.54,3391.12) | -3.44(-3.79,-3.10) |
| Sierra Leone | both | 1185763.10(1087715.60,1274321.27) | 1018988.69(838280.95,1205317.64) | -14.06 | 62939.45(62817.40,63061.67) | 27689.58(27631.95,27747.31) | -2.72(-2.95,-2.50) |
| Singapore | both | 35792.15(29718.04,42948.71) | 19247.82(15004.70,23588.12) | -46.22 | 5518.91(5456.75,5581.67) | 2330.67(2295.90,2365.86) | -2.84(-3.03,-2.64) |
| Slovakia | both | 212226.44(168367.02,263197.27) | 54368.94(42219.86,68672.44) | -74.38 | 16168.89(16096.01,16242.05) | 6366.10(6309.78,6422.83) | -3.02(-3.13,-2.92) |
| Slovenia | both | 41322.82(32308.28,51748.59) | 14592.01(11443.24,18777.41) | -64.69 | 10130.40(10026.74,10234.95) | 4704.81(4624.42,4786.34) | -2.55(-2.65,-2.44) |
| Solomon Islands | both | 91008.83(81542.87,100916.26) | 100577.06(83816.81,118934.38) | 10.51 | 57511.04(57115.36,57908.88) | 38336.17(38085.91,38587.73) | -1.09(-1.21,-0.96) |
| Somalia | both | 3759835.91(3650128.49,3869466.58) | 8870578.91(8408393.87,9275054.58) | 135.93 | 95305.63(95203.76,95407.59) | 84789.58(84730.60,84848.60) | -0.43(-0.46,-0.40) |
| South Africa | both | 3728956.17(3183926.01,4349501.13) | 1320054.35(1053666.39,1671693.06) | -64.60 | 27182.89(27153.66,27212.15) | 8794.58(8778.60,8810.59) | -3.76(-3.82,-3.71) |
| South Sudan | both | 1505864.74(1335271.60,1694572.53) | 1617096.56(1376750.76,1893347.03) | 7.39 | 55469.21(55373.94,55564.61) | 36829.63(36768.31,36891.02) | -1.61(-1.71,-1.50) |
| Spain | both | 258904.65(204319.58,330186.73) | 96699.05(79703.13,118354.57) | -62.65 | 3353.49(3339.49,3367.55) | 1505.35(1495.21,1515.55) | -2.01(-2.24,-1.78) |
| Sri Lanka | both | 1552568.72(1325015.10,1802678.74) | 410253.34(334398.22,492305.52) | -73.58 | 28555.72(28507.18,28604.33) | 8297.83(8270.16,8325.57) | -4.15(-4.27,-4.03) |
| Sudan | both | 3513727.72(3057807.77,3972493.41) | 2024692.65(1628665.38,2458227.86) | -42.38 | 37064.59(37022.13,37107.10) | 12027.71(12009.61,12045.83) | -3.52(-3.88,-3.16) |
| Suriname | both | 15417.75(12264.34,19309.40) | 7287.43(5666.32,9292.80) | -52.73 | 11858.43(11660.07,12059.52) | 5163.14(5037.31,5291.54) | -2.69(-2.74,-2.65) |
| Sweden | both | 42067.82(33402.77,51564.48) | 20784.99(16397.04,25637.27) | -50.59 | 2649.13(2622.36,2676.13) | 1136.76(1120.61,1153.11) | -2.04(-2.32,-1.76) |
| Switzerland | both | 16101.61(12604.60,20287.01) | 9764.56(7531.84,12756.34) | -39.36 | 1374.55(1352.19,1397.21) | 724.96(710.00,740.18) | -1.69(-1.84,-1.54) |
| Syrian Arab Republic | both | 1011403.18(828126.86,1209176.18) | 279113.68(225110.07,338005.04) | -72.40 | 16685.07(16650.00,16720.19) | 8304.03(8269.86,8338.31) | -2.04(-2.22,-1.87) |
| Taiwan (Province of China) | both | 315186.16(247235.76,397304.96) | 53836.30(43644.76,65716.87) | -82.92 | 5796.70(5774.90,5818.57) | 1791.60(1775.65,1807.68) | -3.21(-3.57,-2.84) |
| Tajikistan | both | 428827.57(360675.58,505138.91) | 446940.86(372815.78,539166.48) | 4.22 | 17370.52(17314.31,17426.88) | 11975.08(11937.33,12012.93) | -1.38(-1.81,-0.94) |
| Thailand | both | 4083185.00(3638554.55,4607033.22) | 354904.07(290761.56,427529.11) | -91.31 | 24292.28(24266.73,24317.86) | 3884.59(3870.50,3898.73) | -5.51(-5.78,-5.24) |
| Timor-Leste | both | 211020.05(189973.78,232641.16) | 115061.87(96671.97,136791.13) | -45.47 | 59335.16(59059.17,59612.17) | 21681.00(21543.67,21819.03) | -3.19(-3.27,-3.12) |
| Togo | both | 950490.74(854506.25,1050513.02) | 729321.84(590531.48,894577.04) | -23.27 | 52815.08(52702.17,52928.17) | 21762.50(21709.57,21815.54) | -2.73(-2.85,-2.61) |
| Tokelau | both | 160.84(129.93,195.91) | 44.52(33.69,58.21) | -72.32 | 26916.82(22674.42,31776.23) | 11642.42(8230.09,16180.25) | -2.65(-2.81,-2.48) |
| Tonga | both | 12234.05(10112.58,14641.69) | 6513.81(5295.77,8054.98) | -46.76 | 29180.97(28639.57,29730.49) | 16583.68(16162.97,17013.03) | -1.43(-1.58,-1.29) |
| Trinidad and Tobago | both | 23528.92(18546.78,29429.66) | 7208.67(5847.39,8884.38) | -69.36 | 5917.72(5838.38,5997.92) | 2794.88(2725.50,2865.69) | -2.32(-2.43,-2.22) |
| Tunisia | both | 310624.59(241498.28,398911.91) | 77242.08(61802.65,97940.41) | -75.13 | 10017.79(9980.19,10055.49) | 2806.54(2785.50,2827.70) | -4.20(-4.37,-4.03) |
| Türkiye | both | 3313160.84(2853902.31,3828333.99) | 672715.16(527906.11,843860.70) | -79.70 | 15925.56(15907.02,15944.11) | 3663.81(3654.48,3673.16) | -4.81(-5.02,-4.59) |
| Turkmenistan | both | 111352.13(89356.42,136641.50) | 61775.45(49428.25,77761.02) | -44.52 | 6970.04(6925.80,7014.49) | 3964.44(3931.17,3997.92) | -1.99(-2.43,-1.55) |
| Tuvalu | both | 1571.70(1364.37,1803.62) | 801.90(645.20,989.25) | -48.98 | 43873.78(41583.73,46261.38) | 21545.66(20002.74,23183.02) | -1.99(-2.10,-1.87) |
| Uganda | both | 3141804.26(2670925.74,3630985.25) | 2237968.25(1797287.91,2768466.92) | -28.77 | 35475.20(35432.71,35517.72) | 10975.98(10960.49,10991.49) | -3.75(-3.87,-3.63) |
| Ukraine | both | 516958.17(424304.72,628665.20) | 187684.82(153003.44,226349.64) | -63.69 | 4487.58(4474.31,4500.88) | 3307.38(3290.56,3324.27) | -0.38(-0.64,-0.11) |
| United Arab Emirates | both | 58405.98(48093.41,68524.03) | 90067.50(73933.94,109786.64) | 54.21 | 9374.52(9292.08,9457.54) | 6618.17(6573.11,6663.49) | -0.45(-0.74,-0.15) |
| United Kingdom | both | 379971.68(303287.97,470625.05) | 153383.22(122672.11,188395.41) | -59.63 | 3422.41(3410.91,3433.94) | 1311.31(1304.40,1318.26) | -2.35(-2.62,-2.08) |
| United Republic of Tanzania | both | 3689840.37(3146040.27,4206598.03) | 2773140.10(2211918.52,3405439.22) | -24.84 | 29067.02(29034.88,29099.19) | 11118.05(11103.95,11132.16) | -2.80(-3.02,-2.57) |
| United States of America | both | 1138133.68(919117.00,1393671.29) | 647763.73(518751.45,802896.89) | -43.09 | 2014.24(2010.37,2018.12) | 1071.09(1068.34,1073.84) | -2.09(-2.50,-1.68) |
| United States Virgin Islands | both | 1421.76(1111.20,1766.42) | 193.61(157.90,238.59) | -86.38 | 4430.24(4189.09,4682.48) | 1473.36(1257.40,1718.73) | -3.58(-3.76,-3.39) |
| Uruguay | both | 120658.56(94851.83,152017.33) | 45119.92(34346.41,59413.81) | -62.61 | 14953.04(14863.61,15042.90) | 7012.16(6943.67,7081.21) | -2.37(-2.59,-2.15) |
| Uzbekistan | both | 945407.85(762942.89,1148753.33) | 442352.66(353252.46,540412.37) | -53.21 | 10170.17(10147.83,10192.54) | 4223.74(4210.45,4237.05) | -3.03(-3.09,-2.96) |
| Vanuatu | both | 36993.70(32351.17,41104.62) | 43490.92(36082.96,51500.94) | 17.56 | 53303.17(52727.81,53883.40) | 37053.54(36686.45,37423.54) | -1.03(-1.14,-0.92) |
| Venezuela (Bolivarian Republic of) | both | 845919.28(704448.35,999275.73) | 534173.99(414378.99,683622.45) | -36.85 | 11546.61(11520.03,11573.25) | 8263.94(8240.49,8287.44) | -1.16(-1.68,-0.63) |
| Viet Nam | both | 5291979.24(4554068.58,6227482.13) | 1621270.62(1332018.39,2038150.91) | -69.36 | 19281.12(19263.39,19298.86) | 6596.05(6585.05,6607.05) | -3.14(-3.49,-2.79) |
| Yemen | both | 3734828.59(3300267.57,4149381.29) | 2563014.63(2098057.86,3155543.62) | -31.38 | 50679.05(50623.73,50734.41) | 18548.68(18524.02,18573.36) | -3.41(-3.54,-3.28) |
| Zambia | both | 1596818.89(1390860.11,1797919.20) | 1164405.55(948186.88,1432463.69) | -27.08 | 40556.63(40488.34,40625.00) | 13891.39(13864.18,13918.64) | -3.63(-4.06,-3.20) |
| Zimbabwe | both | 1646944.42(1383776.97,1918885.37) | 1466225.46(1199456.33,1779480.37) | -10.97 | 33708.75(33653.92,33763.65) | 22994.64(22954.94,23034.40) | -0.77(-1.03,-0.51) |
| Global | female | 194527378.11(181841048.78,207601950.36) | 100997786.45(93399033.55,108865560.94) | -48.08 | 22549.85(22546.44,22553.26) | 10424.44(10422.26,10426.63) | -2.43(-2.66,-2.19) |
| Central Europe, eastern Europe, and central Asia | female | 4746087.51(4322097.91,5220624.16) | 1748425.73(1574496.28,1945699.89) | -63.16 | 9084.90(9076.22,9093.58) | 4487.29(4480.18,4494.40) | -2.35(-2.52,-2.17) |
| High-income | female | 3455670.65(3100861.49,3830442.75) | 1572243.70(1372039.54,1794726.23) | -54.50 | 3825.13(3820.87,3829.39) | 1838.66(1835.63,1841.70) | -2.10(-2.23,-1.97) |
| Latin America and Caribbean | female | 13170665.48(11879691.61,14596401.78) | 5470437.12(4666816.27,6466313.42) | -58.46 | 18525.37(18514.78,18535.96) | 7820.89(7813.95,7827.83) | -2.80(-2.85,-2.74) |
| Southeast Asia, east Asia, and Oceania | female | 47695374.04(42097326.61,53172040.47) | 13363143.11(11715600.49,15564625.31) | -71.98 | 19528.45(19522.51,19534.38) | 6399.86(6396.19,6403.53) | -3.43(-3.59,-3.27) |
| Sub-Saharan Africa | female | 46572101.91(44684032.34,48628453.45) | 46156024.91(43493870.81,48861310.24) | -0.89 | 40081.00(40068.62,40093.38) | 19237.06(19231.12,19243.01) | -2.44(-2.64,-2.25) |
| Andean Latin America | female | 948404.35(801176.19,1112613.58) | 510321.56(417271.41,625351.54) | -46.19 | 12877.90(12850.25,12905.60) | 5810.78(5793.98,5827.63) | -2.83(-3.05,-2.61) |
| Australasia | female | 16676.90(12834.91,21455.97) | 13693.34(10632.46,17323.82) | -17.89 | 735.62(723.86,747.53) | 493.53(484.88,502.31) | -0.79(-1.04,-0.54) |
| Caribbean | female | 822085.59(711837.20,941865.69) | 473236.08(381635.88,593610.05) | -42.43 | 14280.87(14247.80,14314.00) | 8358.85(8333.43,8384.33) | -1.86(-1.92,-1.80) |
| Central Asia | female | 1293387.02(1104049.85,1477383.70) | 779763.57(669821.77,903176.08) | -39.71 | 9989.04(9970.52,10007.59) | 5717.83(5704.30,5731.38) | -1.81(-1.99,-1.64) |
| Central Europe | female | 2814882.73(2500890.01,3150023.28) | 740302.11(649551.72,843179.43) | -73.70 | 19685.68(19661.30,19710.09) | 8657.95(8637.14,8678.81) | -2.75(-2.87,-2.64) |
| Central Latin America | female | 4897652.42(4310043.40,5615949.45) | 1970135.53(1643001.51,2447412.35) | -59.77 | 15307.06(15292.64,15321.48) | 6406.22(6396.70,6415.75) | -2.65(-2.75,-2.55) |
| Central Sub-Saharan Africa | female | 5182008.76(4537531.14,5903664.42) | 7143214.33(5974265.72,8552448.04) | 37.85 | 39280.20(39243.62,39316.80) | 24382.64(24363.51,24401.79) | -1.46(-1.89,-1.02) |
| East Asia | female | 23036700.85(18320482.29,28074694.06) | 4977259.39(3830436.15,6674844.88) | -78.39 | 14506.76(14500.47,14513.05) | 4016.68(4012.96,4020.41) | -4.02(-4.14,-3.90) |
| Eastern Europe | female | 637817.76(520738.90,766578.02) | 228360.04(180335.41,281783.96) | -64.20 | 2518.92(2512.24,2525.61) | 1370.45(1364.25,1376.66) | -1.56(-1.77,-1.35) |
| Eastern Sub-Saharan Africa | female | 21476259.11(20299980.88,22651717.84) | 19091085.33(17492797.47,20811813.85) | -11.11 | 46527.01(46505.89,46548.14) | 21459.34(21449.06,21469.63) | -2.66(-2.84,-2.48) |
| High-income Asia Pacific | female | 625887.66(508964.34,770516.35) | 186601.11(151607.90,226207.70) | -70.19 | 3743.58(3733.60,3753.58) | 1751.25(1742.71,1759.83) | -2.07(-2.21,-1.92) |
| High-income North America | female | 814177.46(647822.55,1011787.86) | 410751.69(320848.60,515721.44) | -49.55 | 2699.72(2693.59,2705.86) | 1278.47(1274.35,1282.61) | -2.36(-2.58,-2.15) |
| North Africa and Middle East | female | 10843455.96(10049328.57,11750871.64) | 6222708.10(5640545.71,6921785.74) | -42.61 | 15406.55(15396.68,15416.42) | 6995.13(6989.21,7001.06) | -2.45(-2.70,-2.19) |
| Oceania | female | 290762.71(252797.69,330735.65) | 374431.61(310313.99,448372.95) | 28.78 | 21955.94(21870.47,22041.67) | 14636.85(14586.25,14687.59) | -0.93(-1.10,-0.75) |
| South Asia | female | 68044022.56(59705087.24,77034660.32) | 26464803.78(21198368.15,33018837.03) | -61.11 | 31503.17(31495.02,31511.32) | 11254.96(11250.30,11259.62) | -3.18(-3.50,-2.87) |
| Southeast Asia | female | 24367910.48(22038148.48,26698068.42) | 8011452.11(6914771.31,9383674.28) | -67.12 | 29054.13(29041.68,29066.59) | 9623.83(9616.63,9631.03) | -3.39(-3.49,-3.29) |
| Southern Latin America | female | 999294.14(817619.03,1239370.51) | 490565.87(371777.50,642767.03) | -50.91 | 14004.31(13975.48,14033.19) | 7051.75(7030.90,7072.64) | -2.11(-2.28,-1.94) |
| Southern Sub-Saharan Africa | female | 2619374.29(2263628.70,3039863.61) | 1457414.67(1218273.67,1759148.09) | -44.36 | 25018.70(24986.49,25050.94) | 12256.77(12235.56,12278.01) | -2.17(-2.27,-2.08) |
| Tropical Latin America | female | 6502523.12(5453249.68,7974139.71) | 2516743.96(1847849.17,3410748.95) | -61.30 | 24759.81(24739.72,24779.91) | 10229.92(10216.62,10243.23) | -2.94(-3.01,-2.87) |
| Western Europe | female | 999634.48(892240.18,1120608.94) | 470631.69(401224.26,548475.69) | -52.92 | 2896.11(2890.11,2902.12) | 1414.00(1409.76,1418.25) | -1.70(-1.93,-1.46) |
| Western Sub-Saharan Africa | female | 17294459.75(16251637.65,18386155.73) | 18464310.58(17022955.81,20189678.16) | 6.76 | 37305.71(37286.74,37324.68) | 16765.80(16757.57,16774.03) | -2.66(-2.79,-2.53) |
| Afghanistan | female | 989259.90(835769.86,1151193.85) | 1638612.03(1288149.33,2014937.89) | 65.64 | 45241.48(45145.17,45337.96) | 23198.46(23160.10,23236.87) | -2.28(-2.69,-1.87) |
| Albania | female | 167805.56(135027.14,204666.81) | 32372.89(24600.15,42730.58) | -80.71 | 31387.95(31228.53,31548.02) | 15611.48(15432.00,15792.66) | -2.68(-2.91,-2.45) |
| Algeria | female | 571833.56(443256.16,722060.69) | 283832.00(218642.57,370451.23) | -50.36 | 10833.06(10802.93,10863.26) | 4300.47(4283.59,4317.39) | -3.11(-3.18,-3.04) |
| American Samoa | female | 1390.42(1063.08,1791.04) | 605.96(435.31,822.33) | -56.42 | 15030.41(14202.81,15894.97) | 9043.59(8281.41,9864.62) | -1.44(-1.66,-1.22) |
| Andorra | female | 118.12(89.93,154.83) | 64.49(45.09,91.24) | -45.40 | 2668.15(2172.67,3254.91) | 1275.97(961.70,1677.86) | -2.06(-2.18,-1.95) |
| Angola | female | 1113315.67(932746.77,1286599.52) | 1535352.92(1115311.16,2012070.73) | 37.91 | 44491.54(44401.55,44581.68) | 19746.38(19712.91,19779.90) | -2.70(-2.91,-2.50) |
| Antigua and Barbuda | female | 618.14(474.77,781.51) | 260.91(206.52,344.86) | -57.79 | 6819.59(6255.61,7423.60) | 3145.64(2749.87,3586.92) | -2.19(-2.34,-2.03) |
| Argentina | female | 743631.82(570177.96,976179.81) | 395494.59(278072.19,552230.10) | -46.82 | 15497.38(15460.44,15534.39) | 8102.93(8076.24,8129.70) | -2.03(-2.19,-1.87) |
| Armenia | female | 9971.63(7734.99,12582.49) | 4397.43(3472.42,5438.97) | -55.90 | 1887.52(1848.34,1927.36) | 1713.46(1660.65,1767.61) | 0.05(-0.13,0.23) |
| Australia | female | 10381.77(7662.42,13732.16) | 9474.76(7148.39,12461.01) | -8.74 | 555.77(544.49,567.24) | 412.44(403.75,421.29) | -0.59(-0.80,-0.39) |
| Austria | female | 19011.59(14209.05,25456.37) | 9766.05(7053.11,14246.24) | -48.63 | 2900.51(2856.99,2944.57) | 1545.33(1513.62,1577.58) | -1.78(-2.04,-1.52) |
| Azerbaijan | female | 70740.84(53884.57,91186.12) | 43632.33(33985.35,55060.06) | -38.32 | 5711.60(5666.61,5756.88) | 4077.00(4036.05,4118.29) | -1.04(-1.50,-0.58) |
| Bahamas | female | 1474.65(1061.08,1998.35) | 745.28(548.23,1012.90) | -49.46 | 3747.19(3546.08,3957.62) | 1929.67(1779.70,2090.19) | -1.93(-2.04,-1.82) |
| Bahrain | female | 5437.34(4383.92,7111.27) | 4388.46(3455.07,5665.93) | -19.29 | 6534.45(6348.58,6724.56) | 3037.83(2943.05,3135.10) | -2.03(-2.23,-1.83) |
| Bangladesh | female | 6220184.71(5095473.13,7357015.42) | 1397239.14(1091772.28,1771446.32) | -77.54 | 24235.36(24214.51,24256.23) | 6316.01(6304.56,6327.48) | -4.03(-4.24,-3.81) |
| Barbados | female | 1636.79(1209.04,2191.70) | 702.49(539.58,917.87) | -57.08 | 5318.83(5043.77,5606.11) | 3106.20(2861.15,3368.36) | -1.40(-1.53,-1.26) |
| Belarus | female | 29443.37(21163.61,40696.17) | 7635.37(5536.99,9943.98) | -74.07 | 2552.44(2522.20,2582.97) | 1049.86(1025.38,1074.82) | -2.77(-2.84,-2.69) |
| Belgium | female | 26027.10(19668.32,35317.04) | 11886.97(8940.54,15936.08) | -54.33 | 2955.33(2917.29,2993.78) | 1263.87(1240.11,1288.02) | -2.20(-2.42,-1.98) |
| Belize | female | 5153.06(3760.06,6980.20) | 3264.58(2377.49,4431.44) | -36.65 | 12705.86(12339.21,13081.08) | 5480.41(5281.60,5685.34) | -2.55(-2.63,-2.47) |
| Benin | female | 606621.02(510401.78,706382.34) | 791335.59(616985.45,1004944.63) | 30.45 | 49425.89(49291.86,49560.20) | 25539.52(25479.03,25600.12) | -2.08(-2.19,-1.96) |
| Bermuda | female | 221.97(158.46,297.04) | 73.71(54.63,95.83) | -66.79 | 3755.19(3252.62,4317.31) | 1789.80(1379.68,2294.77) | -2.47(-2.51,-2.43) |
| Bhutan | female | 38354.33(30697.07,48535.49) | 6495.13(4945.30,8624.04) | -83.07 | 29572.45(29253.27,29894.36) | 6974.74(6795.08,7158.27) | -4.75(-4.87,-4.63) |
| Bolivia (Plurinational State of) | female | 179436.99(133681.19,226014.32) | 115675.15(82845.40,154659.90) | -35.53 | 13309.36(13243.56,13375.41) | 6752.42(6711.39,6793.66) | -2.42(-2.63,-2.21) |
| Bosnia and Herzegovina | female | 140401.37(107566.79,179917.27) | 29722.31(21418.00,40700.95) | -78.83 | 26428.16(26282.56,26574.43) | 12600.31(12449.51,12752.62) | -2.90(-3.12,-2.68) |
| Botswana | female | 91207.13(73344.98,111092.72) | 40555.66(29597.72,53401.17) | -55.53 | 30219.50(30010.57,30429.60) | 11623.56(11502.96,11745.18) | -3.00(-3.07,-2.92) |
| Brazil | female | 6344016.87(5305212.20,7801931.27) | 2445412.78(1772974.32,3352719.35) | -61.45 | 24934.80(24914.31,24955.30) | 10350.51(10336.86,10364.17) | -2.93(-3.01,-2.86) |
| Brunei Darussalam | female | 2633.38(2080.40,3354.58) | 1272.43(1015.75,1629.25) | -51.68 | 5689.96(5457.24,5930.35) | 2707.64(2548.54,2874.72) | -2.12(-2.25,-2.00) |
| Bulgaria | female | 149931.79(110031.10,202806.15) | 54557.33(41357.35,70364.15) | -63.61 | 17669.29(17574.27,17764.74) | 11321.53(11221.32,11422.49) | -1.18(-1.36,-1.00) |
| Burkina Faso | female | 1544118.23(1349362.24,1735615.27) | 1379491.63(1063172.40,1763890.63) | -10.66 | 64008.53(63900.11,64117.09) | 25418.73(25373.04,25464.48) | -3.19(-3.32,-3.06) |
| Burundi | female | 555825.18(461518.81,655895.52) | 539935.03(407836.58,701210.99) | -2.86 | 40068.58(39954.42,40183.00) | 17795.64(17744.12,17847.27) | -3.12(-3.42,-2.81) |
| Cabo Verde | female | 19228.36(14807.60,24366.19) | 4167.45(2925.85,5582.40) | -78.33 | 24154.56(23793.93,24519.47) | 5949.85(5759.43,6145.47) | -4.59(-4.70,-4.48) |
| Cambodia | female | 1024666.35(845451.72,1208675.21) | 303171.05(226453.52,397195.75) | -70.41 | 42169.72(42081.51,42258.07) | 12133.09(12086.68,12179.65) | -4.10(-4.18,-4.03) |
| Cameroon | female | 1369209.14(1203595.11,1514995.85) | 1053541.26(739680.72,1378675.78) | -23.05 | 57061.69(56960.84,57162.67) | 15806.20(15774.20,15838.25) | -4.05(-4.16,-3.94) |
| Canada | female | 133530.08(96003.37,181527.29) | 50679.67(37085.22,68653.64) | -62.05 | 4763.16(4736.17,4790.27) | 1685.64(1670.18,1701.23) | -3.09(-3.34,-2.84) |
| Central African Republic | female | 263708.69(218725.53,311276.72) | 341872.63(275858.87,419078.89) | 29.64 | 41579.76(41408.09,41751.99) | 29529.74(29422.72,29637.05) | -1.09(-1.16,-1.02) |
| Chad | female | 1072523.45(948164.67,1185870.18) | 1765859.28(1417801.15,2143068.20) | 64.65 | 70370.80(70227.24,70514.58) | 37438.26(37378.36,37498.24) | -2.11(-2.21,-2.02) |
| Chile | female | 207549.59(151255.53,283052.27) | 74949.48(52279.49,103932.15) | -63.89 | 10592.37(10544.37,10640.54) | 4225.12(4193.33,4257.12) | -2.86(-3.03,-2.69) |
| China | female | 22377532.47(17670861.11,27377568.65) | 4742787.47(3573005.46,6429175.74) | -78.81 | 14614.05(14607.63,14620.48) | 3944.39(3940.65,3948.13) | -4.08(-4.19,-3.97) |
| Colombia | female | 504753.61(379158.19,672482.68) | 155122.60(113131.26,207349.09) | -69.27 | 8709.34(8683.77,8734.97) | 3005.40(2989.55,3021.31) | -3.47(-3.59,-3.35) |
| Comoros | female | 41496.16(34190.45,50092.77) | 16281.13(11963.85,21613.35) | -60.76 | 38185.19(37789.88,38583.73) | 13739.01(13512.80,13968.25) | -3.38(-3.46,-3.30) |
| Congo | female | 238883.27(196560.05,283903.05) | 293226.92(225300.00,372166.50) | 22.75 | 44682.70(44492.51,44873.53) | 30861.10(30742.77,30979.80) | -1.08(-1.39,-0.77) |
| Cook Islands | female | 571.61(411.27,759.37) | 113.64(80.38,162.04) | -80.12 | 18118.99(16589.64,19760.06) | 6220.77(5060.71,7594.43) | -3.35(-3.47,-3.22) |
| Costa Rica | female | 44298.17(33485.58,59554.69) | 19150.14(14718.01,25189.75) | -56.77 | 8006.35(7927.67,8085.65) | 3834.47(3777.43,3892.23) | -1.97(-2.19,-1.75) |
| Croatia | female | 70978.85(51296.87,95706.18) | 22532.23(16037.10,30600.23) | -68.26 | 14886.70(14770.89,15003.27) | 7819.05(7711.07,7928.29) | -2.36(-2.54,-2.18) |
| Cuba | female | 65506.53(47790.95,88886.07) | 23317.24(16633.60,33386.35) | -64.40 | 5283.00(5240.34,5325.94) | 2742.34(2705.19,2779.91) | -2.08(-2.15,-2.01) |
| Cyprus | female | 4593.31(3383.32,6156.08) | 1550.03(1224.44,1987.83) | -66.25 | 4812.75(4665.47,4963.84) | 1459.00(1383.65,1537.64) | -3.46(-3.83,-3.09) |
| Czechia | female | 145736.08(105120.01,196170.81) | 49844.83(34822.73,70910.59) | -65.80 | 13595.64(13521.09,13670.54) | 5941.60(5886.88,5996.73) | -2.38(-2.57,-2.19) |
| C?te d'Ivoire | female | 1230740.30(1039155.26,1423549.42) | 1015530.13(756728.09,1307269.78) | -17.49 | 43029.78(42949.08,43110.59) | 17693.98(17657.29,17730.73) | -2.84(-2.93,-2.75) |
| Democratic People's Republic of Korea | female | 513842.79(390358.20,641390.23) | 202926.25(149082.41,276060.58) | -60.51 | 16492.67(16443.44,16542.02) | 8884.84(8843.39,8926.44) | -2.57(-2.86,-2.28) |
| Democratic Republic of the Congo | female | 3479443.50(2836026.64,4184986.05) | 4927279.55(3807860.82,6252867.65) | 41.61 | 37776.52(37733.62,37819.46) | 26141.46(26116.77,26166.18) | -1.06(-1.59,-0.52) |
| Denmark | female | 9350.05(6952.58,12110.27) | 4232.94(3259.02,5303.67) | -54.73 | 2173.72(2126.97,2221.32) | 907.26(878.74,936.53) | -2.33(-2.59,-2.08) |
| Djibouti | female | 25994.11(20556.91,31792.61) | 30104.13(23575.13,39114.27) | 15.81 | 30298.81(29901.03,30700.79) | 15136.96(14951.68,15324.04) | -2.33(-2.54,-2.12) |
| Dominica | female | 860.01(609.75,1195.88) | 173.95(124.02,246.86) | -79.77 | 7197.02(6696.81,7726.80) | 2679.19(2261.25,3160.96) | -3.02(-3.16,-2.87) |
| Dominican Republic | female | 191968.52(153090.87,243936.53) | 75484.51(53944.36,104860.40) | -60.68 | 14170.44(14103.18,14237.96) | 5231.79(5192.56,5271.27) | -3.33(-3.40,-3.25) |
| Ecuador | female | 207774.63(157066.10,270483.92) | 126797.70(88256.26,176115.87) | -38.97 | 10810.06(10760.10,10860.19) | 5096.44(5066.63,5126.39) | -2.60(-2.86,-2.34) |
| Egypt | female | 1021205.57(825418.50,1265400.87) | 647444.02(511692.13,819814.61) | -36.60 | 9075.33(9056.30,9094.38) | 3516.28(3507.08,3525.49) | -2.29(-2.59,-1.99) |
| El Salvador | female | 211411.45(167042.74,270034.82) | 53312.64(38207.41,74284.13) | -74.78 | 19825.53(19735.23,19916.17) | 6082.33(6027.92,6137.14) | -3.80(-4.14,-3.46) |
| Equatorial Guinea | female | 47281.29(40216.61,54976.01) | 19727.75(14538.08,25921.86) | -58.28 | 46625.72(46171.53,47083.36) | 7600.12(7487.83,7713.77) | -6.30(-6.52,-6.09) |
| Eritrea | female | 399624.75(343494.64,467022.90) | 259644.56(201474.40,325460.72) | -35.03 | 49445.94(49279.83,49612.49) | 20392.86(20307.63,20478.37) | -2.80(-2.95,-2.64) |
| Estonia | female | 4184.35(3133.84,5635.01) | 973.41(754.39,1250.60) | -76.74 | 2463.41(2385.11,2543.74) | 939.31(877.91,1004.15) | -3.05(-3.18,-2.92) |
| Eswatini | female | 57460.31(44448.66,72417.07) | 22476.81(15832.56,30260.74) | -60.88 | 29131.48(28877.79,29386.92) | 11249.61(11093.32,11407.66) | -3.02(-3.09,-2.96) |
| Ethiopia | female | 6723389.50(5825960.13,7613409.50) | 4060114.10(3045585.44,5363202.54) | -39.61 | 54865.56(54821.04,54910.10) | 18586.35(18566.97,18605.74) | -3.84(-4.16,-3.52) |
| Fiji | female | 23358.77(17846.36,30860.34) | 9368.01(6974.42,12437.02) | -59.90 | 16995.17(16763.67,17229.25) | 6934.20(6784.06,7087.02) | -2.86(-3.02,-2.71) |
| Finland | female | 13362.52(10302.74,17071.41) | 5369.44(4041.95,6974.57) | -59.82 | 2839.89(2788.94,2891.59) | 1306.92(1270.03,1344.72) | -2.17(-2.31,-2.02) |
| France | female | 62877.28(48897.17,79692.67) | 43810.44(33848.52,63185.40) | -30.32 | 1101.39(1092.36,1110.48) | 759.82(752.40,767.30) | -0.47(-0.70,-0.24) |
| Gabon | female | 39376.34(29671.11,49975.75) | 25754.55(18536.84,35334.91) | -34.59 | 18655.45(18458.40,18854.14) | 8010.89(7907.30,8115.58) | -2.48(-2.69,-2.27) |
| Gambia | female | 108384.34(91179.15,126957.81) | 102446.76(77727.26,130786.45) | -5.48 | 45539.31(45249.09,45830.98) | 20513.69(20380.11,20647.97) | -2.56(-2.61,-2.51) |
| Georgia | female | 20364.77(15243.58,26980.34) | 7750.94(5493.11,10617.57) | -61.94 | 3046.55(3002.53,3091.09) | 2206.09(2154.72,2258.43) | -1.06(-1.55,-0.56) |
| Germany | female | 77362.75(57923.08,99867.30) | 48961.05(33628.16,64869.64) | -36.71 | 1214.91(1205.96,1223.91) | 828.14(820.60,835.75) | -0.97(-1.17,-0.77) |
| Ghana | female | 1503844.81(1242313.15,1765660.84) | 1175150.99(884482.86,1541843.88) | -21.86 | 43915.26(43839.83,43990.79) | 18263.21(18228.00,18298.47) | -2.92(-3.09,-2.75) |
| Greece | female | 38440.86(27923.12,51868.84) | 14892.11(11309.24,20427.50) | -61.26 | 4035.07(3991.56,4078.99) | 2176.59(2139.86,2213.86) | -1.50(-1.84,-1.15) |
| Greenland | female | 205.16(152.99,269.68) | 87.50(63.20,120.63) | -57.35 | 2873.66(2470.75,3325.76) | 1539.23(1218.64,1923.27) | -2.41(-2.64,-2.18) |
| Grenada | female | 2170.96(1570.74,2878.17) | 437.97(314.81,597.80) | -79.83 | 13109.99(12535.05,13705.62) | 4165.18(3759.93,4605.43) | -3.53(-3.75,-3.31) |
| Guam | female | 2199.98(1575.48,2909.80) | 919.90(663.09,1291.03) | -58.19 | 10714.96(10247.04,11199.55) | 5159.23(4813.04,5525.20) | -2.33(-2.47,-2.18) |
| Guatemala | female | 327515.39(258873.11,398156.63) | 116909.92(83971.63,164931.29) | -64.30 | 15702.83(15644.65,15761.17) | 4897.21(4867.12,4927.44) | -3.76(-3.93,-3.58) |
| Guinea | female | 734801.55(627093.67,853069.28) | 678151.56(521908.22,861443.97) | -7.71 | 51804.55(51676.76,51932.58) | 22119.55(22063.11,22176.11) | -2.72(-2.81,-2.63) |
| Guinea-Bissau | female | 116411.58(96350.67,137193.36) | 97166.42(74136.83,125925.10) | -16.53 | 46635.02(46348.24,46923.21) | 21458.37(21314.40,21603.10) | -2.40(-2.47,-2.32) |
| Guyana | female | 21295.54(16455.28,27553.30) | 5438.42(4114.60,7178.04) | -74.46 | 14065.20(13862.38,14270.36) | 5130.54(4985.95,5278.46) | -2.99(-3.07,-2.90) |
| Haiti | female | 422290.76(340509.42,518320.69) | 324356.26(241681.73,438308.31) | -23.19 | 29699.28(29602.58,29796.21) | 14815.85(14761.44,14870.43) | -2.44(-2.50,-2.37) |
| Honduras | female | 136769.14(105814.41,175831.77) | 79303.71(56560.76,112421.31) | -42.02 | 12306.17(12236.43,12376.22) | 4955.00(4918.46,4991.76) | -2.85(-2.91,-2.78) |
| Hungary | female | 163892.32(115816.11,218989.05) | 52596.87(36868.83,72286.14) | -67.91 | 15979.05(15896.16,16062.31) | 7809.16(7739.09,7879.76) | -2.33(-2.45,-2.22) |
| Iceland | female | 779.22(589.15,1030.54) | 364.96(280.84,480.69) | -53.16 | 2494.52(2310.92,2689.97) | 1099.62(984.21,1226.07) | -2.24(-2.41,-2.07) |
| India | female | 54271804.04(46424240.94,62971180.62) | 21977070.08(16957811.15,28563766.59) | -59.51 | 33709.58(33699.82,33719.33) | 13199.53(13193.52,13205.54) | -2.95(-3.31,-2.59) |
| Indonesia | female | 11459970.43(9572495.92,13603688.33) | 3443934.51(2573961.90,4607212.08) | -69.95 | 34971.60(34949.65,34993.57) | 10576.70(10564.63,10588.77) | -3.69(-3.79,-3.60) |
| Iran (Islamic Republic of) | female | 1315243.94(989355.60,1712260.96) | 295242.02(228727.62,376141.69) | -77.55 | 10557.64(10538.42,10576.87) | 3086.83(3074.92,3098.79) | -3.45(-3.74,-3.16) |
| Iraq | female | 594968.77(457752.62,774771.38) | 304689.47(239148.20,395207.54) | -48.79 | 14297.46(14258.38,14336.63) | 4721.59(4703.61,4739.63) | -3.47(-3.83,-3.10) |
| Ireland | female | 15803.87(11355.80,20872.08) | 5863.20(4125.30,8419.15) | -62.90 | 3392.30(3336.08,3449.30) | 1196.07(1164.17,1228.72) | -2.90(-3.17,-2.63) |
| Israel | female | 111136.78(81673.12,149949.37) | 78953.48(56003.01,106596.17) | -28.96 | 14919.95(14827.65,15012.71) | 6119.14(6074.23,6164.32) | -2.62(-2.86,-2.38) |
| Italy | female | 199845.62(154442.31,263884.68) | 76423.77(58694.02,98012.02) | -61.76 | 4506.42(4485.36,4527.56) | 2071.23(2055.72,2086.85) | -1.83(-2.11,-1.56) |
| Jamaica | female | 35125.30(25628.97,47399.57) | 10622.43(7571.61,14957.56) | -69.76 | 8468.28(8374.64,8562.77) | 3737.01(3661.27,3814.07) | -2.52(-2.62,-2.42) |
| Japan | female | 376830.45(294797.67,479539.56) | 139969.13(110639.98,174997.14) | -62.86 | 3451.68(3439.85,3463.55) | 1905.20(1894.50,1915.94) | -1.49(-1.67,-1.31) |
| Jordan | female | 101753.81(78899.77,130579.75) | 100201.00(74031.43,135906.64) | -1.53 | 12633.34(12551.75,12715.35) | 5771.24(5733.23,5809.45) | -2.59(-3.06,-2.11) |
| Kazakhstan | female | 420640.08(315060.75,558770.40) | 217172.37(156686.95,292338.75) | -48.37 | 16162.89(16110.85,16215.07) | 8094.09(8057.79,8130.52) | -2.23(-2.44,-2.02) |
| Kenya | female | 3201983.37(2757760.17,3618529.44) | 2528273.27(1911551.08,3275179.15) | -21.04 | 57091.37(57025.23,57157.57) | 27754.42(27717.95,27790.92) | -2.25(-2.52,-1.99) |
| Kiribati | female | 5731.97(4616.17,6892.67) | 4940.83(3695.07,6392.67) | -13.80 | 38460.22(37401.40,39542.24) | 24214.23(23504.15,24941.57) | -1.42(-1.48,-1.37) |
| Kuwait | female | 11037.00(8473.09,14345.44) | 5921.23(4562.45,7513.12) | -46.35 | 3964.16(3885.88,4043.65) | 1455.78(1416.41,1496.03) | -3.45(-3.57,-3.32) |
| Kyrgyzstan | female | 56305.71(42284.17,73755.67) | 53580.19(40715.54,70160.89) | -4.84 | 6519.91(6462.31,6577.91) | 4805.85(4762.84,4849.17) | -0.72(-0.99,-0.44) |
| Lao People's Democratic Republic | female | 453951.25(392869.40,517502.32) | 181986.68(131516.94,236729.32) | -59.91 | 48534.27(48383.01,48685.90) | 15973.12(15894.39,16052.15) | -3.81(-3.95,-3.67) |
| Latvia | female | 7213.22(5601.16,9036.36) | 1490.13(1161.89,1864.86) | -79.34 | 2565.89(2503.47,2629.52) | 1057.21(1001.15,1115.80) | -2.83(-2.98,-2.68) |
| Lebanon | female | 39678.04(30839.86,51342.27) | 19927.35(15769.03,25276.07) | -49.78 | 7663.78(7583.19,7745.04) | 3315.89(3266.91,3365.46) | -2.59(-2.89,-2.29) |
| Lesotho | female | 137317.59(113165.89,165728.03) | 54083.79(39983.94,71214.56) | -60.61 | 40255.06(40028.16,40482.99) | 17556.39(17397.69,17716.25) | -2.67(-2.71,-2.64) |
| Liberia | female | 158422.53(127829.23,189917.95) | 116885.85(86088.57,152715.79) | -26.22 | 25982.45(25842.06,26123.43) | 10749.68(10682.85,10816.84) | -3.16(-3.42,-2.90) |
| Libya | female | 72241.79(55521.57,93848.26) | 25327.73(19662.68,32609.46) | -64.94 | 7831.12(7770.18,7892.45) | 3657.23(3608.22,3706.78) | -1.93(-2.17,-1.70) |
| Lithuania | female | 11100.45(8415.27,14276.24) | 1753.98(1372.85,2224.91) | -84.20 | 2708.16(2654.81,2762.36) | 897.67(853.86,943.27) | -3.49(-3.59,-3.39) |
| Luxembourg | female | 768.69(590.57,999.80) | 515.49(394.30,661.38) | -32.94 | 2352.08(2178.52,2536.68) | 1047.58(954.83,1147.60) | -2.24(-2.42,-2.05) |
| Madagascar | female | 1033795.53(853085.45,1241601.70) | 1222743.22(937317.87,1548395.81) | 18.28 | 36514.74(36438.36,36591.25) | 20688.03(20648.29,20727.83) | -1.92(-2.12,-1.71) |
| Malawi | female | 1145075.55(973071.71,1312285.57) | 838705.42(623813.47,1085619.65) | -26.76 | 48298.27(48202.81,48393.88) | 20653.33(20606.18,20700.56) | -2.93(-3.17,-2.70) |
| Malaysia | female | 303777.69(246352.69,369304.07) | 114039.74(90402.96,141774.06) | -62.46 | 8885.13(8850.37,8920.00) | 3051.62(3032.02,3071.33) | -3.05(-3.25,-2.84) |
| Maldives | female | 19766.38(16221.96,23691.30) | 2775.75(2125.15,3664.84) | -85.96 | 36224.22(35682.35,36772.41) | 5845.67(5614.66,6084.25) | -5.68(-6.15,-5.20) |
| Mali | female | 1397026.06(1223120.52,1560460.23) | 1778805.03(1421736.76,2238858.70) | 27.33 | 65686.16(65568.74,65803.75) | 30099.78(30052.48,30147.13) | -2.55(-2.62,-2.47) |
| Malta | female | 1975.60(1458.63,2621.62) | 557.80(424.38,742.08) | -71.77 | 4682.53(4465.49,4908.08) | 1799.20(1645.83,1963.87) | -2.73(-2.92,-2.53) |
| Marshall Islands | female | 4307.04(3457.90,5159.88) | 1935.25(1416.19,2515.54) | -55.07 | 40526.41(39259.43,41826.18) | 22890.60(21824.26,23999.29) | -1.76(-1.82,-1.70) |
| Mauritania | female | 163298.94(132318.62,197218.62) | 105100.09(79240.32,137801.18) | -35.64 | 34104.68(33925.79,34284.31) | 11265.87(11193.05,11339.07) | -3.32(-3.41,-3.23) |
| Mauritius | female | 22685.40(17845.63,28752.89) | 4781.22(3634.32,6133.43) | -78.92 | 14127.97(13928.85,14329.40) | 4764.89(4618.90,4914.61) | -3.49(-3.59,-3.38) |
| Mexico | female | 3194520.70(2636596.51,3831161.67) | 1272581.67(945837.20,1720045.55) | -60.16 | 19330.02(19307.58,19352.48) | 8187.80(8172.62,8203.00) | -2.59(-2.75,-2.43) |
| Micronesia (Federated States of) | female | 10837.52(8925.78,13020.13) | 5268.76(4107.57,6588.24) | -51.38 | 48894.16(47928.99,49874.99) | 35722.96(34705.99,36764.57) | -0.91(-1.00,-0.83) |
| Monaco | female | 26.35(19.76,35.32) | 23.81(16.15,34.37) | -9.66 | 1545.89(985.48,2340.70) | 952.68(598.61,1460.18) | -1.07(-1.24,-0.90) |
| Mongolia | female | 29871.07(23108.90,38754.11) | 12211.74(8880.78,16535.73) | -59.12 | 6392.43(6313.86,6471.76) | 2274.63(2232.02,2317.89) | -3.81(-3.98,-3.64) |
| Montenegro | female | 11217.20(8112.92,15033.68) | 4251.40(3094.85,5779.77) | -62.10 | 14331.81(14054.14,14613.95) | 7986.83(7735.66,8244.64) | -2.32(-2.69,-1.94) |
| Morocco | female | 970872.85(735821.00,1238721.16) | 350727.74(253647.73,486653.10) | -63.88 | 19813.60(19771.42,19855.85) | 7373.39(7347.59,7399.27) | -3.02(-3.34,-2.70) |
| Mozambique | female | 1714364.36(1455739.25,2006957.17) | 1640386.55(1229033.24,2095645.44) | -4.32 | 53629.53(53543.33,53715.83) | 22723.91(22686.94,22760.94) | -2.88(-3.06,-2.69) |
| Myanmar | female | 2746456.59(2154422.83,3398107.48) | 757315.92(559110.54,1001758.47) | -72.43 | 37481.32(37433.64,37529.04) | 9952.17(9928.11,9976.26) | -4.44(-4.51,-4.36) |
| Namibia | female | 64513.14(49467.03,82140.05) | 38059.99(28327.87,49368.47) | -41.00 | 20859.36(20686.84,21033.01) | 9160.63(9062.27,9259.85) | -2.81(-2.98,-2.63) |
| Nauru | female | 425.01(316.77,555.59) | 278.79(198.59,370.43) | -34.40 | 20459.16(18458.22,22625.22) | 14633.13(12884.12,16567.24) | -0.94(-1.40,-0.48) |
| Nepal | female | 1375967.84(1153024.66,1666180.19) | 355609.61(259087.99,476380.15) | -74.16 | 31450.86(31393.69,31508.12) | 7873.31(7845.55,7901.16) | -4.50(-4.57,-4.43) |
| Netherlands | female | 30479.78(22673.35,39551.60) | 15174.17(10529.34,20176.57) | -50.22 | 2268.59(2241.89,2295.55) | 1142.09(1123.36,1161.10) | -1.54(-1.79,-1.29) |
| New Zealand | female | 6295.13(4900.90,8113.79) | 4218.58(3273.57,5436.96) | -32.99 | 1585.97(1545.05,1627.77) | 886.42(858.58,914.99) | -1.10(-1.44,-0.76) |
| Nicaragua | female | 96901.04(76394.72,124055.70) | 19844.03(14780.75,27682.14) | -79.52 | 10578.79(10507.06,10650.91) | 2042.87(2013.01,2073.09) | -5.59(-6.17,-5.01) |
| Niger | female | 1703172.28(1539351.13,1823989.83) | 3352789.11(2851360.70,3903420.00) | 96.86 | 80886.38(80755.38,81017.55) | 51238.75(51179.74,51297.81) | -1.46(-1.52,-1.39) |
| Nigeria | female | 4209954.43(3475099.18,5065761.92) | 4194689.80(3108487.42,5365716.22) | -0.36 | 19383.66(19363.28,19404.05) | 7939.40(7931.14,7947.67) | -3.00(-3.37,-2.62) |
| Niue | female | 64.00(46.35,87.07) | 15.92(11.03,22.43) | -75.12 | 16569.37(12554.75,21557.14) | 8652.24(4753.18,14769.83) | -1.95(-2.05,-1.84) |
| North Macedonia | female | 67339.56(51489.64,85933.85) | 20895.39(15209.73,28741.30) | -68.97 | 26371.89(26162.01,26583.16) | 13175.65(12986.86,13366.72) | -2.50(-2.61,-2.38) |
| Northern Mariana Islands | female | 562.78(420.36,752.09) | 354.52(247.71,486.13) | -37.01 | 9034.47(8259.81,9866.05) | 6642.58(5923.62,7433.21) | -0.45(-0.66,-0.23) |
| Norway | female | 9470.11(7502.90,12332.96) | 4720.39(3554.04,6248.27) | -50.15 | 2409.81(2358.81,2461.71) | 1038.73(1008.07,1070.18) | -1.93(-2.24,-1.61) |
| Oman | female | 57011.32(44487.27,75155.57) | 21023.07(16856.03,26172.16) | -63.12 | 13231.98(13115.10,13349.66) | 3497.28(3446.82,3548.32) | -3.83(-4.12,-3.54) |
| Pakistan | female | 6137711.64(5018910.30,7181906.37) | 2728389.83(2152376.33,3437002.14) | -55.55 | 24718.91(24697.50,24740.35) | 6400.28(6392.00,6408.57) | -3.92(-4.11,-3.73) |
| Palau | female | 257.66(188.79,343.64) | 115.02(78.68,158.14) | -55.36 | 11678.33(10216.12,13307.52) | 7399.74(6022.88,9029.09) | -1.20(-1.34,-1.05) |
| Palestine | female | 59600.29(44626.86,79529.14) | 39769.27(28767.27,54345.16) | -33.27 | 12181.69(12077.22,12286.85) | 4366.31(4320.83,4412.18) | -3.18(-3.52,-2.83) |
| Panama | female | 27746.39(21457.41,36611.53) | 16917.53(11963.04,22882.95) | -39.03 | 6731.99(6648.18,6816.66) | 3004.35(2956.31,3053.03) | -2.38(-2.68,-2.08) |
| Papua New Guinea | female | 154353.36(121279.06,190406.67) | 262459.05(205040.93,331559.86) | 70.04 | 18121.62(18023.91,18219.74) | 13044.32(12990.00,13098.83) | -0.53(-0.77,-0.30) |
| Paraguay | female | 158506.25(116431.13,209317.86) | 71331.18(49572.66,98226.32) | -55.00 | 19249.30(19149.64,19349.37) | 7308.44(7252.09,7365.15) | -2.96(-3.03,-2.89) |
| Peru | female | 561192.73(441708.47,711507.43) | 267848.71(188779.15,364870.14) | -52.27 | 13700.99(13662.89,13739.18) | 5856.95(5833.68,5880.29) | -3.07(-3.28,-2.85) |
| Philippines | female | 3118128.87(2667101.70,3730495.96) | 1923740.31(1475381.35,2481059.94) | -38.30 | 24811.89(24782.16,24841.65) | 11738.49(11720.59,11756.42) | -2.16(-2.38,-1.93) |
| Poland | female | 854910.28(641242.03,1117487.99) | 201833.26(145445.30,282857.64) | -76.39 | 18465.95(18424.57,18507.40) | 7082.53(7050.06,7115.13) | -3.03(-3.08,-2.98) |
| Portugal | female | 58510.42(41516.46,80304.58) | 15714.28(11728.13,21314.40) | -73.14 | 5858.57(5807.02,5910.50) | 2379.15(2339.85,2419.00) | -2.46(-2.70,-2.22) |
| Puerto Rico | female | 22022.93(15675.34,29746.17) | 3513.36(2612.71,4811.32) | -84.05 | 4535.47(4472.11,4599.57) | 1670.56(1609.79,1733.31) | -3.10(-3.23,-2.97) |
| Qatar | female | 2718.58(2096.47,3495.00) | 5851.48(4554.67,7412.70) | 115.24 | 4246.72(4076.95,4421.91) | 2397.00(2333.57,2461.81) | -1.26(-1.50,-1.01) |
| Republic of Korea | female | 228988.87(167511.55,315326.25) | 35251.56(26741.32,45947.04) | -84.61 | 4232.00(4213.29,4250.77) | 1191.77(1178.24,1205.44) | -3.78(-3.99,-3.57) |
| Republic of Moldova | female | 21445.25(16448.14,28127.01) | 3854.24(2941.57,4977.92) | -82.03 | 3509.22(3459.57,3559.43) | 1551.26(1499.35,1604.64) | -2.66(-2.89,-2.43) |
| Romania | female | 519356.82(385198.75,697165.80) | 136399.97(94936.32,187620.74) | -73.74 | 19194.36(19138.99,19249.86) | 9378.36(9325.61,9431.36) | -2.38(-2.53,-2.22) |
| Russian Federation | female | 327023.91(253948.00,401530.35) | 128590.97(97345.22,164915.25) | -60.68 | 1936.29(1929.10,1943.50) | 1036.66(1030.41,1042.94) | -1.85(-2.03,-1.66) |
| Rwanda | female | 650168.49(525754.34,778674.08) | 399587.55(306070.56,514137.94) | -38.54 | 36786.30(36689.95,36882.83) | 16106.64(16053.10,16160.32) | -3.13(-3.46,-2.80) |
| Saint Kitts and Nevis | female | 473.78(348.70,640.54) | 125.95(92.40,169.49) | -73.42 | 6790.32(6158.79,7473.26) | 2587.53(2127.47,3125.50) | -2.99(-3.08,-2.91) |
| Saint Lucia | female | 2563.46(1831.02,3366.01) | 609.36(452.57,810.26) | -76.23 | 10034.33(9629.09,10453.20) | 4295.25(3937.56,4679.30) | -2.49(-2.63,-2.34) |
| Saint Vincent and the Grenadines | female | 2177.81(1572.28,3040.63) | 572.30(387.75,796.33) | -73.72 | 10838.81(10361.26,11334.42) | 4743.43(4338.20,5179.65) | -2.54(-2.63,-2.46) |
| Samoa | female | 8787.09(6754.85,11085.87) | 6646.98(4774.52,8857.34) | -24.36 | 25684.70(25121.35,26258.08) | 17084.42(16655.13,17522.49) | -1.23(-1.30,-1.16) |
| San Marino | female | 37.69(28.42,49.31) | 22.35(16.43,32.40) | -40.70 | 1924.27(1322.09,2735.95) | 1064.42(647.50,1686.60) | -1.38(-1.63,-1.12) |
| Sao Tome and Principe | female | 9334.15(7485.43,11459.18) | 4285.69(3092.05,5831.36) | -54.09 | 32906.68(32196.84,33628.95) | 11239.25(10883.47,11604.47) | -3.72(-4.01,-3.42) |
| Saudi Arabia | female | 188323.78(148529.33,234212.65) | 75020.20(59589.35,93678.21) | -60.16 | 5608.82(5581.31,5636.43) | 2129.08(2112.78,2145.48) | -2.58(-2.86,-2.30) |
| Senegal | female | 599007.36(463698.26,744923.20) | 222762.49(163623.26,295670.23) | -62.81 | 31616.85(31531.22,31702.66) | 7124.20(7092.72,7155.80) | -4.31(-4.67,-3.95) |
| Serbia | female | 353296.88(279572.96,435909.64) | 86760.68(61865.26,119845.15) | -75.44 | 31815.15(31704.46,31926.16) | 14161.13(14060.11,14262.77) | -2.90(-3.14,-2.67) |
| Seychelles | female | 1178.96(846.57,1573.65) | 410.52(310.99,537.38) | -65.18 | 10072.30(9472.18,10702.76) | 3534.70(3177.35,3923.53) | -2.93(-3.21,-2.64) |
| Sierra Leone | female | 436825.25(361330.02,507230.98) | 384431.42(291573.00,487635.75) | -11.99 | 45914.87(45767.04,46063.08) | 20868.93(20798.00,20940.06) | -2.66(-2.91,-2.40) |
| Singapore | female | 17434.95(13417.06,22101.94) | 10107.99(7712.65,12645.87) | -42.02 | 5600.97(5510.70,5692.48) | 2470.13(2419.37,2521.73) | -2.72(-2.88,-2.57) |
| Slovakia | female | 103399.53(75322.06,135263.30) | 29452.19(20797.64,40105.56) | -71.52 | 16058.20(15954.69,16162.26) | 7076.19(6991.31,7161.91) | -2.64(-2.74,-2.54) |
| Slovenia | female | 21601.24(15827.78,29801.00) | 8307.40(5879.16,11660.58) | -61.54 | 10835.64(10682.68,10990.43) | 5512.46(5388.00,5639.29) | -2.24(-2.34,-2.15) |
| Solomon Islands | female | 39032.02(32273.04,46017.83) | 44413.53(34364.71,55080.88) | 13.79 | 51493.48(50953.21,52038.27) | 35339.77(34992.56,35689.72) | -1.02(-1.13,-0.91) |
| Somalia | female | 1668134.18(1568833.16,1754173.50) | 3859496.73(3433950.77,4190564.61) | 131.37 | 90225.74(90080.54,90371.12) | 76671.23(76590.31,76752.22) | -0.58(-0.61,-0.55) |
| South Africa | female | 1590140.66(1263628.80,1999790.75) | 640265.47(471462.77,841863.73) | -59.74 | 23138.37(23100.23,23176.56) | 8623.27(8600.81,8645.76) | -3.22(-3.26,-3.18) |
| South Sudan | female | 632259.46(527010.90,751950.00) | 691791.38(541684.92,855070.94) | 9.42 | 48602.10(48473.01,48731.47) | 32789.16(32705.74,32872.75) | -1.52(-1.62,-1.43) |
| Spain | female | 121595.84(88141.61,162535.70) | 39876.77(30414.37,53073.16) | -67.21 | 3234.03(3214.36,3253.80) | 1265.18(1252.01,1278.48) | -2.31(-2.65,-1.98) |
| Sri Lanka | female | 624125.71(501894.39,794052.69) | 209704.43(157401.46,265776.10) | -66.40 | 23295.56(23232.93,23358.32) | 8597.37(8557.39,8637.51) | -3.41(-3.55,-3.27) |
| Sudan | female | 1277238.55(1035161.75,1559553.79) | 818562.34(632680.48,1054103.33) | -35.91 | 27792.98(27739.94,27846.10) | 9990.85(9967.17,10014.56) | -3.21(-3.54,-2.87) |
| Suriname | female | 6812.26(5083.96,8861.68) | 3521.62(2530.47,4845.52) | -48.30 | 10497.14(10233.32,10766.42) | 5129.52(4950.56,5313.76) | -2.31(-2.34,-2.28) |
| Sweden | female | 19093.52(14823.88,24467.63) | 10260.47(7786.85,13707.54) | -46.26 | 2486.70(2449.54,2524.30) | 1158.20(1134.95,1181.84) | -1.74(-2.03,-1.46) |
| Switzerland | female | 7887.52(6142.32,10173.13) | 4729.35(3453.53,6996.26) | -40.04 | 1386.89(1354.80,1419.59) | 724.16(702.89,745.95) | -1.58(-1.77,-1.39) |
| Syrian Arab Republic | female | 420748.73(325953.62,544123.42) | 122026.01(96181.22,153491.65) | -71.00 | 14165.88(14119.77,14212.12) | 7366.90(7321.05,7412.98) | -1.89(-2.06,-1.72) |
| Taiwan (Province of China) | female | 145325.60(109151.14,194448.53) | 31545.67(23933.58,38722.39) | -78.29 | 5534.13(5503.46,5564.95) | 2178.18(2153.01,2203.59) | -2.49(-2.86,-2.11) |
| Tajikistan | female | 191180.33(151377.27,240659.04) | 201486.52(150743.07,263477.25) | 5.39 | 15734.23(15658.11,15810.63) | 11199.45(11147.03,11252.05) | -1.19(-1.57,-0.82) |
| Thailand | female | 1887577.24(1598409.56,2229296.90) | 187448.36(146532.86,240404.74) | -90.07 | 23751.34(23714.86,23787.86) | 4211.69(4190.72,4232.75) | -5.15(-5.49,-4.81) |
| Timor-Leste | female | 92000.71(79270.56,105650.79) | 50738.30(40192.16,63006.49) | -44.85 | 52889.33(52514.82,53265.98) | 19739.84(19552.07,19929.02) | -3.12(-3.21,-3.03) |
| Togo | female | 310965.18(245985.37,379857.50) | 241504.57(180302.59,316420.45) | -22.34 | 34416.77(34286.99,34546.94) | 14675.61(14613.34,14738.09) | -2.68(-2.82,-2.54) |
| Tokelau | female | 65.58(49.23,86.54) | 20.10(13.74,27.62) | -69.36 | 22715.06(17219.28,29542.71) | 11152.59(6503.99,18277.35) | -2.27(-2.39,-2.14) |
| Tonga | female | 4906.87(3679.55,6344.19) | 2779.28(2072.24,3694.59) | -43.36 | 24330.18(23619.21,25058.12) | 14809.86(14235.94,15401.92) | -1.25(-1.38,-1.12) |
| Trinidad and Tobago | female | 11142.86(8275.76,15271.70) | 3890.45(2959.08,4977.77) | -65.09 | 5649.90(5540.06,5761.49) | 3073.88(2970.39,3180.27) | -1.82(-1.92,-1.71) |
| Tunisia | female | 130224.71(96610.43,169535.26) | 35981.81(27018.60,47574.95) | -72.37 | 8655.07(8605.05,8705.31) | 2724.52(2694.63,2754.67) | -3.91(-4.10,-3.71) |
| Türkiye | female | 1478414.04(1182999.26,1760353.86) | 367179.84(262758.72,505942.41) | -75.16 | 14703.26(14677.76,14728.80) | 4102.30(4088.20,4116.45) | -4.15(-4.33,-3.98) |
| Turkmenistan | female | 50708.20(38406.44,64754.25) | 29643.02(22540.75,38363.81) | -41.54 | 6386.51(6326.43,6447.04) | 3896.87(3849.62,3944.59) | -1.68(-2.02,-1.35) |
| Tuvalu | female | 611.41(482.15,759.03) | 324.94(243.69,413.83) | -46.85 | 36145.91(33143.21,39355.09) | 18425.47(16371.40,20681.05) | -1.92(-2.01,-1.83) |
| Uganda | female | 1432186.42(1143742.31,1729444.88) | 1093567.87(824898.89,1462609.54) | -23.64 | 32517.25(32459.69,32574.89) | 10924.95(10902.88,10947.06) | -3.49(-3.59,-3.40) |
| Ukraine | female | 237407.23(188801.39,299718.02) | 84061.95(64901.25,104436.16) | -64.59 | 4179.10(4160.86,4197.41) | 3012.24(2989.31,3035.31) | -0.28(-0.58,0.02) |
| United Arab Emirates | female | 29459.99(23712.54,36100.04) | 50075.74(40569.23,61244.42) | 69.98 | 9768.94(9648.38,9890.68) | 7518.92(7450.47,7587.88) | -0.23(-0.54,0.09) |
| United Kingdom | female | 170258.33(132775.01,220487.87) | 76483.50(58869.64,96217.60) | -55.08 | 3151.07(3135.30,3166.90) | 1337.65(1327.72,1347.65) | -2.03(-2.29,-1.77) |
| United Republic of Tanzania | female | 1574161.63(1276579.94,1943080.51) | 1376476.35(1026328.59,1839073.58) | -12.56 | 24902.66(24860.43,24944.94) | 11094.99(11075.07,11114.93) | -2.31(-2.55,-2.07) |
| United States of America | female | 680423.56(522429.15,869877.85) | 359978.08(275911.25,465221.40) | -47.10 | 2490.01(2483.84,2496.20) | 1236.27(1232.02,1240.54) | -2.26(-2.52,-1.99) |
| United States Virgin Islands | female | 733.45(531.92,998.61) | 110.48(82.00,146.49) | -84.94 | 4639.45(4290.68,5010.74) | 1743.09(1410.22,2137.78) | -3.24(-3.38,-3.09) |
| Uruguay | female | 48065.27(34383.42,65013.30) | 20094.72(14223.40,28396.27) | -58.19 | 12148.35(12033.56,12264.04) | 6393.50(6300.22,6487.93) | -2.01(-2.20,-1.82) |
| Uzbekistan | female | 443604.39(335385.97,558116.70) | 209889.05(157310.05,276473.40) | -52.69 | 9594.37(9563.66,9625.16) | 4206.88(4187.75,4226.08) | -2.81(-2.86,-2.75) |
| Vanuatu | female | 14750.45(12058.42,17512.55) | 17060.18(13217.88,21307.56) | 15.66 | 44148.51(43392.75,44914.47) | 30097.65(29621.24,30580.12) | -1.12(-1.22,-1.01) |
| Venezuela (Bolivarian Republic of) | female | 353736.53(274271.82,448182.10) | 236993.30(172980.72,315830.86) | -33.00 | 9880.15(9845.03,9915.37) | 7454.44(7422.66,7486.34) | -0.93(-1.40,-0.47) |
| Viet Nam | female | 2578374.46(2106092.84,3176209.26) | 820231.05(634056.14,1074048.10) | -68.19 | 19313.13(19287.87,19338.41) | 6948.92(6932.75,6965.13) | -3.13(-3.43,-2.83) |
| Yemen | female | 1500251.57(1238350.87,1760504.53) | 1005101.36(765584.50,1266286.84) | -33.00 | 42153.55(42080.81,42226.38) | 15002.91(14971.19,15034.68) | -3.41(-3.49,-3.33) |
| Zambia | female | 662452.02(539949.87,785027.55) | 517357.55(386677.20,678062.59) | -21.90 | 33407.83(33320.31,33495.54) | 12420.57(12384.15,12457.07) | -3.34(-3.72,-2.97) |
| Zimbabwe | female | 678735.47(532447.65,846456.08) | 661972.96(500862.80,846679.05) | -2.47 | 27753.81(27683.42,27824.33) | 20885.68(20832.06,20939.41) | -0.44(-0.70,-0.17) |
| Global | male | 283122968.55(264566188.06,301784125.18) | 130621185.79(121202478.48,141892123.16) | -53.86 | 31132.36(31128.47,31136.25) | 12630.63(12628.30,12632.96) | -2.83(-3.06,-2.60) |
| Central Europe, eastern Europe, and central Asia | male | 5576160.90(5024734.97,6147611.95) | 1934264.78(1721987.09,2162992.08) | -65.31 | 10295.80(10286.72,10304.89) | 4670.17(4663.12,4677.22) | -2.70(-2.90,-2.50) |
| High-income | male | 3843561.28(3419268.00,4336111.99) | 1605479.44(1350791.37,1894909.80) | -58.23 | 4062.76(4058.46,4067.06) | 1785.75(1782.83,1788.68) | -2.38(-2.56,-2.19) |
| Latin America and Caribbean | male | 17536126.55(15770988.47,19514537.73) | 6204083.41(5171786.41,7515070.50) | -64.62 | 24042.17(24030.28,24054.07) | 8528.32(8521.23,8535.42) | -3.45(-3.56,-3.35) |
| Southeast Asia, east Asia, and Oceania | male | 70625189.51(62026516.90,80708791.50) | 14766916.15(12651261.00,16986582.36) | -79.09 | 26759.30(26752.64,26765.97) | 6377.77(6374.28,6381.27) | -4.36(-4.54,-4.19) |
| Sub-Saharan Africa | male | 64458782.62(62142360.92,66800572.50) | 61983614.24(58721559.77,65679418.17) | -3.84 | 55431.34(55416.85,55445.84) | 25250.91(25244.18,25257.64) | -2.58(-2.74,-2.43) |
| Andean Latin America | male | 1209632.48(999091.87,1424901.42) | 530063.51(409011.75,682062.92) | -56.18 | 15927.19(15896.78,15957.65) | 5696.75(5680.54,5713.00) | -3.72(-4.01,-3.43) |
| Australasia | male | 18171.86(14022.85,23308.90) | 14777.79(11299.21,19229.95) | -18.68 | 742.21(730.69,753.88) | 490.91(482.51,499.43) | -0.94(-1.16,-0.73) |
| Caribbean | male | 1046197.53(908448.25,1184882.11) | 530382.96(422365.01,655763.23) | -49.30 | 17858.42(17821.80,17895.09) | 8998.71(8972.82,9024.67) | -2.40(-2.50,-2.31) |
| Central Asia | male | 1776790.95(1508095.68,2052590.13) | 1001747.44(845314.51,1161515.20) | -43.62 | 13503.74(13482.47,13525.03) | 6826.88(6812.63,6841.15) | -2.30(-2.49,-2.11) |
| Central Europe | male | 3064314.53(2682554.17,3484331.01) | 650768.04(568609.23,734673.26) | -78.76 | 20648.30(20623.71,20672.92) | 7196.29(7177.78,7214.83) | -3.55(-3.71,-3.39) |
| Central Latin America | male | 7108582.29(6241622.72,8139123.22) | 2334046.11(1857592.55,2963779.24) | -67.17 | 21720.65(21703.67,21737.64) | 7349.93(7339.92,7359.94) | -3.41(-3.51,-3.31) |
| Central Sub-Saharan Africa | male | 7604558.88(6767762.29,8510725.31) | 10695188.03(8889724.21,12559978.72) | 40.64 | 57444.95(57401.00,57488.91) | 35575.37(35552.61,35598.14) | -1.46(-1.88,-1.03) |
| East Asia | male | 38105722.44(30059786.43,47952498.32) | 5329658.21(4032670.47,7076851.53) | -86.01 | 21872.89(21865.52,21880.27) | 3764.84(3761.47,3768.22) | -5.39(-5.53,-5.25) |
| Eastern Europe | male | 735055.42(602340.60,869785.53) | 281749.31(224305.50,344626.58) | -61.67 | 2803.38(2796.46,2810.32) | 1622.16(1615.56,1628.78) | -1.48(-1.69,-1.27) |
| Eastern Sub-Saharan Africa | male | 27468485.09(25985383.14,28826137.40) | 22820385.16(20920420.57,24782390.01) | -16.92 | 57888.26(57865.05,57911.47) | 24805.80(24794.90,24816.70) | -2.90(-3.11,-2.70) |
| High-income Asia Pacific | male | 574805.07(463652.17,695797.76) | 158585.94(127388.13,194124.18) | -72.41 | 3236.84(3227.83,3245.87) | 1425.84(1418.26,1433.45) | -2.23(-2.41,-2.05) |
| High-income North America | male | 584202.74(467961.56,732977.10) | 333611.87(256861.58,425884.88) | -42.89 | 1808.51(1803.62,1813.40) | 959.51(956.08,962.95) | -2.08(-2.56,-1.60) |
| North Africa and Middle East | male | 16087901.13(15086864.32,17248584.86) | 8920825.09(7947085.16,10006478.79) | -44.55 | 21803.01(21791.56,21814.47) | 9446.49(9439.79,9453.20) | -2.61(-2.89,-2.32) |
| Oceania | male | 409051.81(356775.68,474996.25) | 450223.86(379978.45,555555.22) | 10.07 | 28424.69(28331.39,28518.23) | 16207.77(16156.67,16259.00) | -1.36(-1.57,-1.16) |
| South Asia | male | 104995246.56(91056241.24,119869930.32) | 35206002.68(27239396.81,44806338.33) | -66.47 | 45580.72(45571.32,45590.13) | 13807.86(13802.88,13812.84) | -3.71(-4.05,-3.37) |
| Southeast Asia | male | 32110415.26(28648062.86,35653825.77) | 8987034.08(7644832.04,10487779.08) | -72.01 | 36533.21(36519.61,36546.81) | 10203.93(10196.69,10211.18) | -3.90(-4.03,-3.76) |
| Southern Latin America | male | 1419250.10(1160501.74,1719761.68) | 597996.02(424179.90,805098.53) | -57.87 | 19484.94(19451.31,19518.61) | 8280.76(8258.59,8302.97) | -2.62(-2.86,-2.38) |
| Southern Sub-Saharan Africa | male | 3596932.10(3148579.51,4157212.33) | 1679892.14(1360649.14,2095779.55) | -53.30 | 34447.97(34410.20,34485.76) | 13834.86(13812.50,13857.25) | -2.91(-2.99,-2.83) |
| Tropical Latin America | male | 8171714.25(6706501.91,9736039.69) | 2809590.84(1993570.85,4045125.72) | -65.62 | 30178.50(30156.76,30200.25) | 10947.96(10934.50,10961.43) | -3.48(-3.63,-3.33) |
| Western Europe | male | 1247131.51(1096603.52,1436012.35) | 500507.81(421910.19,588215.49) | -59.87 | 3435.25(3428.86,3441.66) | 1436.68(1432.47,1440.91) | -2.26(-2.49,-2.03) |
| Western Sub-Saharan Africa | male | 25788806.56(24620300.20,27103369.30) | 26788148.90(24797364.39,28947529.91) | 3.88 | 57286.30(57262.62,57309.98) | 24079.44(24069.67,24089.21) | -2.81(-2.85,-2.77) |
| Afghanistan | male | 1506024.18(1326217.58,1703110.14) | 2998717.27(2376925.61,3613310.13) | 99.11 | 66738.98(66624.62,66853.49) | 39342.76(39294.65,39390.91) | -1.74(-2.11,-1.38) |
| Albania | male | 223447.55(175228.59,271968.52) | 32646.08(24141.23,45494.05) | -85.39 | 37772.07(37605.03,37939.68) | 14342.34(14177.04,14509.20) | -3.62(-3.88,-3.36) |
| Algeria | male | 870442.15(644521.26,1164087.72) | 366995.13(277849.84,478817.50) | -57.84 | 15764.37(15728.80,15799.99) | 5247.42(5229.34,5265.56) | -3.68(-3.79,-3.58) |
| American Samoa | male | 1679.62(1205.24,2258.90) | 636.80(432.94,942.12) | -62.09 | 16652.00(15818.68,17519.13) | 8837.38(8109.49,9620.48) | -1.78(-2.07,-1.49) |
| Andorra | male | 130.19(92.16,178.15) | 61.75(44.83,81.11) | -52.57 | 2731.63(2247.05,3299.61) | 1216.42(908.31,1610.39) | -2.71(-2.82,-2.59) |
| Angola | male | 1569089.44(1330398.57,1798181.08) | 1825204.11(1352134.77,2353536.32) | 16.32 | 62986.88(62880.21,63093.68) | 23361.17(23324.78,23397.60) | -3.34(-3.58,-3.11) |
| Antigua and Barbuda | male | 607.58(457.78,810.89) | 222.34(167.99,293.99) | -63.41 | 6406.61(5865.65,6986.91) | 2541.02(2191.93,2934.24) | -2.68(-2.81,-2.54) |
| Argentina | male | 1031841.88(808937.48,1315757.57) | 480813.78(319607.11,686985.77) | -53.40 | 21217.35(21174.49,21260.28) | 9502.80(9474.44,9531.23) | -2.49(-2.72,-2.26) |
| Armenia | male | 11576.15(8720.24,14596.98) | 4218.52(3259.62,5449.36) | -63.56 | 2025.06(1985.55,2065.19) | 1397.29(1352.25,1443.52) | -1.13(-1.31,-0.95) |
| Australia | male | 12470.67(9238.73,16447.48) | 10979.47(8290.52,14714.09) | -11.96 | 616.65(605.07,628.42) | 439.33(430.60,448.20) | -0.81(-0.97,-0.64) |
| Austria | male | 24007.13(17428.07,33267.76) | 9713.09(7202.52,12592.53) | -59.54 | 3451.01(3404.73,3497.80) | 1444.81(1414.84,1475.30) | -2.49(-2.75,-2.24) |
| Azerbaijan | male | 81960.08(61153.77,112002.91) | 55897.26(43425.64,72028.33) | -31.80 | 6352.84(6306.27,6399.69) | 4614.06(4573.15,4655.26) | -1.48(-2.09,-0.86) |
| Bahamas | male | 1384.75(975.85,1887.94) | 563.65(415.18,766.34) | -59.30 | 3402.68(3213.20,3601.25) | 1468.21(1336.74,1610.21) | -2.37(-2.52,-2.21) |
| Bahrain | male | 6098.91(4689.49,7797.67) | 4208.95(3321.39,5435.52) | -30.99 | 6957.65(6770.16,7149.22) | 2761.35(2673.04,2852.03) | -2.58(-2.73,-2.44) |
| Bangladesh | male | 7818051.43(6546936.84,9143040.66) | 1427690.20(1153067.93,1775332.96) | -81.74 | 28924.69(28902.51,28946.88) | 6177.34(6166.12,6188.58) | -4.77(-5.03,-4.51) |
| Barbados | male | 1490.69(1063.09,2066.81) | 585.66(438.09,797.36) | -60.71 | 4741.91(4486.33,5009.38) | 2491.32(2276.55,2722.49) | -1.64(-1.79,-1.48) |
| Belarus | male | 33359.21(24208.79,47254.85) | 8361.74(6235.68,10459.36) | -74.93 | 2801.46(2770.25,2832.94) | 1120.37(1095.34,1145.86) | -2.78(-2.86,-2.70) |
| Belgium | male | 31391.59(22834.42,43050.71) | 10774.60(8196.53,13578.40) | -65.68 | 3387.36(3347.43,3427.67) | 1104.58(1082.57,1126.96) | -3.17(-3.37,-2.98) |
| Belize | male | 7132.32(5125.94,9431.19) | 3373.27(2311.58,4749.10) | -52.70 | 16919.82(16501.35,17346.64) | 5512.79(5314.77,5716.83) | -3.33(-3.43,-3.23) |
| Benin | male | 1018020.16(917380.78,1096514.07) | 1318211.41(1034178.96,1610052.35) | 29.49 | 79871.24(79706.14,80036.61) | 41693.26(41617.18,41769.45) | -2.01(-2.09,-1.93) |
| Bermuda | male | 195.63(140.80,273.58) | 60.13(46.36,76.50) | -69.27 | 3167.33(2712.91,3679.80) | 1413.81(1054.49,1866.13) | -2.65(-2.70,-2.59) |
| Bhutan | male | 51262.92(39827.98,63028.82) | 5656.27(4271.88,7590.70) | -88.97 | 37197.81(36849.12,37549.12) | 5959.12(5792.97,6129.12) | -5.97(-6.14,-5.81) |
| Bolivia (Plurinational State of) | male | 206377.54(149152.33,276536.35) | 112085.70(75414.11,166366.59) | -45.69 | 14649.26(14581.15,14717.62) | 6291.19(6252.28,6330.31) | -2.88(-3.14,-2.61) |
| Bosnia and Herzegovina | male | 161821.62(118761.92,216291.26) | 25804.73(18153.81,37424.89) | -84.05 | 29079.57(28930.00,29229.78) | 10322.17(10189.19,10456.59) | -4.01(-4.31,-3.71) |
| Botswana | male | 119148.36(94865.70,143517.01) | 47973.40(34249.78,66514.29) | -59.74 | 39771.80(39531.48,40013.28) | 13445.27(13316.47,13575.06) | -3.38(-3.47,-3.29) |
| Brazil | male | 7990046.60(6548571.81,9559538.25) | 2739091.54(1916164.79,3981435.08) | -65.72 | 30462.33(30440.13,30484.55) | 11118.80(11104.95,11132.66) | -3.47(-3.63,-3.31) |
| Brunei Darussalam | male | 2797.57(2041.12,3816.68) | 1050.48(790.08,1355.15) | -62.45 | 5436.16(5218.21,5661.15) | 1946.82(1818.40,2082.55) | -3.08(-3.22,-2.95) |
| Bulgaria | male | 164935.52(117289.36,231028.49) | 51190.83(38206.05,68506.82) | -68.96 | 18551.15(18455.70,18647.01) | 10052.52(9960.37,10145.39) | -1.71(-1.95,-1.48) |
| Burkina Faso | male | 2256228.73(2111305.87,2361124.70) | 2044084.10(1613238.98,2589752.33) | -9.40 | 91971.50(91844.01,92099.13) | 37318.88(37263.73,37374.08) | -3.15(-3.29,-3.01) |
| Burundi | male | 642904.96(530771.10,773559.20) | 583474.58(431797.65,785437.92) | -9.24 | 46971.81(46847.74,47096.13) | 19315.08(19261.32,19368.96) | -3.40(-3.72,-3.08) |
| Cabo Verde | male | 37316.90(29314.30,45282.00) | 4852.88(3136.32,7074.07) | -87.00 | 46907.10(46407.66,47410.73) | 6717.89(6519.08,6921.71) | -6.39(-6.60,-6.17) |
| Cambodia | male | 1353555.58(1157464.92,1561784.39) | 355570.40(252932.48,495182.91) | -73.73 | 55148.14(55048.18,55248.24) | 13595.24(13547.18,13643.43) | -4.60(-4.67,-4.54) |
| Cameroon | male | 2163070.89(2029004.89,2262588.25) | 1879705.21(1373919.01,2424102.82) | -13.10 | 86590.15(86468.48,86711.95) | 27324.21(27283.21,27365.26) | -3.48(-3.85,-3.11) |
| Canada | male | 126351.53(89877.32,178300.15) | 45758.21(33816.87,61443.23) | -63.78 | 4248.94(4223.95,4274.04) | 1403.16(1389.64,1416.79) | -3.19(-3.38,-2.99) |
| Central African Republic | male | 445457.92(389927.92,493270.01) | 617163.13(505626.82,739357.43) | 38.55 | 70151.82(69931.75,70372.41) | 52225.01(52085.69,52364.62) | -0.93(-0.97,-0.88) |
| Chad | male | 1443285.24(1378829.87,1501659.80) | 2823900.73(2383697.20,3302111.60) | 95.66 | 95357.11(95190.36,95524.10) | 59480.12(59405.49,59554.83) | -1.56(-1.64,-1.49) |
| Chile | male | 314747.52(218634.66,428595.63) | 92124.03(59696.83,137958.08) | -70.73 | 15549.76(15492.53,15607.16) | 4986.54(4952.60,5020.67) | -3.49(-3.77,-3.21) |
| China | male | 37168214.94(29209844.68,47037107.26) | 5006674.37(3678898.83,6744525.45) | -86.53 | 22086.93(22079.40,22094.47) | 3635.86(3632.50,3639.22) | -5.51(-5.65,-5.36) |
| Colombia | male | 601338.13(440718.27,821973.74) | 157553.91(104302.67,215403.82) | -73.80 | 10046.10(10019.06,10073.20) | 2894.69(2879.50,2909.93) | -3.98(-4.03,-3.93) |
| Comoros | male | 57627.43(47767.38,67835.72) | 21249.40(15342.96,28255.66) | -63.13 | 51813.99(51360.48,52270.63) | 17219.65(16971.55,17470.66) | -3.72(-3.84,-3.61) |
| Congo | male | 377414.29(330388.36,418779.60) | 504173.60(409878.85,604699.09) | 33.59 | 71206.44(70966.52,71447.01) | 51960.62(51808.88,52112.72) | -0.92(-1.13,-0.70) |
| Cook Islands | male | 745.85(535.88,1032.27) | 105.14(70.76,147.81) | -85.90 | 21680.91(20077.02,23385.92) | 5409.38(4364.18,6654.19) | -4.33(-4.50,-4.16) |
| Costa Rica | male | 50911.64(36309.39,70320.87) | 17246.11(12276.99,23729.41) | -66.13 | 8799.40(8718.82,8880.57) | 3286.39(3235.43,3338.01) | -2.62(-2.90,-2.34) |
| Croatia | male | 79084.66(56843.42,110228.89) | 20827.11(14624.53,29296.59) | -73.66 | 15802.80(15686.05,15920.27) | 6867.42(6768.50,6967.53) | -3.06(-3.28,-2.84) |
| Cuba | male | 76929.02(52067.40,107022.99) | 23776.96(16449.39,33625.28) | -69.09 | 5865.96(5822.18,5910.01) | 2651.54(2616.02,2687.45) | -2.67(-2.81,-2.54) |
| Cyprus | male | 6817.88(4791.40,9374.80) | 1693.41(1317.21,2201.57) | -75.16 | 6654.35(6486.02,6826.27) | 1476.21(1402.68,1552.83) | -4.43(-4.89,-3.97) |
| Czechia | male | 144016.60(99591.44,201245.28) | 41558.11(29967.80,57109.23) | -71.14 | 12946.12(12874.59,13017.98) | 4762.96(4714.72,4811.60) | -2.87(-3.08,-2.65) |
| C?te d'Ivoire | male | 1744076.53(1503589.61,1995847.75) | 1270885.74(920473.69,1652728.27) | -27.13 | 58404.92(58312.66,58497.30) | 20809.17(20770.50,20847.90) | -3.15(-3.25,-3.04) |
| Democratic People's Republic of Korea | male | 767646.92(587304.80,987046.88) | 300693.21(206297.20,411953.50) | -60.83 | 24148.16(24089.30,24207.13) | 12578.50(12530.18,12626.96) | -2.76(-3.09,-2.42) |
| Democratic Republic of the Congo | male | 5083345.64(4298480.70,5957406.50) | 7693615.18(6103543.78,9470018.28) | 51.35 | 54793.03(54741.73,54844.37) | 39440.01(39410.27,39469.77) | -0.92(-1.43,-0.40) |
| Denmark | male | 10279.72(7584.38,13718.72) | 4538.97(3380.29,5702.87) | -55.85 | 2265.03(2218.24,2312.64) | 917.08(888.98,945.90) | -2.43(-2.69,-2.16) |
| Djibouti | male | 40505.08(32488.41,49452.13) | 39528.15(30064.42,52317.81) | -2.41 | 42740.94(42293.83,43191.82) | 17347.93(17162.94,17534.50) | -3.04(-3.23,-2.85) |
| Dominica | male | 946.50(667.83,1344.77) | 139.35(97.35,199.11) | -85.28 | 7304.16(6818.00,7817.92) | 2110.55(1742.10,2542.10) | -3.78(-4.00,-3.55) |
| Dominican Republic | male | 255592.46(202090.51,323663.90) | 77599.30(53593.52,110212.65) | -69.64 | 18748.66(18671.51,18826.05) | 5138.81(5100.68,5177.18) | -4.38(-4.48,-4.27) |
| Ecuador | male | 263300.92(204921.40,340153.37) | 125285.24(83256.14,175897.12) | -52.42 | 13358.55(13303.40,13413.88) | 4890.34(4861.43,4919.39) | -3.30(-3.56,-3.03) |
| Egypt | male | 1436442.95(1169123.01,1787326.90) | 726794.22(560299.35,950131.93) | -49.40 | 12075.56(12054.17,12096.98) | 3714.49(3705.31,3723.69) | -2.92(-3.31,-2.53) |
| El Salvador | male | 288709.95(226227.91,370775.71) | 59663.57(40310.58,86812.52) | -79.33 | 26190.71(26088.47,26293.26) | 6374.56(6320.48,6429.01) | -4.68(-5.02,-4.35) |
| Equatorial Guinea | male | 73848.26(64800.76,82978.89) | 26422.56(17779.41,36618.78) | -64.22 | 70562.95(70017.24,71111.97) | 8346.08(8238.54,8454.76) | -7.51(-7.79,-7.23) |
| Eritrea | male | 569292.60(488788.03,649012.89) | 342296.34(260697.89,444008.58) | -39.87 | 66482.91(66297.57,66668.65) | 25241.89(25150.09,25333.96) | -3.12(-3.28,-2.95) |
| Estonia | male | 4477.95(3375.74,5916.12) | 1113.51(837.80,1416.62) | -75.13 | 2531.37(2453.01,2611.69) | 1022.75(959.92,1088.87) | -2.84(-3.00,-2.69) |
| Eswatini | male | 79283.41(62952.34,97070.58) | 27824.60(19487.30,38873.83) | -64.90 | 40480.28(40180.58,40781.74) | 13054.97(12891.74,13219.86) | -3.62(-3.68,-3.56) |
| Ethiopia | male | 8628240.42(7573012.76,9611716.92) | 4760580.86(3486765.21,6247823.08) | -44.83 | 66484.32(66436.74,66531.93) | 20591.83(20571.91,20611.77) | -4.10(-4.52,-3.69) |
| Fiji | male | 40723.51(30891.39,52965.29) | 18071.85(12451.57,25148.57) | -55.62 | 28027.57(27741.61,28315.92) | 12800.71(12605.14,12998.74) | -2.23(-2.38,-2.08) |
| Finland | male | 15515.05(11346.86,21146.07) | 5541.50(4187.91,7109.54) | -64.28 | 3146.47(3093.86,3199.81) | 1294.41(1258.24,1331.46) | -2.54(-2.70,-2.39) |
| France | male | 71880.91(55151.90,90391.89) | 47449.68(37526.54,59095.35) | -33.99 | 1198.67(1189.41,1208.00) | 792.51(784.97,800.12) | -0.82(-1.06,-0.59) |
| Gabon | male | 55403.34(42004.00,72320.04) | 28609.47(19113.89,41594.81) | -48.36 | 26705.90(26469.67,26943.79) | 9096.80(8985.51,9209.20) | -3.12(-3.36,-2.87) |
| Gambia | male | 173765.38(154952.99,192999.83) | 166000.64(128965.83,209035.76) | -4.47 | 73229.35(72863.13,73597.01) | 32613.25(32446.34,32780.84) | -2.54(-2.60,-2.48) |
| Georgia | male | 23164.54(16361.22,31774.78) | 8797.95(6163.51,12520.81) | -62.02 | 3330.96(3285.71,3376.70) | 2281.23(2231.15,2332.20) | -1.36(-2.03,-0.69) |
| Germany | male | 92244.92(68427.28,122308.88) | 44224.40(33591.62,58278.90) | -52.06 | 1367.18(1357.87,1376.54) | 708.18(701.25,715.17) | -1.92(-2.12,-1.73) |
| Ghana | male | 2486775.45(2166374.48,2781277.26) | 2064314.69(1558002.33,2647512.92) | -16.99 | 70954.68(70861.00,71048.45) | 30993.72(30948.91,31038.58) | -2.72(-2.86,-2.58) |
| Greece | male | 49291.25(34192.31,68676.29) | 13801.99(10241.27,18165.35) | -72.00 | 4920.74(4873.67,4968.18) | 1940.39(1906.05,1975.25) | -2.74(-3.18,-2.29) |
| Greenland | male | 127.69(97.82,171.19) | 62.78(46.96,85.24) | -50.83 | 1628.06(1336.89,1966.63) | 1005.88(759.50,1311.28) | -2.33(-2.83,-1.82) |
| Grenada | male | 2951.16(2076.13,4004.34) | 428.57(294.34,621.70) | -85.48 | 17309.99(16656.97,17983.19) | 3845.30(3465.89,4258.00) | -4.63(-4.95,-4.32) |
| Guam | male | 2228.83(1559.68,3150.20) | 758.24(514.72,1108.06) | -65.98 | 10369.83(9919.27,10836.35) | 4001.52(3706.61,4314.96) | -2.99(-3.17,-2.81) |
| Guatemala | male | 435653.63(347749.35,545396.28) | 121855.18(84647.75,173995.88) | -72.03 | 20362.20(20296.16,20428.42) | 4987.96(4957.69,5018.37) | -4.44(-4.61,-4.27) |
| Guinea | male | 1199040.18(1103135.95,1284175.85) | 1167577.22(890003.51,1448374.67) | -2.62 | 83539.47(83379.86,83699.33) | 37138.57(37066.49,37210.76) | -2.54(-2.62,-2.46) |
| Guinea-Bissau | male | 203124.76(184942.35,219685.77) | 180117.86(138734.26,224381.90) | -11.33 | 82762.31(82381.25,83144.76) | 38990.11(38799.28,39181.69) | -2.34(-2.40,-2.29) |
| Guyana | male | 29727.19(21865.18,39242.31) | 5629.23(4180.27,7533.74) | -81.06 | 19610.04(19371.10,19851.30) | 5128.77(4986.57,5274.18) | -3.99(-4.10,-3.89) |
| Haiti | male | 548538.59(439859.69,657753.76) | 380403.80(279725.86,500464.12) | -30.65 | 39282.51(39170.61,39394.66) | 16950.62(16893.09,17008.30) | -2.94(-3.04,-2.84) |
| Honduras | male | 189696.14(140004.22,243018.62) | 87621.10(58393.13,127777.30) | -53.81 | 16421.32(16342.19,16500.76) | 5232.52(5195.60,5269.66) | -3.55(-3.65,-3.46) |
| Hungary | male | 166935.63(114035.04,228818.67) | 44474.59(31319.27,61954.01) | -73.36 | 15558.81(15478.56,15639.41) | 6273.50(6212.02,6335.48) | -2.95(-3.08,-2.81) |
| Iceland | male | 904.30(659.15,1208.96) | 370.76(278.85,483.70) | -59.00 | 2748.54(2559.28,2949.19) | 1056.80(945.60,1178.59) | -2.80(-2.97,-2.64) |
| India | male | 87471018.98(74299527.10,101825733.64) | 30758916.56(23069738.70,40362571.79) | -64.84 | 50664.77(50653.35,50676.20) | 16855.39(16848.89,16861.90) | -3.48(-3.85,-3.11) |
| Indonesia | male | 15852622.63(12783182.93,19077508.20) | 3811957.46(2857545.38,5119390.51) | -75.95 | 46082.28(46057.86,46106.71) | 11168.43(11156.25,11180.61) | -4.43(-4.51,-4.35) |
| Iran (Islamic Republic of) | male | 1977115.91(1418241.64,2669077.36) | 325090.22(244355.11,439889.98) | -83.56 | 15360.07(15337.34,15382.83) | 3222.04(3210.18,3233.94) | -4.31(-4.68,-3.94) |
| Iraq | male | 895563.69(669874.58,1158578.92) | 387479.87(289231.37,530037.36) | -56.73 | 20424.79(20379.29,20470.37) | 5667.43(5648.25,5686.67) | -4.13(-4.67,-3.60) |
| Ireland | male | 16671.02(12224.68,22137.72) | 5300.42(3979.60,6951.34) | -68.21 | 3400.41(3345.27,3456.31) | 1044.75(1015.15,1075.07) | -3.35(-3.62,-3.09) |
| Israel | male | 160702.69(116659.14,213885.91) | 92176.90(64091.51,128367.48) | -42.64 | 20445.33(20340.12,20550.98) | 6782.15(6736.01,6828.54) | -3.28(-3.53,-3.02) |
| Italy | male | 253560.03(186785.30,351085.88) | 77957.15(58648.36,103059.15) | -69.25 | 5446.24(5423.56,5469.01) | 2013.81(1998.75,2028.96) | -2.61(-2.91,-2.30) |
| Jamaica | male | 36470.24(26077.07,50464.66) | 8662.84(6067.72,12259.61) | -76.25 | 8616.75(8522.97,8711.37) | 2980.99(2913.69,3049.58) | -3.28(-3.41,-3.15) |
| Japan | male | 331889.72(255197.12,423909.27) | 120556.45(94187.92,150277.70) | -63.68 | 2902.04(2891.41,2912.70) | 1561.40(1551.93,1570.93) | -1.59(-1.78,-1.39) |
| Jordan | male | 152093.26(114606.81,198220.14) | 114011.32(78357.11,164724.81) | -25.04 | 17911.70(17817.34,18006.46) | 6221.07(6182.65,6259.70) | -3.52(-4.07,-2.96) |
| Kazakhstan | male | 741563.11(556047.49,958444.62) | 343086.47(240157.26,476484.18) | -53.73 | 27901.35(27834.33,27968.51) | 12215.92(12172.74,12259.22) | -2.76(-2.94,-2.57) |
| Kenya | male | 3933308.90(3456150.56,4391293.19) | 2951444.65(2219882.29,3875989.90) | -24.96 | 68791.90(68719.75,68864.10) | 31664.96(31626.21,31703.76) | -2.35(-2.63,-2.08) |
| Kiribati | male | 9793.04(8414.05,11156.51) | 8910.84(6813.42,11069.95) | -9.01 | 62855.00(61536.82,64195.03) | 41167.06(40269.84,42080.35) | -1.25(-1.31,-1.19) |
| Kuwait | male | 10948.86(8272.60,14681.26) | 5763.60(4462.31,7256.36) | -47.36 | 3788.82(3713.71,3865.10) | 1358.75(1321.58,1396.77) | -3.60(-3.77,-3.43) |
| Kyrgyzstan | male | 71999.60(52770.88,97906.82) | 65929.87(47792.27,93660.29) | -8.43 | 8275.05(8210.66,8339.83) | 5644.56(5599.01,5690.40) | -0.99(-1.30,-0.69) |
| Lao People's Democratic Republic | male | 590543.28(522063.91,663137.15) | 209582.48(152733.55,276725.07) | -64.51 | 61598.28(61430.44,61766.48) | 17662.80(17581.39,17744.51) | -4.30(-4.53,-4.07) |
| Latvia | male | 6941.54(5193.36,9219.54) | 1711.69(1305.90,2183.78) | -75.34 | 2393.01(2333.67,2453.53) | 1147.56(1090.51,1206.98) | -2.26(-2.45,-2.08) |
| Lebanon | male | 51321.68(39315.97,66875.94) | 21195.46(16781.23,26932.32) | -58.70 | 9224.48(9139.14,9310.44) | 3268.57(3221.72,3315.97) | -3.23(-3.56,-2.89) |
| Lesotho | male | 204052.58(170137.08,237323.29) | 74205.45(54806.37,97767.49) | -63.63 | 58795.21(58524.74,59066.67) | 23542.85(23361.63,23725.21) | -2.99(-3.07,-2.92) |
| Liberia | male | 390307.15(337925.44,441577.66) | 303588.47(221723.17,398264.70) | -22.22 | 66318.95(66095.89,66542.59) | 26770.48(26668.86,26872.41) | -3.16(-3.28,-3.03) |
| Libya | male | 93250.94(69853.35,122123.55) | 30997.14(23705.95,40341.74) | -66.76 | 10147.42(10077.20,10218.02) | 4331.96(4279.08,4385.37) | -2.14(-2.42,-1.86) |
| Lithuania | male | 10728.03(8053.74,13744.67) | 2065.22(1587.64,2620.42) | -80.75 | 2530.04(2479.35,2581.56) | 1006.23(960.73,1053.45) | -3.03(-3.14,-2.92) |
| Luxembourg | male | 859.29(643.19,1142.70) | 511.84(391.86,660.02) | -40.43 | 2485.59(2310.86,2670.86) | 974.39(886.87,1068.88) | -2.61(-2.80,-2.42) |
| Madagascar | male | 1314796.54(1076654.17,1561831.46) | 1490810.71(1143291.46,1876045.52) | 13.39 | 45761.66(45677.06,45846.37) | 24589.79(24546.82,24632.82) | -2.09(-2.35,-1.82) |
| Malawi | male | 1425557.59(1232567.77,1599226.02) | 839237.51(608688.06,1119990.14) | -41.13 | 60180.59(60074.02,60287.30) | 20673.03(20625.60,20720.55) | -3.69(-3.94,-3.44) |
| Malaysia | male | 358970.89(284256.28,450033.94) | 119423.11(90795.68,151790.27) | -66.73 | 10099.26(10062.93,10135.69) | 3019.01(3000.02,3038.10) | -3.50(-3.71,-3.30) |
| Maldives | male | 29240.60(24464.47,34688.28) | 2837.19(2152.86,3686.58) | -90.30 | 52215.29(51573.27,52863.46) | 5745.56(5520.52,5977.86) | -7.05(-7.60,-6.50) |
| Mali | male | 1989106.66(1876308.23,2090719.72) | 2836843.88(2231070.27,3400842.28) | 42.62 | 91980.63(91843.85,92117.57) | 46401.58(46343.76,46459.47) | -2.25(-2.31,-2.20) |
| Malta | male | 2843.08(1970.56,3946.49) | 608.67(448.89,820.26) | -78.59 | 6361.91(6114.56,6617.34) | 1814.03(1665.03,1973.56) | -3.64(-3.89,-3.40) |
| Marshall Islands | male | 6230.36(5017.09,7331.89) | 2703.78(1950.00,3548.57) | -56.60 | 55287.34(53851.46,56753.94) | 30397.37(29198.97,31635.69) | -1.80(-1.89,-1.72) |
| Mauritania | male | 278512.37(240529.68,319108.41) | 152284.02(110384.90,203967.25) | -45.32 | 56417.04(56191.25,56643.54) | 15980.55(15895.02,16066.45) | -3.81(-3.91,-3.72) |
| Mauritius | male | 26348.71(19971.63,34063.03) | 4292.44(3311.17,5525.65) | -83.71 | 15986.81(15777.88,16198.00) | 4156.70(4021.32,4295.73) | -4.24(-4.39,-4.09) |
| Mexico | male | 4876612.24(4031265.40,5895732.96) | 1557214.21(1113356.26,2182347.04) | -68.07 | 29047.44(29020.16,29074.74) | 9778.67(9762.37,9795.00) | -3.44(-3.60,-3.28) |
| Micronesia (Federated States of) | male | 14886.46(12693.75,17124.79) | 6934.65(5379.65,8663.52) | -53.42 | 61942.05(60899.80,62998.70) | 43979.02(42888.08,45092.96) | -0.98(-1.03,-0.93) |
| Monaco | male | 25.99(19.79,33.03) | 20.23(15.47,26.51) | -22.17 | 1385.38(877.05,2110.97) | 798.87(475.49,1278.63) | -1.31(-1.50,-1.12) |
| Mongolia | male | 46432.84(34321.48,62531.14) | 13766.99(9925.22,19190.01) | -70.35 | 9774.03(9677.27,9871.54) | 2429.86(2386.81,2473.51) | -4.98(-5.15,-4.82) |
| Montenegro | male | 10732.81(7371.41,15353.72) | 3390.24(2298.07,4887.11) | -68.41 | 13027.01(12768.94,13289.32) | 5880.57(5673.08,6094.21) | -3.31(-3.80,-2.81) |
| Morocco | male | 1491831.99(1159957.89,1886827.11) | 443000.49(305775.08,621962.64) | -70.30 | 29819.58(29768.41,29870.81) | 8848.32(8820.70,8876.00) | -3.76(-4.13,-3.39) |
| Mozambique | male | 2374483.09(2124038.51,2621219.70) | 2269527.29(1696789.22,2867560.88) | -4.42 | 74830.37(74728.90,74931.95) | 31300.50(31257.09,31343.96) | -2.93(-3.08,-2.78) |
| Myanmar | male | 4132139.70(3388969.51,4846937.00) | 822894.65(583983.56,1164821.58) | -80.09 | 55294.09(55237.03,55351.20) | 10369.29(10345.15,10393.47) | -5.70(-5.89,-5.50) |
| Namibia | male | 87423.27(67960.51,110069.04) | 45847.32(32865.47,62682.36) | -47.56 | 28300.79(28100.55,28502.15) | 10916.11(10808.81,11024.26) | -3.23(-3.43,-3.02) |
| Nauru | male | 542.64(398.28,725.31) | 348.17(243.28,480.01) | -35.84 | 24745.50(22595.92,27050.16) | 16811.63(15010.62,18781.46) | -1.02(-1.67,-0.36) |
| Nepal | male | 1529223.41(1266042.78,1814095.88) | 279771.00(208836.17,378701.43) | -81.71 | 33067.24(33009.70,33124.87) | 5834.46(5810.97,5858.03) | -5.61(-5.68,-5.54) |
| Netherlands | male | 33271.19(24888.80,45662.13) | 14413.03(10443.27,19650.93) | -56.68 | 2351.72(2325.05,2378.65) | 1037.87(1020.19,1055.80) | -2.10(-2.32,-1.88) |
| New Zealand | male | 5701.18(4370.98,7027.66) | 3798.32(2917.98,4822.38) | -33.38 | 1337.49(1300.80,1375.01) | 740.72(715.96,766.19) | -1.22(-1.56,-0.88) |
| Nicaragua | male | 138846.06(105944.27,177201.06) | 19661.42(13961.32,27824.64) | -85.84 | 14713.32(14629.28,14797.74) | 1910.95(1882.73,1939.52) | -6.93(-7.61,-6.25) |
| Niger | male | 2116784.17(2059167.37,2166127.03) | 4915878.94(4334943.15,5447369.08) | 132.23 | 100516.86(100371.58,100662.31) | 73929.17(73859.44,73998.96) | -0.96(-1.00,-0.92) |
| Nigeria | male | 5368620.53(4441915.77,6556304.00) | 4118106.50(3019911.79,5550893.14) | -23.29 | 26457.25(26432.71,26481.81) | 7775.96(7767.72,7784.20) | -4.01(-4.35,-3.66) |
| Niue | male | 85.00(61.99,114.80) | 17.96(12.40,25.31) | -78.87 | 20437.47(16115.12,25645.18) | 8900.57(5066.77,14805.27) | -2.53(-2.69,-2.36) |
| North Macedonia | male | 93343.25(69308.82,120312.20) | 24097.84(16188.49,33415.16) | -74.18 | 34524.17(34291.20,34758.44) | 14280.59(14090.31,14473.00) | -3.22(-3.39,-3.05) |
| Northern Mariana Islands | male | 503.90(356.67,703.50) | 337.09(226.03,495.65) | -33.11 | 8264.89(7511.81,9075.96) | 5772.52(5131.39,6479.83) | -0.44(-0.73,-0.15) |
| Norway | male | 11211.00(8492.82,14182.76) | 4534.77(3445.30,5904.37) | -59.55 | 2686.21(2633.69,2739.58) | 953.14(924.03,983.03) | -2.70(-2.97,-2.44) |
| Oman | male | 76140.16(57504.98,100962.51) | 23092.64(18252.50,28821.53) | -69.67 | 17133.95(17003.22,17265.46) | 3633.87(3583.55,3684.74) | -4.46(-4.81,-4.11) |
| Pakistan | male | 8125689.81(6699451.21,9682225.97) | 2733968.64(2078682.41,3477384.65) | -66.35 | 30582.13(30559.11,30605.16) | 5928.60(5920.81,5936.40) | -4.52(-4.89,-4.14) |
| Palau | male | 300.07(207.42,417.08) | 119.38(78.31,173.21) | -60.21 | 12831.32(11337.38,14482.53) | 7115.85(5817.83,8645.74) | -1.50(-1.71,-1.30) |
| Palestine | male | 161204.52(125684.20,207890.06) | 56953.18(38774.59,78685.58) | -64.67 | 31592.42(31428.52,31756.98) | 5969.43(5917.55,6021.69) | -4.89(-5.08,-4.70) |
| Panama | male | 34631.73(25694.16,46810.95) | 16049.90(11108.38,23349.15) | -53.66 | 8105.33(8014.84,8196.64) | 2707.34(2662.84,2752.44) | -3.39(-3.78,-2.99) |
| Papua New Guinea | male | 208505.63(158894.75,265210.74) | 294984.83(228978.88,396219.42) | 41.48 | 22297.21(22193.35,22401.45) | 13406.77(13353.90,13459.80) | -1.04(-1.32,-0.77) |
| Paraguay | male | 181667.65(126622.61,252497.35) | 70499.30(45804.73,102788.84) | -61.19 | 21267.17(21164.38,21370.36) | 6839.20(6786.25,6892.50) | -3.52(-3.59,-3.45) |
| Peru | male | 739954.02(563583.35,931885.19) | 292692.56(196072.83,431072.89) | -60.44 | 17537.48(17494.87,17580.18) | 5911.09(5888.57,5933.69) | -4.13(-4.48,-3.78) |
| Philippines | male | 3762467.57(3115334.43,4631842.38) | 2414242.26(1742988.13,3207112.59) | -35.83 | 28391.92(28361.11,28422.75) | 13732.40(13713.64,13751.18) | -1.98(-2.36,-1.60) |
| Poland | male | 868092.97(626910.84,1177850.46) | 152908.74(107410.36,212736.36) | -82.39 | 17962.04(17921.97,18002.18) | 5106.88(5079.87,5134.01) | -3.97(-4.03,-3.90) |
| Portugal | male | 86249.23(58976.36,121084.97) | 17070.33(12025.50,23703.28) | -80.21 | 8339.88(8279.40,8400.73) | 2473.21(2433.85,2513.09) | -3.40(-3.73,-3.07) |
| Puerto Rico | male | 21045.45(15443.02,28907.64) | 2654.58(2035.01,3465.99) | -87.39 | 4126.80(4067.43,4186.89) | 1231.99(1180.06,1285.86) | -3.72(-3.84,-3.60) |
| Qatar | male | 2951.68(2243.36,3811.73) | 5835.10(4506.03,7434.16) | 97.69 | 4298.19(4133.42,4468.03) | 2279.62(2219.16,2341.39) | -1.44(-1.68,-1.20) |
| Republic of Korea | male | 221760.58(160059.13,313366.71) | 27839.19(21275.85,35716.60) | -87.45 | 3782.80(3765.86,3799.81) | 917.93(906.11,929.89) | -4.14(-4.43,-3.85) |
| Republic of Moldova | male | 22183.31(16647.69,29931.85) | 4484.37(3392.49,5941.84) | -79.78 | 3501.45(3452.65,3550.78) | 1704.41(1650.95,1759.28) | -2.42(-2.69,-2.15) |
| Romania | male | 561548.58(392353.54,736622.48) | 110928.46(75540.66,155921.43) | -80.25 | 19951.72(19896.18,20007.40) | 7282.16(7236.57,7327.99) | -3.42(-3.61,-3.23) |
| Russian Federation | male | 377814.43(295731.44,461727.78) | 160389.90(122502.19,198787.43) | -57.55 | 2150.65(2143.22,2158.11) | 1234.58(1227.91,1241.27) | -1.64(-1.83,-1.45) |
| Rwanda | male | 737167.18(580114.19,890240.12) | 377868.99(273955.00,513323.95) | -48.74 | 41852.61(41749.48,41955.94) | 14849.50(14798.66,14900.49) | -3.83(-4.25,-3.42) |
| Saint Kitts and Nevis | male | 532.62(363.67,740.63) | 104.60(76.85,140.70) | -80.36 | 7428.77(6773.81,8133.90) | 2104.16(1693.00,2592.31) | -3.75(-3.94,-3.56) |
| Saint Lucia | male | 2606.01(1794.42,3642.69) | 510.52(355.02,738.68) | -80.41 | 10018.47(9617.31,10433.04) | 3374.23(3068.36,3705.02) | -3.14(-3.34,-2.95) |
| Saint Vincent and the Grenadines | male | 2941.94(2074.63,3979.26) | 552.24(378.71,785.21) | -81.23 | 14233.58(13691.66,14793.10) | 4397.50(4012.87,4812.26) | -3.68(-3.86,-3.50) |
| Samoa | male | 14128.74(10802.46,17769.62) | 9251.17(6792.26,12380.96) | -34.52 | 37454.30(36806.35,38111.47) | 22207.14(21734.32,22688.13) | -1.60(-1.67,-1.53) |
| San Marino | male | 39.50(28.99,51.98) | 21.93(16.34,28.93) | -44.47 | 1943.67(1346.51,2743.62) | 969.82(583.15,1548.10) | -1.68(-2.05,-1.30) |
| Sao Tome and Principe | male | 19216.04(16472.39,21861.48) | 6977.90(5055.70,9328.02) | -63.69 | 66624.28(65634.37,67626.11) | 17783.08(17342.15,18233.10) | -4.36(-4.56,-4.16) |
| Saudi Arabia | male | 241856.16(191917.72,298290.59) | 94451.40(72944.83,117891.04) | -60.95 | 7022.09(6991.69,7052.59) | 2483.84(2466.74,2501.04) | -2.57(-2.92,-2.22) |
| Senegal | male | 1512240.91(1383278.35,1619459.12) | 412131.59(294651.05,560583.95) | -72.75 | 82707.01(82567.76,82846.44) | 12486.24(12446.05,12526.53) | -5.47(-6.01,-4.94) |
| Serbia | male | 412802.70(316319.25,524221.32) | 102267.80(71263.36,139222.57) | -75.23 | 39757.48(39628.00,39887.31) | 14869.52(14771.27,14968.33) | -3.55(-3.84,-3.25) |
| Seychelles | male | 1400.74(985.37,1939.87) | 331.30(241.05,450.03) | -76.35 | 11581.65(10947.90,12244.68) | 2753.67(2443.21,3094.72) | -3.99(-4.38,-3.59) |
| Sierra Leone | male | 748937.85(678465.16,809092.06) | 634557.28(492312.17,806489.67) | -15.27 | 79649.72(79456.31,79843.51) | 34462.36(34371.66,34553.25) | -2.76(-2.97,-2.55) |
| Singapore | male | 18357.20(14115.30,23800.67) | 9139.83(7130.17,11448.77) | -50.21 | 5443.84(5358.21,5530.63) | 2192.32(2144.89,2240.57) | -2.97(-3.21,-2.73) |
| Slovakia | male | 108826.91(75568.76,150900.21) | 24916.75(17689.22,35783.19) | -77.10 | 16274.52(16171.99,16377.58) | 5691.98(5617.57,5767.19) | -3.42(-3.54,-3.31) |
| Slovenia | male | 19721.58(13348.08,27419.37) | 6284.60(4484.59,8900.45) | -68.13 | 9461.64(9321.42,9603.61) | 3943.32(3840.68,4048.20) | -2.91(-3.03,-2.80) |
| Solomon Islands | male | 51976.81(44129.64,59096.78) | 56163.52(43466.39,69967.17) | 8.05 | 63048.04(62474.21,63626.03) | 41081.77(40723.36,41442.69) | -1.14(-1.28,-1.00) |
| Somalia | male | 2091701.73(2027844.42,2140816.10) | 5011082.18(4734580.88,5214342.75) | 139.57 | 99698.79(99556.23,99841.52) | 92302.04(92216.66,92387.48) | -0.30(-0.32,-0.27) |
| South Africa | male | 2138815.51(1736495.25,2632631.19) | 679788.88(494291.96,962434.31) | -68.22 | 31239.43(31195.12,31283.79) | 8961.52(8938.78,8984.31) | -4.22(-4.30,-4.14) |
| South Sudan | male | 873605.29(736805.63,1012805.07) | 925305.17(737032.27,1142320.42) | 5.92 | 61719.59(61580.66,61858.77) | 40562.82(40473.52,40652.27) | -1.67(-1.78,-1.55) |
| Spain | male | 137308.81(99171.28,196706.11) | 56822.28(44337.27,71881.48) | -58.62 | 3466.38(3446.49,3486.38) | 1732.44(1717.17,1747.82) | -1.79(-1.96,-1.61) |
| Sri Lanka | male | 928443.00(750566.32,1145637.58) | 200548.91(153499.21,257619.87) | -78.40 | 33676.94(33603.08,33750.94) | 8004.23(7965.98,8042.62) | -4.79(-4.90,-4.68) |
| Sudan | male | 2236489.17(1867563.79,2620465.57) | 1206130.32(896991.32,1587817.97) | -46.07 | 45601.60(45536.29,45666.97) | 13933.67(13906.53,13960.84) | -3.71(-4.09,-3.33) |
| Suriname | male | 8605.49(6174.31,11842.36) | 3765.81(2565.82,5549.76) | -56.24 | 13213.35(12918.07,13514.08) | 5195.75(5019.65,5376.89) | -3.03(-3.10,-2.97) |
| Sweden | male | 22974.30(17064.38,30884.22) | 10524.52(8129.34,13250.51) | -54.19 | 2803.25(2764.85,2842.09) | 1116.63(1094.24,1139.41) | -2.32(-2.60,-2.03) |
| Switzerland | male | 8214.10(5921.72,10700.21) | 5035.21(3794.74,6554.82) | -38.70 | 1362.86(1331.80,1394.51) | 725.78(704.81,747.26) | -1.80(-1.92,-1.67) |
| Syrian Arab Republic | male | 590654.45(440602.91,766968.43) | 157087.68(116576.14,208670.06) | -73.40 | 19082.71(19030.22,19135.31) | 9198.42(9148.03,9249.04) | -2.16(-2.34,-1.97) |
| Taiwan (Province of China) | male | 169860.57(118237.33,241054.34) | 22290.63(17536.41,28833.65) | -86.88 | 6042.82(6011.91,6073.87) | 1433.31(1413.33,1453.54) | -3.99(-4.34,-3.64) |
| Tajikistan | male | 237647.25(184036.43,298480.72) | 245454.34(180328.39,320933.49) | 3.29 | 18961.97(18879.47,19044.76) | 12701.46(12647.31,12755.80) | -1.53(-2.01,-1.05) |
| Thailand | male | 2195607.76(1851722.97,2637146.56) | 167455.72(129136.34,216410.78) | -92.37 | 24812.09(24776.30,24847.93) | 3575.09(3556.17,3594.09) | -5.86(-6.07,-5.65) |
| Timor-Leste | male | 119019.34(103638.58,134548.32) | 64323.57(50434.50,82240.19) | -45.96 | 65219.07(64816.95,65623.14) | 23505.57(23306.21,23706.26) | -3.25(-3.31,-3.18) |
| Togo | male | 639525.57(550115.03,716808.07) | 487817.27(361066.51,656275.06) | -23.72 | 70784.27(70600.64,70968.27) | 28518.74(28434.09,28603.59) | -2.79(-2.89,-2.68) |
| Tokelau | male | 95.25(70.50,123.45) | 24.42(16.13,35.83) | -74.36 | 30780.87(24584.66,38162.91) | 12080.66(7450.77,18920.72) | -2.94(-3.13,-2.74) |
| Tonga | male | 7327.18(5459.62,9243.74) | 3734.53(2668.76,5118.50) | -49.03 | 33675.05(32868.95,34496.84) | 18203.74(17595.60,18828.42) | -1.57(-1.73,-1.41) |
| Trinidad and Tobago | male | 12386.05(8818.28,16491.71) | 3318.23(2508.01,4340.50) | -73.21 | 6179.31(6065.21,6295.12) | 2526.97(2434.62,2622.12) | -2.84(-2.96,-2.71) |
| Tunisia | male | 180399.87(127882.75,252555.15) | 41260.27(30911.68,56639.01) | -77.13 | 11315.75(11259.95,11371.77) | 2882.21(2852.66,2912.01) | -4.44(-4.60,-4.28) |
| Türkiye | male | 1834746.80(1497699.55,2212748.10) | 305535.33(215253.39,435013.14) | -83.35 | 17082.80(17056.00,17109.64) | 3247.95(3235.64,3260.29) | -5.44(-5.71,-5.17) |
| Turkmenistan | male | 60643.93(46388.28,80631.99) | 32132.43(23230.59,43959.45) | -47.01 | 7540.42(7475.67,7605.61) | 4028.26(3981.52,4075.43) | -2.25(-2.77,-1.73) |
| Tuvalu | male | 960.29(784.79,1134.23) | 476.96(326.32,660.54) | -50.33 | 50748.57(47372.41,54311.38) | 24344.44(22098.66,26770.46) | -2.02(-2.15,-1.90) |
| Uganda | male | 1709617.83(1368938.44,2104399.03) | 1144400.38(825881.57,1569542.25) | -33.06 | 38417.54(38355.08,38480.07) | 11027.20(11005.45,11048.99) | -3.98(-4.13,-3.84) |
| Ukraine | male | 279550.94(215238.56,344064.39) | 103622.87(82606.68,126745.26) | -62.93 | 4783.49(4764.27,4802.76) | 3585.32(3560.84,3609.94) | -0.46(-0.69,-0.22) |
| United Arab Emirates | male | 28945.99(23382.79,34371.27) | 39991.76(31448.49,49286.49) | 38.16 | 9000.26(8887.65,9114.00) | 5759.87(5700.88,5819.35) | -0.70(-1.01,-0.38) |
| United Kingdom | male | 209713.36(152136.88,286092.14) | 76899.72(58887.66,99765.51) | -63.33 | 3681.56(3664.88,3698.30) | 1286.04(1276.43,1295.71) | -2.63(-2.91,-2.35) |
| United Republic of Tanzania | male | 2115678.74(1710668.60,2555896.62) | 1396663.76(997457.63,1854661.36) | -33.99 | 33188.47(33140.07,33236.92) | 11137.21(11117.25,11157.19) | -3.20(-3.43,-2.98) |
| United States of America | male | 457710.12(345332.67,598667.03) | 287785.65(216536.80,372640.29) | -37.12 | 1561.17(1556.42,1565.93) | 913.05(909.53,916.57) | -1.89(-2.47,-1.30) |
| United States Virgin Islands | male | 688.31(494.98,959.74) | 83.13(63.13,111.62) | -87.92 | 4225.45(3895.97,4576.98) | 1223.66(954.55,1550.80) | -3.97(-4.20,-3.73) |
| Uruguay | male | 72593.29(52595.95,96442.69) | 25025.20(17044.56,36972.08) | -65.53 | 17663.22(17526.91,17800.39) | 7600.21(7500.47,7701.05) | -2.64(-2.88,-2.40) |
| Uzbekistan | male | 501803.46(374992.38,650542.04) | 232463.61(171405.75,305368.68) | -53.67 | 10735.20(10702.81,10767.65) | 4238.78(4220.33,4257.29) | -3.23(-3.32,-3.14) |
| Vanuatu | male | 22243.25(19272.19,25608.97) | 26430.73(20745.35,33118.68) | 18.83 | 61695.65(60839.16,62561.50) | 43513.01(42961.16,44070.49) | -0.97(-1.08,-0.86) |
| Venezuela (Bolivarian Republic of) | male | 492182.75(378262.67,633026.88) | 297180.69(205507.70,418727.66) | -39.62 | 13153.21(13113.49,13193.03) | 9040.54(9006.19,9075.00) | -1.33(-1.90,-0.76) |
| Viet Nam | male | 2713604.77(2178503.60,3404513.67) | 801039.57(614570.69,1121759.29) | -70.48 | 19245.59(19220.72,19270.49) | 6273.14(6258.18,6288.14) | -3.14(-3.55,-2.74) |
| Yemen | male | 2234577.02(1921580.67,2546963.42) | 1557913.27(1170358.12,2044353.82) | -30.28 | 58551.52(58469.01,58634.12) | 21910.79(21873.34,21948.29) | -3.40(-3.57,-3.23) |
| Zambia | male | 934366.87(783403.53,1111187.71) | 647048.00(465567.42,869549.33) | -30.75 | 47778.53(47673.55,47883.69) | 15351.94(15311.55,15392.42) | -3.86(-4.33,-3.38) |
| Zimbabwe | male | 968208.95(768467.07,1193932.01) | 804252.50(567633.59,1074976.40) | -16.93 | 39674.96(39590.87,39759.19) | 25086.04(25027.55,25144.65) | -1.02(-1.29,-0.75) |

**Stable 3. DALYs cases and Age-standardized DALY rate of Protein-energy malnutrition in 1990 and 2021 and its trends.**

| location | sex | number | | | Age-standardized rate | | |
| --- | --- | --- | --- | --- | --- | --- | --- |
|  |  | 1990 | 2021 | change rate in case(%) | 1990 | 2021 | EAPC |
| Global | both | 38856280.14(32529290.28,49104466.19) | 8615187.61(6818257.96,10357178.71) | -77.83 | 1926.45(1925.76,1927.14) | 403.98(403.68,404.29) | -5.05(-5.60,-4.51) |
| Central Europe, eastern Europe, and central Asia | both | 93873.55(77026.06,112198.84) | 16758.66(12828.70,21517.43) | -82.15 | 84.27(83.68,84.87) | 20.15(19.81,20.50) | -5.36(-5.83,-4.89) |
| High-income | both | 88653.97(82058.64,96669.34) | 21303.00(14575.35,31236.71) | -75.97 | 38.60(38.31,38.90) | 11.00(10.84,11.16) | -3.07(-3.39,-2.75) |
| Latin America and Caribbean | both | 2326430.74(2159854.10,2511511.13) | 283631.98(226442.01,359428.14) | -87.81 | 1305.18(1303.24,1307.12) | 161.20(160.50,161.90) | -6.79(-7.02,-6.56) |
| Southeast Asia, east Asia, and Oceania | both | 4210849.07(3636388.83,5147753.48) | 400173.73(333089.81,470119.37) | -90.50 | 734.59(733.80,735.37) | 93.67(93.34,94.00) | -9.08(-10.85,-7.27) |
| Sub-Saharan Africa | both | 15392027.70(11770755.65,20727597.54) | 5610462.89(4136644.42,7132332.75) | -63.55 | 5220.89(5217.81,5223.97) | 975.84(974.90,976.77) | -5.18(-5.82,-4.54) |
| Andean Latin America | both | 379003.74(310918.28,462677.68) | 37980.07(28129.28,49769.34) | -89.98 | 1718.93(1712.03,1725.84) | 162.27(160.32,164.24) | -7.76(-8.12,-7.39) |
| Australasia | both | 95.54(83.14,108.42) | 56.14(39.00,153.96) | -41.24 | 1.38(1.05,1.79) | 0.82(0.59,1.12) | -1.12(-1.55,-0.69) |
| Caribbean | both | 281131.64(227691.57,344229.34) | 80718.57(57363.98,113333.84) | -71.29 | 2018.00(2009.56,2026.47) | 631.43(626.58,636.30) | -3.50(-3.83,-3.17) |
| Central Asia | both | 51006.01(43111.80,60217.37) | 7833.73(6082.20,9879.98) | -84.64 | 165.28(163.68,166.89) | 24.72(24.12,25.32) | -7.16(-7.79,-6.52) |
| Central Europe | both | 7368.91(5896.26,9163.22) | 1533.60(1296.14,1818.06) | -79.19 | 26.16(25.51,26.83) | 7.95(7.51,8.42) | -4.59(-5.06,-4.13) |
| Central Latin America | both | 947766.35(876973.14,1033886.20) | 126227.59(96854.63,165640.04) | -86.68 | 1119.85(1117.21,1122.49) | 151.87(150.84,152.91) | -6.16(-6.31,-6.01) |
| Central Sub-Saharan Africa | both | 1899292.63(1366384.24,2821910.78) | 541623.21(341185.40,781551.43) | -71.48 | 5744.95(5735.53,5754.38) | 826.96(824.51,829.41) | -6.13(-6.71,-5.54) |
| East Asia | both | 2229928.39(1837792.28,2700273.95) | 44123.88(34971.51,54262.20) | -98.02 | 566.39(565.56,567.23) | 19.36(19.15,19.56) | -15.54(-18.58,-12.38) |
| Eastern Europe | both | 35498.62(26630.44,46027.94) | 7391.33(5245.41,10175.43) | -79.18 | 67.70(66.92,68.48) | 21.55(20.99,22.12) | -3.88(-4.29,-3.48) |
| Eastern Sub-Saharan Africa | both | 8771099.50(6714464.95,11817909.75) | 2567173.34(1894310.09,3283874.44) | -70.73 | 7555.16(7549.33,7561.00) | 1231.09(1229.36,1232.82) | -5.58(-6.53,-4.63) |
| High-income Asia Pacific | both | 2189.96(1769.56,2688.95) | 666.56(530.53,818.30) | -69.56 | 6.08(5.80,6.38) | 3.10(2.84,3.37) | -1.97(-2.06,-1.88) |
| High-income North America | both | 4335.10(1924.00,10441.20) | 11889.38(7113.14,18012.04) | 174.26 | 6.68(6.48,6.89) | 17.21(16.90,17.53) | 3.30(2.44,4.17) |
| North Africa and Middle East | both | 1168717.12(912851.05,1684088.46) | 273826.68(218379.52,339303.77) | -76.57 | 706.58(705.15,708.01) | 140.92(140.33,141.50) | -5.25(-5.61,-4.90) |
| Oceania | both | 15420.85(12082.70,19243.80) | 18328.79(13659.17,23955.31) | 18.86 | 325.74(318.91,332.70) | 224.82(220.64,229.07) | -0.87(-1.25,-0.49) |
| South Asia | both | 15575728.00(12759029.58,18948015.52) | 2009030.67(1591986.20,2477867.49) | -87.10 | 3133.74(3132.02,3135.47) | 410.84(410.22,411.46) | -5.92(-6.18,-5.65) |
| Southeast Asia | both | 1965499.83(1592311.49,2638469.48) | 337721.06(278189.12,402543.99) | -82.82 | 1073.99(1072.32,1075.66) | 187.39(186.68,188.10) | -5.53(-5.59,-5.47) |
| Southern Latin America | both | 78587.93(73423.94,84190.99) | 4499.44(3749.39,5342.33) | -94.27 | 403.82(400.52,407.15) | 25.13(24.22,26.07) | -8.60(-9.13,-8.07) |
| Southern Sub-Saharan Africa | both | 545001.45(443976.46,683979.69) | 334061.55(252512.15,422783.64) | -38.70 | 1767.99(1761.92,1774.07) | 1070.60(1066.14,1075.08) | -0.91(-1.23,-0.58) |
| Tropical Latin America | both | 718529.01(639468.25,802761.09) | 38705.75(30948.87,47941.68) | -94.61 | 1290.46(1287.16,1293.77) | 64.27(63.55,65.00) | -9.75(-10.36,-9.12) |
| Western Europe | both | 3445.44(2901.57,4342.91) | 4191.47(1792.72,8008.90) | 21.65 | 4.85(4.68,5.02) | 5.83(5.65,6.02) | 2.29(1.66,2.92) |
| Western Sub-Saharan Africa | both | 4176634.12(3121315.02,5821528.61) | 2167604.78(1534495.80,2840530.76) | -48.10 | 3438.08(3434.09,3442.08) | 801.66(800.41,802.90) | -4.52(-4.71,-4.34) |
| Afghanistan | both | 90753.84(60067.61,133300.72) | 61723.40(42414.12,85879.72) | -31.99 | 1559.01(1547.75,1570.35) | 356.68(353.67,359.72) | -4.82(-5.34,-4.29) |
| Albania | both | 4027.47(3012.07,5275.59) | 221.01(137.16,333.03) | -94.51 | 334.25(322.94,345.86) | 43.06(36.55,50.44) | -7.03(-7.68,-6.36) |
| Algeria | both | 39874.13(28082.45,57498.30) | 5353.29(3927.08,7005.93) | -86.57 | 361.10(357.33,364.91) | 36.88(35.81,37.97) | -7.82(-8.23,-7.42) |
| American Samoa | both | 97.32(68.82,130.38) | 24.19(14.94,35.65) | -75.14 | 202.35(146.57,274.78) | 103.91(50.48,192.40) | -2.22(-2.53,-1.91) |
| Andorra | both | 0.09(0.02,0.15) | 0.94(0.00,3.25) | 998.35 | 1.08(0.00,65.24) | 8.05(0.15,75.70) | 8.77(7.29,10.26) |
| Angola | both | 788274.57(555621.24,1183738.83) | 173615.12(110384.22,237916.80) | -77.98 | 13322.16(13288.13,13356.26) | 1006.41(1001.16,1011.68) | -8.44(-8.79,-8.09) |
| Antigua and Barbuda | both | 62.72(52.49,77.34) | 26.64(21.96,32.04) | -57.52 | 249.25(177.36,342.18) | 101.78(56.08,172.44) | -2.99(-3.73,-2.25) |
| Argentina | both | 65142.65(60339.34,70313.95) | 3095.51(2547.37,3788.70) | -95.25 | 507.93(503.36,512.52) | 24.97(23.88,26.10) | -9.36(-9.87,-8.84) |
| Armenia | both | 1671.04(1285.23,2042.03) | 86.56(61.31,119.48) | -94.82 | 150.44(143.04,158.15) | 17.21(13.67,21.43) | -7.27(-7.75,-6.78) |
| Australia | both | 92.09(79.89,104.92) | 50.35(37.83,55.72) | -45.32 | 1.62(1.22,2.11) | 0.88(0.62,1.22) | -1.65(-2.09,-1.20) |
| Austria | both | 24.98(11.61,43.35) | 64.58(0.93,537.68) | 158.53 | 1.96(1.27,2.94) | 4.87(3.75,6.25) | 6.03(2.83,9.33) |
| Azerbaijan | both | 11595.99(8966.65,14659.52) | 1366.01(993.32,1815.67) | -88.22 | 409.71(401.65,417.91) | 63.12(59.56,66.86) | -6.80(-7.18,-6.41) |
| Bahamas | both | 244.46(200.66,294.89) | 52.60(38.93,73.35) | -78.48 | 261.31(225.04,302.25) | 59.08(41.00,83.00) | -4.68(-5.41,-3.95) |
| Bahrain | both | 299.10(237.51,372.05) | 56.46(44.48,72.09) | -81.12 | 164.85(145.36,186.39) | 17.29(12.56,23.33) | -7.09(-7.33,-6.86) |
| Bangladesh | both | 4541664.86(3686824.88,5421086.33) | 127036.09(90305.93,167762.16) | -97.20 | 7961.58(7953.55,7969.62) | 278.66(276.96,280.37) | -10.58(-10.87,-10.28) |
| Barbados | both | 134.66(111.12,160.62) | 17.25(12.44,23.69) | -87.19 | 190.18(155.03,231.57) | 33.43(17.47,58.96) | -5.83(-6.22,-5.44) |
| Belarus | both | 810.68(529.89,1056.55) | 158.16(118.67,210.60) | -80.49 | 33.27(30.82,35.87) | 10.57(8.84,12.56) | -5.16(-6.38,-3.92) |
| Belgium | both | 82.09(66.67,102.56) | 101.42(25.73,422.17) | 23.55 | 4.44(3.50,5.59) | 5.08(4.12,6.24) | 2.21(0.97,3.46) |
| Belize | both | 1417.46(1244.88,1619.48) | 322.07(255.07,405.66) | -77.28 | 1379.90(1299.03,1464.84) | 196.36(170.43,225.40) | -6.20(-7.03,-5.35) |
| Benin | both | 138424.29(83415.80,217708.37) | 41236.69(24282.09,62277.25) | -70.21 | 4370.10(4343.30,4397.04) | 535.26(529.35,541.22) | -6.65(-6.94,-6.36) |
| Bermuda | both | 6.37(4.82,7.90) | 0.80(0.60,1.00) | -87.47 | 33.73(8.71,92.94) | 6.62(0.00,77.66) | -5.53(-6.30,-4.75) |
| Bhutan | both | 3050.88(1567.65,5032.70) | 128.01(62.62,217.36) | -95.80 | 946.25(908.11,985.68) | 63.04(51.45,76.64) | -8.86(-9.08,-8.64) |
| Bolivia (Plurinational State of) | both | 146659.91(94574.33,200703.37) | 15944.56(10853.53,22853.03) | -89.13 | 3364.23(3342.19,3386.40) | 332.80(326.43,339.27) | -7.39(-7.49,-7.29) |
| Bosnia and Herzegovina | both | 156.54(102.58,227.84) | 23.91(16.07,34.05) | -84.72 | 16.29(13.82,19.11) | 5.48(3.43,8.38) | -5.62(-6.40,-4.83) |
| Botswana | both | 10225.35(6996.49,13698.87) | 7861.57(4949.98,11630.95) | -23.12 | 1209.14(1179.26,1239.62) | 755.02(732.64,777.94) | -1.01(-1.29,-0.72) |
| Brazil | both | 709576.50(631082.68,793331.75) | 34927.94(28105.24,42968.55) | -95.08 | 1326.87(1323.45,1330.29) | 58.98(58.27,59.69) | -10.06(-10.70,-9.41) |
| Brunei Darussalam | both | 14.16(9.19,22.10) | 4.09(2.33,6.53) | -71.14 | 5.54(1.76,13.50) | 1.46(0.07,7.93) | -3.53(-3.90,-3.15) |
| Bulgaria | both | 224.01(148.50,308.22) | 115.06(85.61,153.16) | -48.64 | 8.26(6.84,9.91) | 8.64(6.75,10.92) | -0.24(-0.64,0.16) |
| Burkina Faso | both | 366489.78(249746.19,506993.46) | 220565.59(143378.92,324832.98) | -39.82 | 5601.95(5579.89,5624.07) | 1555.87(1548.23,1563.54) | -3.94(-4.36,-3.52) |
| Burundi | both | 366649.76(202244.54,650460.38) | 80655.05(39932.09,139050.34) | -78.00 | 11173.50(11131.77,11215.36) | 1168.09(1158.98,1177.25) | -7.02(-7.36,-6.68) |
| Cabo Verde | both | 4679.53(3198.92,6344.09) | 167.52(115.20,249.22) | -96.42 | 2236.72(2164.04,2311.37) | 94.78(77.98,114.34) | -10.48(-10.97,-9.98) |
| Cambodia | both | 237825.94(146695.89,397357.66) | 12647.60(9222.61,17692.17) | -94.68 | 4121.04(4102.67,4139.46) | 234.55(230.11,239.06) | -10.15(-10.68,-9.62) |
| Cameroon | both | 90040.33(60302.96,125799.47) | 50632.11(31371.58,74606.69) | -43.77 | 1318.63(1308.28,1329.05) | 290.24(287.25,293.26) | -4.93(-5.28,-4.58) |
| Canada | both | 71.50(50.66,64.23) | 1064.12(103.34,2289.21) | 1388.30 | 1.16(0.89,1.49) | 16.26(15.29,17.29) | 8.60(6.54,10.69) |
| Central African Republic | both | 62904.70(39090.50,100269.82) | 67244.70(43373.15,96689.61) | 6.90 | 3970.05(3935.25,4005.09) | 2526.33(2504.88,2547.93) | -1.03(-1.43,-0.62) |
| Chad | both | 238124.53(155832.43,353399.69) | 148725.19(104725.65,207088.99) | -37.54 | 6106.02(6077.22,6134.93) | 1234.70(1227.40,1242.04) | -5.14(-5.30,-4.99) |
| Chile | both | 10598.41(9682.60,11574.28) | 1204.96(957.54,1496.46) | -88.63 | 183.71(179.53,187.96) | 25.66(23.88,27.55) | -5.97(-6.64,-5.30) |
| China | both | 2188333.03(1804410.70,2650884.46) | 42061.57(33309.90,51821.86) | -98.08 | 575.69(574.83,576.55) | 19.21(19.00,19.42) | -12.60(-13.39,-11.80) |
| Colombia | both | 100857.38(85088.56,116709.64) | 29544.94(20172.57,41934.97) | -70.71 | 651.35(646.70,656.03) | 190.49(187.71,193.31) | -3.27(-3.89,-2.65) |
| Comoros | both | 13119.75(8769.92,17895.50) | 2234.35(1550.23,3246.81) | -82.97 | 4952.98(4857.13,5050.36) | 884.70(844.30,926.61) | -5.68(-5.85,-5.50) |
| Congo | both | 18785.41(13028.93,25232.13) | 8111.59(5524.60,11174.62) | -56.82 | 1552.14(1527.64,1576.94) | 417.68(407.77,427.78) | -4.56(-5.19,-3.92) |
| Cook Islands | both | 1.47(0.96,2.17) | 0.16(0.10,0.22) | -89.38 | 9.73(0.02,92.17) | 2.81(0.00,154.84) | -7.58(-8.85,-6.30) |
| Costa Rica | both | 807.24(705.52,917.32) | 351.05(76.68,548.19) | -56.51 | 59.91(55.39,64.71) | 30.75(27.45,34.40) | -0.08(-0.92,0.77) |
| Croatia | both | 30.35(17.93,47.54) | 13.79(8.97,20.39) | -54.56 | 3.70(2.48,5.33) | 2.39(1.23,4.28) | -2.35(-3.13,-1.56) |
| Cuba | both | 1237.48(1124.68,1369.83) | 280.54(223.63,348.21) | -77.33 | 38.72(36.24,41.33) | 12.60(10.84,14.59) | -3.78(-4.99,-2.56) |
| Cyprus | both | 9.29(6.17,13.68) | 1.05(0.39,2.13) | -88.66 | 4.85(2.23,9.55) | 0.48(0.01,3.07) | -7.02(-7.59,-6.46) |
| Czechia | both | 281.74(233.80,365.45) | 260.21(213.37,324.58) | -7.64 | 12.80(11.19,14.60) | 13.69(11.90,15.70) | -0.08(-0.46,0.30) |
| C?te d'Ivoire | both | 148170.77(101783.77,203618.29) | 46637.21(30668.98,66192.89) | -68.52 | 1902.41(1891.10,1913.77) | 309.27(306.03,312.54) | -5.78(-6.12,-5.44) |
| Democratic People's Republic of Korea | both | 38248.60(26343.94,54859.45) | 1731.63(1108.82,2541.10) | -95.47 | 498.42(492.77,504.12) | 31.16(29.45,32.94) | -18.18(-24.84,-10.92) |
| Democratic Republic of the Congo | both | 1017936.21(646096.00,1562134.51) | 289198.30(155287.22,476283.04) | -71.59 | 4218.38(4208.94,4227.84) | 681.64(678.87,684.41) | -5.58(-6.39,-4.77) |
| Denmark | both | 27.65(18.66,39.83) | 13.21(6.85,99.32) | -52.25 | 3.08(2.02,4.56) | 1.34(0.70,2.39) | -2.52(-3.71,-1.30) |
| Djibouti | both | 9407.51(6485.87,13008.78) | 4990.11(3320.86,7129.00) | -46.96 | 4494.93(4390.59,4601.25) | 1086.87(1053.06,1121.52) | -4.61(-5.15,-4.07) |
| Dominica | both | 70.86(51.67,95.96) | 22.60(14.46,34.59) | -68.11 | 223.40(166.98,294.35) | 173.53(99.32,284.57) | -0.51(-1.36,0.34) |
| Dominican Republic | both | 85640.46(68565.41,102619.66) | 10324.35(6112.83,15516.70) | -87.94 | 2418.84(2400.12,2437.67) | 285.73(279.47,292.09) | -6.42(-6.98,-5.85) |
| Ecuador | both | 52880.33(47930.74,58844.28) | 7005.33(5062.13,9556.34) | -86.75 | 971.83(961.74,982.01) | 127.68(124.35,131.08) | -7.03(-8.08,-5.97) |
| Egypt | both | 81591.14(61553.67,106159.48) | 23370.31(17642.75,30051.01) | -71.36 | 291.94(289.70,294.20) | 53.39(52.62,54.16) | -4.38(-4.81,-3.95) |
| El Salvador | both | 22475.64(18220.15,27358.12) | 1876.64(1273.33,2607.46) | -91.65 | 795.65(783.67,807.79) | 89.63(85.09,94.36) | -7.14(-7.34,-6.95) |
| Equatorial Guinea | both | 7068.27(4648.92,10087.87) | 1870.21(958.25,3126.64) | -73.54 | 2686.20(2614.41,2759.56) | 329.67(313.83,346.14) | -7.93(-8.43,-7.42) |
| Eritrea | both | 266557.79(177472.33,363804.14) | 47706.42(31906.85,67372.23) | -82.10 | 13809.17(13748.03,13870.52) | 1556.08(1539.63,1572.66) | -6.78(-7.11,-6.46) |
| Estonia | both | 280.15(239.50,329.76) | 15.26(12.69,18.64) | -94.55 | 74.83(65.46,85.22) | 6.46(3.33,11.49) | -8.85(-10.20,-7.48) |
| Eswatini | both | 8453.15(5937.61,11381.28) | 3929.60(2579.22,5746.16) | -53.51 | 1524.20(1483.89,1565.38) | 787.86(759.49,817.07) | -1.64(-2.04,-1.25) |
| Ethiopia | both | 2649414.57(1922135.52,3688724.20) | 577062.32(420547.51,747189.50) | -78.22 | 8988.77(8976.35,9001.21) | 1158.33(1154.93,1161.74) | -7.21(-7.64,-6.76) |
| Fiji | both | 823.77(587.86,1119.06) | 501.38(329.35,757.53) | -39.14 | 209.22(191.34,228.40) | 89.03(77.46,101.93) | -2.74(-2.97,-2.51) |
| Finland | both | 18.57(9.88,30.47) | 4.57(1.08,5.31) | -75.38 | 2.04(1.21,3.26) | 0.55(0.16,1.49) | -3.02(-4.26,-1.77) |
| France | both | 1898.67(1721.53,2098.91) | 1004.62(567.01,3164.57) | -47.09 | 15.59(14.85,16.37) | 8.08(7.54,8.65) | -1.45(-2.19,-0.70) |
| Gabon | both | 4323.48(2953.02,6177.92) | 1583.27(889.42,2589.60) | -63.38 | 835.21(807.18,864.03) | 230.48(217.88,243.64) | -3.32(-3.90,-2.74) |
| Gambia | both | 21103.08(14560.42,28329.99) | 6567.04(4628.76,9127.18) | -68.88 | 3761.63(3702.53,3821.46) | 566.43(550.47,582.75) | -6.40(-6.73,-6.07) |
| Georgia | both | 154.72(102.39,221.23) | 12.40(7.66,16.28) | -91.99 | 12.08(10.19,14.23) | 1.62(0.79,3.00) | -6.70(-7.59,-5.79) |
| Germany | both | 184.58(96.43,294.06) | 1191.79(48.72,2454.94) | 545.69 | 1.43(1.23,1.66) | 9.77(9.22,10.35) | 7.37(5.85,8.92) |
| Ghana | both | 318176.24(200749.86,479410.19) | 83321.52(50202.26,124748.81) | -73.81 | 3726.50(3711.22,3741.83) | 564.59(560.22,569.00) | -5.49(-5.74,-5.24) |
| Greece | both | 0.57(0.39,0.85) | 27.50(0.37,529.67) | 4738.40 | 0.04(0.00,0.36) | 1.86(1.22,2.76) | 18.49(14.79,22.31) |
| Greenland | both | 1.09(0.79,1.53) | 0.36(0.15,1.52) | -67.17 | 6.44(0.18,42.51) | 3.02(0.00,46.63) | -2.28(-3.04,-1.50) |
| Grenada | both | 151.44(119.44,190.83) | 36.55(28.40,46.74) | -75.87 | 316.35(257.57,385.40) | 127.73(82.05,191.75) | -2.94(-4.11,-1.76) |
| Guam | both | 80.84(62.13,104.74) | 33.24(23.22,46.87) | -58.88 | 88.98(63.76,122.05) | 39.20(21.16,67.64) | -1.57(-2.00,-1.13) |
| Guatemala | both | 146566.37(130795.53,163947.10) | 24645.69(18997.64,32174.70) | -83.18 | 2607.54(2591.63,2623.53) | 379.38(373.47,385.37) | -5.56(-5.98,-5.15) |
| Guinea | both | 242381.99(162139.96,346692.16) | 99777.18(62644.65,147001.59) | -58.83 | 6332.12(6302.01,6362.34) | 1313.33(1303.86,1322.86) | -4.71(-5.05,-4.36) |
| Guinea-Bissau | both | 28919.48(17936.46,45789.54) | 3402.21(2046.06,5258.14) | -88.24 | 4932.53(4866.14,4999.63) | 324.95(312.79,337.48) | -8.70(-9.01,-8.38) |
| Guyana | both | 8737.17(7048.23,10690.36) | 1046.63(793.23,1351.44) | -88.02 | 2527.26(2470.97,2584.62) | 472.52(442.31,504.35) | -4.39(-4.85,-3.94) |
| Haiti | both | 161787.82(119536.82,222227.40) | 64705.54(44112.09,94234.67) | -60.01 | 4531.33(4506.43,4556.34) | 1234.57(1224.03,1245.19) | -4.08(-4.31,-3.85) |
| Honduras | both | 16394.20(12787.18,20506.23) | 2071.54(1086.81,3348.02) | -87.36 | 605.58(595.13,616.19) | 53.35(50.71,56.11) | -7.76(-7.88,-7.64) |
| Hungary | both | 128.13(94.45,172.34) | 90.05(71.63,109.39) | -29.72 | 6.70(5.53,8.05) | 5.50(4.27,6.99) | -0.37(-1.18,0.44) |
| Iceland | both | 1.02(0.58,1.57) | 1.59(0.35,13.15) | 55.23 | 1.49(0.03,10.84) | 2.17(0.15,11.28) | 2.37(1.12,3.63) |
| India | both | 9736773.04(7613663.81,12271331.68) | 1478986.48(1158947.92,1866900.63) | -84.81 | 2629.56(2627.72,2631.39) | 439.45(438.68,440.22) | -5.11(-5.48,-4.74) |
| Indonesia | both | 938814.36(760817.69,1206382.64) | 186749.81(148654.51,231108.03) | -80.11 | 1347.69(1344.67,1350.71) | 265.86(264.51,267.22) | -4.98(-5.04,-4.92) |
| Iran (Islamic Republic of) | both | 107040.81(88990.27,134349.83) | 4573.84(3344.43,6054.13) | -95.73 | 418.71(415.98,421.46) | 23.49(22.74,24.25) | -8.21(-8.60,-7.81) |
| Iraq | both | 40380.61(30361.16,55409.41) | 8021.18(6074.00,10797.72) | -80.14 | 425.91(421.45,430.41) | 60.33(58.90,61.80) | -6.21(-6.70,-5.72) |
| Ireland | both | 18.27(11.24,26.84) | 69.10(1.66,285.95) | 278.24 | 2.34(1.38,3.74) | 6.48(5.04,8.29) | 6.48(4.45,8.55) |
| Israel | both | 45.53(33.30,60.51) | 119.33(12.73,530.09) | 162.09 | 2.98(2.15,4.04) | 4.47(3.70,5.38) | 4.12(2.93,5.33) |
| Italy | both | 180.35(103.55,292.98) | 540.81(72.55,1494.90) | 199.86 | 2.26(1.94,2.62) | 6.65(6.09,7.25) | 6.61(5.22,8.01) |
| Jamaica | both | 7398.46(6338.55,8590.49) | 396.10(295.99,521.01) | -94.65 | 643.20(625.09,661.73) | 57.18(50.60,64.45) | -6.85(-8.02,-5.65) |
| Japan | both | 1252.59(957.98,1650.63) | 604.23(476.96,753.35) | -51.76 | 5.60(5.26,5.96) | 4.00(3.65,4.37) | -1.05(-1.31,-0.78) |
| Jordan | both | 1240.75(991.61,1481.04) | 408.53(322.81,518.08) | -67.07 | 63.61(59.84,67.58) | 11.57(10.39,12.86) | -5.40(-5.59,-5.20) |
| Kazakhstan | both | 4873.10(3888.24,5868.18) | 1216.88(965.04,1482.55) | -75.03 | 80.96(78.49,83.48) | 17.80(16.70,18.95) | -5.74(-6.25,-5.23) |
| Kenya | both | 457384.02(338286.47,601259.15) | 182277.86(140883.50,236202.69) | -60.15 | 2996.87(2986.51,3007.26) | 863.18(858.50,867.87) | -3.49(-3.74,-3.25) |
| Kiribati | both | 1686.62(1181.86,2252.26) | 620.25(402.63,883.65) | -63.23 | 2271.44(2107.42,2445.84) | 698.15(616.78,787.97) | -3.83(-3.99,-3.67) |
| Kuwait | both | 178.61(134.70,229.35) | 10.17(7.71,13.59) | -94.31 | 29.95(25.30,35.21) | 1.12(0.48,2.27) | -10.84(-11.77,-9.90) |
| Kyrgyzstan | both | 1778.63(1486.64,2129.83) | 157.43(128.55,192.95) | -91.15 | 92.45(87.85,97.23) | 6.32(5.29,7.51) | -9.54(-10.40,-8.68) |
| Lao People's Democratic Republic | both | 84126.64(54972.98,145806.84) | 7968.86(5274.03,11795.01) | -90.53 | 3888.13(3859.69,3916.74) | 326.66(319.18,334.30) | -8.10(-8.36,-7.84) |
| Latvia | both | 367.85(308.10,448.37) | 25.32(21.16,30.10) | -93.12 | 62.09(55.45,69.33) | 8.37(5.14,12.96) | -8.01(-9.32,-6.67) |
| Lebanon | both | 1132.93(823.35,1555.65) | 210.73(124.00,327.01) | -81.40 | 103.35(97.02,109.99) | 18.25(15.75,21.06) | -5.03(-5.70,-4.35) |
| Lesotho | both | 15920.49(11522.73,20873.78) | 8517.95(5338.59,12810.67) | -46.50 | 1821.10(1788.07,1854.63) | 1177.33(1148.78,1206.45) | -1.15(-1.46,-0.85) |
| Liberia | both | 129477.97(89126.00,187159.64) | 17998.06(10763.80,27495.75) | -86.10 | 7394.73(7344.47,7445.27) | 657.74(646.43,669.20) | -8.32(-9.04,-7.60) |
| Libya | both | 2108.30(1553.56,2738.38) | 746.82(538.00,979.78) | -64.58 | 106.87(101.93,111.99) | 54.62(50.29,59.23) | -0.83(-1.30,-0.37) |
| Lithuania | both | 445.91(372.93,534.52) | 29.26(24.70,34.23) | -93.44 | 50.89(45.94,56.26) | 7.26(4.71,10.78) | -7.23(-8.50,-5.94) |
| Luxembourg | both | 4.04(3.25,5.27) | 1.65(1.15,2.09) | -59.13 | 5.32(1.31,15.70) | 1.41(0.08,7.47) | -4.14(-5.04,-3.23) |
| Madagascar | both | 838112.21(665991.72,1001367.69) | 279442.47(191267.27,390438.72) | -66.66 | 11764.99(11734.93,11795.11) | 2199.98(2190.82,2209.18) | -4.75(-5.01,-4.48) |
| Malawi | both | 467344.25(319822.58,670340.40) | 93043.75(57557.34,128241.35) | -80.09 | 6969.23(6944.97,6993.56) | 994.65(987.17,1002.18) | -6.43(-6.81,-6.04) |
| Malaysia | both | 17887.87(12914.73,24140.02) | 7515.32(5337.36,10256.44) | -57.99 | 252.17(248.10,256.29) | 99.43(96.93,101.98) | -3.13(-3.30,-2.95) |
| Maldives | both | 2977.46(1951.27,4728.63) | 114.85(83.65,151.89) | -96.14 | 2370.32(2277.07,2466.57) | 123.11(99.87,150.33) | -9.10(-9.79,-8.41) |
| Mali | both | 562126.02(374115.15,811575.87) | 558290.64(350318.97,808542.15) | -0.68 | 10400.98(10368.47,10433.57) | 3810.80(3799.33,3822.30) | -3.08(-3.47,-2.69) |
| Malta | both | 2.41(1.35,3.90) | 0.55(0.14,0.77) | -77.18 | 3.07(0.48,10.91) | 0.84(0.00,9.11) | -4.82(-6.14,-3.49) |
| Marshall Islands | both | 84.53(50.02,128.09) | 33.21(21.22,50.96) | -60.71 | 210.62(152.05,285.89) | 95.78(53.45,161.05) | -2.77(-3.39,-2.14) |
| Mauritania | both | 33527.81(21219.93,54712.87) | 5221.74(3514.91,7843.97) | -84.43 | 2652.27(2618.04,2686.86) | 237.70(230.24,245.35) | -7.56(-7.72,-7.39) |
| Mauritius | both | 1435.95(1216.42,1704.26) | 233.01(177.98,304.94) | -83.77 | 412.51(388.69,437.51) | 113.35(97.48,131.18) | -3.40(-3.75,-3.05) |
| Mexico | both | 568265.89(512119.92,635001.66) | 44934.61(34504.13,58213.24) | -92.09 | 1315.62(1311.62,1319.63) | 119.54(118.24,120.85) | -7.35(-7.51,-7.19) |
| Micronesia (Federated States of) | both | 289.08(183.54,421.47) | 38.93(25.10,56.60) | -86.53 | 344.93(290.89,406.74) | 69.37(41.06,111.21) | -5.06(-5.23,-4.89) |
| Monaco | both | 0.13(0.01,0.74) | 0.40(0.01,1.33) | 205.63 | 3.56(0.00,159.38) | 7.76(0.00,116.66) | 2.83(2.03,3.64) |
| Mongolia | both | 909.21(545.22,1483.42) | 78.08(45.42,129.72) | -91.41 | 86.72(80.69,93.10) | 6.16(4.74,7.90) | -9.34(-9.83,-8.85) |
| Montenegro | both | 11.03(8.26,14.63) | 4.60(3.05,6.52) | -58.33 | 6.15(2.73,12.13) | 4.24(1.21,11.17) | -0.68(-2.20,0.86) |
| Morocco | both | 40246.94(27731.75,59476.94) | 3510.94(2348.65,5215.69) | -91.28 | 339.42(335.78,343.10) | 32.76(31.57,33.98) | -7.23(-8.00,-6.45) |
| Mozambique | both | 678625.97(440430.35,996257.84) | 160728.66(104064.77,234233.53) | -76.32 | 8810.04(8785.32,8834.82) | 891.17(886.06,896.29) | -7.38(-7.57,-7.18) |
| Myanmar | both | 208989.21(137839.46,326325.14) | 19847.63(13806.33,28291.48) | -90.50 | 1249.44(1243.37,1255.54) | 113.24(111.48,115.02) | -7.95(-8.28,-7.62) |
| Namibia | both | 17552.95(12751.94,23557.79) | 8154.84(5432.34,12195.41) | -53.54 | 2158.22(2119.14,2197.86) | 733.11(713.13,753.53) | -2.87(-3.20,-2.54) |
| Nauru | both | 27.30(18.16,38.83) | 13.21(8.35,19.83) | -51.62 | 221.91(102.80,426.62) | 127.31(38.59,319.86) | -1.90(-2.96,-0.83) |
| Nepal | both | 556726.40(384759.16,810854.21) | 30638.67(20718.75,44643.44) | -94.50 | 4960.69(4945.60,4975.81) | 324.16(320.29,328.06) | -8.47(-8.64,-8.29) |
| Netherlands | both | 258.07(92.49,910.26) | 396.29(46.93,1044.38) | 53.56 | 9.32(8.21,10.55) | 14.29(12.91,15.80) | 2.55(1.99,3.10) |
| New Zealand | both | 3.45(2.77,4.02) | 5.79(0.88,102.08) | 67.69 | 0.37(0.07,1.21) | 0.56(0.19,1.35) | 4.61(3.07,6.18) |
| Nicaragua | both | 25305.53(19949.96,32561.04) | 3451.41(2229.03,5094.52) | -86.36 | 1147.50(1131.78,1163.39) | 134.25(128.91,139.77) | -7.28(-7.72,-6.85) |
| Niger | both | 502995.71(312341.50,824490.31) | 147401.50(99570.42,210961.83) | -70.70 | 8609.53(8580.14,8639.01) | 844.52(839.39,849.66) | -7.97(-8.41,-7.52) |
| Nigeria | both | 944017.64(704769.90,1378201.44) | 459526.27(332046.38,603917.69) | -51.32 | 1593.46(1589.44,1597.50) | 366.04(364.81,367.28) | -4.47(-4.86,-4.07) |
| Niue | both | 2.51(1.67,3.60) | 2.24(1.63,3.05) | -10.83 | 154.82(4.14,914.04) | 409.52(18.30,2136.44) | -0.54(-1.86,0.80) |
| North Macedonia | both | 184.13(136.25,244.67) | 23.36(15.57,34.14) | -87.31 | 32.96(27.96,38.67) | 6.48(3.81,10.43) | -4.56(-4.85,-4.26) |
| Northern Mariana Islands | both | 14.50(9.50,20.91) | 6.03(4.40,8.31) | -58.39 | 54.08(22.14,116.54) | 31.74(6.01,102.14) | -0.99(-1.29,-0.70) |
| Norway | both | 14.38(7.84,23.91) | 47.96(5.84,158.10) | 233.45 | 1.70(0.92,2.95) | 4.81(3.52,6.50) | 7.01(5.69,8.35) |
| Oman | both | 3813.40(2662.39,5766.69) | 673.18(462.25,950.65) | -82.35 | 372.00(358.91,385.48) | 52.38(48.12,56.94) | -5.16(-5.78,-4.54) |
| Pakistan | both | 737512.83(569669.41,930635.99) | 372241.42(277656.61,494504.95) | -49.53 | 1123.37(1120.40,1126.34) | 375.79(374.42,377.17) | -2.55(-2.95,-2.15) |
| Palau | both | 9.75(6.41,14.37) | 2.48(1.67,3.59) | -74.58 | 114.92(35.83,289.55) | 42.80(1.33,249.26) | -2.56(-2.84,-2.27) |
| Palestine | both | 1274.59(897.64,1771.63) | 263.84(190.58,357.34) | -79.30 | 107.67(101.31,114.34) | 13.08(11.39,14.98) | -6.54(-7.04,-6.04) |
| Panama | both | 5525.01(4675.40,6494.72) | 2754.58(2021.42,3577.84) | -50.14 | 469.61(454.37,485.26) | 115.60(108.95,122.57) | -5.42(-6.14,-4.70) |
| Papua New Guinea | both | 8958.61(6526.27,11846.21) | 14945.28(10901.28,19927.22) | 66.83 | 314.25(305.88,322.80) | 242.91(238.00,247.90) | -0.50(-0.98,-0.03) |
| Paraguay | both | 8952.51(7034.08,11437.02) | 3777.81(2553.13,5186.59) | -57.80 | 379.80(370.41,389.38) | 200.15(193.56,206.93) | -2.31(-2.98,-1.63) |
| Peru | both | 179463.51(137018.43,232810.58) | 15030.18(9562.03,21471.84) | -91.62 | 1495.65(1486.97,1504.37) | 118.17(115.91,120.46) | -8.43(-8.89,-7.97) |
| Philippines | both | 290119.71(245033.34,365150.62) | 75695.59(61378.92,91949.34) | -73.91 | 971.11(967.07,975.17) | 205.02(203.37,206.69) | -4.38(-4.67,-4.08) |
| Poland | both | 564.01(396.32,828.57) | 177.12(140.40,233.92) | -68.60 | 6.75(6.18,7.36) | 3.04(2.57,3.57) | -3.21(-3.75,-2.68) |
| Portugal | both | 139.34(112.46,172.58) | 21.81(16.78,26.75) | -84.35 | 7.98(6.66,9.51) | 1.62(0.97,2.56) | -5.32(-6.55,-4.08) |
| Puerto Rico | both | 928.21(832.32,1035.53) | 127.37(105.13,154.58) | -86.28 | 83.20(77.17,89.61) | 35.94(29.13,43.93) | -3.08(-4.29,-1.85) |
| Qatar | both | 44.66(32.76,59.96) | 32.36(24.24,43.58) | -27.54 | 26.10(18.40,36.29) | 4.89(3.14,7.34) | -4.68(-5.19,-4.17) |
| Republic of Korea | both | 782.93(604.13,998.13) | 53.58(38.60,68.11) | -93.16 | 6.30(5.81,6.82) | 0.81(0.57,1.12) | -6.20(-6.44,-5.97) |
| Republic of Moldova | both | 870.66(725.22,1025.98) | 72.84(55.09,96.15) | -91.63 | 67.05(62.28,72.10) | 15.36(11.78,19.74) | -6.04(-7.52,-4.53) |
| Romania | both | 1244.74(1047.44,1542.11) | 462.16(377.25,557.07) | -62.87 | 23.21(21.81,24.68) | 13.97(12.56,15.51) | -1.74(-2.42,-1.07) |
| Russian Federation | both | 13808.77(9899.95,18617.17) | 2106.34(1579.59,2802.51) | -84.75 | 39.96(39.23,40.70) | 7.74(7.35,8.13) | -6.21(-6.86,-5.57) |
| Rwanda | both | 273563.18(183267.52,381716.99) | 40731.12(27616.82,57264.94) | -85.11 | 6480.49(6452.61,6508.47) | 696.03(688.20,703.93) | -8.64(-9.41,-7.86) |
| Saint Kitts and Nevis | both | 176.62(152.68,201.57) | 39.84(30.43,52.83) | -77.44 | 963.79(798.74,1154.98) | 260.91(163.26,399.74) | -3.88(-4.90,-2.86) |
| Saint Lucia | both | 298.81(243.28,359.73) | 38.48(27.32,53.80) | -87.12 | 455.56(396.01,522.04) | 106.65(69.77,157.60) | -4.58(-5.43,-3.71) |
| Saint Vincent and the Grenadines | both | 459.06(369.61,563.25) | 78.93(58.46,104.02) | -82.81 | 994.79(892.43,1106.45) | 267.01(199.95,350.76) | -4.37(-5.37,-3.35) |
| Samoa | both | 290.65(186.48,427.59) | 111.42(70.55,160.31) | -61.67 | 187.58(156.53,223.46) | 60.00(44.05,80.28) | -3.51(-3.60,-3.43) |
| San Marino | both | 0.06(0.03,0.09) | 0.13(0.00,1.00) | 117.97 | 1.58(0.00,146.80) | 2.77(0.00,141.38) | 2.60(1.42,3.79) |
| Sao Tome and Principe | both | 3487.27(2571.70,4455.28) | 267.11(175.58,384.58) | -92.34 | 4920.53(4730.05,5117.09) | 356.95(312.26,406.57) | -8.21(-8.76,-7.66) |
| Saudi Arabia | both | 36114.10(25231.66,50920.76) | 3766.07(2511.27,5551.98) | -89.57 | 483.23(477.73,488.78) | 53.72(51.90,55.59) | -6.58(-6.79,-6.36) |
| Senegal | both | 127669.44(84496.21,182706.80) | 17885.59(10705.60,26428.87) | -85.99 | 2725.22(2707.49,2743.05) | 237.82(233.80,241.89) | -7.78(-8.28,-7.27) |
| Serbia | both | 268.99(198.92,366.70) | 65.54(43.08,89.54) | -75.64 | 11.36(9.87,13.02) | 6.02(4.57,7.81) | -1.13(-1.97,-0.27) |
| Seychelles | both | 27.60(21.18,35.16) | 14.37(10.92,18.83) | -47.92 | 106.75(67.63,161.89) | 54.86(27.97,98.66) | -1.15(-1.57,-0.73) |
| Sierra Leone | both | 229912.70(153720.81,316455.08) | 250762.36(156067.47,376223.61) | 9.07 | 8651.32(8609.01,8693.81) | 4819.92(4796.63,4843.31) | -0.85(-1.41,-0.29) |
| Singapore | both | 140.28(74.03,223.61) | 4.67(2.20,13.63) | -96.67 | 20.84(17.04,25.29) | 0.44(0.10,1.29) | -12.39(-13.20,-11.56) |
| Slovakia | both | 119.20(93.01,153.61) | 46.48(34.90,60.20) | -61.01 | 9.16(7.47,11.15) | 4.85(3.43,6.70) | -1.64(-2.27,-1.00) |
| Slovenia | both | 10.71(6.82,16.06) | 8.00(5.74,10.76) | -25.34 | 2.96(1.38,5.62) | 2.38(0.91,5.25) | -1.31(-1.75,-0.87) |
| Solomon Islands | both | 1317.17(887.03,1854.48) | 761.96(547.64,1017.38) | -42.15 | 437.20(405.63,470.79) | 169.76(153.68,187.15) | -2.74(-2.95,-2.52) |
| Somalia | both | 446950.74(281986.12,647339.31) | 303510.20(190605.51,438988.19) | -32.09 | 9927.76(9895.00,9960.61) | 2489.84(2480.17,2499.53) | -3.09(-5.89,-0.21) |
| South Africa | both | 384843.42(312421.24,487205.42) | 133032.89(100620.18,165562.25) | -65.43 | 1759.07(1751.55,1766.63) | 741.15(736.49,745.84) | -1.93(-2.26,-1.59) |
| South Sudan | both | 435079.12(294962.96,649177.61) | 239422.16(158187.55,365706.67) | -44.97 | 14051.70(14004.34,14099.19) | 4868.93(4846.66,4891.29) | -3.48(-4.22,-2.74) |
| Spain | both | 56.81(49.90,64.21) | 31.71(26.21,37.33) | -44.17 | 0.86(0.64,1.14) | 0.55(0.36,0.80) | -1.34(-1.80,-0.88) |
| Sri Lanka | both | 19730.40(15286.49,24649.39) | 7067.27(4789.57,9983.64) | -64.18 | 355.57(349.99,361.22) | 151.99(148.05,156.01) | -2.79(-3.03,-2.55) |
| Sudan | both | 358845.17(219438.67,621573.41) | 62679.33(42104.51,88349.62) | -82.53 | 3004.94(2993.35,3016.57) | 334.38(331.39,337.40) | -6.71(-7.08,-6.34) |
| Suriname | both | 1310.84(969.85,1684.76) | 255.98(176.89,363.33) | -80.47 | 925.97(872.23,982.39) | 159.07(137.25,183.59) | -5.99(-6.38,-5.58) |
| Sweden | both | 32.29(19.11,48.94) | 79.21(8.73,396.28) | 145.33 | 1.89(1.28,2.71) | 4.19(3.32,5.26) | 5.87(4.48,7.28) |
| Switzerland | both | 35.11(27.96,47.31) | 58.16(9.62,267.54) | 65.67 | 2.87(1.97,4.08) | 4.20(3.18,5.49) | 3.54(2.46,4.64) |
| Syrian Arab Republic | both | 38851.82(29768.94,49165.39) | 5910.90(4261.13,7788.72) | -84.79 | 599.80(593.35,606.30) | 171.61(166.52,176.83) | -3.16(-3.80,-2.52) |
| Taiwan (Province of China) | both | 3346.76(3036.97,3704.15) | 330.68(276.82,392.54) | -90.12 | 58.64(56.43,60.93) | 11.93(10.56,13.44) | -5.69(-7.37,-3.97) |
| Tajikistan | both | 9917.54(7663.07,12878.37) | 3000.09(2063.84,4307.95) | -69.75 | 325.70(318.46,333.07) | 70.00(67.23,72.85) | -6.26(-7.40,-5.12) |
| Thailand | both | 30257.65(22949.07,38743.67) | 6247.16(4822.80,8097.26) | -79.35 | 174.97(172.78,177.19) | 66.55(64.70,68.44) | -2.30(-2.74,-1.86) |
| Timor-Leste | both | 29025.13(16636.64,54408.48) | 4581.31(3362.35,6534.44) | -84.22 | 7157.20(7067.99,7247.35) | 803.32(777.79,829.51) | -7.59(-8.03,-7.14) |
| Togo | both | 46771.69(30531.05,67064.47) | 9193.97(5932.38,13815.97) | -80.34 | 2249.79(2226.53,2273.24) | 234.44(228.92,240.06) | -7.39(-7.66,-7.12) |
| Tokelau | both | 3.01(1.69,4.64) | 2.11(1.41,2.92) | -29.99 | 200.72(4.93,1216.84) | 443.96(15.42,2414.25) | -2.99(-5.17,-0.76) |
| Tonga | both | 216.49(144.85,302.68) | 101.79(71.36,144.36) | -52.98 | 220.95(178.01,271.99) | 113.71(81.71,154.77) | -1.84(-2.18,-1.49) |
| Trinidad and Tobago | both | 1475.37(1225.60,1745.74) | 209.21(160.43,270.38) | -85.82 | 336.52(317.34,356.58) | 74.28(62.95,87.15) | -5.28(-5.49,-5.06) |
| Tunisia | both | 4999.53(3640.71,6708.76) | 345.37(234.61,488.74) | -93.09 | 154.67(150.09,159.36) | 11.67(10.33,13.13) | -8.05(-8.48,-7.62) |
| Türkiye | both | 101286.41(74783.72,141156.85) | 6516.58(4700.58,9201.63) | -93.57 | 443.07(440.11,446.05) | 31.06(30.18,31.97) | -8.39(-8.68,-8.08) |
| Turkmenistan | both | 3153.75(2387.67,3909.36) | 464.06(335.97,611.01) | -85.29 | 158.82(152.51,165.34) | 26.15(23.60,28.93) | -6.05(-6.41,-5.68) |
| Tuvalu | both | 59.62(40.10,83.16) | 6.13(4.01,9.13) | -89.71 | 705.90(469.81,1035.10) | 75.74(13.28,254.69) | -6.72(-6.93,-6.51) |
| Uganda | both | 534309.76(344448.94,766463.00) | 211199.33(117911.76,326613.11) | -60.47 | 4057.74(4044.61,4070.91) | 813.96(809.85,818.09) | -5.13(-5.36,-4.89) |
| Ukraine | both | 18914.62(13850.17,25115.82) | 4984.14(3199.38,7231.76) | -73.65 | 160.83(158.27,163.41) | 91.47(88.57,94.44) | -1.67(-2.09,-1.24) |
| United Arab Emirates | both | 1881.39(1279.37,2596.28) | 997.31(561.25,1657.70) | -46.99 | 287.66(273.51,302.39) | 72.94(68.21,77.92) | -4.09(-4.33,-3.85) |
| United Kingdom | both | 408.32(301.32,568.83) | 409.40(80.10,1121.28) | 0.27 | 3.48(3.14,3.85) | 3.32(3.00,3.66) | 2.84(1.74,3.96) |
| United Republic of Tanzania | both | 1017841.54(723475.59,1362207.55) | 268654.85(173835.74,389693.17) | -73.61 | 6329.16(6315.10,6343.25) | 812.97(809.22,816.72) | -6.58(-6.80,-6.36) |
| United States of America | both | 4262.41(1865.26,10380.25) | 10824.72(6798.20,16691.12) | 153.96 | 7.25(7.03,7.48) | 17.30(16.97,17.64) | 3.04(2.21,3.88) |
| United States Virgin Islands | both | 73.90(49.80,104.66) | 5.48(3.32,8.47) | -92.58 | 192.70(146.18,250.54) | 33.96(7.91,96.83) | -4.92(-5.37,-4.46) |
| Uruguay | both | 2843.14(2575.49,3134.81) | 198.72(155.11,246.85) | -93.01 | 313.04(300.60,325.90) | 24.48(20.38,29.21) | -7.88(-8.62,-7.14) |
| Uzbekistan | both | 16952.04(12194.05,21961.26) | 1452.22(1083.10,1979.83) | -91.43 | 148.38(145.82,150.98) | 12.13(11.47,12.81) | -9.41(-9.88,-8.95) |
| Vanuatu | both | 473.84(294.89,700.39) | 301.86(179.31,457.31) | -36.30 | 350.12(308.16,396.65) | 128.52(108.39,151.55) | -3.00(-3.58,-2.40) |
| Venezuela (Bolivarian Republic of) | both | 61569.08(56989.33,66870.39) | 16597.13(11992.13,22529.89) | -73.04 | 601.43(595.71,607.20) | 166.90(163.64,170.21) | -5.10(-6.19,-3.99) |
| Viet Nam | both | 101438.63(70735.39,144640.93) | 8567.22(6007.04,12105.50) | -91.55 | 356.25(353.82,358.69) | 34.00(33.19,34.82) | -7.59(-7.78,-7.39) |
| Yemen | both | 216119.56(144469.01,325873.12) | 84400.64(58469.53,113735.22) | -60.95 | 2313.57(2302.68,2324.51) | 556.98(552.83,561.15) | -4.92(-5.22,-4.61) |
| Zambia | both | 310470.90(213525.20,473581.02) | 73279.75(43867.62,108880.13) | -76.40 | 5349.00(5325.19,5372.91) | 702.48(696.38,708.62) | -6.47(-7.13,-5.80) |
| Zimbabwe | both | 108006.09(75318.17,144800.34) | 172564.70(118318.68,238531.12) | 59.77 | 1818.77(1806.29,1831.31) | 1905.67(1894.16,1917.24) | 0.97(0.55,1.39) |
| Global | female | 20144738.01(16347332.54,25329033.17) | 4146609.53(3345822.40,4967168.98) | -79.42 | 2047.05(2046.02,2048.07) | 398.73(398.30,399.17) | -5.20(-5.73,-4.68) |
| Central Europe, eastern Europe, and central Asia | female | 43085.83(35411.65,51681.49) | 6847.79(5410.68,8612.62) | -84.11 | 77.10(76.28,77.92) | 16.22(15.78,16.67) | -5.73(-6.21,-5.24) |
| High-income | female | 39392.09(35975.02,43657.23) | 9049.52(6041.50,14000.46) | -77.03 | 34.35(33.95,34.75) | 9.39(9.18,9.60) | -3.70(-3.99,-3.40) |
| Latin America and Caribbean | female | 1107035.02(1021280.76,1207848.88) | 131225.88(104732.83,165550.07) | -88.15 | 1243.98(1241.28,1246.69) | 152.23(151.26,153.20) | -6.77(-6.99,-6.56) |
| Southeast Asia, east Asia, and Oceania | female | 2184907.80(1861803.87,2684347.56) | 187127.70(156542.46,222373.73) | -91.44 | 793.78(792.59,794.97) | 93.15(92.68,93.63) | -9.24(-10.95,-7.50) |
| Sub-Saharan Africa | female | 7447094.21(5574023.73,10103523.27) | 2656325.60(2003027.14,3308757.78) | -64.33 | 5070.07(5065.76,5074.39) | 923.73(922.44,925.03) | -5.25(-5.88,-4.62) |
| Andean Latin America | female | 187286.24(149183.63,233436.07) | 17547.54(13013.52,23124.77) | -90.63 | 1711.94(1702.11,1721.81) | 147.10(144.44,149.80) | -7.97(-8.30,-7.65) |
| Australasia | female | 34.58(28.67,41.36) | 25.99(16.45,109.86) | -24.84 | 0.72(0.40,1.21) | 0.69(0.41,1.12) | -0.04(-0.53,0.46) |
| Caribbean | female | 139048.33(106355.56,184465.62) | 35860.05(24446.66,52053.34) | -74.21 | 2047.49(2035.32,2059.72) | 590.80(584.10,597.57) | -3.66(-4.02,-3.29) |
| Central Asia | female | 23549.67(19610.03,27950.54) | 3261.56(2540.58,4076.00) | -86.15 | 151.27(149.08,153.49) | 20.45(19.67,21.25) | -7.31(-7.96,-6.66) |
| Central Europe | female | 3441.33(2785.81,4184.16) | 723.98(610.58,863.92) | -78.96 | 24.69(23.79,25.63) | 7.11(6.50,7.75) | -4.72(-5.14,-4.29) |
| Central Latin America | female | 453959.65(417396.80,495698.33) | 59246.43(45565.92,75584.25) | -86.95 | 1070.87(1067.19,1074.55) | 143.81(142.38,145.25) | -6.16(-6.30,-6.01) |
| Central Sub-Saharan Africa | female | 882309.83(574877.66,1351605.92) | 236637.91(146095.29,347189.05) | -73.18 | 5286.95(5274.13,5299.78) | 736.79(733.51,740.08) | -6.22(-6.80,-5.62) |
| East Asia | female | 1261449.91(1034362.57,1519193.59) | 17611.11(13627.42,22233.42) | -98.60 | 675.28(673.94,676.62) | 16.58(16.30,16.86) | -16.44(-19.39,-13.38) |
| Eastern Europe | female | 16094.84(12305.86,20750.50) | 2862.25(2085.87,3890.31) | -82.22 | 62.30(61.23,63.38) | 16.78(16.07,17.51) | -4.50(-4.98,-4.02) |
| Eastern Sub-Saharan Africa | female | 4279458.53(3178073.55,5639514.67) | 1233928.48(938498.63,1555324.18) | -71.17 | 7474.10(7465.83,7482.38) | 1207.22(1204.78,1209.66) | -5.59(-6.51,-4.66) |
| High-income Asia Pacific | female | 1240.61(998.96,1543.26) | 295.94(229.94,380.83) | -76.15 | 7.28(6.84,7.75) | 2.93(2.58,3.32) | -2.59(-2.78,-2.40) |
| High-income North America | female | 1330.78(662.03,4434.34) | 2868.78(1489.64,5764.59) | 115.57 | 4.08(3.85,4.31) | 8.42(8.10,8.76) | 1.44(0.51,2.37) |
| North Africa and Middle East | female | 646633.53(500167.95,880150.86) | 141235.89(108733.32,183339.56) | -78.16 | 796.32(794.15,798.50) | 150.47(149.61,151.34) | -5.44(-5.81,-5.07) |
| Oceania | female | 7623.45(5733.81,10166.24) | 8856.86(6493.79,11892.37) | 16.18 | 322.19(312.34,332.29) | 224.83(218.79,231.01) | -0.80(-1.20,-0.40) |
| South Asia | female | 8676589.52(6678186.52,10804956.45) | 1014797.16(800087.15,1278508.15) | -88.30 | 3584.37(3581.69,3587.05) | 432.94(432.01,433.86) | -6.03(-6.32,-5.74) |
| Southeast Asia | female | 915834.44(723165.62,1229116.19) | 160659.74(132886.20,193269.65) | -82.46 | 1030.29(1027.94,1032.66) | 185.34(184.33,186.36) | -5.39(-5.45,-5.34) |
| Southern Latin America | female | 35477.03(32482.72,38773.75) | 2350.41(1935.52,2823.01) | -93.37 | 363.02(358.55,367.54) | 26.02(24.70,27.39) | -8.27(-8.82,-7.72) |
| Southern Sub-Saharan Africa | female | 259419.89(211883.20,309755.85) | 166380.38(126638.17,210067.93) | -35.86 | 1771.71(1762.99,1780.47) | 1046.55(1040.28,1052.85) | -0.76(-1.18,-0.33) |
| Tropical Latin America | female | 326740.80(289999.93,363597.60) | 18571.85(14975.83,22880.44) | -94.32 | 1170.66(1166.17,1175.16) | 63.66(62.63,64.71) | -9.54(-10.16,-8.91) |
| Western Europe | female | 1309.09(1051.45,1999.93) | 3508.40(1102.57,7291.64) | 168.00 | 3.65(3.45,3.87) | 9.97(9.64,10.32) | 5.10(4.28,5.92) |
| Western Sub-Saharan Africa | female | 2025905.96(1460526.69,2917008.79) | 1019378.83(732236.24,1306559.29) | -49.68 | 3296.07(3290.52,3301.63) | 735.36(733.67,737.06) | -4.67(-4.87,-4.48) |
| Afghanistan | female | 57780.16(35558.39,85337.60) | 35866.73(22498.58,56843.50) | -37.93 | 2042.62(2024.03,2061.35) | 431.70(426.91,436.52) | -4.98(-5.47,-4.48) |
| Albania | female | 1931.84(1460.94,2472.37) | 127.64(78.97,188.97) | -93.39 | 339.67(323.20,356.79) | 47.70(37.75,59.55) | -6.74(-7.22,-6.26) |
| Algeria | female | 20104.48(13964.55,28677.17) | 2229.90(1570.05,3038.21) | -88.91 | 374.80(369.31,380.37) | 32.11(30.68,33.58) | -8.39(-8.84,-7.94) |
| American Samoa | female | 55.44(36.74,80.16) | 14.71(8.82,22.53) | -73.46 | 242.33(156.55,363.74) | 128.54(48.21,283.19) | -2.07(-2.55,-1.59) |
| Andorra | female | 0.03(0.00,0.02) | 0.94(0.00,3.24) | 3492.67 | 0.53(0.00,134.41) | 16.23(0.31,154.60) | 11.60(9.39,13.85) |
| Angola | female | 351329.39(215698.97,560131.44) | 78338.11(47557.14,111927.71) | -77.70 | 11829.76(11784.24,11875.42) | 928.23(921.09,935.42) | -8.31(-8.65,-7.96) |
| Antigua and Barbuda | female | 32.23(26.96,39.63) | 13.08(10.64,15.78) | -59.41 | 265.24(162.94,411.26) | 98.72(39.03,211.71) | -3.33(-4.04,-2.61) |
| Argentina | female | 29457.02(26587.17,32594.79) | 1577.20(1291.43,1948.41) | -94.65 | 455.49(449.33,461.71) | 24.68(23.14,26.30) | -9.19(-9.75,-8.63) |
| Armenia | female | 740.48(496.55,937.09) | 45.75(26.67,73.11) | -93.82 | 135.81(125.72,146.53) | 20.14(14.62,27.12) | -5.95(-6.55,-5.34) |
| Australia | female | 33.33(27.63,40.17) | 22.42(15.87,24.38) | -32.73 | 0.82(0.45,1.41) | 0.69(0.38,1.17) | -0.84(-1.32,-0.37) |
| Austria | female | 2.95(0.50,9.53) | 63.46(0.27,536.79) | 2052.86 | 0.47(0.09,1.52) | 9.86(7.58,12.68) | 19.29(14.63,24.13) |
| Azerbaijan | female | 6080.42(4637.40,7811.44) | 607.48(430.48,806.24) | -90.01 | 433.06(421.23,445.15) | 56.03(51.20,61.22) | -7.33(-7.77,-6.89) |
| Bahamas | female | 115.65(94.49,140.60) | 20.13(15.09,27.48) | -82.59 | 258.66(207.98,319.01) | 46.01(24.70,79.66) | -5.45(-6.08,-4.80) |
| Bahrain | female | 163.32(124.51,209.80) | 32.20(25.00,42.00) | -80.28 | 183.78(154.92,216.81) | 20.00(12.94,29.81) | -6.89(-7.27,-6.51) |
| Bangladesh | female | 2544342.12(2021614.81,3120390.45) | 62417.64(43004.55,88674.40) | -97.55 | 9227.75(9215.20,9240.32) | 280.94(278.48,283.41) | -10.92(-11.25,-10.59) |
| Barbados | female | 66.31(54.72,78.37) | 7.84(5.73,10.50) | -88.18 | 192.51(143.16,254.79) | 32.27(11.65,73.48) | -5.91(-6.30,-5.52) |
| Belarus | female | 395.12(226.02,511.14) | 74.06(54.47,96.50) | -81.26 | 34.43(30.88,38.28) | 10.47(8.05,13.42) | -5.15(-6.50,-3.78) |
| Belgium | female | 27.58(23.13,34.63) | 85.03(12.23,406.05) | 208.29 | 2.96(1.91,4.45) | 8.58(6.82,10.72) | 6.02(4.65,7.40) |
| Belize | female | 609.45(520.90,704.92) | 109.73(87.94,139.11) | -82.00 | 1170.73(1065.17,1284.62) | 129.00(99.89,164.60) | -6.68(-7.47,-5.88) |
| Benin | female | 70598.47(42070.11,112229.52) | 20023.48(11673.53,31452.04) | -71.64 | 4558.94(4519.62,4598.54) | 527.20(518.86,535.65) | -6.87(-7.21,-6.53) |
| Bermuda | female | 3.82(2.71,4.77) | 0.53(0.38,0.69) | -86.15 | 37.53(4.73,143.29) | 7.86(0.00,148.52) | -5.44(-6.24,-4.63) |
| Bhutan | female | 1704.40(773.51,3061.04) | 70.07(30.85,127.44) | -95.89 | 1067.59(1009.28,1128.56) | 70.27(53.04,91.68) | -8.94(-9.18,-8.69) |
| Bolivia (Plurinational State of) | female | 70502.17(45198.33,103802.62) | 7348.28(4989.14,10297.18) | -89.58 | 3163.60(3133.06,3194.38) | 308.71(299.95,317.67) | -7.49(-7.63,-7.34) |
| Bosnia and Herzegovina | female | 67.31(41.98,99.65) | 14.81(9.14,21.88) | -78.00 | 14.41(11.15,18.39) | 7.37(4.03,12.50) | -3.87(-4.46,-3.29) |
| Botswana | female | 4568.08(2902.46,6717.91) | 3624.03(2105.90,5957.23) | -20.67 | 1107.85(1066.95,1149.96) | 635.21(606.03,665.49) | -0.86(-1.30,-0.41) |
| Brazil | female | 321151.28(284323.56,358460.56) | 17101.26(13874.74,21067.09) | -94.68 | 1200.95(1196.31,1205.60) | 59.55(58.53,60.58) | -9.80(-10.45,-9.15) |
| Brunei Darussalam | female | 6.25(3.51,11.91) | 1.64(0.74,3.07) | -73.72 | 5.62(0.82,19.81) | 1.63(0.01,14.22) | -3.12(-3.59,-2.66) |
| Bulgaria | female | 114.40(72.17,167.77) | 61.78(44.70,83.81) | -46.00 | 8.71(6.65,11.24) | 9.34(6.60,12.93) | -0.20(-0.53,0.14) |
| Burkina Faso | female | 175643.27(107019.60,266211.60) | 109993.29(66503.91,180364.03) | -37.38 | 5399.21(5368.22,5430.34) | 1551.69(1540.82,1562.61) | -3.79(-4.16,-3.43) |
| Burundi | female | 175495.10(95359.15,331211.51) | 37554.30(18923.84,68065.63) | -78.60 | 10965.55(10906.97,11024.38) | 1116.95(1104.37,1129.65) | -7.05(-7.39,-6.71) |
| Cabo Verde | female | 2633.97(1711.22,3840.55) | 68.76(42.45,111.25) | -97.39 | 2697.16(2583.29,2815.08) | 80.08(58.58,107.34) | -11.65(-12.14,-11.16) |
| Cambodia | female | 112537.83(66588.09,191814.04) | 5042.34(3540.88,6999.83) | -95.52 | 3975.75(3949.95,4001.69) | 194.29(188.49,200.24) | -10.71(-11.30,-10.12) |
| Cameroon | female | 42187.22(26450.95,62867.19) | 22775.20(13830.14,35538.69) | -46.01 | 1231.20(1216.93,1245.61) | 259.41(255.38,263.49) | -5.12(-5.49,-4.76) |
| Canada | female | 26.39(19.75,25.40) | 196.56(46.51,1140.35) | 644.75 | 0.84(0.53,1.28) | 6.16(5.31,7.13) | 6.05(4.67,7.46) |
| Central African Republic | female | 28434.43(15998.81,51471.64) | 28499.64(16824.74,44434.97) | 0.23 | 3532.28(3485.79,3579.27) | 2108.57(2080.74,2136.69) | -1.12(-1.47,-0.76) |
| Chad | female | 106669.91(61710.06,184884.91) | 63240.39(41405.46,96597.95) | -40.71 | 5511.25(5472.30,5550.42) | 1082.29(1072.53,1092.13) | -5.35(-5.54,-5.16) |
| Chile | female | 4800.28(4326.90,5326.51) | 676.23(517.98,883.23) | -85.91 | 169.03(163.32,174.90) | 29.61(26.91,32.53) | -5.30(-5.99,-4.60) |
| China | female | 1239438.07(1014992.72,1488256.22) | 16606.94(12711.45,20940.05) | -98.66 | 688.01(686.63,689.39) | 16.33(16.05,16.61) | -13.78(-14.64,-12.90) |
| Colombia | female | 44863.71(37316.53,52913.74) | 12589.84(8787.58,17657.24) | -71.94 | 589.98(583.65,596.37) | 174.59(170.79,178.46) | -2.93(-3.60,-2.25) |
| Comoros | female | 6349.05(4045.23,9444.91) | 1207.49(787.68,1817.19) | -80.98 | 4974.22(4837.39,5114.16) | 1028.12(965.74,1093.64) | -5.16(-5.35,-4.97) |
| Congo | female | 8034.30(5037.69,12113.89) | 3361.87(2149.65,4912.43) | -58.16 | 1351.30(1318.94,1384.29) | 352.51(339.64,365.77) | -4.64(-5.30,-3.98) |
| Cook Islands | female | 0.97(0.53,1.61) | 0.11(0.06,0.16) | -88.48 | 12.56(0.00,180.98) | 4.21(0.00,318.68) | -7.27(-8.61,-5.91) |
| Costa Rica | female | 304.08(260.12,353.62) | 29.97(23.92,38.26) | -90.15 | 44.23(38.77,50.30) | 4.90(2.96,7.69) | -7.41(-7.92,-6.91) |
| Croatia | female | 10.33(4.00,20.04) | 6.21(3.79,9.72) | -39.92 | 2.58(1.23,4.84) | 2.13(0.71,5.12) | -1.50(-2.22,-0.78) |
| Cuba | female | 486.83(431.13,553.32) | 89.76(70.47,113.15) | -81.56 | 32.04(28.83,35.54) | 8.19(6.21,10.63) | -4.31(-5.34,-3.28) |
| Cyprus | female | 1.98(0.84,3.51) | 0.17(0.10,0.27) | -91.55 | 2.11(0.24,8.90) | 0.15(0.00,4.97) | -7.42(-8.31,-6.52) |
| Czechia | female | 126.70(107.26,159.10) | 108.06(89.62,129.01) | -14.71 | 10.97(8.89,13.43) | 10.70(8.49,13.35) | -0.31(-0.66,0.03) |
| C?te d'Ivoire | female | 65686.24(42432.27,99332.78) | 20126.19(12458.46,31814.17) | -69.36 | 1672.69(1657.67,1687.82) | 274.37(270.00,278.79) | -5.80(-6.14,-5.45) |
| Democratic People's Republic of Korea | female | 20108.34(13167.44,30296.21) | 857.86(455.67,1469.26) | -95.73 | 533.41(525.10,541.82) | 31.43(28.98,34.03) | -18.15(-24.71,-11.01) |
| Democratic Republic of the Congo | female | 489488.65(287924.49,763317.71) | 125141.24(62506.27,220748.10) | -74.43 | 4005.51(3992.47,4018.57) | 603.79(600.10,607.50) | -5.78(-6.59,-4.95) |
| Denmark | female | 5.84(3.93,10.20) | 8.43(3.18,94.16) | 44.25 | 1.28(0.44,3.12) | 1.73(0.75,3.58) | 1.51(0.05,2.98) |
| Djibouti | female | 4089.55(2511.31,6582.66) | 2343.06(1419.26,3726.71) | -42.71 | 4166.50(4020.88,4316.30) | 1080.93(1032.19,1131.48) | -4.44(-5.04,-3.85) |
| Dominica | female | 28.13(19.07,39.83) | 8.83(5.35,14.19) | -68.62 | 184.49(113.83,286.45) | 141.47(54.01,308.03) | -0.44(-1.32,0.44) |
| Dominican Republic | female | 42003.82(32422.38,53118.22) | 3980.44(2297.01,6125.40) | -90.52 | 2424.59(2398.06,2451.36) | 244.45(236.24,252.90) | -6.66(-7.26,-6.05) |
| Ecuador | female | 26155.08(23314.25,29265.90) | 2771.91(1994.38,3797.78) | -89.40 | 960.93(946.71,975.32) | 88.86(84.90,92.97) | -7.87(-8.78,-6.95) |
| Egypt | female | 42834.48(30857.24,58187.78) | 9855.95(7054.12,13075.63) | -76.99 | 305.21(301.94,308.51) | 46.15(45.13,47.18) | -5.00(-5.47,-4.53) |
| El Salvador | female | 10848.14(8362.69,13535.89) | 831.32(563.72,1153.84) | -92.34 | 755.06(738.48,771.95) | 87.05(80.67,93.83) | -6.97(-7.17,-6.77) |
| Equatorial Guinea | female | 3188.77(1855.52,5202.13) | 761.07(363.64,1353.38) | -76.13 | 2514.46(2414.41,2617.79) | 286.12(264.94,308.65) | -8.20(-8.73,-7.67) |
| Eritrea | female | 115826.14(76535.16,170603.47) | 22244.82(14508.37,32590.89) | -80.79 | 12740.35(12655.66,12825.48) | 1508.14(1484.99,1531.57) | -6.61(-6.98,-6.24) |
| Estonia | female | 138.19(118.76,162.71) | 8.85(7.43,10.66) | -93.60 | 74.15(61.02,89.37) | 7.53(2.96,16.15) | -7.93(-9.41,-6.42) |
| Eswatini | female | 3407.40(2163.11,5001.64) | 1587.58(997.77,2433.51) | -53.41 | 1294.26(1241.09,1349.19) | 637.37(601.07,675.42) | -1.57(-1.97,-1.17) |
| Ethiopia | female | 1329514.97(922809.63,1849089.93) | 285644.97(196609.24,377255.72) | -78.52 | 9202.88(9184.90,9220.89) | 1172.62(1167.75,1177.51) | -7.18(-7.63,-6.71) |
| Fiji | female | 395.45(274.72,558.69) | 210.34(131.19,327.41) | -46.81 | 205.06(179.94,232.86) | 71.97(57.45,89.28) | -3.55(-3.95,-3.16) |
| Finland | female | 2.58(0.84,6.85) | 3.04(0.41,0.62) | 17.60 | 0.57(0.09,2.02) | 0.68(0.14,2.40) | 5.16(2.64,7.73) |
| France | female | 797.95(703.73,904.17) | 641.49(267.24,2801.74) | -19.61 | 13.22(12.25,14.26) | 10.51(9.66,11.43) | 0.09(-0.72,0.90) |
| Gabon | female | 1834.29(1163.35,2987.83) | 535.97(268.38,898.87) | -70.78 | 733.28(696.02,772.15) | 160.22(145.61,175.98) | -3.97(-4.59,-3.34) |
| Gambia | female | 9920.92(6246.53,14653.05) | 3127.72(1861.25,4982.18) | -68.47 | 3567.64(3485.79,3650.97) | 525.61(503.80,548.15) | -6.50(-6.86,-6.14) |
| Georgia | female | 116.77(74.87,166.24) | 6.29(3.99,7.96) | -94.61 | 18.48(15.14,22.36) | 1.60(0.51,3.89) | -8.30(-9.35,-7.24) |
| Germany | female | 37.20(18.02,63.14) | 1136.50(18.08,2349.96) | 2955.44 | 0.56(0.39,0.80) | 19.23(18.13,20.39) | 10.30(7.77,12.88) |
| Ghana | female | 166891.67(100549.89,268719.85) | 38505.10(23368.16,57931.08) | -76.93 | 3866.54(3844.40,3888.77) | 526.29(520.31,532.34) | -5.68(-5.95,-5.42) |
| Greece | female | 0.28(0.20,0.40) | 27.25(0.19,529.38) | 9713.52 | 0.04(0.00,0.68) | 3.76(2.48,5.60) | 21.46(17.01,26.07) |
| Greenland | female | 0.39(0.26,0.56) | 0.09(0.06,0.13) | -76.54 | 4.71(0.00,72.99) | 1.61(0.00,89.30) | -2.64(-2.97,-2.32) |
| Grenada | female | 59.37(47.48,73.94) | 9.90(7.65,12.56) | -83.32 | 282.23(204.98,380.82) | 65.14(24.02,147.11) | -4.42(-5.62,-3.21) |
| Guam | female | 38.52(28.48,52.99) | 18.84(12.48,27.43) | -51.09 | 80.30(47.38,130.02) | 45.18(18.71,94.21) | -0.51(-1.04,0.02) |
| Guatemala | female | 74499.47(66000.42,84082.70) | 11459.10(8935.53,14813.82) | -84.62 | 2697.36(2674.25,2720.63) | 351.07(342.99,359.31) | -5.98(-6.35,-5.60) |
| Guinea | female | 124229.33(79609.81,181194.22) | 45748.15(28265.51,69359.33) | -63.17 | 6661.21(6616.95,6705.72) | 1204.29(1191.32,1217.38) | -5.19(-5.52,-4.85) |
| Guinea-Bissau | female | 13483.31(7053.65,24369.00) | 1598.57(864.90,2896.24) | -88.14 | 4584.12(4493.19,4676.46) | 323.23(306.03,341.20) | -8.45(-8.77,-8.13) |
| Guyana | female | 3920.23(3071.81,4832.23) | 486.21(362.80,634.01) | -87.60 | 2381.34(2302.05,2462.86) | 490.21(446.10,537.70) | -3.92(-4.50,-3.35) |
| Haiti | female | 81705.67(55513.68,121873.35) | 29428.43(18816.79,44511.07) | -63.98 | 4648.61(4612.55,4684.89) | 1167.33(1152.67,1182.14) | -4.26(-4.55,-3.98) |
| Honduras | female | 8434.35(6258.20,11115.30) | 854.85(436.61,1381.10) | -89.86 | 612.91(597.79,628.34) | 47.52(43.93,51.33) | -8.23(-8.38,-8.07) |
| Hungary | female | 52.29(38.61,73.64) | 43.98(35.52,52.97) | -15.90 | 5.38(3.93,7.24) | 4.88(3.28,7.04) | 0.11(-0.86,1.09) |
| Iceland | female | 0.30(0.21,0.40) | 1.41(0.23,12.87) | 376.78 | 0.81(0.00,18.63) | 4.00(0.23,22.40) | 7.39(6.10,8.70) |
| India | female | 5405148.51(3896716.08,6997427.46) | 731291.40(562867.30,944903.16) | -86.47 | 2990.37(2987.53,2993.21) | 457.89(456.75,459.03) | -5.18(-5.59,-4.76) |
| Indonesia | female | 499349.19(390781.83,640631.15) | 98311.74(78439.56,119748.53) | -80.31 | 1467.25(1462.72,1471.80) | 282.75(280.76,284.75) | -5.02(-5.10,-4.95) |
| Iran (Islamic Republic of) | female | 55789.32(45300.78,71380.07) | 2003.37(1378.92,2833.09) | -96.41 | 442.49(438.47,446.53) | 21.08(20.07,22.13) | -8.72(-9.10,-8.33) |
| Iraq | female | 20004.91(14309.38,27925.89) | 3333.07(2357.38,4667.96) | -83.34 | 435.21(428.78,441.72) | 49.32(47.47,51.23) | -6.87(-7.43,-6.31) |
| Ireland | female | 2.63(1.08,6.36) | 67.55(0.46,284.33) | 2472.13 | 0.67(0.11,2.27) | 12.91(10.01,16.54) | 16.58(14.05,19.18) |
| Israel | female | 12.03(8.92,18.54) | 109.31(5.41,521.97) | 808.71 | 1.54(0.76,2.84) | 8.48(6.96,10.27) | 9.35(7.87,10.86) |
| Italy | female | 34.20(12.51,71.79) | 495.80(29.95,1446.32) | 1349.91 | 0.83(0.57,1.19) | 12.32(11.25,13.49) | 11.71(9.37,14.11) |
| Jamaica | female | 3221.44(2709.68,3854.28) | 162.20(123.26,213.71) | -94.96 | 575.75(551.43,600.94) | 47.64(39.24,57.45) | -6.96(-8.05,-5.87) |
| Japan | female | 637.90(473.86,866.53) | 262.42(201.45,344.48) | -58.86 | 5.94(5.44,6.48) | 3.72(3.25,4.24) | -1.30(-1.53,-1.08) |
| Jordan | female | 669.57(523.96,826.23) | 222.50(167.55,294.72) | -66.77 | 69.92(64.30,75.95) | 12.78(11.01,14.78) | -5.43(-5.68,-5.18) |
| Kazakhstan | female | 2156.82(1618.55,2648.85) | 695.71(547.63,873.75) | -67.74 | 69.59(66.34,72.97) | 20.51(18.82,22.31) | -4.14(-4.48,-3.81) |
| Kenya | female | 231225.03(162366.89,303760.47) | 84844.33(64900.34,111249.75) | -63.31 | 3065.21(3050.28,3080.21) | 790.26(783.87,796.70) | -3.69(-3.98,-3.39) |
| Kiribati | female | 926.06(583.69,1357.09) | 319.72(193.63,490.80) | -65.48 | 2493.48(2246.28,2762.43) | 733.32(614.30,870.14) | -4.01(-4.25,-3.76) |
| Kuwait | female | 72.59(51.78,101.60) | 6.67(5.24,8.24) | -90.81 | 24.89(18.99,32.08) | 1.41(0.45,3.43) | -9.31(-10.20,-8.41) |
| Kyrgyzstan | female | 589.86(462.42,734.04) | 70.24(55.91,87.39) | -88.09 | 56.59(51.57,62.01) | 5.65(4.29,7.34) | -7.64(-8.39,-6.89) |
| Lao People's Democratic Republic | female | 39711.30(21941.63,73451.30) | 3445.04(1986.18,5568.60) | -91.32 | 3793.28(3752.93,3833.99) | 291.64(281.53,302.05) | -8.31(-8.56,-8.07) |
| Latvia | female | 161.23(130.14,201.97) | 13.25(11.20,15.63) | -91.78 | 54.91(46.13,64.93) | 9.01(4.46,16.46) | -7.14(-8.53,-5.72) |
| Lebanon | female | 394.58(257.69,570.82) | 56.42(25.12,117.02) | -85.70 | 68.83(61.68,76.63) | 9.77(7.25,12.94) | -5.29(-6.32,-4.25) |
| Lesotho | female | 6418.31(4268.78,9005.27) | 3111.58(1920.10,5030.49) | -51.52 | 1508.76(1465.45,1553.09) | 830.24(796.28,865.35) | -1.65(-1.96,-1.34) |
| Liberia | female | 64191.32(41218.32,97405.76) | 8026.77(4588.59,12616.73) | -87.50 | 7212.73(7142.33,7283.70) | 583.42(568.29,598.86) | -8.62(-9.40,-7.83) |
| Libya | female | 1074.82(708.18,1554.93) | 314.59(210.18,445.08) | -70.73 | 112.31(105.16,119.84) | 47.27(41.56,53.57) | -1.40(-1.90,-0.90) |
| Lithuania | female | 206.25(170.97,259.42) | 16.22(13.75,19.02) | -92.13 | 48.56(41.74,56.24) | 8.21(4.48,13.98) | -6.46(-7.88,-5.02) |
| Luxembourg | female | 1.32(1.09,1.71) | 0.77(0.49,0.75) | -41.70 | 3.51(0.13,21.37) | 1.34(0.00,12.80) | -2.77(-3.83,-1.70) |
| Madagascar | female | 398278.89(307703.33,488643.38) | 122993.06(75422.08,186410.66) | -69.12 | 11474.65(11432.51,11516.91) | 1972.71(1960.40,1985.08) | -5.03(-5.30,-4.77) |
| Malawi | female | 225429.32(146742.15,348751.97) | 43071.11(26375.49,62588.92) | -80.89 | 6734.40(6700.55,6768.38) | 917.31(907.16,927.55) | -6.62(-7.05,-6.20) |
| Malaysia | female | 8398.07(5895.04,11626.29) | 3493.42(2391.60,4882.98) | -58.40 | 243.97(238.23,249.83) | 95.57(92.05,99.20) | -3.14(-3.31,-2.97) |
| Maldives | female | 1685.39(1004.78,2864.89) | 54.58(38.09,79.76) | -96.76 | 2731.42(2588.64,2880.34) | 113.97(83.00,153.17) | -9.70(-10.41,-8.98) |
| Mali | female | 270052.98(168183.86,421826.30) | 235625.60(144211.02,350087.88) | -12.75 | 10185.46(10139.52,10231.56) | 3013.25(2998.80,3027.77) | -3.89(-4.29,-3.48) |
| Malta | female | 0.41(0.12,0.89) | 0.36(0.04,0.07) | -11.77 | 1.07(0.00,14.17) | 1.16(0.00,17.94) | 0.57(-2.52,3.75) |
| Marshall Islands | female | 41.38(21.89,65.88) | 17.69(9.86,29.42) | -57.25 | 206.65(126.23,322.79) | 95.50(38.95,201.64) | -2.74(-3.46,-2.01) |
| Mauritania | female | 16824.60(9795.95,30125.63) | 2365.71(1436.10,4443.54) | -85.94 | 2736.31(2686.54,2786.80) | 218.85(208.74,229.35) | -7.84(-8.00,-7.68) |
| Mauritius | female | 531.81(422.08,665.87) | 105.46(76.24,143.99) | -80.17 | 318.39(288.45,350.75) | 103.04(81.84,128.28) | -3.36(-3.65,-3.07) |
| Mexico | female | 271597.49(245240.05,303891.34) | 22943.09(17992.90,29257.24) | -91.55 | 1243.27(1237.77,1248.79) | 118.84(117.00,120.71) | -7.29(-7.47,-7.11) |
| Micronesia (Federated States of) | female | 156.01(83.98,248.20) | 19.50(11.66,32.13) | -87.50 | 369.17(289.72,465.06) | 70.56(32.17,137.93) | -5.23(-5.41,-5.06) |
| Monaco | female | 0.11(0.00,0.72) | 0.39(0.00,1.32) | 256.45 | 6.36(0.00,329.48) | 15.31(0.00,232.96) | 2.95(2.05,3.86) |
| Mongolia | female | 226.93(145.53,349.85) | 16.95(9.07,28.39) | -92.53 | 42.42(36.49,49.08) | 2.68(1.44,4.63) | -10.09(-10.76,-9.42) |
| Montenegro | female | 3.48(2.31,5.08) | 0.48(0.26,0.86) | -86.27 | 4.28(0.84,13.44) | 0.71(0.00,10.93) | -5.60(-8.93,-2.15) |
| Morocco | female | 22777.51(14333.42,35521.63) | 1534.58(941.95,2438.51) | -93.26 | 385.21(379.66,390.83) | 27.57(26.00,29.22) | -8.15(-8.81,-7.48) |
| Mozambique | female | 326719.94(191361.33,499626.21) | 75004.10(42649.75,114640.02) | -77.04 | 8442.13(8407.67,8476.71) | 849.51(842.44,856.63) | -7.32(-7.52,-7.12) |
| Myanmar | female | 53959.81(34894.62,92583.10) | 4138.34(2801.76,5964.13) | -92.33 | 667.39(660.99,673.85) | 48.47(46.83,50.16) | -8.62(-9.08,-8.16) |
| Namibia | female | 7457.14(4937.49,10514.59) | 3409.14(1984.26,5455.19) | -54.28 | 1835.34(1784.00,1887.83) | 595.70(570.16,622.13) | -2.76(-3.24,-2.28) |
| Nauru | female | 15.19(9.19,24.18) | 7.33(4.22,12.05) | -51.75 | 243.39(76.71,596.80) | 139.45(21.41,487.18) | -1.92(-3.04,-0.78) |
| Nepal | female | 327930.87(217162.66,484771.59) | 15935.09(10248.28,22952.42) | -95.14 | 5900.43(5876.70,5924.24) | 348.73(342.97,354.56) | -8.81(-8.99,-8.62) |
| Netherlands | female | 159.22(19.21,802.95) | 311.46(11.06,678.50) | 95.61 | 11.75(9.98,13.77) | 22.74(20.27,25.47) | 3.31(2.62,4.01) |
| New Zealand | female | 1.24(0.86,1.21) | 3.56(0.52,48.72) | 186.49 | 0.28(0.01,1.76) | 0.71(0.17,2.17) | 5.61(4.14,7.09) |
| Nicaragua | female | 10838.36(8505.18,13936.15) | 1512.56(980.19,2150.57) | -86.04 | 996.36(975.28,1017.79) | 118.82(111.66,126.34) | -7.34(-7.88,-6.78) |
| Niger | female | 269595.98(153885.32,425878.00) | 70701.04(45293.39,101062.60) | -73.78 | 9210.07(9166.60,9253.70) | 804.87(797.77,812.03) | -8.45(-8.97,-7.92) |
| Nigeria | female | 428669.44(293341.85,686426.02) | 218414.06(154627.21,292133.74) | -49.05 | 1401.26(1395.91,1406.63) | 347.04(345.35,348.74) | -4.23(-4.64,-3.82) |
| Niue | female | 0.85(0.52,1.23) | 1.18(0.82,1.71) | 39.34 | 157.54(0.16,1627.37) | 471.84(2.80,3770.71) | -0.49(-1.94,0.98) |
| North Macedonia | female | 125.75(92.33,166.30) | 13.20(7.90,20.31) | -89.50 | 44.60(36.30,54.36) | 6.90(3.15,13.38) | -5.48(-5.92,-5.05) |
| Northern Mariana Islands | female | 8.47(5.19,12.86) | 3.32(2.25,4.94) | -60.80 | 61.83(17.26,170.91) | 35.35(2.64,164.97) | -1.21(-1.59,-0.82) |
| Norway | female | 2.11(0.94,5.37) | 43.80(2.36,153.69) | 1971.47 | 0.48(0.05,2.08) | 9.03(6.53,12.34) | 12.23(10.74,13.74) |
| Oman | female | 2037.13(1244.58,3017.66) | 301.60(192.39,459.30) | -85.20 | 398.03(378.89,417.94) | 47.40(41.67,53.72) | -5.65(-6.33,-4.96) |
| Pakistan | female | 397463.62(287920.52,520761.69) | 205082.96(141999.52,282007.83) | -48.40 | 1203.48(1199.06,1207.92) | 422.97(420.89,425.05) | -2.34(-2.74,-1.94) |
| Palau | female | 5.38(3.22,8.89) | 1.25(0.78,1.98) | -76.70 | 127.53(21.43,441.94) | 45.00(0.07,446.65) | -2.83(-3.12,-2.54) |
| Palestine | female | 671.23(450.94,948.10) | 119.02(82.38,169.89) | -82.27 | 116.82(107.44,126.86) | 11.23(9.04,13.83) | -7.31(-7.75,-6.87) |
| Panama | female | 2666.61(2237.35,3151.63) | 1454.81(1057.66,1869.59) | -45.44 | 453.69(432.19,476.05) | 128.35(118.37,138.98) | -5.32(-6.14,-4.48) |
| Papua New Guinea | female | 4276.96(3017.71,6185.91) | 7174.88(5022.46,10018.53) | 67.76 | 302.95(291.02,315.29) | 242.38(235.28,249.64) | -0.33(-0.83,0.17) |
| Paraguay | female | 5589.51(4369.15,7130.71) | 1470.60(991.60,2055.48) | -73.69 | 408.87(395.05,423.09) | 169.89(161.15,179.00) | -3.00(-3.69,-2.31) |
| Peru | female | 90629.00(65018.25,121781.56) | 7427.35(4519.57,10923.17) | -91.80 | 1558.92(1546.24,1571.68) | 117.39(114.17,120.68) | -8.48(-8.99,-7.98) |
| Philippines | female | 132323.80(108982.82,162726.16) | 34400.60(28645.43,41555.55) | -74.00 | 930.22(924.47,936.00) | 208.40(205.98,210.84) | -4.14(-4.41,-3.88) |
| Poland | female | 230.84(147.54,364.91) | 78.25(60.02,111.80) | -66.10 | 5.53(4.80,6.35) | 2.54(1.95,3.27) | -3.06(-3.51,-2.61) |
| Portugal | female | 39.50(29.74,53.79) | 5.51(4.29,5.92) | -86.04 | 4.52(3.16,6.31) | 0.79(0.24,2.01) | -5.61(-6.95,-4.25) |
| Puerto Rico | female | 394.99(349.26,447.47) | 46.00(38.19,55.67) | -88.35 | 76.20(67.92,85.27) | 25.46(17.57,35.81) | -3.67(-4.73,-2.61) |
| Qatar | female | 23.94(16.55,34.37) | 19.01(13.20,27.69) | -20.58 | 28.16(17.13,44.46) | 5.68(3.11,9.73) | -4.25(-4.79,-3.70) |
| Republic of Korea | female | 525.42(391.17,691.76) | 29.40(20.36,36.19) | -94.40 | 9.22(8.37,10.15) | 0.91(0.57,1.41) | -7.05(-7.43,-6.67) |
| Republic of Moldova | female | 362.81(287.54,449.00) | 32.93(24.54,43.96) | -90.92 | 56.77(50.55,63.56) | 14.27(9.49,20.73) | -5.77(-7.56,-3.94) |
| Romania | female | 549.12(463.07,674.74) | 210.59(166.71,263.57) | -61.65 | 20.53(18.66,22.54) | 12.06(10.21,14.17) | -1.80(-2.49,-1.10) |
| Russian Federation | female | 6081.85(4128.51,8513.10) | 846.08(658.18,1110.90) | -86.09 | 36.01(35.02,37.02) | 6.39(5.90,6.92) | -6.45(-7.11,-5.78) |
| Rwanda | female | 133012.45(81015.32,195900.28) | 19331.52(12649.01,27582.21) | -85.47 | 6041.14(6003.27,6079.19) | 667.77(656.85,678.82) | -8.57(-9.36,-7.77) |
| Saint Kitts and Nevis | female | 94.67(81.11,109.42) | 19.20(15.11,24.78) | -79.72 | 1053.32(810.16,1350.71) | 275.07(138.40,498.71) | -4.02(-5.02,-3.01) |
| Saint Lucia | female | 122.36(99.90,146.30) | 16.08(11.66,22.23) | -86.86 | 420.05(339.20,515.32) | 90.59(44.73,166.74) | -4.71(-5.42,-3.99) |
| Saint Vincent and the Grenadines | female | 199.88(161.82,244.82) | 35.09(27.03,45.01) | -82.44 | 909.96(772.02,1067.04) | 261.81(171.47,386.44) | -4.03(-5.10,-2.95) |
| Samoa | female | 136.28(83.09,210.72) | 56.74(34.35,91.73) | -58.37 | 200.31(154.68,256.13) | 69.72(45.54,103.12) | -3.24(-3.30,-3.18) |
| San Marino | female | 0.03(0.01,0.02) | 0.13(0.00,1.00) | 408.34 | 1.15(0.00,294.68) | 5.59(0.00,294.59) | 4.55(2.83,6.29) |
| Sao Tome and Principe | female | 1558.00(1042.15,2110.72) | 116.27(70.29,177.81) | -92.54 | 4475.96(4215.78,4748.71) | 302.08(245.19,369.00) | -8.52(-9.05,-8.00) |
| Saudi Arabia | female | 16655.67(10726.75,26090.28) | 1189.95(694.07,1904.58) | -92.86 | 449.99(442.49,457.60) | 34.86(32.79,37.04) | -7.71(-7.94,-7.47) |
| Senegal | female | 61961.93(40446.36,92880.10) | 9387.02(5726.02,14902.73) | -84.85 | 2627.44(2602.82,2652.23) | 256.53(250.55,262.62) | -7.29(-7.78,-6.80) |
| Serbia | female | 119.57(78.75,192.90) | 25.25(15.00,41.51) | -78.88 | 9.46(7.62,11.65) | 4.46(2.75,6.89) | -1.25(-2.27,-0.23) |
| Seychelles | female | 8.91(6.52,11.63) | 6.98(5.22,9.33) | -21.63 | 70.27(29.10,146.29) | 55.32(19.68,127.15) | -0.13(-0.40,0.13) |
| Sierra Leone | female | 113440.95(70000.36,167931.33) | 145566.28(91835.80,216885.65) | 28.32 | 8138.85(8080.74,8197.32) | 5351.56(5316.82,5386.48) | -0.08(-0.75,0.60) |
| Singapore | female | 71.04(32.97,121.12) | 2.48(1.23,7.82) | -96.51 | 21.89(16.39,28.78) | 0.48(0.05,2.01) | -12.03(-13.01,-11.05) |
| Slovakia | female | 50.40(37.86,66.45) | 18.72(12.84,25.97) | -62.85 | 7.72(5.56,10.50) | 3.83(2.13,6.47) | -1.59(-2.23,-0.95) |
| Slovenia | female | 4.27(2.49,7.02) | 4.46(3.35,6.01) | 4.55 | 2.34(0.58,6.46) | 2.71(0.67,7.71) | -0.24(-0.55,0.07) |
| Solomon Islands | female | 685.25(403.56,1086.74) | 400.29(262.32,593.54) | -41.58 | 441.03(395.64,490.67) | 185.64(161.52,212.54) | -2.46(-2.68,-2.24) |
| Somalia | female | 234834.98(138195.14,379318.95) | 148619.70(87458.32,231905.76) | -36.71 | 10970.66(10920.62,11020.87) | 2547.90(2533.84,2562.02) | -3.27(-6.00,-0.46) |
| South Africa | female | 189338.48(151990.28,229785.10) | 67235.18(51368.72,82678.38) | -64.49 | 1881.54(1870.34,1892.80) | 757.37(750.68,764.11) | -1.80(-2.27,-1.32) |
| South Sudan | female | 201985.32(129850.04,317272.60) | 117189.95(74723.45,170704.70) | -41.98 | 12860.63(12794.73,12926.81) | 4900.19(4868.09,4932.47) | -3.19(-3.92,-2.46) |
| Spain | female | 19.43(16.42,22.69) | 11.19(9.13,13.51) | -42.38 | 0.60(0.34,0.97) | 0.39(0.18,0.74) | -1.38(-1.83,-0.93) |
| Sri Lanka | female | 9325.39(7088.92,11579.80) | 3219.15(2098.20,4579.34) | -65.48 | 341.59(333.80,349.52) | 141.73(136.32,147.32) | -2.90(-3.14,-2.67) |
| Sudan | female | 200148.08(108835.81,354740.20) | 29670.78(18990.69,49421.74) | -85.18 | 3530.76(3512.55,3549.04) | 328.27(324.04,332.54) | -7.24(-7.63,-6.84) |
| Suriname | female | 581.75(414.27,766.89) | 110.91(72.85,168.48) | -80.93 | 837.73(765.83,915.04) | 143.98(115.16,178.35) | -5.95(-6.37,-5.53) |
| Sweden | female | 7.10(5.51,11.36) | 74.20(4.64,389.88) | 945.39 | 0.86(0.34,1.89) | 8.05(6.32,10.18) | 11.32(9.64,13.03) |
| Switzerland | female | 11.35(9.73,13.48) | 52.29(4.88,261.43) | 360.80 | 1.83(0.88,3.47) | 7.79(5.80,10.32) | 7.80(6.57,9.04) |
| Syrian Arab Republic | female | 17822.25(13213.25,23462.42) | 2398.47(1704.95,3232.99) | -86.54 | 571.45(562.49,580.54) | 139.30(132.78,146.07) | -3.68(-4.40,-2.95) |
| Taiwan (Province of China) | female | 1903.50(1700.52,2175.59) | 146.31(122.42,171.95) | -92.31 | 69.22(65.76,72.84) | 10.85(9.00,12.98) | -6.51(-8.22,-4.77) |
| Tajikistan | female | 4418.06(3335.74,5825.95) | 1198.90(789.66,1715.12) | -72.86 | 283.61(273.99,293.51) | 56.23(52.67,59.97) | -6.45(-7.69,-5.21) |
| Thailand | female | 20270.33(14295.05,27763.27) | 3102.43(2349.10,4098.59) | -84.69 | 230.47(226.92,234.07) | 68.74(66.04,71.52) | -3.39(-3.91,-2.87) |
| Timor-Leste | female | 13871.34(7532.80,28596.15) | 2059.89(1385.03,2992.06) | -85.15 | 7272.41(7141.35,7405.50) | 763.03(727.35,800.10) | -7.86(-8.33,-7.39) |
| Togo | female | 21599.58(13573.44,33397.30) | 3957.33(2441.80,6185.72) | -81.68 | 2065.82(2034.14,2097.88) | 207.05(199.69,214.62) | -7.54(-7.85,-7.23) |
| Tokelau | female | 2.31(1.18,3.80) | 1.21(0.75,1.81) | -47.59 | 240.24(0.37,2285.17) | 534.36(2.26,4385.36) | -3.05(-5.31,-0.74) |
| Tonga | female | 112.11(66.73,171.14) | 50.54(32.04,76.89) | -54.91 | 235.76(172.85,315.93) | 114.99(70.00,179.54) | -2.14(-2.56,-1.72) |
| Trinidad and Tobago | female | 664.17(550.20,794.95) | 99.66(76.72,129.67) | -84.99 | 311.58(285.59,339.38) | 74.39(58.39,93.57) | -5.05(-5.27,-4.83) |
| Tunisia | female | 2851.38(1828.80,4157.63) | 109.10(66.55,158.19) | -96.17 | 183.99(176.84,191.38) | 7.60(6.09,9.39) | -10.10(-10.62,-9.58) |
| Türkiye | female | 54239.42(33884.78,84665.81) | 3184.98(2094.81,5266.43) | -94.13 | 485.15(480.70,489.63) | 31.27(30.00,32.57) | -8.56(-9.04,-8.07) |
| Turkmenistan | female | 1426.62(968.55,1817.17) | 241.68(167.53,342.32) | -83.06 | 139.70(131.27,148.56) | 26.21(22.59,30.29) | -5.57(-5.86,-5.28) |
| Tuvalu | female | 29.55(16.75,46.06) | 3.18(1.85,5.13) | -89.24 | 652.57(332.49,1182.66) | 68.19(2.25,397.89) | -6.80(-6.98,-6.62) |
| Uganda | female | 232793.01(133536.83,356530.08) | 98237.78(55144.33,158428.17) | -57.80 | 3691.30(3673.51,3709.16) | 765.90(760.24,771.60) | -4.71(-4.95,-4.47) |
| Ukraine | female | 8749.39(6616.81,11429.69) | 1870.85(1203.96,2848.09) | -78.62 | 149.65(146.14,153.22) | 69.17(65.55,72.94) | -2.50(-3.02,-1.98) |
| United Arab Emirates | female | 909.85(627.22,1281.73) | 445.08(154.57,802.48) | -51.08 | 285.40(265.33,306.65) | 66.16(59.81,73.05) | -4.44(-4.66,-4.23) |
| United Kingdom | female | 141.92(112.71,196.53) | 364.84(38.78,1073.58) | 157.07 | 2.39(1.99,2.86) | 6.00(5.40,6.67) | 6.77(5.46,8.10) |
| United Republic of Tanzania | female | 498201.66(343879.08,668991.86) | 138360.55(85876.42,201686.48) | -72.23 | 6282.22(6262.33,6302.17) | 873.76(868.25,879.30) | -6.25(-6.47,-6.03) |
| United States of America | female | 1303.97(639.65,4411.08) | 2672.09(1433.68,5652.32) | 104.92 | 4.41(4.16,4.67) | 8.65(8.31,9.01) | 1.25(0.31,2.20) |
| United States Virgin Islands | female | 29.23(19.07,42.46) | 2.47(1.42,3.86) | -91.56 | 164.61(105.13,248.13) | 35.68(3.03,150.29) | -4.14(-4.57,-3.72) |
| Uruguay | female | 1218.05(1062.69,1367.83) | 96.85(75.97,121.42) | -92.05 | 266.38(250.09,283.54) | 26.06(20.10,33.31) | -7.28(-8.02,-6.53) |
| Uzbekistan | female | 7793.69(5535.00,10552.12) | 378.55(234.43,672.39) | -95.14 | 138.17(134.58,141.84) | 6.49(5.78,7.27) | -10.75(-11.37,-10.14) |
| Vanuatu | female | 250.92(138.34,406.37) | 158.36(86.71,263.13) | -36.89 | 372.06(310.22,443.58) | 135.25(106.17,170.42) | -3.04(-3.71,-2.36) |
| Venezuela (Bolivarian Republic of) | female | 29907.43(27194.04,32735.11) | 7570.91(5515.12,10231.45) | -74.69 | 579.50(571.46,587.64) | 151.92(147.47,156.48) | -5.08(-6.16,-3.99) |
| Viet Nam | female | 22536.44(15705.08,30962.42) | 3055.69(1736.60,4746.27) | -86.44 | 167.67(165.25,170.12) | 25.52(24.51,26.55) | -6.10(-6.57,-5.63) |
| Yemen | female | 129255.14(80434.50,200956.57) | 48210.19(28108.50,71926.71) | -62.70 | 2807.69(2790.41,2825.05) | 659.99(653.56,666.48) | -4.95(-5.28,-4.62) |
| Zambia | female | 162644.73(109891.39,231379.67) | 36207.49(22864.46,54826.66) | -77.74 | 5435.24(5401.26,5469.39) | 687.22(678.70,695.81) | -6.67(-7.21,-6.13) |
| Zimbabwe | female | 48230.48(33791.47,65037.03) | 87412.86(58949.88,120248.46) | 81.24 | 1608.23(1591.58,1625.03) | 1849.20(1833.09,1865.43) | 1.43(0.98,1.87) |
| Global | male | 18711542.13(15565030.02,23395035.80) | 4468578.07(3496020.00,5524492.65) | -76.12 | 1812.98(1812.05,1813.91) | 408.91(408.48,409.34) | -4.90(-5.46,-4.34) |
| Central Europe, eastern Europe, and central Asia | male | 50787.71(41075.96,61602.32) | 9910.87(7548.02,13123.85) | -80.49 | 91.17(90.30,92.05) | 23.85(23.33,24.37) | -5.11(-5.57,-4.65) |
| High-income | male | 49261.88(44619.10,55582.22) | 12253.48(8085.80,18172.67) | -75.13 | 42.63(42.20,43.07) | 12.53(12.30,12.77) | -2.65(-3.09,-2.21) |
| Latin America and Caribbean | male | 1219395.72(1128008.18,1324073.74) | 152406.11(119853.00,199734.70) | -87.50 | 1364.34(1361.56,1367.13) | 169.79(168.79,170.80) | -6.81(-7.06,-6.56) |
| Southeast Asia, east Asia, and Oceania | male | 2025941.27(1693785.58,2512585.27) | 213046.03(175502.03,255535.07) | -89.48 | 680.93(679.88,681.97) | 94.13(93.68,94.59) | -8.92(-10.75,-7.04) |
| Sub-Saharan Africa | male | 7944933.48(6138212.92,10720917.17) | 2954137.29(2112481.09,3896157.42) | -62.82 | 5368.32(5363.93,5372.71) | 1026.37(1025.03,1027.72) | -5.12(-5.76,-4.48) |
| Andean Latin America | male | 191717.50(149559.49,242230.39) | 20432.54(14875.59,27686.07) | -89.34 | 1725.57(1715.91,1735.27) | 176.76(173.92,179.63) | -7.56(-7.99,-7.14) |
| Australasia | male | 60.96(52.38,71.05) | 30.15(21.61,34.26) | -50.54 | 2.01(1.46,2.71) | 0.94(0.61,1.41) | -1.71(-2.20,-1.22) |
| Caribbean | male | 142083.31(107787.88,181612.94) | 44858.52(29244.41,69145.02) | -68.43 | 1988.91(1977.21,2000.68) | 670.35(663.38,677.37) | -3.36(-3.66,-3.06) |
| Central Asia | male | 27456.35(23007.27,32327.19) | 4572.17(3505.69,5894.57) | -83.35 | 178.67(176.36,181.02) | 28.67(27.79,29.59) | -7.05(-7.68,-6.43) |
| Central Europe | male | 3927.58(3069.60,5073.37) | 809.62(672.20,978.00) | -79.39 | 27.57(26.63,28.54) | 8.75(8.10,9.44) | -4.50(-5.01,-3.98) |
| Central Latin America | male | 493806.70(454332.34,539283.45) | 66981.16(50233.12,89921.30) | -86.44 | 1167.41(1163.62,1171.21) | 159.65(158.17,161.14) | -6.16(-6.32,-6.00) |
| Central Sub-Saharan Africa | male | 1016982.80(733770.95,1526191.44) | 304985.30(184627.36,453219.64) | -70.01 | 6193.26(6179.47,6207.08) | 914.39(910.77,918.03) | -6.06(-6.63,-5.48) |
| East Asia | male | 968478.48(788497.34,1183420.14) | 26512.78(20229.59,33777.51) | -97.26 | 470.60(469.56,471.65) | 21.76(21.47,22.06) | -14.73(-17.87,-11.46) |
| Eastern Europe | male | 19403.79(13921.32,25533.44) | 4529.07(3065.38,6462.20) | -76.66 | 72.93(71.80,74.07) | 26.06(25.21,26.94) | -3.46(-3.87,-3.05) |
| Eastern Sub-Saharan Africa | male | 4491640.97(3451642.15,6079558.22) | 1333244.86(952469.82,1764234.11) | -70.32 | 7633.60(7625.38,7641.83) | 1254.21(1251.76,1256.67) | -5.58(-6.55,-4.60) |
| High-income Asia Pacific | male | 949.35(730.79,1180.45) | 370.62(295.17,459.70) | -60.96 | 4.97(4.61,5.34) | 3.26(2.89,3.66) | -1.33(-1.54,-1.12) |
| High-income North America | male | 3004.32(1189.96,8521.80) | 9020.60(5172.87,14327.80) | 200.25 | 9.16(8.83,9.50) | 25.61(25.08,26.16) | 3.84(2.72,4.97) |
| North Africa and Middle East | male | 522083.58(388738.03,802598.19) | 132590.78(101480.93,168926.28) | -74.60 | 621.27(619.39,623.14) | 131.92(131.13,132.71) | -5.04(-5.38,-4.69) |
| Oceania | male | 7797.40(5862.09,10147.78) | 9471.93(6709.85,12973.99) | 21.48 | 329.15(319.69,338.84) | 224.86(219.08,230.75) | -0.93(-1.29,-0.57) |
| South Asia | male | 6899138.48(5600169.12,8463605.93) | 994233.51(761654.19,1259660.23) | -85.59 | 2715.13(2712.91,2717.34) | 390.58(389.74,391.43) | -5.79(-6.02,-5.55) |
| Southeast Asia | male | 1049665.39(821689.95,1418763.94) | 177061.32(144440.05,215153.02) | -83.13 | 1115.26(1112.89,1117.63) | 189.32(188.33,190.31) | -5.66(-5.74,-5.57) |
| Southern Latin America | male | 43110.90(39602.20,47283.99) | 2149.03(1749.51,2587.49) | -95.02 | 443.16(438.32,448.05) | 24.28(23.03,25.58) | -8.90(-9.44,-8.36) |
| Southern Sub-Saharan Africa | male | 285581.56(227444.50,383018.94) | 167681.18(122280.00,219149.78) | -41.28 | 1762.24(1753.81,1770.69) | 1094.35(1087.99,1100.73) | -1.03(-1.30,-0.77) |
| Tropical Latin America | male | 391788.22(344841.55,441245.86) | 20133.90(15847.03,25397.14) | -94.86 | 1405.87(1401.05,1410.72) | 64.86(63.84,65.88) | -9.93(-10.55,-9.31) |
| Western Europe | male | 2136.34(1792.51,2633.48) | 683.07(536.14,1278.94) | -68.03 | 5.98(5.71,6.25) | 1.91(1.75,2.07) | -3.08(-3.68,-2.47) |
| Western Sub-Saharan Africa | male | 2150728.16(1617461.41,2910907.05) | 1148225.96(776732.22,1553866.03) | -46.61 | 3577.75(3572.01,3583.49) | 866.00(864.18,867.83) | -4.39(-4.56,-4.22) |
| Afghanistan | male | 32973.68(20412.36,58094.81) | 25856.67(16898.56,37666.13) | -21.58 | 1103.83(1090.75,1117.04) | 287.49(283.74,291.28) | -4.55(-5.13,-3.98) |
| Albania | male | 2095.63(1557.43,2891.68) | 93.37(53.13,155.22) | -95.54 | 329.30(313.87,345.33) | 38.84(30.65,48.69) | -7.30(-8.14,-6.44) |
| Algeria | male | 19769.65(12911.07,31660.27) | 3123.39(2172.00,4268.89) | -84.20 | 348.18(343.01,353.41) | 41.36(39.79,42.97) | -7.32(-7.69,-6.95) |
| American Samoa | male | 41.88(26.52,60.62) | 9.48(5.69,14.78) | -77.36 | 165.48(98.69,265.63) | 80.84(22.47,211.76) | -2.43(-2.63,-2.24) |
| Andorra | male | 0.06(0.02,0.12) | 0.00(0.00,0.01) | -97.29 | 1.56(0.00,123.37) | 0.04(0.00,127.17) | -11.23(-12.73,-9.71) |
| Angola | male | 436945.18(289554.83,659952.53) | 95277.01(60187.69,141878.40) | -78.19 | 14789.24(14738.74,14839.88) | 1083.36(1075.69,1091.07) | -8.55(-8.91,-8.19) |
| Antigua and Barbuda | male | 30.49(25.00,37.19) | 13.56(10.96,16.59) | -55.52 | 233.35(139.90,369.51) | 104.73(43.80,216.55) | -2.67(-3.45,-1.88) |
| Argentina | male | 35685.63(32314.48,39534.32) | 1518.31(1223.80,1856.28) | -95.75 | 558.68(551.97,565.45) | 25.25(23.73,26.85) | -9.51(-10.02,-8.99) |
| Armenia | male | 930.56(719.92,1160.77) | 40.81(30.67,59.34) | -95.61 | 164.08(153.41,175.35) | 14.60(10.29,20.19) | -8.43(-8.94,-7.92) |
| Australia | male | 58.75(50.19,68.87) | 27.93(21.18,32.34) | -52.47 | 2.37(1.71,3.22) | 1.05(0.66,1.60) | -2.05(-2.55,-1.55) |
| Austria | male | 22.03(9.59,37.28) | 1.12(0.62,3.81) | -94.91 | 3.37(2.11,5.18) | 0.17(0.00,1.05) | -10.08(-10.98,-9.17) |
| Azerbaijan | male | 5515.56(4269.60,7005.74) | 758.52(544.26,1065.35) | -86.25 | 388.00(377.03,399.22) | 69.36(64.23,74.81) | -6.33(-6.67,-5.98) |
| Bahamas | male | 128.80(102.37,156.62) | 32.46(23.41,47.05) | -74.80 | 263.87(213.59,323.47) | 71.68(44.40,110.71) | -4.10(-4.90,-3.30) |
| Bahrain | male | 135.78(104.04,176.18) | 24.25(18.16,34.30) | -82.14 | 146.70(121.12,176.39) | 14.72(8.87,23.22) | -7.33(-7.47,-7.19) |
| Bangladesh | male | 1997322.75(1546782.18,2465681.67) | 64618.45(45885.09,86342.57) | -96.76 | 6777.45(6767.26,6787.64) | 276.99(274.63,279.36) | -10.18(-10.46,-9.91) |
| Barbados | male | 68.36(54.19,82.85) | 9.42(6.26,13.33) | -86.22 | 187.85(139.93,248.31) | 34.53(13.33,75.21) | -5.76(-6.18,-5.34) |
| Belarus | male | 415.56(217.39,593.60) | 84.10(61.16,113.90) | -79.76 | 32.18(28.84,35.82) | 10.66(8.28,13.56) | -5.16(-6.31,-4.01) |
| Belgium | male | 54.51(40.16,73.33) | 16.39(12.96,20.50) | -69.93 | 5.85(4.36,7.74) | 1.74(0.96,2.94) | -3.42(-4.24,-2.59) |
| Belize | male | 808.01(704.80,922.57) | 212.34(166.99,269.24) | -73.72 | 1582.82(1462.04,1711.61) | 262.12(220.36,310.08) | -5.91(-6.79,-5.03) |
| Benin | male | 67825.82(35300.65,112210.82) | 21213.21(11689.77,35539.12) | -68.72 | 4190.51(4154.01,4227.27) | 543.01(534.67,551.46) | -6.43(-6.68,-6.18) |
| Bermuda | male | 2.55(1.96,3.29) | 0.27(0.19,0.37) | -89.44 | 30.07(2.80,129.03) | 5.45(0.00,138.87) | -5.63(-6.39,-4.87) |
| Bhutan | male | 1346.48(585.97,2817.95) | 57.94(25.02,107.76) | -95.70 | 829.41(780.36,880.95) | 56.04(41.21,74.88) | -8.76(-8.95,-8.56) |
| Bolivia (Plurinational State of) | male | 76157.74(43144.60,112593.18) | 8596.28(5354.62,13170.30) | -88.71 | 3556.76(3525.05,3588.71) | 355.93(346.74,365.32) | -7.30(-7.37,-7.22) |
| Bosnia and Herzegovina | male | 89.23(55.44,131.97) | 9.10(6.09,13.51) | -89.80 | 18.08(14.49,22.34) | 3.72(1.58,7.57) | -7.55(-8.57,-6.52) |
| Botswana | male | 5657.26(3756.39,8015.96) | 4237.54(2618.06,6368.95) | -25.10 | 1307.21(1263.92,1351.67) | 871.21(837.58,905.89) | -1.11(-1.31,-0.91) |
| Brazil | male | 388425.22(341907.82,438258.92) | 17826.68(14107.92,22146.00) | -95.41 | 1448.21(1443.22,1453.22) | 58.44(57.46,59.43) | -10.28(-10.93,-9.62) |
| Brunei Darussalam | male | 7.91(4.72,12.15) | 2.44(1.14,4.58) | -69.10 | 5.47(0.96,18.28) | 1.32(0.00,12.85) | -3.91(-4.25,-3.58) |
| Bulgaria | male | 109.61(68.45,164.48) | 53.28(36.58,77.84) | -51.39 | 7.83(5.94,10.17) | 7.97(5.52,11.23) | -0.29(-0.78,0.20) |
| Burkina Faso | male | 190846.51(127499.89,278029.06) | 110572.30(66533.31,172265.90) | -42.06 | 5797.64(5766.29,5829.11) | 1559.54(1548.81,1570.33) | -4.07(-4.55,-3.59) |
| Burundi | male | 191154.66(99928.94,337296.08) | 43100.75(19439.12,75441.46) | -77.45 | 11378.32(11318.93,11437.97) | 1218.49(1205.35,1231.75) | -6.99(-7.33,-6.65) |
| Cabo Verde | male | 2045.57(1271.53,2935.19) | 98.75(62.54,152.08) | -95.17 | 1789.55(1699.27,1883.74) | 108.95(84.15,139.22) | -9.25(-9.72,-8.78) |
| Cambodia | male | 125288.11(71397.13,206355.09) | 7605.26(5341.61,10718.42) | -93.93 | 4260.33(4234.24,4286.54) | 273.05(266.40,279.84) | -9.70(-10.17,-9.22) |
| Cameroon | male | 47853.11(29785.48,71838.19) | 27856.91(16146.06,42923.37) | -41.79 | 1402.70(1387.76,1417.77) | 319.73(315.33,324.18) | -4.78(-5.12,-4.43) |
| Canada | male | 45.11(30.11,39.85) | 867.56(49.05,1555.52) | 1823.35 | 1.47(1.05,2.00) | 25.82(24.12,27.63) | 9.24(6.93,11.60) |
| Central African Republic | male | 34470.27(21111.00,57000.51) | 38745.06(24737.70,56530.72) | 12.40 | 4397.19(4345.61,4449.26) | 2928.66(2896.21,2961.40) | -0.97(-1.41,-0.52) |
| Chad | male | 131454.61(80479.01,200062.32) | 85484.80(55112.87,127457.22) | -34.97 | 6682.30(6640.00,6724.82) | 1380.72(1369.92,1391.60) | -4.99(-5.13,-4.85) |
| Chile | male | 5798.13(5146.74,6483.85) | 528.73(413.92,743.85) | -90.88 | 197.76(191.72,203.97) | 21.87(19.56,24.39) | -6.68(-7.35,-6.00) |
| China | male | 948894.96(770423.80,1164509.97) | 25454.63(19382.48,32246.71) | -97.32 | 477.12(476.05,478.19) | 21.70(21.40,22.00) | -11.52(-12.24,-10.81) |
| Colombia | male | 55993.67(46404.91,65983.50) | 16955.10(11283.60,24960.83) | -69.72 | 710.42(703.64,717.26) | 205.63(201.59,209.73) | -3.61(-4.25,-2.97) |
| Comoros | male | 6770.71(4295.64,9904.14) | 1026.86(702.36,1506.26) | -84.83 | 4932.51(4798.73,5069.29) | 747.28(696.04,801.48) | -6.26(-6.44,-6.09) |
| Congo | male | 10751.10(6736.17,15435.94) | 4749.72(3033.18,6757.21) | -55.82 | 1750.48(1713.85,1787.74) | 481.12(466.16,496.45) | -4.50(-5.12,-3.87) |
| Cook Islands | male | 0.51(0.30,0.82) | 0.04(0.03,0.07) | -91.11 | 7.16(0.00,155.28) | 1.50(0.00,291.32) | -8.22(-9.34,-7.08) |
| Costa Rica | male | 503.16(432.24,589.72) | 321.08(46.40,516.11) | -36.19 | 74.88(67.85,82.49) | 55.78(49.62,62.60) | 1.64(0.67,2.62) |
| Croatia | male | 20.02(10.63,34.33) | 7.58(4.02,13.11) | -62.12 | 4.75(2.87,7.46) | 2.64(1.04,5.70) | -2.87(-3.71,-2.02) |
| Cuba | male | 750.65(672.06,846.12) | 190.78(147.79,237.50) | -74.58 | 44.96(41.27,48.92) | 16.72(13.92,19.95) | -3.48(-4.82,-2.13) |
| Cyprus | male | 7.32(4.52,11.11) | 0.89(0.24,1.91) | -87.88 | 7.43(3.04,15.93) | 0.79(0.01,5.72) | -6.95(-7.46,-6.45) |
| Czechia | male | 155.05(115.07,230.00) | 152.15(119.97,198.88) | -1.86 | 14.55(12.15,17.31) | 16.54(13.81,19.70) | 0.08(-0.38,0.54) |
| C?te d'Ivoire | male | 82484.54(54088.12,119499.08) | 26511.02(16651.39,39878.08) | -67.86 | 2123.63(2106.81,2140.56) | 342.60(337.83,347.42) | -5.77(-6.11,-5.43) |
| Democratic People's Republic of Korea | male | 18140.25(12284.64,26013.59) | 873.78(467.79,1422.33) | -95.18 | 465.13(457.46,472.91) | 30.90(28.54,33.41) | -18.19(-24.96,-10.82) |
| Democratic Republic of the Congo | male | 528447.56(315568.81,870371.65) | 164057.06(87810.20,287332.58) | -68.95 | 4427.23(4413.57,4440.92) | 756.74(752.64,760.86) | -5.43(-6.23,-4.62) |
| Denmark | male | 21.81(13.62,33.44) | 4.78(3.39,7.91) | -78.09 | 4.78(2.97,7.44) | 0.96(0.28,2.53) | -4.77(-5.62,-3.91) |
| Djibouti | male | 5317.96(3567.33,7892.72) | 2647.05(1787.31,3830.51) | -50.22 | 4793.35(4645.16,4945.29) | 1091.76(1045.14,1139.99) | -4.76(-5.25,-4.27) |
| Dominica | male | 42.73(29.81,58.93) | 13.77(8.14,21.86) | -67.77 | 260.09(177.37,371.52) | 204.53(96.64,386.77) | -0.55(-1.38,0.29) |
| Dominican Republic | male | 43636.65(34098.20,54414.84) | 6343.91(3450.68,9931.42) | -85.46 | 2413.73(2387.37,2440.33) | 325.30(315.96,334.86) | -6.22(-6.78,-5.66) |
| Ecuador | male | 26725.25(23392.87,30350.09) | 4233.42(3017.21,5805.90) | -84.16 | 982.41(968.12,996.87) | 164.98(159.70,170.40) | -6.42(-7.61,-5.22) |
| Egypt | male | 38756.66(27185.79,53592.48) | 13514.36(9808.02,18392.16) | -65.13 | 279.35(276.28,282.44) | 60.24(59.10,61.40) | -3.82(-4.24,-3.41) |
| El Salvador | male | 11627.51(9070.86,14647.74) | 1045.33(680.65,1503.87) | -91.01 | 834.87(817.64,852.41) | 92.06(85.69,98.80) | -7.29(-7.53,-7.05) |
| Equatorial Guinea | male | 3879.50(2323.27,5972.01) | 1109.15(526.33,2028.10) | -71.41 | 2847.43(2745.25,2952.70) | 368.97(345.88,393.28) | -7.72(-8.22,-7.23) |
| Eritrea | male | 150731.65(88641.54,217781.69) | 25461.59(15938.13,37866.15) | -83.11 | 14810.07(14722.24,14898.31) | 1601.06(1577.78,1624.60) | -6.93(-7.22,-6.64) |
| Estonia | male | 141.96(113.35,175.78) | 6.42(5.03,8.16) | -95.48 | 75.53(62.51,90.57) | 5.46(1.83,12.97) | -9.83(-11.11,-8.54) |
| Eswatini | male | 5045.75(3390.39,6947.12) | 2342.02(1464.92,3572.03) | -53.58 | 1749.83(1689.64,1811.74) | 931.41(888.40,976.09) | -1.70(-2.14,-1.27) |
| Ethiopia | male | 1319899.60(904247.01,1912464.51) | 291417.35(208892.89,388647.73) | -77.92 | 8784.37(8767.20,8801.57) | 1144.85(1140.09,1149.62) | -7.24(-7.66,-6.81) |
| Fiji | male | 428.31(287.13,609.37) | 291.04(171.60,488.11) | -32.05 | 213.07(188.10,240.58) | 104.95(87.57,124.97) | -2.10(-2.27,-1.93) |
| Finland | male | 15.99(7.86,27.60) | 1.54(0.59,4.11) | -90.39 | 3.44(1.96,5.72) | 0.42(0.03,2.04) | -6.88(-7.63,-6.13) |
| France | male | 1100.72(968.90,1275.87) | 363.13(294.41,439.28) | -67.01 | 17.85(16.75,19.02) | 5.75(5.10,6.46) | -3.11(-3.73,-2.49) |
| Gabon | male | 2489.19(1589.73,3663.40) | 1047.30(570.03,1710.41) | -57.93 | 935.32(893.77,978.44) | 299.74(279.45,321.18) | -2.90(-3.47,-2.33) |
| Gambia | male | 11182.16(7841.17,15653.16) | 3439.32(2290.59,5186.25) | -69.24 | 3950.85(3865.92,4037.22) | 605.80(582.66,629.64) | -6.32(-6.63,-6.01) |
| Georgia | male | 37.95(20.30,59.55) | 6.11(3.69,7.90) | -83.91 | 6.01(4.23,8.31) | 1.64(0.57,3.80) | -4.31(-5.69,-2.91) |
| Germany | male | 147.38(71.18,250.07) | 55.29(26.58,80.54) | -62.49 | 2.26(1.90,2.66) | 0.87(0.64,1.15) | -2.70(-3.58,-1.81) |
| Ghana | male | 151284.58(92083.57,228181.34) | 44816.41(26320.00,69786.74) | -70.38 | 3593.01(3571.93,3614.19) | 601.24(594.90,607.64) | -5.32(-5.57,-5.07) |
| Greece | male | 0.29(0.17,0.49) | 0.25(0.17,0.36) | -15.35 | 0.03(0.00,0.64) | 0.04(0.00,0.85) | 0.57(0.13,1.00) |
| Greenland | male | 0.70(0.46,1.03) | 0.27(0.07,1.42) | -61.91 | 8.16(0.04,76.84) | 4.34(0.00,86.96) | -2.15(-3.36,-0.92) |
| Grenada | male | 92.07(71.02,117.98) | 26.65(20.60,34.18) | -71.06 | 349.52(264.54,455.05) | 187.18(111.30,299.36) | -2.25(-3.38,-1.11) |
| Guam | male | 42.31(31.61,56.97) | 14.40(9.51,21.81) | -65.97 | 97.19(61.55,148.54) | 33.72(12.53,75.94) | -2.71(-3.06,-2.35) |
| Guatemala | male | 72066.90(62969.83,82007.76) | 13186.60(9870.08,17767.94) | -81.70 | 2519.53(2497.65,2541.56) | 406.88(398.30,415.60) | -5.16(-5.64,-4.67) |
| Guinea | male | 118152.66(76940.47,172761.07) | 54029.03(32593.67,82826.15) | -54.27 | 6014.17(5973.28,6055.29) | 1417.47(1403.73,1431.33) | -4.26(-4.62,-3.90) |
| Guinea-Bissau | male | 15436.16(9692.05,24644.53) | 1803.64(1087.69,2791.26) | -88.32 | 5274.93(5178.50,5372.76) | 326.61(309.57,344.39) | -8.92(-9.23,-8.61) |
| Guyana | male | 4816.93(3879.36,5865.21) | 560.41(403.22,759.76) | -88.37 | 2660.01(2580.71,2741.37) | 455.62(414.80,499.61) | -4.80(-5.24,-4.36) |
| Haiti | male | 80082.15(51656.28,114922.33) | 35277.10(21294.32,56762.79) | -55.95 | 4411.07(4376.78,4445.58) | 1299.46(1284.34,1314.72) | -3.91(-4.10,-3.72) |
| Honduras | male | 7959.85(5597.41,10841.16) | 1216.70(605.05,2121.46) | -84.71 | 598.50(584.08,613.20) | 58.95(55.11,63.00) | -7.34(-7.51,-7.17) |
| Hungary | male | 75.84(50.99,109.20) | 46.06(34.18,58.96) | -39.26 | 7.95(6.21,10.08) | 6.08(4.32,8.39) | -0.73(-1.45,-0.01) |
| Iceland | male | 0.73(0.34,1.28) | 0.17(0.11,0.34) | -76.21 | 2.14(0.01,20.07) | 0.43(0.00,16.24) | -4.80(-5.75,-3.84) |
| India | male | 4331624.53(3361058.13,5597753.81) | 747695.09(566899.58,964627.53) | -82.74 | 2295.32(2292.96,2297.69) | 422.79(421.74,423.84) | -5.03(-5.35,-4.70) |
| Indonesia | male | 439465.17(356202.72,557045.18) | 88438.07(69219.17,111126.13) | -79.88 | 1234.29(1230.28,1238.31) | 249.86(248.02,251.70) | -4.93(-5.01,-4.86) |
| Iran (Islamic Republic of) | male | 51251.49(41910.99,65926.77) | 2570.48(1819.12,3553.41) | -94.98 | 395.74(392.02,399.50) | 25.76(24.68,26.89) | -7.73(-8.14,-7.32) |
| Iraq | male | 20375.70(14274.20,30148.43) | 4688.10(3396.41,6374.14) | -76.99 | 417.25(411.08,423.50) | 70.70(68.53,72.92) | -5.66(-6.11,-5.22) |
| Ireland | male | 15.64(9.03,24.24) | 1.54(1.15,2.53) | -90.14 | 3.91(2.20,6.48) | 0.33(0.02,1.64) | -7.55(-8.13,-6.97) |
| Israel | male | 33.50(22.02,48.43) | 10.02(6.98,12.70) | -70.08 | 4.34(2.96,6.18) | 0.67(0.30,1.32) | -4.95(-5.70,-4.20) |
| Italy | male | 146.16(79.85,236.92) | 45.00(34.22,60.55) | -69.21 | 3.60(3.04,4.25) | 1.29(0.92,1.78) | -2.32(-3.09,-1.54) |
| Jamaica | male | 4177.02(3511.75,4880.14) | 233.90(167.50,310.93) | -94.40 | 708.96(682.33,736.42) | 66.41(56.54,77.64) | -6.77(-8.04,-5.47) |
| Japan | male | 614.70(463.01,822.11) | 341.81(268.24,429.95) | -44.39 | 5.28(4.82,5.77) | 4.26(3.77,4.81) | -0.80(-1.15,-0.46) |
| Jordan | male | 571.19(435.41,736.58) | 186.03(137.27,251.28) | -67.43 | 57.66(52.66,63.04) | 10.43(8.89,12.19) | -5.36(-5.51,-5.20) |
| Kazakhstan | male | 2716.27(2138.51,3252.76) | 521.16(411.77,655.17) | -80.81 | 91.83(88.17,95.61) | 15.25(13.86,16.77) | -7.04(-7.79,-6.28) |
| Kenya | male | 226158.99(159750.96,311925.95) | 97433.52(75053.34,129849.68) | -56.92 | 2930.05(2915.69,2944.47) | 933.48(926.68,940.32) | -3.31(-3.52,-3.11) |
| Kiribati | male | 760.56(525.41,1053.48) | 300.53(192.53,451.39) | -60.48 | 2062.78(1848.99,2296.65) | 665.18(556.69,790.09) | -3.62(-3.75,-3.50) |
| Kuwait | male | 106.02(75.99,142.77) | 3.50(1.85,6.16) | -96.70 | 34.81(27.89,42.95) | 0.85(0.18,2.58) | -12.24(-13.15,-11.32) |
| Kyrgyzstan | male | 1188.77(981.74,1436.21) | 87.19(68.06,112.16) | -92.67 | 127.29(119.71,135.25) | 6.96(5.48,8.75) | -10.58(-11.54,-9.61) |
| Lao People's Democratic Republic | male | 44415.35(25453.03,78076.64) | 4523.82(2772.95,7349.19) | -89.81 | 3978.94(3938.93,4019.29) | 360.26(349.30,371.50) | -7.92(-8.19,-7.64) |
| Latvia | male | 206.61(161.47,267.48) | 12.07(9.53,14.96) | -94.16 | 68.97(59.27,79.88) | 7.77(3.67,14.61) | -8.79(-10.12,-7.44) |
| Lebanon | male | 738.35(499.31,1019.92) | 154.32(84.89,243.16) | -79.10 | 135.44(125.30,146.23) | 26.04(21.91,30.76) | -4.94(-5.45,-4.43) |
| Lesotho | male | 9502.18(6278.53,13218.92) | 5406.37(3070.82,8425.40) | -43.10 | 2121.82(2072.32,2172.27) | 1516.58(1471.09,1563.22) | -0.85(-1.21,-0.48) |
| Liberia | male | 65286.65(40842.51,99000.24) | 9971.29(5810.56,15764.78) | -84.73 | 7574.40(7502.77,7646.59) | 728.43(711.76,745.40) | -8.09(-8.76,-7.42) |
| Libya | male | 1033.48(732.37,1426.03) | 432.23(295.43,598.70) | -58.18 | 101.52(94.78,108.65) | 61.62(55.25,68.56) | -0.36(-0.79,0.08) |
| Lithuania | male | 239.66(187.82,305.63) | 13.04(10.61,15.65) | -94.56 | 53.13(46.10,60.99) | 6.36(3.21,11.46) | -7.93(-9.12,-6.73) |
| Luxembourg | male | 2.71(2.03,3.82) | 0.88(0.64,1.32) | -67.62 | 7.08(1.14,25.93) | 1.48(0.01,12.60) | -4.95(-5.76,-4.14) |
| Madagascar | male | 439833.33(343832.64,536193.29) | 156449.42(99734.51,231115.65) | -64.43 | 12049.97(12007.13,12092.93) | 2419.64(2406.12,2433.22) | -4.50(-4.77,-4.23) |
| Malawi | male | 241914.93(163798.28,356606.71) | 49972.64(29578.32,73137.46) | -79.34 | 7200.43(7165.69,7235.31) | 1071.09(1060.11,1082.15) | -6.25(-6.60,-5.89) |
| Malaysia | male | 9489.80(6781.24,12857.82) | 4021.91(2797.75,5553.02) | -57.62 | 259.94(254.19,265.78) | 103.05(99.53,106.68) | -3.11(-3.29,-2.93) |
| Maldives | male | 1292.08(777.00,2058.05) | 60.27(42.97,81.73) | -95.34 | 2020.51(1900.94,2145.98) | 131.77(98.61,172.83) | -8.49(-9.13,-7.84) |
| Mali | male | 292073.04(177642.83,425149.52) | 322665.04(191763.14,489864.36) | 10.47 | 10608.35(10562.39,10654.47) | 4579.83(4562.13,4597.58) | -2.43(-2.82,-2.05) |
| Malta | male | 1.99(1.08,3.21) | 0.19(0.10,0.43) | -90.68 | 4.97(0.59,19.73) | 0.55(0.00,15.83) | -6.96(-7.60,-6.33) |
| Marshall Islands | male | 43.15(24.18,70.52) | 15.52(9.03,25.35) | -64.03 | 214.07(134.80,326.44) | 95.82(40.50,197.46) | -2.80(-3.34,-2.27) |
| Mauritania | male | 16703.21(9702.73,28989.17) | 2856.04(1934.61,4265.60) | -82.90 | 2571.81(2524.82,2619.48) | 255.76(244.91,267.00) | -7.30(-7.53,-7.07) |
| Mauritius | male | 904.14(772.43,1058.32) | 127.55(95.33,163.14) | -85.89 | 503.92(467.41,542.72) | 123.33(100.43,150.10) | -3.42(-3.86,-2.98) |
| Mexico | male | 296668.40(266030.53,333471.30) | 21991.51(16505.49,29083.35) | -92.59 | 1386.49(1380.70,1392.30) | 120.22(118.39,122.07) | -7.41(-7.58,-7.23) |
| Micronesia (Federated States of) | male | 133.07(76.74,208.54) | 19.43(11.65,30.90) | -85.40 | 322.54(251.55,408.65) | 68.23(31.17,132.70) | -4.89(-5.07,-4.71) |
| Monaco | male | 0.02(0.01,0.04) | 0.01(0.00,0.01) | -64.33 | 1.06(0.00,298.31) | 0.28(0.00,209.27) | -5.31(-6.75,-3.84) |
| Mongolia | male | 682.28(385.16,1137.19) | 61.13(34.91,102.31) | -91.04 | 129.96(119.65,140.98) | 9.45(7.02,12.50) | -9.16(-9.59,-8.72) |
| Montenegro | male | 7.56(5.68,9.94) | 4.12(2.67,6.02) | -45.48 | 7.91(2.78,18.17) | 7.52(2.00,20.59) | 0.67(-0.01,1.35) |
| Morocco | male | 17469.44(11173.52,25634.59) | 1976.37(1191.43,3042.83) | -88.69 | 295.10(290.37,299.90) | 37.70(35.93,39.53) | -6.37(-7.26,-5.47) |
| Mozambique | male | 351906.03(200442.16,556584.92) | 85724.56(52341.77,138187.18) | -75.64 | 9170.71(9135.30,9206.24) | 932.02(924.67,939.42) | -7.43(-7.63,-7.23) |
| Myanmar | male | 155029.41(94175.42,248181.76) | 15709.29(10686.61,23165.24) | -89.87 | 1813.61(1803.40,1823.88) | 175.02(171.96,178.12) | -7.75(-8.03,-7.47) |
| Namibia | male | 10095.81(6975.14,13955.68) | 4745.70(2998.15,7016.56) | -52.99 | 2473.71(2415.13,2533.41) | 868.30(837.79,899.69) | -2.94(-3.20,-2.68) |
| Nauru | male | 12.11(7.28,18.71) | 5.88(3.40,9.67) | -51.46 | 201.70(60.09,515.86) | 116.15(16.30,425.38) | -1.88(-2.88,-0.88) |
| Nepal | male | 228795.52(151248.27,348231.62) | 14703.58(9541.40,21900.18) | -93.57 | 4065.86(4046.99,4084.80) | 301.17(295.98,306.42) | -8.05(-8.22,-7.87) |
| Netherlands | male | 98.85(64.72,154.49) | 84.84(29.21,607.54) | -14.18 | 6.99(5.67,8.57) | 6.25(4.97,7.78) | 0.22(-0.35,0.79) |
| New Zealand | male | 2.21(1.88,2.89) | 2.22(0.34,0.60) | 0.69 | 0.45(0.04,1.96) | 0.41(0.05,1.68) | 3.48(1.64,5.37) |
| Nicaragua | male | 14467.16(10736.34,19830.54) | 1938.86(1145.15,3050.01) | -86.60 | 1292.23(1269.07,1315.73) | 148.88(141.05,157.06) | -7.24(-7.58,-6.89) |
| Niger | male | 233399.73(142011.63,402892.35) | 76700.46(49777.89,116683.83) | -67.14 | 8034.46(7994.77,8074.31) | 882.61(875.24,890.02) | -7.49(-7.86,-7.13) |
| Nigeria | male | 515348.20(379683.59,717478.37) | 241112.21(173745.57,327806.28) | -53.21 | 1786.07(1780.05,1792.11) | 384.76(382.98,386.54) | -4.67(-5.06,-4.28) |
| Niue | male | 1.66(0.93,2.57) | 1.05(0.71,1.56) | -36.46 | 152.43(0.18,1507.36) | 352.23(0.52,3418.96) | -0.59(-1.78,0.61) |
| North Macedonia | male | 58.38(40.80,81.05) | 10.16(6.29,15.55) | -82.60 | 22.12(16.58,29.06) | 6.10(2.72,12.03) | -3.22(-3.61,-2.83) |
| Northern Mariana Islands | male | 6.03(3.69,9.37) | 2.71(1.76,4.09) | -55.01 | 46.15(9.95,147.97) | 28.40(1.36,147.58) | -0.75(-1.01,-0.49) |
| Norway | male | 12.27(6.27,20.23) | 4.16(3.17,5.98) | -66.06 | 2.85(1.46,5.17) | 0.81(0.19,2.42) | -2.89(-4.86,-0.88) |
| Oman | male | 1776.27(1145.68,3052.08) | 371.59(235.34,552.69) | -79.08 | 346.78(329.00,365.31) | 57.17(50.97,63.94) | -4.70(-5.26,-4.14) |
| Pakistan | male | 340049.21(256028.54,450709.50) | 167158.46(124902.65,225331.21) | -50.84 | 1048.09(1044.12,1052.08) | 331.15(329.34,332.96) | -2.79(-3.19,-2.38) |
| Palau | male | 4.37(2.48,6.97) | 1.23(0.72,1.99) | -71.98 | 103.17(14.53,389.56) | 40.74(0.06,409.02) | -2.25(-2.56,-1.94) |
| Palestine | male | 603.35(423.21,842.39) | 144.82(97.55,206.14) | -76.00 | 98.99(90.48,108.12) | 14.85(12.33,17.75) | -5.87(-6.45,-5.30) |
| Panama | male | 2858.40(2378.66,3367.62) | 1299.77(919.40,1797.66) | -54.53 | 484.78(463.34,507.03) | 103.58(94.84,112.94) | -5.55(-6.25,-4.85) |
| Papua New Guinea | male | 4681.65(3187.50,6582.71) | 7770.39(5254.81,10909.75) | 65.98 | 324.89(313.21,336.93) | 243.49(236.71,250.42) | -0.66(-1.11,-0.21) |
| Paraguay | male | 3363.00(2478.66,4575.86) | 2307.21(1491.14,3294.25) | -31.39 | 352.10(339.42,365.17) | 228.78(219.02,238.89) | -1.71(-2.39,-1.02) |
| Peru | male | 88834.51(66219.54,115062.53) | 7602.83(4659.43,11668.64) | -91.44 | 1434.41(1422.55,1446.35) | 119.03(115.89,122.25) | -8.37(-8.86,-7.88) |
| Philippines | male | 157795.91(129604.32,202348.17) | 41295.00(32850.15,51224.11) | -73.83 | 1008.76(1003.08,1014.46) | 201.90(199.63,204.19) | -4.59(-4.92,-4.25) |
| Poland | male | 333.17(221.20,527.74) | 98.86(73.37,138.30) | -70.33 | 7.91(7.06,8.84) | 3.50(2.82,4.32) | -3.31(-3.92,-2.69) |
| Portugal | male | 99.84(77.00,126.45) | 16.29(12.27,21.40) | -83.68 | 11.28(9.11,13.86) | 2.41(1.33,4.09) | -5.19(-6.43,-3.92) |
| Puerto Rico | male | 533.22(466.89,611.27) | 81.37(64.67,101.81) | -84.74 | 89.85(81.23,99.21) | 45.88(35.25,58.81) | -2.70(-4.01,-1.36) |
| Qatar | male | 20.72(14.75,28.28) | 13.35(9.30,19.21) | -35.58 | 24.14(14.20,39.15) | 4.13(2.00,7.71) | -5.24(-5.75,-4.73) |
| Republic of Korea | male | 257.51(177.62,361.18) | 24.18(15.60,33.82) | -90.61 | 3.66(3.16,4.23) | 0.71(0.41,1.16) | -4.95(-5.10,-4.80) |
| Republic of Moldova | male | 507.85(409.51,630.32) | 39.91(30.33,53.34) | -92.14 | 76.90(69.78,84.56) | 16.38(11.34,23.00) | -6.27(-7.58,-4.94) |
| Romania | male | 695.63(551.10,936.20) | 251.56(203.45,306.50) | -63.84 | 25.78(23.72,27.97) | 15.79(13.71,18.11) | -1.70(-2.38,-1.03) |
| Russian Federation | male | 7726.92(5391.38,10512.91) | 1260.26(874.20,1886.31) | -83.69 | 43.74(42.68,44.83) | 9.01(8.43,9.61) | -6.06(-6.78,-5.34) |
| Rwanda | male | 140550.73(95267.82,205601.15) | 21399.60(13790.98,30975.90) | -84.77 | 6920.73(6879.82,6961.84) | 723.35(712.16,734.68) | -8.71(-9.47,-7.94) |
| Saint Kitts and Nevis | male | 81.95(69.74,95.34) | 20.64(14.91,28.51) | -74.81 | 876.68(662.39,1143.03) | 247.27(121.87,456.65) | -3.73(-4.78,-2.67) |
| Saint Lucia | male | 176.45(138.25,214.56) | 22.40(15.72,31.84) | -87.31 | 489.49(404.70,588.00) | 122.02(68.96,203.19) | -4.49(-5.48,-3.48) |
| Saint Vincent and the Grenadines | male | 259.18(204.12,324.85) | 43.84(31.69,59.73) | -83.09 | 1076.72(928.96,1242.90) | 271.92(178.66,399.27) | -4.66(-5.63,-3.68) |
| Samoa | male | 154.37(89.86,235.53) | 54.68(31.20,86.64) | -64.58 | 175.88(135.17,226.07) | 50.98(31.46,79.11) | -3.84(-4.00,-3.68) |
| San Marino | male | 0.04(0.02,0.06) | 0.00(0.00,0.01) | -90.41 | 1.98(0.00,287.34) | 0.18(0.00,264.38) | -6.79(-7.59,-5.99) |
| Sao Tome and Principe | male | 1929.27(1321.54,2616.40) | 150.83(90.23,219.70) | -92.18 | 5350.02(5074.47,5637.47) | 409.82(342.83,486.65) | -7.95(-8.54,-7.37) |
| Saudi Arabia | male | 19458.43(13380.83,28191.61) | 2576.13(1643.19,3719.18) | -86.76 | 515.83(507.81,523.94) | 71.23(68.31,74.24) | -5.91(-6.12,-5.70) |
| Senegal | male | 65707.52(38900.04,101382.22) | 8498.57(3973.54,15163.08) | -87.07 | 2822.68(2797.17,2848.37) | 220.08(214.70,225.56) | -8.26(-8.78,-7.73) |
| Serbia | male | 149.42(106.90,207.00) | 40.29(26.19,57.41) | -73.04 | 13.36(11.07,16.03) | 7.48(5.27,10.35) | -1.10(-1.83,-0.37) |
| Seychelles | male | 18.69(13.96,24.74) | 7.39(5.02,10.59) | -60.45 | 141.64(80.24,235.08) | 54.37(19.70,123.66) | -1.83(-2.36,-1.29) |
| Sierra Leone | male | 116471.75(76992.88,164074.96) | 105196.08(63775.98,170719.59) | -9.68 | 9152.79(9091.40,9214.52) | 4304.00(4272.89,4335.30) | -1.63(-2.09,-1.16) |
| Singapore | male | 69.24(28.03,126.76) | 2.19(0.84,8.96) | -96.83 | 19.88(14.83,26.21) | 0.39(0.03,1.82) | -12.77(-13.46,-12.08) |
| Slovakia | male | 68.81(52.38,91.75) | 27.76(19.98,38.99) | -59.66 | 10.53(8.03,13.61) | 5.81(3.69,8.78) | -1.67(-2.32,-1.02) |
| Slovenia | male | 6.44(3.46,10.95) | 3.53(2.24,5.35) | -45.16 | 3.55(1.27,8.00) | 2.08(0.39,6.66) | -2.11(-2.71,-1.52) |
| Solomon Islands | male | 631.92(393.10,963.82) | 361.67(245.63,529.20) | -42.77 | 433.68(390.39,480.88) | 155.21(134.20,178.75) | -3.02(-3.24,-2.79) |
| Somalia | male | 212115.76(129421.01,316575.20) | 154890.50(92143.21,230364.00) | -26.98 | 8983.47(8940.55,9026.55) | 2435.70(2422.40,2449.05) | -2.92(-5.78,0.04) |
| South Africa | male | 195504.94(152281.54,275454.26) | 65797.71(48171.14,83820.55) | -66.34 | 1635.92(1625.87,1646.02) | 725.65(719.14,732.20) | -2.05(-2.30,-1.80) |
| South Sudan | male | 233093.80(147276.50,348374.77) | 122232.21(75252.83,201377.51) | -47.56 | 15149.80(15082.06,15217.77) | 4839.90(4809.00,4870.97) | -3.74(-4.48,-2.99) |
| Spain | male | 37.38(31.68,43.34) | 20.52(16.50,24.98) | -45.10 | 1.11(0.77,1.58) | 0.70(0.41,1.12) | -1.32(-1.81,-0.83) |
| Sri Lanka | male | 10405.01(7918.75,13303.83) | 3848.12(2601.85,5512.46) | -63.02 | 369.10(361.14,377.19) | 161.89(156.21,167.73) | -2.69(-2.95,-2.42) |
| Sudan | male | 158697.09(88803.70,303619.30) | 33008.56(22347.00,48762.40) | -79.20 | 2527.03(2512.35,2541.77) | 340.02(335.78,344.30) | -6.14(-6.49,-5.80) |
| Suriname | male | 729.10(511.32,1004.39) | 145.07(92.65,224.35) | -80.10 | 1012.95(934.05,1097.24) | 173.64(141.89,210.81) | -6.02(-6.42,-5.62) |
| Sweden | male | 25.19(12.98,40.48) | 5.01(3.91,8.01) | -80.10 | 2.86(1.85,4.31) | 0.56(0.17,1.41) | -4.82(-5.74,-3.88) |
| Switzerland | male | 23.76(16.82,35.70) | 5.87(4.49,8.31) | -75.28 | 3.85(2.43,5.90) | 0.81(0.26,1.97) | -4.45(-5.04,-3.86) |
| Syrian Arab Republic | male | 21029.56(16109.49,27149.39) | 3512.42(2455.56,4750.51) | -83.30 | 626.76(617.53,636.09) | 202.34(194.61,210.31) | -2.78(-3.35,-2.20) |
| Taiwan (Province of China) | male | 1443.26(1248.76,1678.16) | 184.37(148.95,225.95) | -87.23 | 48.89(46.11,51.81) | 12.94(10.97,15.17) | -4.87(-6.52,-3.18) |
| Tajikistan | male | 5499.47(4243.09,7271.78) | 1801.19(1245.76,2642.08) | -67.25 | 366.26(355.52,377.25) | 82.86(78.71,87.19) | -6.15(-7.21,-5.07) |
| Thailand | male | 9987.32(7005.51,13376.45) | 3144.73(2291.25,4322.76) | -68.51 | 121.59(118.99,124.24) | 64.49(61.97,67.10) | -0.86(-1.31,-0.41) |
| Timor-Leste | male | 15153.79(7808.07,29339.59) | 2521.42(1657.48,3786.62) | -83.36 | 7035.99(6915.08,7158.66) | 841.12(804.92,878.62) | -7.32(-7.75,-6.90) |
| Togo | male | 25172.11(14999.71,41475.93) | 5236.64(3031.59,8661.46) | -79.20 | 2429.72(2395.77,2464.05) | 260.99(252.84,269.35) | -7.27(-7.52,-7.02) |
| Tokelau | male | 0.70(0.41,1.13) | 0.89(0.50,1.37) | 28.56 | 167.54(0.09,1927.37) | 362.09(0.23,3777.98) | -2.93(-5.03,-0.79) |
| Tonga | male | 104.38(66.53,161.85) | 51.24(31.92,79.00) | -50.91 | 207.24(151.22,279.08) | 112.52(70.12,172.59) | -1.52(-1.84,-1.21) |
| Trinidad and Tobago | male | 811.21(660.98,973.32) | 109.55(82.53,143.28) | -86.50 | 360.85(333.02,390.46) | 74.18(58.67,92.70) | -5.49(-5.74,-5.23) |
| Tunisia | male | 2148.15(1496.57,2974.68) | 236.27(161.07,337.17) | -89.00 | 126.88(121.13,132.86) | 15.42(13.31,17.80) | -6.39(-6.72,-6.06) |
| Türkiye | male | 47047.00(30550.67,71680.84) | 3331.60(2272.03,4890.33) | -92.92 | 403.12(399.18,407.09) | 30.87(29.64,32.14) | -8.21(-8.45,-7.96) |
| Turkmenistan | male | 1727.13(1318.79,2182.19) | 222.38(156.71,317.08) | -87.12 | 177.23(167.94,186.94) | 26.10(22.58,30.05) | -6.45(-6.90,-6.01) |
| Tuvalu | male | 30.06(19.15,47.37) | 2.95(1.77,4.64) | -90.17 | 752.98(436.12,1250.97) | 82.60(6.08,390.39) | -6.66(-6.90,-6.41) |
| Uganda | male | 301516.75(179854.84,472507.25) | 112961.55(59673.77,185960.58) | -62.54 | 4417.67(4398.40,4437.02) | 860.03(854.09,866.00) | -5.47(-5.74,-5.19) |
| Ukraine | male | 10165.23(7049.85,14405.44) | 3113.29(1900.37,4725.35) | -69.37 | 171.78(168.09,175.53) | 112.48(108.03,117.07) | -1.10(-1.47,-0.72) |
| United Arab Emirates | male | 971.54(633.91,1413.57) | 552.24(296.36,937.80) | -43.16 | 289.79(270.03,310.67) | 79.40(72.51,86.80) | -3.79(-4.06,-3.52) |
| United Kingdom | male | 266.39(174.53,400.01) | 44.57(31.46,78.99) | -83.27 | 4.52(3.98,5.12) | 0.75(0.53,1.03) | -4.63(-5.09,-4.15) |
| United Republic of Tanzania | male | 519639.88(355410.61,719555.70) | 130294.29(77158.05,194057.30) | -74.93 | 6375.33(6355.46,6395.25) | 753.28(748.21,758.37) | -6.93(-7.14,-6.71) |
| United States of America | male | 2958.45(1156.36,8484.83) | 8152.63(4604.91,12922.29) | 175.57 | 9.95(9.59,10.33) | 25.59(25.02,26.16) | 3.56(2.48,4.66) |
| United States Virgin Islands | male | 44.67(27.61,66.24) | 3.01(1.66,4.92) | -93.25 | 219.57(152.01,309.72) | 32.61(3.17,135.36) | -5.56(-6.05,-5.07) |
| Uruguay | male | 1625.09(1428.87,1844.17) | 101.88(76.60,132.35) | -93.73 | 358.01(339.44,377.42) | 22.99(17.55,29.66) | -8.41(-9.19,-7.63) |
| Uzbekistan | male | 9158.34(6510.28,12079.38) | 1073.68(779.44,1444.73) | -88.28 | 158.09(154.46,161.80) | 17.30(16.23,18.43) | -8.63(-9.13,-8.11) |
| Vanuatu | male | 222.93(142.23,337.27) | 143.50(80.16,230.88) | -35.63 | 329.90(274.40,394.26) | 122.28(95.38,154.92) | -2.95(-3.45,-2.46) |
| Venezuela (Bolivarian Republic of) | male | 31661.65(28172.77,35553.30) | 9026.22(6398.12,12556.72) | -71.49 | 622.31(614.19,630.52) | 181.11(176.39,185.93) | -5.11(-6.22,-3.98) |
| Viet Nam | male | 78902.19(50206.46,120359.92) | 5511.54(3744.26,7910.56) | -93.01 | 535.27(531.13,539.44) | 41.82(40.59,43.08) | -8.28(-8.42,-8.15) |
| Yemen | male | 86864.42(52075.09,149410.86) | 36190.44(25697.29,49510.43) | -58.34 | 1846.68(1833.18,1860.25) | 459.37(454.08,464.71) | -4.87(-5.15,-4.58) |
| Zambia | male | 147826.17(88886.39,245320.00) | 37072.26(19863.38,58810.46) | -74.92 | 5262.67(5229.32,5296.20) | 717.67(708.96,726.46) | -6.27(-7.07,-5.48) |
| Zimbabwe | male | 59775.61(40276.56,83833.23) | 85151.84(53651.93,123885.63) | 42.45 | 2025.88(2007.36,2044.54) | 1961.84(1945.40,1978.39) | 0.59(0.19,1.00) |

**Stable 4. Incidence cases and ASIR of Protein-energy malnutrition in 1990 and 2021 and its trends.**

| location | sex | number | | | Age-standardized rate | | |
| --- | --- | --- | --- | --- | --- | --- | --- |
| 1990 | 2021 | change rate in case(%) | 1990 | 2021 | EAPC |
| Global | both | 56211712.57(41170616.11,72662955.75) | 34509841.70(25924285.50,44393403.88) | -38.61 | 2886.42(2885.57,2887.28) | 1684.50(1683.87,1685.13) | -1.23(-1.71,-0.75) |
| Central Europe, eastern Europe, and central Asia | both | 1734536.72(1305378.97,2230660.47) | 930190.70(698404.60,1195437.42) | -46.37 | 1593.27(1590.66,1595.88) | 1149.14(1146.59,1151.70) | -0.87(-1.07,-0.67) |
| High-income | both | 1573470.64(1115454.92,2083782.98) | 1020089.01(741892.72,1333559.34) | -35.17 | 822.37(821.01,823.73) | 568.54(567.37,569.71) | -0.57(-0.93,-0.21) |
| Latin America and Caribbean | both | 1552979.41(1193176.90,1974409.48) | 732228.47(561111.27,923453.33) | -52.85 | 1020.86(1019.08,1022.63) | 504.61(503.35,505.87) | -1.38(-1.79,-0.96) |
| Southeast Asia, east Asia, and Oceania | both | 12366407.41(9116516.40,15930059.83) | 5463499.59(4168392.28,7060681.36) | -55.82 | 2269.83(2268.40,2271.25) | 1241.88(1240.74,1243.02) | -1.38(-1.82,-0.94) |
| Sub-Saharan Africa | both | 8361261.73(6198839.43,10800518.80) | 8107560.36(5768131.68,10683216.81) | -3.03 | 2798.37(2796.15,2800.58) | 1402.24(1401.11,1403.37) | -2.01(-2.41,-1.61) |
| Andean Latin America | both | 111035.19(87360.22,137072.95) | 54560.48(41599.56,68546.84) | -50.86 | 629.10(624.87,633.36) | 275.86(273.29,278.45) | -2.49(-2.81,-2.17) |
| Australasia | both | 26710.62(19312.47,35768.31) | 23624.46(17732.57,31341.81) | -11.55 | 561.86(554.67,569.12) | 407.95(402.44,413.52) | -0.66(-0.89,-0.42) |
| Caribbean | both | 147258.51(114193.46,181593.29) | 88796.63(63098.89,117782.89) | -39.70 | 1124.62(1118.10,1131.16) | 717.89(712.52,723.29) | -1.28(-1.65,-0.91) |
| Central Asia | both | 506093.04(360111.34,673306.59) | 352867.43(259248.43,462211.09) | -30.28 | 1714.26(1708.96,1719.58) | 1167.32(1163.10,1171.55) | -1.58(-1.80,-1.36) |
| Central Europe | both | 330086.85(251748.51,426391.57) | 162706.28(121237.91,212065.68) | -50.71 | 1159.44(1155.12,1163.76) | 929.45(924.62,934.31) | -0.37(-0.60,-0.15) |
| Central Latin America | both | 816107.77(632263.18,1023163.77) | 338118.06(261902.62,420984.33) | -58.57 | 1170.53(1167.72,1173.35) | 544.61(542.61,546.61) | -1.58(-1.97,-1.18) |
| Central Sub-Saharan Africa | both | 859084.95(611692.31,1129078.27) | 713370.89(498881.67,962656.68) | -16.96 | 2496.65(2490.43,2502.88) | 1018.20(1015.42,1020.99) | -2.95(-3.61,-2.29) |
| East Asia | both | 5910693.47(4329909.53,7566282.28) | 2420700.02(1809115.78,3215537.24) | -59.05 | 1658.25(1656.76,1659.74) | 915.59(914.36,916.83) | -0.98(-1.39,-0.56) |
| Eastern Europe | both | 898356.83(679839.09,1148524.08) | 414616.99(311231.87,529782.39) | -53.85 | 1736.51(1732.58,1740.45) | 1227.58(1223.43,1231.74) | -0.68(-0.94,-0.41) |
| Eastern Sub-Saharan Africa | both | 3288211.64(2476296.91,4170993.86) | 2751437.57(2003488.75,3549452.62) | -16.32 | 2757.03(2753.56,2760.51) | 1287.04(1285.26,1288.82) | -2.61(-2.87,-2.36) |
| High-income Asia Pacific | both | 460673.47(338729.77,597717.90) | 201879.10(150050.51,260942.95) | -56.18 | 1340.49(1336.27,1344.72) | 938.64(934.19,943.12) | -0.70(-0.92,-0.48) |
| High-income North America | both | 417157.33(284881.41,587999.05) | 325583.51(229644.84,438458.62) | -21.95 | 655.19(653.13,657.25) | 469.55(467.87,471.24) | -0.38(-0.93,0.18) |
| North Africa and Middle East | both | 3569675.60(2783070.01,4456105.85) | 2856119.48(2138289.30,3665064.29) | -19.99 | 2318.04(2315.34,2320.75) | 1518.95(1516.99,1520.91) | -1.19(-1.58,-0.81) |
| Oceania | both | 78058.67(56542.30,100099.84) | 158351.81(115424.76,201047.30) | 102.86 | 2399.88(2380.55,2419.34) | 2497.73(2483.61,2511.92) | 0.54(0.13,0.96) |
| South Asia | both | 27053381.05(19128692.68,35366972.90) | 15400154.08(11564999.14,19981470.03) | -43.07 | 5471.41(5469.05,5473.76) | 3185.45(3183.68,3187.22) | -1.08(-1.61,-0.55) |
| Southeast Asia | both | 6377655.27(4659077.67,8208973.49) | 2884447.77(2101708.26,3768934.00) | -54.77 | 3476.28(3473.23,3479.33) | 1636.41(1634.28,1638.54) | -2.02(-2.45,-1.59) |
| Southern Latin America | both | 97830.19(73032.21,124849.90) | 49905.21(39210.80,62532.20) | -48.99 | 659.37(654.85,663.92) | 370.61(367.08,374.17) | -1.81(-2.06,-1.57) |
| Southern Sub-Saharan Africa | both | 380795.13(272620.35,499985.03) | 211383.31(162565.56,265875.86) | -44.49 | 1614.37(1608.60,1620.15) | 799.54(795.66,803.43) | -2.28(-2.41,-2.15) |
| Tropical Latin America | both | 478577.95(351427.29,630499.98) | 250753.31(187149.21,321615.35) | -47.60 | 900.35(897.57,903.13) | 490.54(488.47,492.61) | -0.72(-1.26,-0.18) |
| Western Europe | both | 571099.04(399489.96,768202.04) | 419096.75(299390.76,555068.01) | -26.62 | 804.20(802.02,806.39) | 608.59(606.66,610.52) | -0.21(-0.53,0.11) |
| Western Sub-Saharan Africa | both | 3833170.01(2846011.09,4959728.10) | 4431368.58(3078829.25,5906648.45) | 15.61 | 3170.43(3166.71,3174.15) | 1654.55(1652.74,1656.35) | -1.55(-2.04,-1.07) |
| Afghanistan | both | 108971.68(77528.79,143893.84) | 199163.93(139759.06,263428.67) | 82.77 | 2150.15(2135.57,2164.82) | 1258.59(1252.41,1264.80) | -1.88(-2.46,-1.28) |
| Albania | both | 27459.66(19071.33,37372.59) | 6226.12(4595.44,8108.15) | -77.33 | 2469.36(2437.64,2501.41) | 1503.82(1464.49,1543.99) | -2.18(-2.62,-1.73) |
| Algeria | both | 262712.56(192370.66,339176.33) | 218682.08(168005.73,283790.66) | -16.76 | 2377.78(2367.69,2387.89) | 1552.64(1545.55,1559.75) | -2.35(-2.67,-2.04) |
| American Samoa | both | 190.62(142.08,240.82) | 94.46(72.53,118.81) | -50.45 | 826.88(700.86,970.21) | 721.22(564.99,909.69) | -0.19(-0.53,0.15) |
| Andorra | both | 63.17(41.62,89.91) | 71.57(46.85,105.62) | 13.30 | 686.63(518.44,898.50) | 688.45(527.82,891.34) | 0.45(0.22,0.69) |
| Angola | both | 230826.25(158612.87,296497.82) | 155107.33(110726.58,204557.42) | -32.80 | 3451.75(3435.09,3468.48) | 788.39(783.68,793.13) | -4.50(-4.83,-4.17) |
| Antigua and Barbuda | both | 345.57(266.27,431.06) | 175.47(135.99,220.36) | -49.22 | 1763.36(1556.60,1990.92) | 1007.03(848.47,1188.60) | -1.11(-1.57,-0.64) |
| Argentina | both | 74794.13(55411.77,97272.65) | 39425.34(30809.69,49622.20) | -47.29 | 745.25(739.37,751.15) | 419.21(414.69,423.77) | -1.86(-2.12,-1.60) |
| Armenia | both | 11042.30(7684.15,14924.22) | 5759.03(4158.38,7548.25) | -47.85 | 950.76(931.45,970.38) | 1061.05(1031.97,1090.77) | 1.00(0.69,1.31) |
| Australia | both | 20556.83(14860.21,27614.00) | 18965.97(14321.57,25071.97) | -7.74 | 526.29(518.60,534.07) | 395.14(389.19,401.17) | -0.61(-0.80,-0.41) |
| Austria | both | 10912.50(7498.97,15381.99) | 10529.08(6496.52,15636.34) | -3.51 | 807.98(792.18,824.04) | 803.17(787.39,819.21) | 0.26(0.14,0.38) |
| Azerbaijan | both | 49111.17(33084.86,68665.00) | 41286.69(32142.10,51316.73) | -15.93 | 1818.51(1800.87,1836.29) | 1866.67(1847.15,1886.34) | 0.62(0.16,1.07) |
| Bahamas | both | 542.91(401.20,697.09) | 378.73(282.88,494.53) | -30.24 | 680.82(618.79,747.71) | 539.74(479.76,605.47) | -0.31(-0.65,0.04) |
| Bahrain | both | 4191.29(3327.40,5229.57) | 4001.52(3096.49,5033.05) | -4.53 | 2296.65(2219.42,2375.98) | 1338.99(1294.64,1384.57) | -1.12(-1.43,-0.80) |
| Bangladesh | both | 2969249.19(2342666.07,3543327.80) | 1261874.25(1027953.33,1511168.58) | -57.50 | 4835.12(4828.69,4841.55) | 2770.15(2764.64,2775.66) | -1.21(-1.54,-0.89) |
| Barbados | both | 779.30(601.21,985.30) | 399.05(306.96,508.42) | -48.79 | 1243.35(1145.97,1347.17) | 889.93(793.65,995.18) | -0.21(-0.66,0.24) |
| Belarus | both | 13429.51(9448.15,18194.56) | 7740.08(5492.33,10408.15) | -42.37 | 620.78(609.99,631.72) | 562.93(549.97,576.12) | 0.14(-0.06,0.34) |
| Belgium | both | 12248.40(8641.70,16618.52) | 9905.43(6850.44,13640.23) | -19.13 | 673.93(661.43,686.63) | 510.52(500.05,521.18) | -0.29(-0.54,-0.04) |
| Belize | both | 1035.57(777.75,1332.31) | 709.89(542.79,956.51) | -31.45 | 1194.10(1115.17,1277.33) | 618.01(568.75,670.54) | -1.63(-1.88,-1.37) |
| Benin | both | 91550.40(72639.66,111733.11) | 102729.14(70088.11,136658.70) | 12.21 | 2708.31(2687.79,2728.97) | 1264.75(1255.59,1273.97) | -1.91(-2.29,-1.53) |
| Bermuda | both | 88.51(67.69,112.55) | 51.83(39.66,66.81) | -41.44 | 698.66(548.89,878.58) | 621.51(450.00,841.47) | -0.24(-0.47,0.00) |
| Bhutan | both | 6076.35(4143.61,8296.50) | 2847.74(2026.03,3738.77) | -53.13 | 1964.76(1908.39,2022.45) | 1448.85(1391.66,1507.95) | -0.69(-0.92,-0.46) |
| Bolivia (Plurinational State of) | both | 27439.84(20339.77,35287.68) | 14301.63(10636.10,18401.05) | -47.88 | 826.94(815.80,838.21) | 371.64(364.84,378.55) | -2.22(-2.53,-1.91) |
| Bosnia and Herzegovina | both | 11679.97(7946.79,16567.79) | 4377.41(3080.28,5842.57) | -62.52 | 1099.68(1078.64,1121.05) | 919.69(890.66,949.49) | -0.87(-1.16,-0.58) |
| Botswana | both | 14593.63(10168.84,19214.05) | 11614.61(8798.98,15231.41) | -20.41 | 2086.39(2047.91,2125.45) | 1487.29(1456.83,1518.27) | -0.83(-1.00,-0.65) |
| Brazil | both | 473965.60(347748.08,624489.22) | 245155.34(182922.01,314475.04) | -48.28 | 922.35(919.49,925.22) | 499.66(497.53,501.79) | -0.72(-1.27,-0.18) |
| Brunei Darussalam | both | 1501.51(1102.23,1939.81) | 1019.56(776.49,1294.54) | -32.10 | 1296.35(1220.50,1375.85) | 920.24(856.17,988.14) | -0.50(-0.78,-0.23) |
| Bulgaria | both | 20187.09(14196.58,27424.60) | 27355.19(19504.24,36534.38) | 35.51 | 1022.39(1006.41,1038.59) | 2576.15(2544.10,2608.56) | 3.87(3.32,4.42) |
| Burkina Faso | both | 257768.62(191975.92,326292.28) | 299946.65(201118.13,415498.27) | 16.36 | 3777.27(3759.98,3794.63) | 2081.17(2072.29,2090.09) | -1.73(-2.54,-0.92) |
| Burundi | both | 93041.60(68339.98,117861.71) | 91128.82(69824.90,113567.00) | -2.06 | 2639.20(2619.47,2659.05) | 1180.01(1170.78,1189.29) | -3.49(-4.61,-2.35) |
| Cabo Verde | both | 2823.98(2307.89,3412.22) | 489.83(336.74,642.47) | -82.65 | 1487.56(1425.77,1551.45) | 345.63(312.11,381.92) | -4.62(-4.92,-4.31) |
| Cambodia | both | 287780.12(197979.24,375673.51) | 67392.33(44874.48,91092.44) | -76.58 | 5024.59(5003.81,5045.43) | 1264.84(1253.99,1275.76) | -3.94(-4.44,-3.45) |
| Cameroon | both | 101616.50(76526.05,128968.45) | 127760.94(88822.92,177814.01) | 25.73 | 1461.98(1451.44,1472.58) | 763.60(758.63,768.58) | -1.48(-2.11,-0.84) |
| Canada | both | 27676.51(19807.65,38046.38) | 30822.32(19591.76,45728.70) | 11.37 | 462.40(456.69,468.16) | 468.50(463.10,473.96) | 0.74(0.30,1.18) |
| Central African Republic | both | 30180.83(19655.33,41802.12) | 54576.31(40776.17,69082.14) | 80.83 | 1776.58(1752.80,1800.62) | 1799.03(1780.83,1817.38) | -0.31(-0.94,0.33) |
| Chad | both | 192515.09(146059.33,243734.12) | 400837.87(304535.84,514014.78) | 108.21 | 4292.17(4269.29,4315.15) | 3008.09(2996.86,3019.35) | -1.02(-1.26,-0.78) |
| Chile | both | 16830.48(12381.22,21409.89) | 7654.00(5302.50,10048.48) | -54.52 | 413.24(406.45,420.12) | 213.69(208.69,218.79) | -2.19(-2.45,-1.92) |
| China | both | 5622488.91(4123591.18,7223879.25) | 2342235.90(1744789.64,3111046.38) | -58.34 | 1634.12(1632.61,1635.62) | 912.42(911.18,913.67) | -0.81(-1.25,-0.36) |
| Colombia | both | 80154.78(60133.20,98719.03) | 26861.44(19901.48,35339.15) | -66.49 | 622.80(618.02,627.61) | 241.87(238.67,245.11) | -2.18(-2.52,-1.84) |
| Comoros | both | 7858.21(5393.31,10214.35) | 3927.69(2916.22,5182.95) | -50.02 | 2941.19(2866.21,3017.73) | 1466.80(1413.70,1521.46) | -2.25(-2.56,-1.95) |
| Congo | both | 18730.19(13213.12,24114.40) | 16863.28(11660.89,22598.19) | -9.97 | 1459.28(1435.19,1483.68) | 814.11(799.94,828.46) | -1.45(-2.06,-0.84) |
| Cook Islands | both | 54.76(41.92,70.33) | 24.85(18.68,32.26) | -54.62 | 789.26(577.37,1057.61) | 640.42(392.65,1000.03) | -0.36(-0.63,-0.08) |
| Costa Rica | both | 5002.49(3494.52,6898.06) | 11578.86(8332.78,15075.71) | 131.46 | 424.81(412.12,437.81) | 1103.77(1083.02,1124.86) | 4.30(3.18,5.43) |
| Croatia | both | 9023.09(6330.14,12103.06) | 5560.69(3853.07,7421.22) | -38.37 | 951.34(930.14,972.92) | 954.80(927.97,982.25) | -0.09(-0.31,0.13) |
| Cuba | both | 16648.70(12466.44,21673.29) | 7150.65(5335.41,9517.04) | -57.05 | 551.85(542.33,561.50) | 438.94(428.03,450.06) | -0.59(-0.90,-0.28) |
| Cyprus | both | 1697.02(1189.27,2354.42) | 1346.20(976.34,1770.13) | -20.67 | 857.33(814.66,901.82) | 604.94(571.40,640.05) | -0.71(-0.89,-0.54) |
| Czechia | both | 22775.16(17828.41,28193.55) | 14425.09(10283.83,19176.68) | -36.66 | 1104.44(1088.55,1120.52) | 853.91(838.98,869.06) | -0.22(-0.50,0.05) |
| C?te d'Ivoire | both | 169416.78(118295.87,222524.55) | 126063.65(85334.67,176353.55) | -25.59 | 2151.71(2139.80,2163.66) | 857.42(851.82,863.06) | -2.31(-2.85,-1.77) |
| Democratic People's Republic of Korea | both | 223749.55(152374.96,292043.86) | 42936.66(31679.09,55873.89) | -80.81 | 3154.26(3139.22,3169.34) | 933.69(923.83,943.64) | -5.23(-6.11,-4.34) |
| Democratic Republic of the Congo | both | 565472.43(397706.96,763888.23) | 481545.12(320741.07,663126.30) | -14.84 | 2367.98(2360.72,2375.26) | 1090.53(1086.92,1094.15) | -2.66(-3.48,-1.84) |
| Denmark | both | 6578.96(4517.10,9103.66) | 5066.11(3534.65,6801.30) | -23.00 | 734.34(715.67,753.41) | 522.86(507.80,538.29) | -0.61(-0.86,-0.37) |
| Djibouti | both | 11093.25(8469.22,14139.30) | 16279.88(11521.91,21200.50) | 46.75 | 5310.39(5196.66,5426.09) | 3436.94(3375.85,3498.90) | -1.52(-1.99,-1.04) |
| Dominica | both | 196.58(143.16,257.62) | 61.75(45.19,80.35) | -68.59 | 754.75(642.18,882.26) | 525.24(387.80,698.07) | -0.67(-0.96,-0.39) |
| Dominican Republic | both | 23096.13(17597.60,29331.40) | 12640.23(9451.08,16135.77) | -45.27 | 771.99(761.15,782.95) | 398.76(391.09,406.55) | -1.44(-1.68,-1.20) |
| Ecuador | both | 28883.35(20370.70,38278.04) | 21082.88(15648.88,27202.08) | -27.01 | 636.81(628.42,645.30) | 392.26(386.33,398.26) | -1.65(-2.36,-0.94) |
| Egypt | both | 435501.97(333344.72,545716.88) | 519030.92(382087.41,685091.69) | 19.18 | 1763.59(1757.76,1769.43) | 1302.25(1298.32,1306.19) | -0.56(-0.93,-0.20) |
| El Salvador | both | 21087.92(16630.84,25923.53) | 5959.25(4454.14,7707.63) | -71.74 | 859.13(846.19,872.23) | 314.55(305.71,323.59) | -2.15(-2.61,-1.67) |
| Equatorial Guinea | both | 7189.75(4839.60,9786.76) | 2270.21(1529.10,3187.87) | -68.42 | 2688.27(2616.60,2761.51) | 398.50(380.35,417.31) | -6.51(-6.83,-6.20) |
| Eritrea | both | 111301.12(82630.65,141128.07) | 77428.26(56831.20,99343.74) | -30.43 | 5320.59(5284.30,5357.08) | 2283.78(2264.25,2303.44) | -2.89(-3.09,-2.69) |
| Estonia | both | 3330.82(2336.51,4597.07) | 1460.27(1035.87,1957.05) | -56.16 | 967.94(932.85,1004.05) | 691.70(654.45,730.64) | -1.20(-1.37,-1.04) |
| Eswatini | both | 3387.11(2470.72,4395.15) | 2054.47(1505.29,2634.61) | -39.34 | 686.29(660.06,713.35) | 437.01(415.93,458.95) | -1.46(-1.68,-1.23) |
| Ethiopia | both | 1349263.84(960227.11,1785784.69) | 737597.89(495022.77,1013901.14) | -45.33 | 4281.78(4273.36,4290.22) | 1459.55(1455.70,1463.40) | -3.16(-3.49,-2.83) |
| Fiji | both | 6604.11(4716.88,8568.33) | 3672.92(2615.49,4772.83) | -44.38 | 2227.00(2166.73,2288.57) | 1193.19(1149.34,1238.36) | -2.11(-2.30,-1.92) |
| Finland | both | 8272.06(5630.96,11435.33) | 5655.03(3888.62,7798.65) | -31.64 | 855.88(836.65,875.48) | 670.27(651.90,689.08) | -0.35(-0.52,-0.18) |
| France | both | 73551.55(51655.18,98554.31) | 63552.16(47133.22,87834.96) | -13.60 | 629.79(625.01,634.59) | 535.10(530.75,539.48) | 0.07(-0.18,0.33) |
| Gabon | both | 6685.49(4310.25,9252.49) | 3008.64(2117.93,4011.38) | -55.00 | 1312.57(1277.39,1348.53) | 466.08(447.77,484.97) | -2.48(-2.88,-2.08) |
| Gambia | both | 17279.71(11602.61,23554.30) | 16573.65(11905.72,21660.50) | -4.09 | 2670.19(2623.82,2717.23) | 1338.72(1314.45,1363.34) | -1.25(-1.72,-0.77) |
| Georgia | both | 9330.20(6312.27,12606.18) | 2978.17(2016.32,4165.79) | -68.08 | 687.84(672.85,703.09) | 392.02(377.06,407.46) | -2.34(-2.73,-1.95) |
| Germany | both | 82596.82(56067.69,115177.40) | 60855.50(38737.96,83959.34) | -26.32 | 622.11(617.65,626.60) | 496.03(491.96,500.14) | -0.32(-0.66,0.02) |
| Ghana | both | 233214.79(186782.71,284854.36) | 176806.68(124901.90,231118.52) | -24.19 | 2481.01(2469.17,2492.89) | 1071.86(1065.91,1077.85) | -2.18(-2.72,-1.64) |
| Greece | both | 10594.25(7068.01,14996.31) | 11114.16(7154.05,17544.41) | 4.91 | 539.63(528.53,550.92) | 774.86(759.88,790.09) | 1.62(1.39,1.85) |
| Greenland | both | 83.88(59.68,112.94) | 37.44(26.82,51.41) | -55.37 | 534.67(417.81,675.65) | 302.83(207.89,428.93) | -1.20(-1.61,-0.79) |
| Grenada | both | 330.80(241.35,429.29) | 126.24(96.05,164.18) | -61.84 | 922.10(814.87,1039.98) | 587.81(478.43,715.71) | -0.94(-1.26,-0.61) |
| Guam | both | 264.53(200.98,348.39) | 186.40(139.02,237.01) | -29.54 | 570.68(497.58,651.98) | 458.70(388.40,538.79) | -0.19(-0.58,0.20) |
| Guatemala | both | 77641.49(67958.23,87466.38) | 14644.54(11507.89,18073.97) | -81.14 | 1600.03(1587.23,1612.90) | 271.24(266.29,276.26) | -5.93(-6.22,-5.63) |
| Guinea | both | 117676.73(77416.30,159152.75) | 125708.61(91564.49,165978.82) | 6.83 | 2791.49(2772.64,2810.46) | 1568.14(1557.79,1578.55) | -1.25(-1.57,-0.94) |
| Guinea-Bissau | both | 20190.88(14506.32,26348.68) | 11623.68(8021.17,15512.41) | -42.43 | 3151.65(3101.80,3202.14) | 1066.68(1043.97,1089.78) | -3.17(-3.56,-2.78) |
| Guyana | both | 8936.61(6928.68,11087.29) | 2389.20(1788.38,3041.77) | -73.27 | 2679.44(2616.08,2743.98) | 1055.38(1007.91,1104.56) | -2.66(-3.04,-2.28) |
| Haiti | both | 64678.93(48849.84,80609.20) | 52331.79(34172.57,70059.31) | -19.09 | 1848.73(1832.06,1865.52) | 1006.90(996.84,1017.03) | -2.12(-2.63,-1.61) |
| Honduras | both | 21939.69(18428.24,25911.30) | 11776.11(8791.45,14966.89) | -46.33 | 885.52(872.47,898.74) | 336.75(329.91,343.69) | -2.94(-3.24,-2.65) |
| Hungary | both | 14439.95(9354.05,20184.15) | 7518.31(5138.03,10134.62) | -47.93 | 728.55(715.77,741.52) | 545.86(532.75,559.23) | -0.46(-0.60,-0.31) |
| Iceland | both | 411.09(278.27,552.74) | 403.10(275.67,554.90) | -1.94 | 634.26(571.35,702.87) | 583.60(525.34,647.19) | 0.12(-0.12,0.35) |
| India | both | 21601303.48(15091151.25,28582732.76) | 11850337.82(8910515.77,15473055.78) | -45.14 | 5929.37(5926.53,5932.21) | 3503.66(3501.46,3505.86) | -1.11(-1.69,-0.52) |
| Indonesia | both | 2849056.46(1930462.50,3843028.50) | 1183052.65(835491.59,1597928.22) | -58.48 | 4004.62(3999.34,4009.90) | 1734.34(1730.83,1737.86) | -2.16(-2.74,-1.57) |
| Iran (Islamic Republic of) | both | 546817.64(393536.73,724680.86) | 249078.50(191215.22,319069.15) | -54.45 | 2140.71(2134.48,2146.95) | 1290.23(1284.70,1295.78) | -1.25(-1.49,-1.01) |
| Iraq | both | 248221.51(188906.66,316695.98) | 185262.92(141951.39,236582.46) | -25.36 | 2648.89(2637.17,2660.66) | 1388.49(1381.52,1395.50) | -2.44(-2.95,-1.92) |
| Ireland | both | 6841.61(4627.01,9441.12) | 6090.68(3919.62,8870.97) | -10.98 | 726.30(708.19,744.79) | 602.93(587.20,619.01) | -0.18(-0.40,0.04) |
| Israel | both | 11031.55(7433.28,15554.48) | 14166.57(9503.25,19742.86) | 28.42 | 717.81(703.86,731.98) | 530.03(520.99,539.20) | -0.46(-0.74,-0.17) |
| Italy | both | 106339.32(75008.63,142425.83) | 54696.64(37512.78,75347.44) | -48.56 | 1166.49(1159.14,1173.88) | 716.30(709.99,722.65) | -0.64(-1.05,-0.22) |
| Jamaica | both | 10132.52(8041.22,12118.97) | 2937.26(2181.41,3711.03) | -71.01 | 1161.68(1136.48,1187.33) | 543.80(521.88,566.44) | -1.98(-2.23,-1.72) |
| Japan | both | 350831.71(256127.78,457111.11) | 156394.88(114787.33,203384.69) | -55.42 | 1584.26(1578.59,1589.95) | 1046.71(1041.09,1052.35) | -0.87(-1.14,-0.59) |
| Jordan | both | 18780.68(14537.96,23595.86) | 25489.04(18900.97,33182.02) | 35.72 | 1043.22(1026.65,1060.00) | 729.93(720.15,739.82) | -0.78(-1.13,-0.43) |
| Kazakhstan | both | 51898.49(33736.24,72382.56) | 48283.95(30750.03,68068.45) | -6.96 | 934.96(926.30,943.68) | 822.62(814.71,830.60) | -0.02(-0.66,0.63) |
| Kenya | both | 205261.22(137778.64,277460.30) | 142598.37(96510.79,192274.97) | -30.53 | 1568.59(1560.86,1576.35) | 712.08(707.85,716.34) | -2.39(-2.92,-1.86) |
| Kiribati | both | 1223.31(939.04,1546.39) | 533.48(393.92,694.28) | -56.39 | 3219.90(3019.37,3431.06) | 1103.03(1000.57,1213.85) | -3.72(-3.86,-3.59) |
| Kuwait | both | 8123.74(6220.76,10388.61) | 6102.05(4485.32,8057.52) | -24.89 | 1379.17(1346.38,1412.58) | 742.15(722.15,762.61) | -2.47(-2.99,-1.95) |
| Kyrgyzstan | both | 23455.09(16628.81,31108.70) | 18780.44(13742.08,24554.11) | -19.93 | 1271.90(1254.04,1289.95) | 805.54(793.13,818.11) | -2.21(-2.62,-1.79) |
| Lao People's Democratic Republic | both | 69515.88(46938.36,93229.73) | 24581.62(16719.68,33153.27) | -64.64 | 3041.10(3015.08,3067.30) | 990.91(976.83,1005.15) | -3.31(-3.88,-2.74) |
| Latvia | both | 5830.13(4048.31,7792.56) | 2203.85(1603.23,2890.42) | -62.20 | 1016.22(988.33,1044.72) | 767.07(733.32,802.06) | -0.97(-1.20,-0.74) |
| Lebanon | both | 26037.23(20060.01,32517.72) | 18152.94(14243.73,22730.25) | -30.28 | 2312.65(2281.82,2343.80) | 1475.20(1452.13,1498.55) | -0.84(-1.24,-0.43) |
| Lesotho | both | 19194.32(14273.07,24453.99) | 5793.97(4412.99,7628.71) | -69.81 | 2580.62(2540.76,2620.97) | 845.57(820.98,870.75) | -3.94(-4.26,-3.63) |
| Liberia | both | 38609.35(27278.20,50524.28) | 22899.01(16804.68,29881.82) | -40.69 | 2407.85(2380.35,2435.61) | 858.31(845.22,871.56) | -3.34(-3.64,-3.03) |
| Libya | both | 35369.13(26436.70,45173.52) | 21370.73(16859.53,26578.58) | -39.58 | 1817.96(1796.89,1839.22) | 1582.43(1558.80,1606.35) | 0.39(0.04,0.73) |
| Lithuania | both | 8950.98(6367.41,11907.80) | 2775.68(2018.52,3580.67) | -68.99 | 1067.74(1043.91,1091.99) | 697.00(669.72,725.16) | -1.45(-1.58,-1.32) |
| Luxembourg | both | 448.57(314.41,609.97) | 562.06(408.86,764.05) | 25.30 | 658.58(595.62,726.89) | 548.74(502.08,598.92) | -0.27(-0.50,-0.04) |
| Madagascar | both | 189832.63(159196.66,220053.80) | 330123.20(260230.74,405174.71) | 73.90 | 2697.83(2683.76,2711.96) | 2310.79(2301.37,2320.25) | -0.91(-1.25,-0.57) |
| Malawi | both | 122742.80(97119.36,149053.49) | 83844.45(64430.54,107912.45) | -31.69 | 1818.74(1806.90,1830.64) | 877.31(870.30,884.36) | -2.87(-3.27,-2.48) |
| Malaysia | both | 266342.61(214812.62,324134.56) | 173474.49(125651.24,224211.87) | -34.87 | 3628.27(3612.74,3643.85) | 2242.50(2230.66,2254.40) | -1.42(-1.67,-1.17) |
| Maldives | both | 5887.68(4234.30,7527.27) | 1722.37(1241.02,2189.66) | -70.75 | 4774.68(4638.89,4913.54) | 1881.94(1786.37,1981.43) | -2.78(-3.08,-2.47) |
| Mali | both | 265266.35(181363.15,360422.12) | 404005.34(306452.79,509301.04) | 52.30 | 4170.30(4151.52,4189.15) | 2492.64(2483.73,2501.58) | -1.67(-1.92,-1.41) |
| Malta | both | 754.02(523.36,1037.88) | 410.57(276.32,575.13) | -45.55 | 873.94(809.51,942.50) | 633.50(571.12,701.29) | -0.63(-0.85,-0.42) |
| Marshall Islands | both | 37.49(28.46,48.78) | 27.95(21.98,35.33) | -25.46 | 169.17(116.71,238.66) | 147.57(96.16,220.24) | 0.01(-0.25,0.26) |
| Mauritania | both | 58902.65(47579.25,71830.06) | 41150.66(29921.01,54552.12) | -30.14 | 4875.90(4830.32,4921.82) | 1998.21(1976.16,2020.46) | -2.04(-2.48,-1.60) |
| Mauritius | both | 11047.23(8194.82,14220.10) | 3865.97(2824.99,4946.26) | -65.01 | 3385.69(3314.48,3458.10) | 1912.42(1845.05,1981.71) | -2.23(-2.54,-1.91) |
| Mexico | both | 473845.82(335433.16,619703.45) | 210631.22(163806.53,260155.23) | -55.55 | 1354.38(1350.16,1358.60) | 692.00(688.82,695.19) | -1.11(-1.58,-0.63) |
| Micronesia (Federated States of) | both | 668.12(483.83,854.43) | 298.38(216.26,389.18) | -55.34 | 1334.88(1223.12,1454.52) | 929.21(810.97,1060.57) | -0.87(-1.23,-0.51) |
| Monaco | both | 21.52(14.69,31.10) | 28.65(18.22,41.70) | 33.12 | 591.94(360.32,935.03) | 564.90(371.35,834.08) | 0.29(0.01,0.57) |
| Mongolia | both | 10516.95(6750.78,14540.56) | 5798.87(4188.77,7699.66) | -44.86 | 1013.12(991.65,1034.95) | 506.24(492.36,520.43) | -3.53(-3.88,-3.18) |
| Montenegro | both | 1116.92(761.90,1532.76) | 918.12(656.05,1198.41) | -17.80 | 714.93(670.56,761.60) | 836.24(779.04,896.76) | 0.60(-0.05,1.26) |
| Morocco | both | 134745.76(103397.38,167851.09) | 71379.52(52124.10,94895.52) | -47.03 | 1253.19(1245.66,1260.75) | 724.71(718.89,730.57) | -1.88(-2.84,-0.92) |
| Mozambique | both | 209351.65(151941.85,269517.51) | 141529.54(102142.54,184610.47) | -32.40 | 2461.27(2448.92,2473.67) | 785.32(780.49,790.18) | -3.87(-4.01,-3.74) |
| Myanmar | both | 500678.87(339657.79,677226.64) | 161368.26(108335.10,217819.00) | -67.77 | 3077.52(3067.72,3087.34) | 989.44(983.93,994.98) | -2.96(-3.58,-2.34) |
| Namibia | both | 21209.76(17517.16,25171.17) | 12302.37(8840.26,16172.75) | -42.00 | 2755.82(2712.48,2799.71) | 1312.42(1285.75,1339.53) | -1.88(-2.25,-1.51) |
| Nauru | both | 35.47(25.42,45.98) | 23.67(18.44,30.51) | -33.25 | 682.42(458.25,986.25) | 540.10(332.75,839.77) | -0.65(-1.02,-0.28) |
| Nepal | both | 405904.84(292033.09,524074.81) | 136832.51(101992.38,179071.71) | -66.29 | 3564.57(3551.58,3577.59) | 1402.77(1394.28,1411.31) | -2.48(-2.90,-2.06) |
| Netherlands | both | 20607.75(13238.62,31761.03) | 17667.18(11410.66,25791.31) | -14.27 | 738.35(728.01,748.81) | 643.24(633.54,653.07) | 0.24(-0.07,0.54) |
| New Zealand | both | 6153.78(4529.93,8300.10) | 4658.49(3340.00,6345.73) | -24.30 | 728.12(708.85,747.81) | 469.97(455.77,484.54) | -0.79(-1.18,-0.40) |
| Nicaragua | both | 17311.39(13177.85,21647.45) | 6364.49(4804.69,8191.24) | -63.24 | 844.37(830.54,858.38) | 306.79(298.45,315.30) | -2.41(-2.78,-2.03) |
| Niger | both | 308071.12(256146.62,360594.80) | 430678.47(289282.03,567972.29) | 39.80 | 4839.27(4818.86,4859.75) | 2306.67(2298.27,2315.10) | -1.48(-2.05,-0.90) |
| Nigeria | both | 1703918.82(1164672.86,2308859.72) | 1961612.82(1298615.49,2727909.13) | 15.12 | 3388.43(3382.52,3394.35) | 1672.12(1669.42,1674.82) | -1.56(-2.11,-1.00) |
| Niue | both | 6.55(5.02,8.19) | 2.91(2.25,3.71) | -55.61 | 803.67(274.78,1870.75) | 731.51(109.98,2610.78) | -0.30(-0.65,0.05) |
| North Macedonia | both | 6960.03(4922.93,9341.75) | 3593.41(2550.22,4727.08) | -48.37 | 1285.19(1252.36,1318.72) | 1065.87(1027.72,1105.17) | -0.17(-0.44,0.10) |
| Northern Mariana Islands | both | 90.14(68.14,115.01) | 72.22(57.86,87.44) | -19.88 | 632.62(496.05,797.85) | 637.16(483.69,827.69) | 0.38(0.05,0.71) |
| Norway | both | 7910.97(5638.69,10589.43) | 6571.88(4524.24,9102.73) | -16.93 | 962.63(940.65,985.03) | 702.25(684.61,720.27) | -0.26(-0.65,0.13) |
| Oman | both | 29355.46(22391.52,37410.94) | 26121.73(20177.42,33268.28) | -11.02 | 3117.21(3077.29,3157.54) | 2094.62(2067.06,2122.47) | -1.03(-1.20,-0.86) |
| Pakistan | both | 2070847.19(1360789.66,2781027.18) | 2148261.77(1477392.72,2878890.40) | 3.74 | 3560.09(3554.52,3565.67) | 2274.99(2271.50,2278.49) | -0.38(-0.87,0.12) |
| Palau | both | 36.81(27.71,45.95) | 22.01(17.26,27.37) | -40.21 | 765.89(518.17,1100.04) | 669.65(391.28,1083.16) | 0.02(-0.37,0.41) |
| Palestine | both | 17103.76(12369.45,22544.59) | 12334.67(9087.72,16196.88) | -27.88 | 1478.18(1453.27,1503.43) | 645.80(633.31,658.48) | -3.21(-3.71,-2.70) |
| Panama | both | 6228.32(4889.48,7697.29) | 3297.40(2608.11,4086.49) | -47.06 | 701.34(682.17,720.94) | 261.68(251.71,271.95) | -2.64(-2.94,-2.33) |
| Papua New Guinea | both | 56644.30(40990.12,72949.85) | 138794.78(101061.34,176245.62) | 145.03 | 2682.85(2657.34,2708.56) | 2781.28(2764.46,2798.18) | 0.51(0.03,0.99) |
| Paraguay | both | 4612.35(3218.36,6541.06) | 5597.97(4127.83,7393.61) | 21.37 | 271.35(263.26,279.63) | 268.36(260.82,276.07) | 0.34(-0.52,1.21) |
| Peru | both | 54712.00(44735.35,65968.97) | 19175.97(13926.44,24624.60) | -64.95 | 558.11(552.76,563.49) | 182.50(179.69,185.34) | -3.51(-3.68,-3.35) |
| Philippines | both | 716166.17(523748.71,904231.41) | 503449.05(368624.94,644973.63) | -29.70 | 2510.43(2503.90,2516.98) | 1379.52(1375.13,1383.92) | -1.42(-1.71,-1.12) |
| Poland | both | 110177.53(82969.96,145661.92) | 50026.38(37468.78,65155.33) | -54.59 | 1195.34(1187.73,1202.98) | 866.30(858.21,874.45) | -0.43(-0.81,-0.05) |
| Portugal | both | 12821.21(9049.21,17727.85) | 7008.74(4916.39,9292.89) | -45.33 | 638.62(626.75,650.67) | 510.92(498.38,523.72) | -0.12(-0.44,0.19) |
| Puerto Rico | both | 7484.10(5723.04,9472.55) | 2106.28(1664.01,2682.80) | -71.86 | 734.32(716.19,752.82) | 523.26(498.16,549.40) | -0.81(-1.05,-0.58) |
| Qatar | both | 2008.87(1553.86,2601.40) | 8372.22(6210.67,10934.95) | 316.76 | 1398.02(1332.00,1466.59) | 1668.69(1631.91,1706.15) | 1.51(1.09,1.94) |
| Republic of Korea | both | 94031.94(68303.60,122745.93) | 31445.48(23026.53,41624.26) | -66.56 | 814.24(808.37,820.14) | 529.74(523.24,536.31) | -0.90(-1.06,-0.74) |
| Republic of Moldova | both | 14502.79(10643.98,18923.54) | 3996.36(2920.09,5147.65) | -72.44 | 1146.94(1126.58,1167.58) | 796.82(770.08,824.32) | -1.49(-1.87,-1.11) |
| Romania | both | 64002.82(50273.30,79910.85) | 19588.86(13798.98,26185.64) | -69.39 | 1195.95(1185.75,1206.22) | 677.58(667.32,687.96) | -1.48(-1.65,-1.31) |
| Russian Federation | both | 561607.34(422632.17,716763.60) | 256824.95(190495.23,330209.11) | -54.27 | 1630.45(1625.80,1635.11) | 1013.52(1009.17,1017.88) | -1.33(-1.53,-1.13) |
| Rwanda | both | 66689.05(48286.90,84150.00) | 30747.83(21798.02,40293.00) | -53.89 | 1405.12(1392.57,1417.76) | 496.68(490.19,503.24) | -4.79(-5.63,-3.94) |
| Saint Kitts and Nevis | both | 136.73(106.70,166.17) | 57.77(45.82,72.74) | -57.75 | 929.21(764.62,1120.47) | 580.12(424.90,776.44) | -1.02(-1.33,-0.72) |
| Saint Lucia | both | 584.69(455.12,718.68) | 219.59(168.97,276.12) | -62.44 | 1119.22(1021.56,1224.01) | 782.50(671.26,907.69) | -0.64(-0.96,-0.33) |
| Saint Vincent and the Grenadines | both | 391.72(303.71,495.15) | 121.36(89.55,156.95) | -69.02 | 953.73(851.82,1065.12) | 513.01(415.75,627.19) | -1.62(-1.85,-1.39) |
| Samoa | both | 1472.22(993.25,2008.42) | 907.55(599.37,1274.75) | -38.36 | 1762.34(1662.32,1867.15) | 936.59(867.89,1009.55) | -1.71(-1.97,-1.45) |
| San Marino | both | 25.61(17.80,37.14) | 25.26(16.03,37.17) | -1.37 | 634.69(401.67,970.90) | 572.34(359.81,882.30) | 0.18(-0.03,0.39) |
| Sao Tome and Principe | both | 1142.80(812.19,1481.20) | 542.97(353.61,747.24) | -52.49 | 1686.46(1575.92,1803.12) | 642.35(580.49,709.23) | -2.45(-3.31,-1.58) |
| Saudi Arabia | both | 246056.82(182931.91,321259.48) | 144233.56(108546.63,181956.61) | -41.38 | 3585.51(3569.97,3601.10) | 1975.32(1964.33,1986.35) | -1.38(-1.75,-1.01) |
| Senegal | both | 125627.76(96125.33,160566.32) | 77105.84(53986.86,104179.86) | -38.62 | 2495.78(2479.76,2511.87) | 1080.88(1072.06,1089.76) | -1.99(-2.62,-1.35) |
| Serbia | both | 22046.73(15880.56,29116.05) | 11018.65(7888.84,14735.55) | -50.02 | 1058.16(1043.05,1073.45) | 936.32(917.34,955.62) | -0.15(-0.41,0.10) |
| Seychelles | both | 233.08(181.86,291.89) | 214.18(160.51,279.18) | -8.11 | 938.29(808.42,1083.94) | 881.79(753.61,1026.24) | 0.57(0.08,1.07) |
| Sierra Leone | both | 74799.19(61150.23,88836.09) | 62004.12(42555.58,84587.09) | -17.11 | 2755.78(2732.22,2779.50) | 1256.43(1244.59,1268.35) | -2.62(-3.23,-2.00) |
| Singapore | both | 14308.30(10526.19,18567.19) | 13019.17(9305.03,17277.44) | -9.01 | 2185.15(2145.40,2225.51) | 1564.25(1535.72,1593.20) | -1.24(-1.33,-1.16) |
| Slovakia | both | 11699.07(8112.59,15586.09) | 7303.47(5171.17,9699.03) | -37.57 | 912.59(894.67,930.81) | 851.10(830.21,872.41) | 0.04(-0.35,0.43) |
| Slovenia | both | 3240.11(2354.89,4280.00) | 2426.32(1660.57,3354.73) | -25.12 | 824.81(794.25,856.31) | 791.44(758.13,825.92) | 0.15(-0.06,0.35) |
| Solomon Islands | both | 3830.98(2962.77,4764.25) | 4731.29(3112.30,6238.84) | 23.50 | 1983.78(1912.75,2056.91) | 1533.24(1483.22,1584.59) | -0.09(-0.44,0.27) |
| Somalia | both | 212848.73(152977.78,275514.63) | 374595.81(279542.61,477444.99) | 75.99 | 4382.71(4361.19,4404.32) | 2775.84(2765.43,2786.29) | -2.37(-2.67,-2.07) |
| South Africa | both | 260818.56(176041.85,354033.91) | 95286.13(68360.33,127355.64) | -63.47 | 1728.52(1721.12,1735.95) | 620.16(615.78,624.56) | -3.13(-3.40,-2.85) |
| South Sudan | both | 164022.37(109810.92,218629.49) | 183561.09(130537.23,246908.48) | 11.91 | 4985.95(4957.80,5014.22) | 3581.20(3561.81,3600.68) | -1.21(-1.85,-0.56) |
| Spain | both | 60510.40(41387.57,85514.07) | 45871.70(32543.47,61966.13) | -24.19 | 785.11(778.31,791.96) | 716.10(709.07,723.19) | 0.14(-0.20,0.49) |
| Sri Lanka | both | 267091.27(220478.70,315816.55) | 161811.16(121300.72,205087.67) | -39.42 | 4851.70(4831.03,4872.45) | 3350.99(3332.65,3369.40) | -0.96(-1.29,-0.63) |
| Sudan | both | 597906.30(520513.32,684092.91) | 477533.77(335429.73,622020.16) | -20.13 | 5167.89(5152.26,5183.56) | 2654.64(2646.00,2663.30) | -1.69(-2.22,-1.16) |
| Suriname | both | 1984.30(1375.82,2632.51) | 1045.88(711.64,1443.68) | -47.29 | 1499.03(1426.02,1574.91) | 781.78(729.59,836.82) | -1.42(-1.85,-0.97) |
| Sweden | both | 14564.90(10129.19,19708.47) | 12533.35(8643.74,17198.07) | -13.95 | 902.32(887.09,917.76) | 681.79(669.40,694.37) | -0.25(-0.71,0.21) |
| Switzerland | both | 7367.05(4839.98,10373.45) | 7123.71(5029.01,10032.35) | -3.30 | 627.13(612.24,642.33) | 526.70(514.02,539.63) | -0.17(-0.40,0.06) |
| Syrian Arab Republic | both | 195613.21(136096.41,257984.78) | 95907.66(74565.57,123709.60) | -50.97 | 3068.04(3052.87,3083.26) | 3000.40(2979.12,3021.81) | 0.18(-0.09,0.44) |
| Taiwan (Province of China) | both | 64455.01(50210.08,81833.11) | 35527.46(26424.77,46830.36) | -44.88 | 1208.02(1197.55,1218.56) | 1168.28(1155.46,1181.22) | 1.04(0.57,1.51) |
| Tajikistan | both | 63792.65(44365.58,84893.61) | 76423.91(54957.79,101085.61) | 19.80 | 2238.59(2218.85,2258.48) | 1773.28(1758.93,1787.73) | -1.60(-2.02,-1.18) |
| Thailand | both | 365671.02(291353.14,447160.68) | 141192.09(104912.18,180317.01) | -61.39 | 2213.73(2205.71,2221.78) | 1570.58(1561.47,1579.73) | -1.10(-1.38,-0.83) |
| Timor-Leste | both | 26958.36(18867.38,34658.72) | 17507.61(12448.01,23551.10) | -35.06 | 6316.44(6229.86,6403.99) | 3105.57(3052.58,3159.27) | -1.58(-2.05,-1.10) |
| Togo | both | 52651.95(41112.64,65257.98) | 42776.92(29749.62,56065.44) | -18.76 | 2217.07(2194.83,2239.49) | 1039.27(1027.64,1050.99) | -2.48(-3.08,-1.87) |
| Tokelau | both | 6.77(5.01,8.46) | 2.80(2.22,3.44) | -58.63 | 1079.61(365.59,2522.62) | 799.96(106.10,2950.62) | -0.92(-1.28,-0.55) |
| Tonga | both | 253.17(192.66,324.93) | 468.19(351.48,605.40) | 84.93 | 561.50(488.84,642.46) | 1061.90(955.66,1177.09) | 3.19(2.37,4.01) |
| Trinidad and Tobago | both | 4645.46(3549.49,6033.86) | 2821.25(2186.23,3609.01) | -39.27 | 1258.32(1219.95,1297.61) | 1181.27(1133.52,1230.56) | 0.24(-0.18,0.66) |
| Tunisia | both | 44954.49(32886.04,57905.78) | 23351.12(17262.98,30313.78) | -48.06 | 1450.90(1436.19,1465.72) | 848.09(836.33,859.99) | -2.39(-2.78,-2.00) |
| Türkiye | both | 205863.86(140380.39,285914.06) | 77402.55(56300.55,102828.57) | -62.40 | 922.97(918.52,927.45) | 406.14(403.02,409.28) | -2.73(-3.08,-2.38) |
| Turkmenistan | both | 34017.31(22557.45,47121.49) | 21094.27(15023.00,28662.61) | -37.99 | 1925.50(1902.51,1948.71) | 1302.48(1283.34,1321.84) | -1.50(-1.85,-1.16) |
| Tuvalu | both | 75.55(54.39,95.91) | 25.58(19.23,32.05) | -66.14 | 1627.90(1239.01,2111.12) | 652.84(401.49,1009.66) | -2.20(-2.48,-1.91) |
| Uganda | both | 145357.24(108146.93,186892.49) | 197898.96(131159.85,266289.71) | 36.15 | 1101.03(1094.18,1107.92) | 737.55(733.69,741.41) | -2.17(-2.60,-1.73) |
| Ukraine | both | 290705.25(217973.80,378008.22) | 139615.80(106035.96,178020.30) | -51.97 | 2479.93(2469.92,2489.97) | 2527.00(2511.99,2542.08) | 0.91(0.53,1.28) |
| United Arab Emirates | both | 38179.05(29785.03,47582.66) | 79094.60(62729.82,98392.16) | 107.17 | 5987.92(5922.16,6054.25) | 5795.02(5752.96,5837.33) | 0.46(0.13,0.79) |
| United Kingdom | both | 114459.35(80113.90,152835.85) | 77472.43(54369.29,103599.05) | -32.31 | 1023.93(1017.77,1030.13) | 660.84(655.97,665.75) | -0.51(-0.95,-0.08) |
| United Republic of Tanzania | both | 309370.65(253960.29,366943.11) | 251421.83(175233.80,330375.77) | -18.73 | 1882.97(1875.25,1890.72) | 843.13(839.36,846.92) | -2.79(-2.99,-2.59) |
| United States of America | both | 389387.37(265663.15,552471.55) | 294718.64(208798.02,399717.57) | -24.31 | 675.48(673.28,677.68) | 469.56(467.79,471.33) | -0.47(-1.03,0.10) |
| United States Virgin Islands | both | 233.02(173.21,298.39) | 67.42(52.13,84.43) | -71.07 | 699.63(604.26,806.53) | 525.97(395.48,688.00) | -0.43(-0.66,-0.20) |
| Uruguay | both | 6200.93(4440.18,8015.57) | 2823.12(2147.59,3607.75) | -54.47 | 817.11(795.43,839.25) | 492.10(472.82,511.99) | -1.47(-1.88,-1.06) |
| Uzbekistan | both | 252928.87(178277.08,336765.49) | 132462.09(97176.84,175210.41) | -47.63 | 2330.78(2320.42,2341.18) | 1187.67(1180.72,1194.66) | -2.47(-2.70,-2.23) |
| Vanuatu | both | 1584.03(1151.03,2047.28) | 1352.82(985.48,1717.32) | -14.60 | 1864.21(1760.36,1972.84) | 992.61(932.68,1055.52) | -1.83(-2.09,-1.57) |
| Venezuela (Bolivarian Republic of) | both | 112895.87(95893.55,131587.55) | 47004.74(34472.87,62005.26) | -58.36 | 1413.92(1404.47,1423.43) | 722.75(715.43,730.12) | -1.99(-2.25,-1.73) |
| Viet Nam | both | 1002000.65(806362.16,1221083.40) | 440792.80(334144.32,553946.08) | -56.01 | 3462.62(3454.99,3470.27) | 1760.32(1754.56,1766.09) | -2.12(-2.47,-1.77) |
| Yemen | both | 361207.82(285385.21,453571.67) | 391389.53(264941.19,532787.67) | 8.36 | 4384.64(4368.48,4400.85) | 2722.68(2713.00,2732.39) | -1.09(-1.52,-0.65) |
| Zambia | both | 87827.31(69759.56,106727.55) | 86358.59(66207.14,107150.67) | -1.67 | 1603.28(1590.70,1615.95) | 889.91(883.07,896.79) | -1.47(-2.01,-0.93) |
| Zimbabwe | both | 61591.75(47249.36,79844.03) | 84331.76(69479.10,103315.68) | 36.92 | 1033.49(1024.07,1042.99) | 1063.12(1054.71,1071.58) | -0.20(-0.57,0.18) |
| Global | female | 25906697.72(18849116.21,33653386.97) | 14852422.61(11056914.81,19266417.53) | -42.67 | 2734.63(2733.44,2735.82) | 1502.66(1501.81,1503.51) | -1.43(-1.92,-0.93) |
| Central Europe, eastern Europe, and central Asia | female | 827564.46(614871.10,1058993.10) | 399176.69(296067.05,521648.44) | -51.76 | 1547.29(1543.62,1550.97) | 1016.99(1013.53,1020.45) | -1.11(-1.30,-0.91) |
| High-income | female | 725351.24(509668.77,964780.46) | 456973.94(333367.84,602112.61) | -37.00 | 782.03(780.14,783.94) | 526.07(524.46,527.67) | -0.65(-0.98,-0.31) |
| Latin America and Caribbean | female | 746520.17(559619.90,959405.02) | 347572.00(268011.70,442486.89) | -53.44 | 976.82(974.36,979.28) | 482.80(481.04,484.56) | -1.33(-1.77,-0.90) |
| Southeast Asia, east Asia, and Oceania | female | 5763734.99(4273667.79,7399568.49) | 2555553.81(1937281.42,3309250.29) | -55.66 | 2223.82(2221.79,2225.86) | 1226.98(1225.33,1228.63) | -1.45(-1.86,-1.04) |
| Sub-Saharan Africa | female | 3928871.12(2902698.72,5076081.67) | 3612575.44(2566210.73,4765549.21) | -8.05 | 2668.58(2665.51,2671.66) | 1285.59(1284.05,1287.14) | -2.13(-2.53,-1.73) |
| Andean Latin America | female | 50545.55(39503.50,62788.33) | 25290.51(19176.37,31949.76) | -49.96 | 586.22(580.44,592.06) | 259.92(256.41,263.48) | -2.60(-2.99,-2.21) |
| Australasia | female | 12038.17(8610.71,16390.94) | 10738.96(7835.37,14380.70) | -10.79 | 528.31(518.33,538.44) | 387.93(380.24,395.75) | -0.56(-0.83,-0.29) |
| Caribbean | female | 67978.77(52605.70,83607.42) | 41486.10(29397.16,55110.79) | -38.97 | 1064.88(1055.85,1073.96) | 694.95(687.41,702.55) | -1.37(-1.67,-1.07) |
| Central Asia | female | 275117.13(194886.43,356154.85) | 152632.69(111540.97,204471.57) | -44.52 | 1898.86(1890.95,1906.80) | 1055.08(1049.29,1060.90) | -2.17(-2.38,-1.97) |
| Central Europe | female | 154180.24(116308.58,198092.21) | 72949.78(53682.03,94372.50) | -52.69 | 1101.94(1095.94,1107.96) | 856.66(849.99,863.37) | -0.41(-0.65,-0.18) |
| Central Latin America | female | 396724.15(298592.73,500393.37) | 158411.00(123653.46,197608.10) | -60.07 | 1127.26(1123.36,1131.18) | 514.64(511.88,517.42) | -1.54(-1.97,-1.10) |
| Central Sub-Saharan Africa | female | 410551.06(276064.28,550920.53) | 272168.24(179966.85,378005.81) | -33.71 | 2416.48(2407.79,2425.20) | 811.26(807.70,814.83) | -3.45(-4.11,-2.79) |
| East Asia | female | 2594119.91(1903038.99,3344323.13) | 1122374.45(835205.77,1513044.09) | -56.73 | 1560.48(1558.38,1562.59) | 909.51(907.72,911.30) | -0.94(-1.28,-0.61) |
| Eastern Europe | female | 398267.09(295112.19,514087.78) | 173594.22(128266.93,227823.09) | -56.41 | 1564.49(1559.15,1569.84) | 1050.85(1045.34,1056.38) | -0.73(-1.02,-0.44) |
| Eastern Sub-Saharan Africa | female | 1514971.00(1144338.77,1936282.65) | 1136905.99(824193.19,1461297.47) | -24.96 | 2592.47(2587.67,2597.27) | 1099.86(1097.50,1102.21) | -2.86(-3.13,-2.60) |
| High-income Asia Pacific | female | 234971.34(171562.31,307891.44) | 100908.22(74082.64,131454.74) | -57.06 | 1413.26(1407.03,1419.51) | 959.50(953.07,965.96) | -0.80(-1.02,-0.58) |
| High-income North America | female | 182871.09(122226.94,263062.59) | 133179.40(95311.06,181591.89) | -27.17 | 603.55(600.71,606.41) | 406.83(404.55,409.13) | -0.65(-1.12,-0.17) |
| North Africa and Middle East | female | 1640805.90(1271396.05,2043414.67) | 1266921.19(942850.98,1617189.46) | -22.79 | 2169.92(2166.19,2173.67) | 1390.08(1387.40,1392.77) | -1.33(-1.68,-0.98) |
| Oceania | female | 36216.48(27308.47,46599.89) | 83559.12(62124.75,107322.16) | 130.72 | 2326.43(2298.92,2354.21) | 2765.08(2743.74,2786.55) | 0.77(0.42,1.12) |
| South Asia | female | 12273849.84(8531439.13,16149610.66) | 6213649.55(4619644.92,8145464.50) | -49.37 | 5107.83(5104.56,5111.10) | 2694.61(2692.26,2696.97) | -1.35(-1.95,-0.74) |
| Southeast Asia | female | 3133398.60(2303666.11,4039209.43) | 1349620.23(991071.33,1761136.87) | -56.93 | 3486.08(3481.72,3490.45) | 1573.49(1570.50,1576.49) | -2.18(-2.59,-1.75) |
| Southern Latin America | female | 50786.44(36747.25,66216.01) | 25022.87(19154.01,31652.64) | -50.73 | 695.22(688.61,701.87) | 376.80(371.74,381.92) | -1.83(-2.06,-1.59) |
| Southern Sub-Saharan Africa | female | 185513.98(131942.62,247180.46) | 96713.89(72796.72,122825.19) | -47.87 | 1588.69(1580.58,1596.83) | 752.86(747.49,758.26) | -2.11(-2.29,-1.92) |
| Tropical Latin America | female | 231271.70(168541.84,308237.24) | 122384.39(90758.66,156846.54) | -47.08 | 859.15(855.29,863.02) | 476.09(473.18,479.01) | -0.63(-1.20,-0.05) |
| Western Europe | female | 244684.20(171439.44,327628.49) | 187124.50(127156.83,256489.63) | -23.52 | 709.59(706.66,712.52) | 553.56(550.97,556.15) | 0.03(-0.33,0.39) |
| Western Sub-Saharan Africa | female | 1817835.08(1338020.64,2344982.10) | 2106787.32(1481256.06,2775261.18) | 15.90 | 3039.66(3034.49,3044.83) | 1609.54(1607.01,1612.07) | -1.58(-2.07,-1.09) |
| Afghanistan | female | 59349.52(41707.11,80157.32) | 94416.98(63527.79,130821.32) | 59.09 | 2380.73(2358.88,2402.74) | 1264.93(1255.97,1273.94) | -2.57(-3.10,-2.05) |
| Albania | female | 12251.25(8152.19,16552.22) | 2959.77(2216.24,3805.42) | -75.84 | 2361.65(2316.56,2407.42) | 1522.76(1465.17,1582.14) | -1.87(-2.26,-1.47) |
| Algeria | female | 111545.60(78327.01,148660.92) | 90296.08(68863.76,114436.87) | -19.05 | 2073.31(2059.81,2086.87) | 1317.38(1307.98,1326.83) | -2.51(-2.87,-2.15) |
| American Samoa | female | 90.86(67.40,115.45) | 47.92(35.36,62.58) | -47.25 | 834.71(655.09,1051.04) | 760.36(537.11,1050.87) | -0.05(-0.38,0.27) |
| Andorra | female | 27.68(18.24,40.34) | 32.60(17.94,57.00) | 17.78 | 625.27(403.50,941.62) | 613.57(411.56,903.21) | 0.92(0.54,1.30) |
| Angola | female | 109842.83(76455.06,141211.09) | 60048.66(38334.15,83273.50) | -45.33 | 3340.27(3316.88,3363.79) | 641.89(635.83,647.98) | -5.11(-5.47,-4.74) |
| Antigua and Barbuda | female | 169.83(128.14,215.94) | 82.39(62.46,105.02) | -51.49 | 1837.58(1540.14,2177.60) | 985.64(765.88,1253.13) | -1.25(-1.75,-0.75) |
| Argentina | female | 40645.11(29080.16,53426.93) | 20156.51(15478.72,25718.96) | -50.41 | 821.23(812.48,830.06) | 434.53(427.97,441.18) | -1.98(-2.24,-1.71) |
| Armenia | female | 4445.44(3048.60,6185.52) | 2615.85(1852.35,3561.73) | -41.16 | 794.47(769.16,820.43) | 1071.06(1028.36,1115.15) | 1.97(1.58,2.35) |
| Australia | female | 9193.25(6456.57,12674.00) | 8627.97(6281.96,11478.51) | -6.15 | 491.24(480.62,502.06) | 376.04(367.72,384.51) | -0.50(-0.72,-0.28) |
| Austria | female | 4448.02(2965.88,6561.16) | 4909.85(2832.00,8860.44) | 10.38 | 681.72(661.01,702.95) | 773.18(751.22,795.67) | 0.59(0.41,0.78) |
| Azerbaijan | female | 26942.51(16748.80,39469.78) | 17232.01(12862.10,22319.77) | -36.04 | 2041.95(2015.38,2068.80) | 1654.64(1627.80,1681.82) | 0.29(-0.25,0.83) |
| Bahamas | female | 238.01(166.91,312.09) | 174.40(132.15,224.49) | -26.73 | 619.48(536.11,712.80) | 501.99(421.07,594.69) | -0.27(-0.63,0.10) |
| Bahrain | female | 1951.99(1519.39,2440.52) | 1926.61(1482.77,2439.34) | -1.30 | 2178.66(2071.38,2290.23) | 1323.12(1260.30,1388.45) | -0.94(-1.34,-0.53) |
| Bangladesh | female | 1437180.96(1140806.97,1739783.09) | 529155.15(422933.44,644130.49) | -63.18 | 4748.96(4739.84,4758.09) | 2395.02(2387.69,2402.38) | -1.53(-1.89,-1.17) |
| Barbados | female | 405.87(296.80,521.04) | 198.12(144.18,262.14) | -51.19 | 1287.13(1147.31,1440.06) | 892.91(756.62,1047.78) | -0.27(-0.73,0.19) |
| Belarus | female | 4934.09(3165.68,7267.79) | 2821.73(1871.90,3962.88) | -42.81 | 463.37(450.11,476.94) | 423.41(407.41,439.91) | -0.01(-0.23,0.21) |
| Belgium | female | 5105.79(3487.11,7165.14) | 4671.37(2953.62,7616.84) | -8.51 | 579.48(562.99,596.37) | 487.88(473.56,502.58) | 0.27(-0.07,0.61) |
| Belize | female | 419.78(284.03,555.86) | 359.60(271.40,488.50) | -14.33 | 1045.97(941.37,1159.42) | 675.01(602.05,754.68) | -0.92(-1.23,-0.62) |
| Benin | female | 41128.85(31094.30,52311.95) | 45444.06(29788.53,59514.21) | 10.49 | 2475.70(2447.76,2503.90) | 1157.82(1145.31,1170.44) | -1.76(-2.20,-1.31) |
| Bermuda | female | 38.83(28.30,51.26) | 23.15(17.09,30.45) | -40.39 | 639.19(441.13,899.95) | 564.26(342.09,887.85) | -0.29(-0.53,-0.04) |
| Bhutan | female | 2702.59(1638.62,3777.78) | 1354.75(944.13,1840.71) | -49.87 | 1795.06(1718.23,1874.59) | 1405.41(1325.76,1488.94) | -0.41(-0.73,-0.10) |
| Bolivia (Plurinational State of) | female | 9938.24(7232.39,13019.90) | 5795.96(4381.27,7546.35) | -41.68 | 636.99(623.08,651.16) | 306.83(298.06,315.81) | -2.33(-2.60,-2.06) |
| Bosnia and Herzegovina | female | 5448.13(3592.52,7819.62) | 2140.89(1516.06,2941.15) | -60.70 | 1052.73(1023.28,1082.86) | 935.66(893.55,979.35) | -0.61(-0.89,-0.34) |
| Botswana | female | 6414.50(4255.17,8741.36) | 5207.89(3844.95,6738.51) | -18.81 | 1799.35(1748.50,1851.37) | 1349.76(1308.35,1392.20) | -0.61(-0.86,-0.36) |
| Brazil | female | 228695.06(166530.83,304833.22) | 119653.28(88623.83,153326.17) | -47.68 | 878.60(874.63,882.58) | 483.88(480.89,486.88) | -0.64(-1.21,-0.06) |
| Brunei Darussalam | female | 748.16(556.92,968.85) | 565.03(426.58,730.23) | -24.48 | 1440.02(1323.73,1564.10) | 1131.92(1029.26,1242.68) | -0.16(-0.43,0.11) |
| Bulgaria | female | 10162.33(6950.38,13837.46) | 13349.27(9715.61,17616.57) | 31.36 | 1057.08(1033.96,1080.62) | 2590.74(2544.68,2637.52) | 3.63(3.06,4.21) |
| Burkina Faso | female | 122820.03(82816.04,158031.49) | 156014.02(103623.90,215564.63) | 27.03 | 3685.95(3661.59,3710.44) | 2219.96(2207.00,2232.98) | -1.64(-2.29,-0.98) |
| Burundi | female | 43797.60(31112.90,55787.88) | 39501.13(29039.96,50294.98) | -9.81 | 2478.73(2451.69,2506.00) | 1036.03(1023.71,1048.46) | -3.73(-4.90,-2.54) |
| Cabo Verde | female | 1438.20(1172.24,1743.17) | 254.28(176.00,335.16) | -82.32 | 1544.10(1454.82,1637.63) | 364.07(315.71,418.10) | -4.52(-4.82,-4.22) |
| Cambodia | female | 137968.61(95287.11,179463.15) | 31971.63(22228.36,42779.57) | -76.83 | 4795.02(4766.33,4823.84) | 1216.04(1200.83,1231.39) | -3.94(-4.39,-3.48) |
| Cameroon | female | 51161.37(37067.93,67976.59) | 58797.14(38359.85,82525.24) | 14.92 | 1547.04(1531.44,1562.77) | 746.34(739.33,753.40) | -1.70(-2.31,-1.08) |
| Canada | female | 12151.49(8629.18,16510.20) | 12102.81(7786.93,21049.60) | -0.40 | 427.32(419.44,435.32) | 392.07(384.81,399.45) | 0.33(0.02,0.64) |
| Central African Republic | female | 11952.45(7051.55,17066.89) | 22823.53(15534.89,29821.54) | 90.95 | 1408.74(1378.54,1439.47) | 1541.05(1517.14,1565.27) | -0.19(-0.87,0.49) |
| Chad | female | 90940.32(65618.62,116527.23) | 185320.96(136567.31,237917.87) | 103.78 | 4175.85(4143.75,4208.15) | 2847.94(2832.46,2863.49) | -0.99(-1.25,-0.72) |
| Chile | female | 7045.39(4938.23,9429.02) | 3498.88(2297.82,4988.14) | -50.34 | 347.72(338.93,356.68) | 195.67(189.06,202.48) | -1.69(-1.79,-1.60) |
| China | female | 2453632.44(1788959.02,3165106.14) | 1081068.36(800417.94,1462819.15) | -55.94 | 1532.17(1530.05,1534.29) | 903.30(901.49,905.12) | -0.79(-1.15,-0.44) |
| Colombia | female | 39903.35(29225.88,49929.28) | 12656.17(9006.58,17562.60) | -68.28 | 645.45(638.49,652.47) | 240.31(235.72,244.97) | -2.30(-2.71,-1.90) |
| Comoros | female | 3421.33(2317.83,4508.19) | 1713.49(1219.68,2275.09) | -49.92 | 2622.94(2521.50,2727.59) | 1316.47(1244.21,1391.95) | -2.20(-2.58,-1.82) |
| Congo | female | 8811.04(5859.30,11636.28) | 7505.73(4548.92,10659.78) | -14.81 | 1395.58(1362.22,1429.58) | 774.28(754.50,794.47) | -1.35(-2.03,-0.66) |
| Cook Islands | female | 23.79(17.62,30.97) | 11.17(8.34,14.67) | -53.07 | 716.30(437.47,1117.24) | 595.58(274.69,1159.02) | -0.20(-0.49,0.10) |
| Costa Rica | female | 3413.01(2325.67,4709.73) | 6048.28(4485.26,7706.11) | 77.21 | 592.34(570.74,614.57) | 1200.62(1168.93,1233.01) | 3.19(2.19,4.21) |
| Croatia | female | 4119.56(2857.92,5497.25) | 2513.53(1693.63,3317.46) | -38.99 | 892.29(862.93,922.44) | 891.05(853.83,929.59) | -0.16(-0.40,0.09) |
| Cuba | female | 8397.53(6300.29,10944.61) | 3302.44(2409.63,4439.40) | -60.67 | 581.28(567.30,595.54) | 406.18(391.25,421.56) | -1.11(-1.41,-0.81) |
| Cyprus | female | 701.29(467.73,996.42) | 580.95(410.10,781.54) | -17.16 | 737.71(681.44,797.78) | 546.50(501.28,594.99) | -0.42(-0.66,-0.18) |
| Czechia | female | 10433.83(7771.68,13055.52) | 5894.11(3910.27,7931.94) | -43.51 | 1024.98(1003.14,1047.19) | 698.54(679.38,718.14) | -0.41(-0.83,0.01) |
| C?te d'Ivoire | female | 73928.51(48002.00,99093.72) | 59317.75(38818.24,83215.99) | -19.76 | 1940.45(1924.14,1956.87) | 829.15(821.29,837.07) | -2.58(-3.20,-1.97) |
| Democratic People's Republic of Korea | female | 108514.16(72135.30,142241.47) | 20640.32(15306.27,27126.61) | -80.98 | 3084.64(3063.56,3105.84) | 920.21(906.22,934.37) | -4.94(-5.75,-4.12) |
| Democratic Republic of the Congo | female | 273253.12(176995.71,378753.28) | 179310.59(113809.90,258282.06) | -34.38 | 2315.12(2304.94,2325.35) | 848.49(843.93,853.08) | -3.19(-4.00,-2.38) |
| Denmark | female | 2853.83(1865.17,4046.33) | 2052.21(1421.73,2997.56) | -28.09 | 657.84(632.72,683.78) | 436.91(417.43,457.15) | -0.79(-1.09,-0.49) |
| Djibouti | female | 4958.01(3666.44,6230.02) | 7076.82(4760.74,9213.85) | 42.74 | 5030.50(4869.76,5195.43) | 3113.56(3029.08,3199.86) | -1.69(-2.22,-1.16) |
| Dominica | female | 89.15(63.58,118.84) | 26.98(19.65,36.21) | -69.74 | 724.03(568.46,910.98) | 466.98(290.28,717.53) | -0.80(-1.10,-0.50) |
| Dominican Republic | female | 9916.36(7331.89,12802.12) | 4698.89(3377.08,6105.50) | -52.61 | 688.80(674.30,703.56) | 303.65(294.14,313.40) | -1.80(-2.05,-1.55) |
| Ecuador | female | 14120.90(9537.63,18997.70) | 10373.56(7040.20,13539.46) | -26.54 | 628.33(616.60,640.24) | 389.25(380.89,397.74) | -1.91(-2.87,-0.94) |
| Egypt | female | 191816.12(144335.14,243211.18) | 224760.31(162563.13,292558.29) | 17.17 | 1577.69(1569.81,1585.60) | 1157.85(1152.53,1163.19) | -0.44(-0.80,-0.08) |
| El Salvador | female | 9953.35(7331.54,12665.59) | 2122.94(1568.05,2764.07) | -78.67 | 841.54(823.38,860.03) | 238.38(227.35,249.82) | -2.93(-3.66,-2.19) |
| Equatorial Guinea | female | 3596.48(2292.50,5142.16) | 1178.89(766.48,1734.53) | -67.22 | 2777.74(2674.56,2884.13) | 449.69(421.48,479.34) | -5.88(-6.26,-5.50) |
| Eritrea | female | 50571.14(37846.90,64397.51) | 32309.23(22848.28,42899.36) | -36.11 | 4834.57(4785.28,4884.27) | 1912.70(1887.04,1938.64) | -3.01(-3.26,-2.76) |
| Estonia | female | 1371.53(908.47,2006.60) | 577.80(419.94,777.67) | -57.87 | 815.94(770.11,863.86) | 563.57(515.74,614.89) | -1.37(-1.56,-1.17) |
| Eswatini | female | 1528.22(1011.91,2127.13) | 826.56(540.27,1115.04) | -45.91 | 635.04(599.38,672.39) | 370.01(342.41,399.37) | -1.72(-2.02,-1.43) |
| Ethiopia | female | 609162.28(428804.57,809598.27) | 293705.00(194268.42,400567.95) | -51.79 | 3999.37(3987.75,4011.02) | 1225.10(1220.03,1230.19) | -3.51(-3.88,-3.14) |
| Fiji | female | 3220.12(2263.58,4164.06) | 1846.37(1266.50,2383.54) | -42.66 | 2228.97(2142.71,2317.93) | 1226.31(1162.68,1292.68) | -1.96(-2.20,-1.72) |
| Finland | female | 3443.52(2360.04,4841.39) | 2368.71(1544.17,3464.25) | -31.21 | 732.26(706.98,758.30) | 571.59(547.71,596.38) | -0.42(-0.65,-0.19) |
| France | female | 32745.44(22611.86,43638.84) | 28546.61(19856.28,47263.40) | -12.82 | 577.93(571.42,584.49) | 487.92(482.08,493.82) | 0.24(-0.04,0.52) |
| Gabon | female | 3095.14(1925.16,4436.58) | 1300.84(898.41,1820.50) | -57.97 | 1176.75(1129.72,1225.37) | 407.40(383.22,432.76) | -2.47(-2.87,-2.07) |
| Gambia | female | 7362.26(5064.53,10001.55) | 6820.63(4913.01,8914.27) | -7.36 | 2301.03(2239.73,2363.67) | 1124.80(1093.20,1157.12) | -1.31(-1.79,-0.82) |
| Georgia | female | 4173.27(2742.98,5713.29) | 1082.42(684.42,1536.30) | -74.06 | 626.86(606.45,647.81) | 301.28(282.42,321.15) | -2.78(-3.21,-2.35) |
| Germany | female | 36737.03(23670.82,52100.60) | 30881.47(17238.56,44881.12) | -15.94 | 573.13(567.04,579.27) | 517.52(511.66,523.45) | 0.16(-0.18,0.50) |
| Ghana | female | 104689.28(81396.41,129093.54) | 82006.93(56981.66,107544.32) | -21.67 | 2271.83(2255.61,2288.15) | 1022.88(1014.59,1031.23) | -2.14(-2.82,-1.45) |
| Greece | female | 4681.28(3069.61,6677.91) | 5895.49(3685.72,11779.73) | 25.94 | 490.52(475.55,505.88) | 835.91(814.03,858.31) | 2.51(2.13,2.89) |
| Greenland | female | 35.03(25.16,46.34) | 16.65(11.75,22.66) | -52.47 | 478.58(323.93,684.27) | 289.73(160.72,486.75) | -0.86(-1.29,-0.44) |
| Grenada | female | 163.94(118.21,216.03) | 59.49(43.79,76.95) | -63.71 | 941.13(789.03,1114.95) | 570.14(419.53,759.43) | -1.08(-1.41,-0.75) |
| Guam | female | 130.08(95.55,168.86) | 97.83(72.71,124.78) | -24.79 | 576.86(473.50,697.18) | 497.86(394.92,621.20) | 0.06(-0.30,0.43) |
| Guatemala | female | 35704.29(30159.29,41397.64) | 6234.40(4721.18,8120.68) | -82.54 | 1569.48(1551.67,1587.45) | 235.69(229.20,242.34) | -6.33(-6.61,-6.04) |
| Guinea | female | 55657.14(33995.48,74725.39) | 60513.95(40605.29,79186.87) | 8.73 | 2747.12(2720.38,2774.09) | 1601.71(1586.76,1616.77) | -1.11(-1.39,-0.83) |
| Guinea-Bissau | female | 9572.92(6657.52,12697.47) | 5591.21(3799.61,7492.09) | -41.59 | 3042.47(2973.07,3113.20) | 1044.06(1012.28,1076.62) | -3.27(-3.61,-2.92) |
| Guyana | female | 4479.93(3394.55,5517.10) | 1118.79(823.61,1436.96) | -75.03 | 2700.16(2610.22,2792.49) | 1002.18(936.37,1071.50) | -2.84(-3.16,-2.52) |
| Haiti | female | 29419.55(22351.37,36681.01) | 25636.37(16934.70,34999.03) | -12.86 | 1703.82(1681.15,1726.74) | 1030.76(1016.30,1045.38) | -2.10(-2.54,-1.65) |
| Honduras | female | 10252.54(8651.74,12165.07) | 4721.53(3542.05,6318.94) | -53.95 | 838.53(820.48,856.88) | 286.42(277.43,295.64) | -3.27(-3.48,-3.05) |
| Hungary | female | 5968.98(3959.50,8295.08) | 3187.89(2118.93,4537.70) | -46.59 | 622.42(605.52,639.71) | 480.39(462.75,498.56) | -0.53(-0.66,-0.40) |
| Iceland | female | 170.30(111.77,235.99) | 173.37(114.45,283.72) | 1.80 | 541.95(460.31,635.36) | 516.07(439.56,603.52) | 0.41(0.11,0.71) |
| India | female | 9691027.41(6534517.75,12835536.94) | 4795704.37(3546476.46,6313085.51) | -50.51 | 5490.32(5486.38,5494.26) | 2995.00(2992.04,2997.96) | -1.32(-1.99,-0.64) |
| Indonesia | female | 1445794.40(981069.05,1958694.60) | 534449.33(371557.46,721296.41) | -63.03 | 4131.93(4124.29,4139.59) | 1591.01(1586.20,1595.84) | -2.48(-3.08,-1.87) |
| Iran (Islamic Republic of) | female | 274021.32(194511.54,361561.07) | 124124.02(94907.10,157444.15) | -54.70 | 2176.12(2167.16,2185.11) | 1319.74(1311.75,1327.77) | -1.34(-1.55,-1.14) |
| Iraq | female | 118382.70(87578.21,150192.63) | 81595.52(61047.68,105010.10) | -31.07 | 2586.64(2570.04,2603.32) | 1258.29(1248.74,1267.91) | -2.60(-3.10,-2.10) |
| Ireland | female | 3161.82(2075.05,4430.08) | 2839.72(1564.38,4813.56) | -10.19 | 689.68(664.64,715.50) | 567.06(545.77,589.08) | -0.01(-0.28,0.27) |
| Israel | female | 4720.55(3158.07,6424.64) | 6382.88(3788.74,10440.88) | 35.21 | 635.86(617.15,655.03) | 494.82(482.45,507.45) | -0.14(-0.46,0.17) |
| Italy | female | 46828.38(32816.84,62708.12) | 25451.07(16525.80,37374.56) | -45.65 | 1054.31(1044.37,1064.34) | 676.47(667.87,685.17) | -0.21(-0.71,0.29) |
| Jamaica | female | 4924.78(3727.94,6213.57) | 1299.64(792.51,1705.10) | -73.61 | 1163.81(1127.89,1200.64) | 487.71(458.27,518.61) | -2.24(-2.53,-1.96) |
| Japan | female | 177422.58(128893.90,232638.87) | 78737.05(57839.28,102805.55) | -55.62 | 1644.89(1636.63,1653.19) | 1081.54(1073.38,1089.75) | -0.85(-1.13,-0.56) |
| Jordan | female | 7426.44(5544.99,9668.07) | 11756.99(8450.88,15187.09) | 58.31 | 854.38(833.02,876.18) | 690.84(677.18,704.71) | -0.53(-0.85,-0.21) |
| Kazakhstan | female | 27744.50(17744.63,39177.80) | 21933.70(13457.94,32817.61) | -20.94 | 1030.79(1017.94,1043.77) | 761.87(751.01,772.86) | -0.34(-1.04,0.37) |
| Kenya | female | 95601.24(63979.62,129339.18) | 63297.55(43479.33,85637.47) | -33.79 | 1450.70(1440.20,1461.25) | 638.83(633.14,644.56) | -2.44(-2.89,-1.99) |
| Kiribati | female | 584.25(437.38,748.11) | 261.04(189.65,342.61) | -55.32 | 3204.42(2918.60,3512.38) | 1152.24(1003.42,1318.48) | -3.66(-3.83,-3.49) |
| Kuwait | female | 3875.18(3003.26,4962.07) | 2612.33(1915.36,3476.52) | -32.59 | 1332.47(1286.47,1379.71) | 647.01(620.25,674.69) | -2.79(-3.22,-2.36) |
| Kyrgyzstan | female | 9934.60(6653.72,13939.31) | 7384.94(5228.79,9966.23) | -25.66 | 1038.40(1015.57,1061.63) | 646.77(630.81,663.04) | -2.04(-2.50,-1.59) |
| Lao People's Democratic Republic | female | 32861.49(22454.40,45313.14) | 11220.03(7306.44,15541.22) | -65.86 | 2943.39(2906.84,2980.30) | 937.14(917.58,957.02) | -3.22(-3.78,-2.66) |
| Latvia | female | 2748.60(1852.89,3691.83) | 866.72(603.38,1163.68) | -68.47 | 966.92(928.15,1006.94) | 624.31(580.80,670.39) | -1.57(-1.74,-1.40) |
| Lebanon | female | 9554.69(6612.95,12416.17) | 7882.23(5972.70,10097.50) | -17.50 | 1705.99(1667.80,1744.86) | 1320.87(1289.44,1352.90) | -0.11(-0.60,0.38) |
| Lesotho | female | 9050.38(6286.43,11614.93) | 2556.23(1823.02,3624.15) | -71.76 | 2508.24(2452.36,2565.14) | 758.41(725.49,792.51) | -4.27(-4.79,-3.75) |
| Liberia | female | 19105.87(13138.13,25279.95) | 10903.02(7532.54,14775.22) | -42.93 | 2434.14(2394.51,2474.31) | 854.70(835.92,873.81) | -3.44(-3.81,-3.06) |
| Libya | female | 16429.51(11571.75,21621.08) | 9337.45(7034.19,11786.91) | -43.17 | 1700.04(1671.19,1729.29) | 1423.44(1391.38,1456.08) | 0.17(-0.14,0.49) |
| Lithuania | female | 4162.38(2715.06,5859.69) | 1082.26(739.51,1451.58) | -74.00 | 1008.62(975.51,1042.61) | 559.57(524.73,596.22) | -1.83(-1.98,-1.68) |
| Luxembourg | female | 191.32(133.50,265.04) | 242.73(170.55,350.24) | 26.87 | 580.14(497.00,674.32) | 491.05(428.99,560.36) | -0.18(-0.42,0.06) |
| Madagascar | female | 84890.03(70882.59,100594.67) | 129135.79(97317.26,159950.64) | 52.12 | 2484.99(2465.75,2504.35) | 1885.54(1873.41,1897.73) | -1.35(-1.61,-1.08) |
| Malawi | female | 56304.94(41319.74,73448.28) | 31436.25(22559.26,41157.28) | -44.17 | 1696.08(1679.90,1712.38) | 666.14(657.46,674.91) | -3.53(-3.94,-3.11) |
| Malaysia | female | 126661.00(99369.57,154443.91) | 79058.63(57936.50,99256.13) | -37.58 | 3540.26(3518.24,3562.38) | 2106.22(2089.70,2122.83) | -1.49(-1.72,-1.26) |
| Maldives | female | 2822.92(2055.49,3580.26) | 693.30(519.27,874.86) | -75.44 | 4572.13(4385.24,4765.21) | 1519.43(1397.33,1649.63) | -3.06(-3.45,-2.67) |
| Mali | female | 119424.29(76520.98,163742.45) | 149194.26(104864.37,197295.16) | 24.93 | 3858.26(3832.51,3884.14) | 1897.06(1885.94,1908.23) | -1.87(-2.27,-1.48) |
| Malta | female | 317.94(212.71,445.00) | 177.94(121.61,254.81) | -44.03 | 761.96(676.89,855.58) | 574.00(490.17,669.10) | -0.53(-0.79,-0.26) |
| Marshall Islands | female | 18.12(13.13,23.80) | 14.55(10.21,19.43) | -19.69 | 169.41(97.19,277.92) | 159.85(86.70,278.40) | 0.25(-0.01,0.51) |
| Mauritania | female | 26159.15(20729.65,32286.67) | 19392.61(14234.76,25849.77) | -25.87 | 4473.86(4411.24,4537.18) | 1909.54(1878.88,1940.59) | -2.05(-2.40,-1.70) |
| Mauritius | female | 5423.01(3904.29,6959.43) | 1792.05(1226.93,2390.91) | -66.95 | 3391.63(3290.08,3495.64) | 1810.30(1717.17,1907.34) | -2.40(-2.73,-2.07) |
| Mexico | female | 229122.66(157203.34,308835.10) | 98060.48(76488.08,121201.16) | -57.20 | 1254.29(1248.58,1260.02) | 638.45(634.12,642.81) | -0.95(-1.49,-0.41) |
| Micronesia (Federated States of) | female | 317.94(236.50,411.82) | 151.32(103.46,200.05) | -52.40 | 1323.40(1165.03,1498.26) | 982.16(810.74,1180.82) | -0.68(-1.02,-0.35) |
| Monaco | female | 9.13(5.46,15.23) | 13.85(7.76,23.00) | 51.76 | 528.03(235.54,1065.00) | 548.78(294.79,958.41) | 0.61(0.26,0.95) |
| Mongolia | female | 4989.27(3056.13,6987.83) | 2221.36(1488.58,3095.65) | -55.48 | 961.11(931.43,991.54) | 402.73(385.06,421.04) | -4.16(-4.61,-3.70) |
| Montenegro | female | 603.00(413.99,827.61) | 434.48(305.17,580.56) | -27.95 | 798.56(731.61,870.27) | 839.34(756.85,928.82) | 0.35(-0.35,1.06) |
| Morocco | female | 66452.96(49930.66,84163.41) | 31355.63(22672.74,41626.73) | -52.82 | 1246.48(1235.81,1257.23) | 654.46(646.52,662.47) | -2.13(-3.03,-1.23) |
| Mozambique | female | 100796.77(70085.64,132196.80) | 56129.85(39760.62,73626.22) | -44.31 | 2437.34(2420.02,2454.76) | 657.48(651.18,663.82) | -4.27(-4.34,-4.20) |
| Myanmar | female | 230610.30(142521.44,319325.92) | 74959.98(49950.79,99485.01) | -67.49 | 2886.89(2873.36,2900.48) | 948.11(940.41,955.86) | -3.24(-3.86,-2.62) |
| Namibia | female | 9255.82(7501.78,11178.91) | 4586.48(2952.34,6209.34) | -50.45 | 2439.44(2381.54,2498.47) | 997.12(964.06,1031.07) | -2.14(-2.60,-1.68) |
| Nauru | female | 16.65(11.73,22.11) | 12.60(9.63,16.54) | -24.34 | 670.68(365.23,1147.80) | 607.27(304.98,1109.19) | -0.31(-0.68,0.07) |
| Nepal | female | 203321.71(146381.45,260370.88) | 59471.28(42702.31,79871.43) | -70.75 | 3678.40(3659.61,3697.27) | 1253.30(1241.76,1264.93) | -2.84(-3.22,-2.46) |
[truncated: 654,020 more chars]
